# Straightforward Superbase-Mediated Reductive O-Phosphorylation of Aromatic and Heteroaromatic Ketones with Red Phosphorus in the Superbase Suspension KOH/DMSO(H_2_O)

**DOI:** 10.3390/molecules30061367

**Published:** 2025-03-18

**Authors:** Vladimir A. Kuimov, Svetlana F. Malysheva, Natalia A. Belogorlova, Ruslan I. Fattakhov, Alexander I. Albanov, Irina Yu. Bagryanskaya, Nikolay I. Tikhonov, Boris A. Trofimov

**Affiliations:** 1 A. E. Favorsky Irkutsk Institute of Chemistry, Siberian Branch, Russian Academy of Sciences, 1 Favorsky Str., Irkutsk 664033, Russia; vladimir_kuimov@irioch.irk.ru (V.A.K.); mal@irioch.irk.ru (S.F.M.); belog@irioch.irk.ru (N.A.B.); fattakhov@irioch.irk.ru (R.I.F.); albanov@irioch.irk.ru (A.I.A.); tikhonov@irioch.irk.ru (N.I.T.); 2 N. N. Vorozhtsov Novosibirsk Institute of Organic Chemistry, Siberian Branch, Russian Academy of Sciences, Novosibirsk 630090, Russia; bagryan@nioch.nsc.ru

**Keywords:** red phosphorus, strong base, bis(benzhydryl)phosphates, benzophenone, single-electron transfer

## Abstract

It was shown for the first time that diaryl(hetaryl)ketones are capable of directly phosphorylating with red phosphorus in the superbase suspension KOH/DMSO(H_2_O) at 85 °C for 1.5 h to afford potassium bis(diaryl(hetaryl)methyl)phosphates that were earlier inaccessible in a yield of up to 45%. The ESR data demonstrate that unlike previously published phosphorylation with elemental phosphorus, this new phosphorylation reaction proceeds via a single electron transfer from polyphospide anions to diaryl(hetaryl)ketones. This is the first example of the C-O-P bond generation during the phosphorylation with elemental phosphorus in strongly basic media, which usually provides C-P bond formation.

## 1. Introduction

Now, it is commonly admitted [1,2,3,4,5,6,7,8,9,10,11] that the methods for the synthesis of organophosphorus compounds (OPCs) from elemental phosphorus (both white and red) and appropriate electrophiles might be potentially of lower cost and less negative ecological impact compared to traditional technologies based on phosphorus chloride [12]. Therefore, the search for and development of chlorine-free syntheses of OPCs still remains a challenge of modern chemistry [13,14,15,16]. White phosphorus (P_4_) is known to be an intermediate (via its chlorides) for the industrial-scale synthesis of phosphorus-containing herbicides, battery electrolytes, pharmaceuticals, detergents, and catalyst ligands [8,17,18,19,20,21]. In recent years, significant advances (on the laboratory level) towards the utilization of P_4_ for the chlorine-free synthesis of value-added OPCs such as secondary and tertiary phosphines and phosphine oxides [22,23] as well as aminophosphonates [24] and tertiary phosphates [25] have been reached. Though red phosphorus (P_red_) is a product of the high-temperature transformation of P_4_, it is considerably less toxic and much safer in handling than its white modification [8,26,27,28]. This makes P_red_ a competitive intermediate in the synthesis of OPCs [29].

Since 1988, we pioneered the systematic study of the activation of P_red_ in simple and available superbase suspensions of the type alkali metal hydroxide/strongly polar complexing solvent (ligand) [30,31] that allowed a number of new environmentally friendly methods for the synthesis of various important OPCs to be developed. Among them are secondary and tertiary phosphines and phosphine oxides, as well as phosphinic and phosphonic acids [29,31]. In multiphase superbase suspensions such as P_red_/KOH/DMSO(H_2_O), three-dimensional phosphorus molecules are disassembled to polyphosphide anions having supernucleophilic properties, which are capable of reacting with various electrophiles (organyl halides [31,32,33], alkenes [31,34,35], acetylenes [31,36,37], and oxiranes [38]) securing the easy and mild formation of the corresponding OPCs. Altogether these syntheses represent an efficient general approach to the direct formation of the C-P bond. It is important that, in no case, the products with С-O-P (phosphates and other phosphorus esters) were formed seemingly due to the proneness of such systems to alkaline hydrolysis.

Meanwhile, OPCs play an important role in life-sustaining activity and human practice. Thus, organic phosphates are widely employed as pesticides [39], extractants [40,41], plasticizers [42], and flame retardants [43,44] due to the successful combination of their physicochemical properties. In our case, it is important to underline that phosphoric acid diesters are utilized as wetting agents [45], antistatics for textiles [46], emulsifiers for cosmetics [47,48], corrosion inhibition in metalworking, cutting and grinding fluids [49], surfactants [50,51], lubricants [52], agrochemical additives [39,53], cleaners formulations [51], organocatalysts [54], in emulsion polymerization reactions [55], etc. For example, commercially available dibenzylphosphate and diphenylphosphate are used as phosphorylating agents [56,57], Brønsted acid catalysts for many reactions [58,59,60].

Organic phosphates play an important role in biological systems [61,62,63,64], since they perform numerous vital functions in living species (DNA, RNA, ATP, etc.) [65,66,67,68,69]. Phosphates such as perifosine, miltefosine, inositol phosphates, and citicoline exhibit antiviral, anticancer, and antiproliferative activities [62].

Currently, for the synthesis of organic phosphates, phosphorus chlorides (PCl_3_ or POCl_3_) [70] or phosphorus(V) oxide or polyphosphoric acid and alcohols [19] are mostly employed. Usually, these methods are not selective and mixtures of mono-, di-, and triphosphates, often difficult to separate, are formed. Phosphates with diarylmethyl (benzhydryl) or triarylmethyl (trityl) substituents remain understudied due to their inaccessibility. There are only two methods for the preparation of bis- and tris(diphenylmethyl)phosphates [71] utilizing diphenyldiazamethane and phosphoric acid or benzophenone hydrazone, (diacetoxyiodo)benzene, and phosphoric acid [72,73,74].

Thus, complementing the chemistry of organic phosphates with new chlorine-free, and hence ecologically neutral (as generating no hazardous waste) phosphorylation technology employing available, inexpensive and safe starting materials and catalysts/mediators (aromatic or heteroaromatic ketones, red phosphorus, KOH, and DMSO) is an awaited task.

All of these facts justify the efforts we spent to elaborate a straightforward method for the synthesis of so far less accessible diesters of phosphoric acids. For this, we took diaryl(hetaryl)ketones as electrophiles of choice, which due to their carbonyl might be involved in the generation of C-O-P bond systems while reacting with P_red_ in strongly basic compositions.

At the same time, according to the known phosphorylation of arylacetylenes stereoselectively providing tris(*Z*-styryl)phosphine [36], the formation of the C-P bond here seemed to be more preferable. In other words, in this case, the synthesis of tris(diphenylhydroxymethyl)phosphine should be expected.

**Scheme 1:**
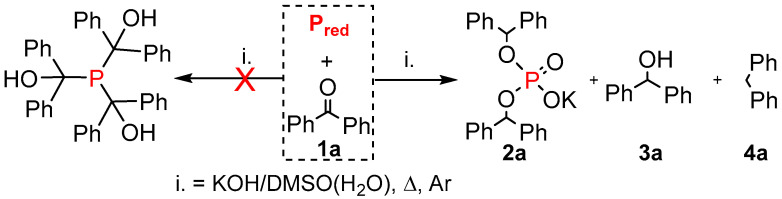
The reaction of diaryl(hetaryl)ketones with red phosphorus.

However, against this expectation, when benzophenone **1a** reacted with red phosphorus under the conditions analogous to those developed for the synthesis of tris(*Z*-styryl)phosphine (60 °C, 1.5 h), we isolated bis(diphenylmethyl)phosphate (**2a**), thereby proving our initial assumption. The synthesis was accompanied by the formation of phosphorus-free products, diphenylcarbinol (**3a**) and diphenylmethane (**4a**), the reduced derivatives of benzophenone (Scheme 1).

Consequently, it was the first event when a compound with the C-О-P bond system was selectively obtained in the reaction of electrophiles with P_red_ in strongly basic conditions.

From structure of phosphate **2a** it followed that we dealt with a new process cardinally different from previously investigated reactions of electrophiles with red phosphorus in multiphase superbase media, which as mentioned above provided exclusively the compounds with a P-C bond [29,31].

This essential difference results from the steric shielding of the carbon atom of the keto group by the ortho-hydrogen of two phenyl substituents and the oxidizing properties of benzophenone itself.

After this first result, it became clear that we encounter a new reaction, which needs to be systematically developed.

## 2. Results and Discussion

We commenced the work with the investigation of the reference reaction between benzophenone **1a** and P_red_ in KOH/DMSO suspension to find the conditions providing the best yield of the target phosphate **2a** (Table 1).

As mentioned above, the second major product was diphenylcarbinol (**3a**) inherently associated with the reaction mechanism (see below mechanistic rationale).

Initially, the synthesis was implemented under the conditions established for the preparation of tris(*Z*-styryl)phosphine [36]. The varying reaction parameters were benzophenone/P_red_/KOH ratio, water content, complexing solvent, temperature, and time, while the controlling (optimizing) functions were the conversion of **1a** and yields of phosphate **2a**, alcohol **3a,** and diphenylmethane **4a**.

Note that, during the process, the elemental phosphorus is partially oxidized mostly to inorganic phosphite and also in a degree to hypophosphite, which are not extractable by organic extractants. This is why the combined amounts of organophosphorus and organic products are always less than 100%, even at a complete conversion of the starting materials.

The experiments show that the most influencing reaction factor was the nature of the solvent and base (for the screening of bases, see Supplementary Materials Section S2.2), which remarkably affects the conversion of **1a**, yields, and ratio of the products. The reaction medium of choice appeared to be the KOH/DMSO [75] suspension, ensuring the best yield of phosphate **2a** and decreasing the amount of **3a** and **4a** (entry 10).

Another reaction parameter governing the outcome of the phosphorylation was the molar ratio of **1a** and P_red_, the optimum of which was found to be 1:3 (entry 10), while the higher or lower content of **1a** gave inferior results (entry 5, 8). Also, the yield of phosphate **2a** noticeably depends on water additives to the reaction mixture: the most suitable result (45% yield) was reached with 21 mmol of water per 73 mmol of P_red_, while a higher amount of additives (167 mmol) of water led to the formation of **2a** in 24% yield (entry 1), but without specially added water this dropped to 39% (entry 13). When commercial DMSO (~2% of water), used in all the experiments, was replaced by dry DMSO, the yield of **2a** was further decreased (31%, entry 14). As seen from entry 11, when **1a** was slowly (dropwise) added to the reaction mixture under the best condition (entry 10), carbinol **3a** was almost cleanly formed (84% yield), whereas the yield of phosphate **2a** dropped to 11%.

**Figure 1:**
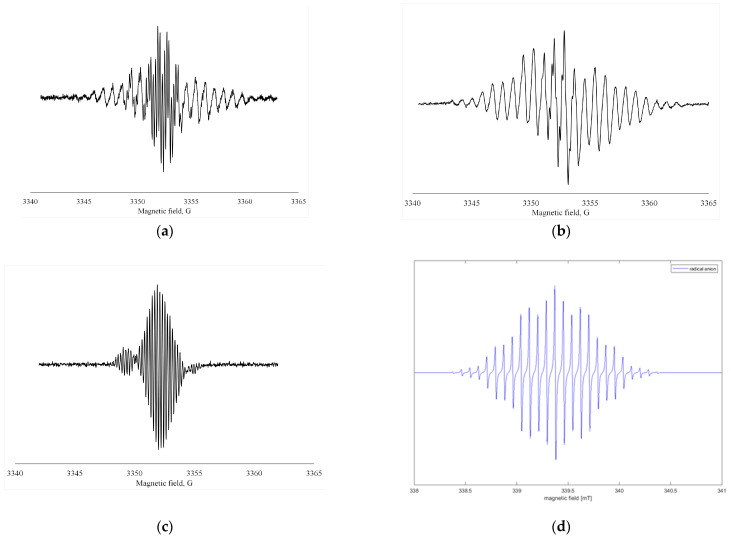
ESR spectra. (**a**) Experimental spectrum of the reaction mixture P_red_/**1a**/KOH/DMSO/H_2_O 5–30 min after the reaction begins. (**b**) Experimental spectrum of the benzophenone anion-radical. (**c**) Experimental spectrum of polyphosphinyl radical. (**d**) Simulated spectrum of the benzophenone anion-radical.

Temperature and time, which covered 60–96 °C and 1.5–6 h, with average values of 85 °C and 1.5 h being optimal, had less effect on the reaction efficiency. The experimental details imply the radical anion character of the process. Indeed, just after the contact of the reactants, the reaction mixture acquired the red color, quickly turning via bright purple to red-brownish, that commonly accompanies the presence of radical ions. In the presence of 10 mol% (relative to **1a**) of typical radical scavengers (hydroquinone, TEMPO, and quinhydrone), the yield of **2a** dropped to 22, 29, and 18%, correspondingly. Since the radical scavengers were taken in amounts much less than equimolar compared to the ketones, they trap only a small part of forming radicals, while a main part of the starting compounds further participate in the major process. In addition, the free-radical species may not come to the solution from anion–radical pairs. In the ESR spectra, typical signals of benzophenone radical anions and signals attributable to the polyphosphinyl radical were observed (Figure 1, see also Supplementary Materials). In the ^31^P NMR spectra of the reaction mixtures obtained in the presence of the above radical scavengers, characteristic signals assignable to intermediate P-C adducts are observed (see Supplementary Materials), implying the initial attack of the carbonyl carbon by a P-centered free radical and subsequent phospha-Brook rearrangement [76,77,78,79] to the final C-O-P products.

Since the initial splitting of the P-P bond by OH^−^ gives P^−^ and H-O-P-terminated species [29,31], the reaction of the latter with diarylcarbinol in a nucleophilic substitution manner might lead to the corresponding C-O-P product. However, the control reaction between carbinol **3a** and P_red_ under the optimum conditions did not produce any amount of phosphate **2a,** thus ruling out such a method for the formation of phosphate **2a**.

**Scheme 2:**
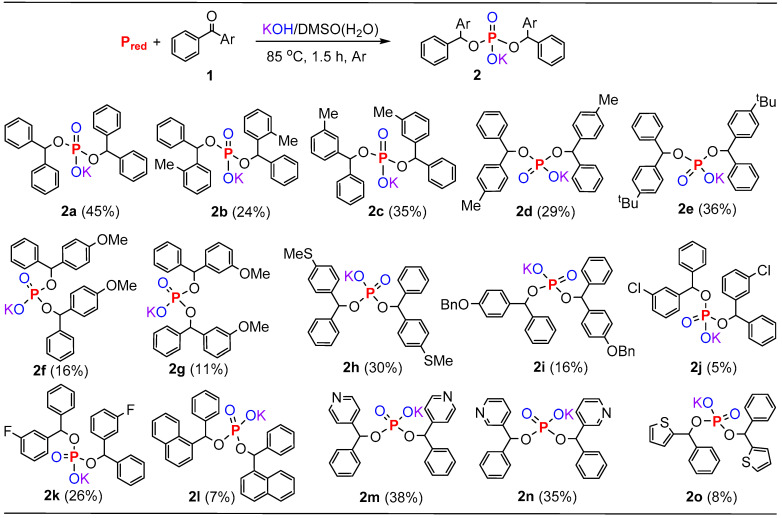
Substrate scope of phosphorylation of diaryl(hetaryl)ketones **1a**–**o** with red phosphorus. Reagents and conditions: P_red_ (73 mmol), **1** (24 mmol), KOH (154 mmol), H_2_O (21 mmol), Ar, 85 °C, 1.5 h.

Next, the substrate scope of this reaction was assessed using a variety of diaryl(hetaryl)ketones, mostly asymmetrical ones (Scheme 2). The yields of phosphates **2** spanned 5–45% and were very sensitive to substitution in the benzene ring and to the nature of the heterocyclic ring attached to the carbonyl group.

However, because of the physical–chemical complexity of this multi-phase process, no clear-cut regularities in the structure/yield relationship can be deduced. Meanwhile, the expected radical anion character of at least one step of the cascade sequence should be facilitated by electron-withdrawing groups due to the decrease in LUMO level, i.e., the higher electron affinity of the intermediates and stabilization of the ion radical intermediate, while electron-donating ones should have an opposite influence. In fact, this is confirmed by the elevated yields of the target phosphates in the case of pyridine ketones **1m**,**n**, which contain π-acceptor substituents (see below).

At the same time, the higher than average yields of the corresponding phosphates (**2c**, **2e**, and **2h**) for ketones with certain electron-donating substituents (3-Me, 4-^t^Bu, and 4-MeS), give a clue that electron-deficient intermediates such as free radicals, formed by the quenching of radical anion with protons, also play an important role in the phosphorylation cascade. The lower yields of phosphates **2f**, **2i**, and **2o** for ketones with electron-donating substituents (4-MeO-, 4-BnO-, and 2-Thiophenyl) are likely due to competition between two steps: (i) the reduction of ketones and (ii) their phosphorylation. Consequently, the more active radical anions generated from the ketones having the donor substituents are rapidly transformed to carbinols **3**, while the phosphorylation becomes the minor direction. Accordingly, the case with two stronger electron-donating groups (i-Pr) in both benzene rings directed the reaction exclusively towards the corresponding carbinol and diarylmethane (see Supplementary Materials). The ketones with stronger π-donors in one of the benzene rings, such as 4-Me_2_N-, 4-OH, 4-C_6_H_13_O, and 4-MeSCH_2_O, did not afford a phosphorylation product, and only in some cases, small amounts of the corresponding carbinols were detectable (^1^H NMR) in the reaction mixtures, which is consistent with the ion-radical character of the process. Although the electron-donating powers of 4-MeO, 4-BnO, and 4-C_6_H_13_O groups are close (σ ≈ −0.25, −0.42, −0.34) [80], the latter long-chain substituent should create impassable spatial encumbrance for the interaction of 3D-oligomeric phosphorus-centered radicals with ketones. In the cases of 4-OVin and 4-O, all substituents in one of the benzene rings, the oligomerization over the double bonds likely prevails, since nether carbinols nor phosphorylated products were formed.

In the case of halo-substituted ketones (**2j** and **2k**), the yields of the target products are decreased because of the reductive elimination of the halogens that leads to the halogen-free phosphate **2a** which was isolated. The ketones with F, Cl, and Br substituents in 2- and 4- positions gave only unsubstituted phosphate **2a** in up to a 37% yield, meaning that the reductive elimination of the halogens takes place during the phosphorylation. The reaction of 2,4-difluorophenyl-(phenyl)ketone proceeds as a nucleophilic substitution of fluorine atom by the phosphinate moiety [32]. Apparently, here the S_RN_1 reaction occurs, involving the initial intermediate radical anions (detected by ESR), which eliminate the halogen anion, followed by the recombination of the radical formed with either the hydrogen or polyphosphinyl radical. Since the S_RN_1 reaction is commonly determined [81,82] as involving radical anions, such a scheme may be considered as justifiable. Thus, this reductive elimination of halogens once again supports the radical anion character of the phosphorylation studied (see Supplementary Materials).

Ketones with electron-rich aromatic heterocyclic substituents (2-Th and 2-Fu) were found to be almost inert in this phosphorylation, which corresponds to their lower activity in single-electron transfer processes (SETs). Consequently, the ketones having π-electron-deficient aromatic substituents (3-Py and 4-Py) such as **1m**,**n** gave yields close to the better ones (35 and 38%). Unfortunately, such functional groups as ester, amide, cyano, nitro, and others, which are sensitive to strongly basic media, do not tolerate the reaction conditions. The condensed aromatic ketones (like 1-naphthyl-phenylketone) mainly undergo reduction to the corresponding carbinol and 1-naphthylphenylmethane (isolated in 51% yield). This results from its susceptibility to the reduction and the anticipated steric shielding of the radical anion center in the initial intermediate by the bulky naphthyl substituents that almost prevents the attack by P-centered free-radicals. This is supported by the fact that Ph-2-MeO-naphthylketone under the aforementioned conditions was not phosphorylated at all, giving the corresponding carbinol in a 32% yield. Expectedly, fatty aromatic ketones were not able to be phosphorylated owing to the domination of the aldol-krotone processes. The testing ketones there are acetophenone, dibenzylketone, ([1,1′-biphenyl]-4-yl)(benzyl)ketone, anthrone, 1,2,3,4-tetrafluoro-9-fluorenone, and 9-fluorenone.

The stability of potassium bis(diphenylmethyl)phosphate was evaluated (KOH/DMSO(H_2_O), 85 °C, 1.5 h): 64% of the initial product was recovered along with 36% of potassium phosphate, diphenylcarbinol, benzophenone, and diphenylmethane, thus indicating the partial solvolysis of the target phosphates **2** under the reaction conditions. Consequently, this, in a degree, lowers the reaction efficiency, but leaves room for a further finer optimization.

**Figure 2:**
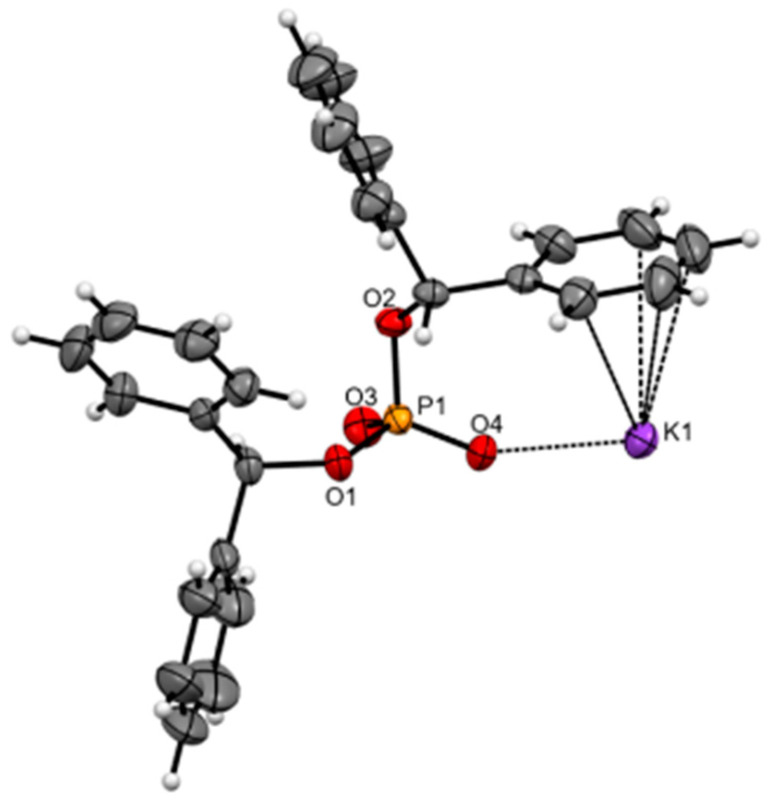
Molecular structure of **2a** (50% thermal ellipsoids are shown), from *n*-hexane-DMF 1:1, CCDC 2366416.

According to the X-ray diffraction data of molecule **2a,** the crystal is situated on the inversion center (Figure 2). XRD study proved the structure of **2a** in which atom K is five coordinate (see Supplementary Materials, Figures S13 and S14) through four K π-arene interactions with the phenyl substituent and four atoms of O phosphate-groups. The K–C(sp^2^) distances are in the range of 3.308(3)–3.385(3) Å [83,84], the distance between the centroid of the Ph ring and K is 3.078 Å, and the K–O distances are 2.944(1), 2.591(1), and 2.575(1) Å for O1, O3, and O4 correspondingly. This coordination of K with O4 leads to four-membered planar metallocycles—K1O4K1O4. The coordination of K with the atom O1 leads to the coordination dimer (Figure S15) and finally, the coordination of K with the atom O3 leads to the resulting structure in the crystal—the chained coordination polymer along the crystallographic axis a (Figure S16).

**Scheme 3:**
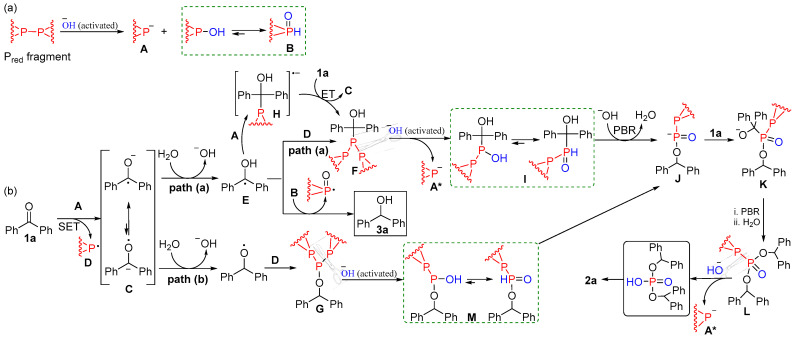
Proposed mechanism. (**a**) The first stage of the reaction is the P-P bond cleavage of the 3D-polymer P_red_ by the superbasic hydroxide-anion; (**b**) the second stage is phosphorylation of benzophenone with polyphosphide anion **A**.

In the light of the above experimental details, the preliminary mechanistic rationale of the C=O bond phosphorylation in aryl(hetaryl)ketones by the superbase P_red_/KOH/DMSO(H_2_O) can be proposed as follows below, though a special theoretical and physical–chemical study is required to establish the true mechanism of this reaction. In any case, the cleavage of the P-P bond of elemental phosphorus by hydroxide anions in superbase media (e.g., KOH/DMSO) to deliver P-centered polyphosphide anions like intermediate **A** (Scheme 3) is now commonly accepted in numerous publications; for references, see reviews [31,85,86]. The aggregation between polar ketone molecules and DMSO may also contribute to the reaction mechanism [87,88]. Meanwhile, the ESR spectral detection of both P-centered and diaryl ketone-derived radical species (Figure 1) as well as the suppression of the reaction by standard radical scavengers (hydroquinone, TEMPO, and quinhydrone), and the characteristic coloration of the reaction mixture may be considered as evidence in favor of the proposed mechanism.

The P-P bond of the 3D-polymer P_red_ is cleaved by the superbasic hydroxide-anion to deliver the polyphospide anion (**A**) and polyphosphinite **B** (Scheme 3a) [31]. Then follows the single-electron transfer (SET) from intermediate **A** to ketones.

This thereby generates radical anion **C** and polyphosphinyl radical **D** (Scheme 3b), which further recombine with the participation of water molecules to give either P-С-bonds (pathway a) or Р-О-С bonds (pathway b, intermediates **F** and **G**, correspondingly). Intermediate **E** can also interact with polyphosphide anion **A** to furnish the new anion-radical **H**; SET from **H** to **1a** renders intermediate **F**. The intermediate **F** undergoes P-P bond cleavage by the OH anion to produce intermediate **I** (P=O) and the new polyphosphide anion (A*). The polyphosphinite anion **J**, formed after the abstraction of a proton from polyphosphinite **I** and phospha-Brook rearrangement [76,77,78,79] (PBR), adds to the next molecule of benzophenone to release alkoxide anion **K**. Then follows the PBR of **K** to polyphosphonate **L**, and after the cleavage of the remaining P-P bond by the hydroxide anion, we arrive at the final phosphorylation product, which is isolable as potassium salt **2a**. The formation of the intermediate P-C bonds (Figures S1–S5) was detected in the ^31^P NMR of the reaction mixture (see Supplementary Materials).

The appearance of diaryl(hetaryl)carbinols is the expected result of the competitive recombination of the initial radical anion **C** with hydrogen radicals. The hydride transfer in the carbinols via the disproportionation [89] gave diaryl(hetaryl)methanes and the corresponding ketones.

**Scheme 4:**
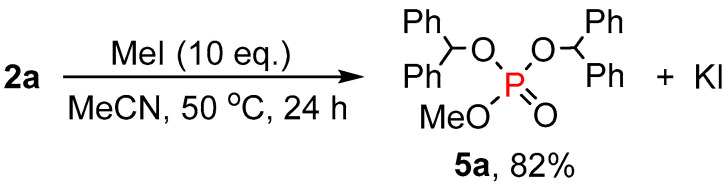
An example of alkylation of potassium phosphates **2**.

The straightforward alkylation of salts **2** may open an easy route to a series of asymmetrical phosphates as exemplified by the methylation of **2a** (MeI, MeCN, 50 °C, 1 day, **5a**), Scheme 4. Consequently, this, in a degree, lowers the reaction efficiency, but leaves room for a further finer optimization.

## 3. Materials and Methods

### 3.1. General Information

All manipulations were carried out under argon atmosphere. ^1^H, ^13^C, ^31^P, and ^19^F NMR spectra were recorded at 400.13, 100.62, 161.98, and 376.50 MHz, respectively, in DMSO or CDCl_3_ solutions with a Bruker (Bruker Corp., Karlsruhe, Germany) DPX-400 and AV-400 spectrometers. Chemical shifts were reported in δ (ppm) relative to DMSO_d_6_ or CDCl_3_ (^1^H and ^13^C) as internal standards or H_3_PO_4_ (^31^P) as an external standard. The assignment of signals in the ^1^Н NMR spectra was made using COSY experiments (XWinNMR ver. 2.6). Resonance signals of carbon atoms were assigned based on ^1^H-^13^C HSQC and ^1^H-^13^C HMBC experiments (XWinNMR ver. 2.6). IR spectra were run on a Vertex 70 (Bruker Optics GmbH, Karlsruhe, Germany) spectrometer. The EI mass spectra were obtained on a Shimadzu (KRATOS Analytical Ltd., Manchester, UK) GSMS-QP5050 mass spectrometer. Melting points were established using a Kofler micro hot-stage apparatus (Wagner&Munz GmbH, München, Germany) and are uncorrected. Commercially available red phosphorus (KSAN Sia, Riga, Latvia) was purified by consecutive washing with aq. NaOH (1–2%), H_2_O, EtOH, and Et_2_O to remove all acidic impurities, dried in vacuum at 25–30 °C to a constant weight, and stored under inert atmosphere (N_2_). Commercially available undried DMSO (~1% of water) was used (Vekton, St. Petersburg, Russia). Commercially available potassium hydroxide (from Sigma-Aldrich, Saint Louis, MO, USA) of composition KOH (~85%) and H_2_O (≤15%) that almost exactly corresponds to the formula KOH∙0.5H_2_O [90,91] was used without further purification. The water content in KOH∙0.5H_2_O was determined using moisture analyzer (HB43-S Halogen, METTLER TOLEDO, Powai, Mumbai, India). Most of the diaryl(hetaryl)ketones are commercial products: **1a**–**f,j,l**–**o,** 9H-fluoren-9-one, and anthracen-9(10H)-one. Other ketones (3-Methoxybenzophenone (**1g**), (4-(methylthio)phenyl)(phenyl)methanone (**1h**), benzyloxybenzophenone (**1i**), 3-fluorobenzophenone (**1k**), 1-naphthyl(phenyl)methanone (**1l**), 2-benzoylfurane (**1p**), and 2-methoxynaphthalen-1-yl(phenyl)methanone) were synthesized according to the literature methods (see Supplementary Materials). The EPR spectra of the samples were recorded with the ELEXSYS E-580 spectrometer (Bruker BioSpin GmbH, Karlsruhe, Germany), X-band 9.7 GHz. Raman spectra were obtained on an ATP 8900Ad Raman vacuum Fourier transform infrared spectrometer (Optosky Photonics, Xiamen, China), 785 and 1064 nm. The C, H microanalyses were performed on a Flash 2000 CHNS analyzer (Thermo Fisher Scientific, Delft, The Netherlands).

### 3.2. Synthetic Procedures

#### 3.2.1. Procedure for the Preparation of the Potassium Phosphate **2a**

(1). A 250 mL round-bottom flask was sequentially charged with red phosphorus (2.25 g, 73 mmol), ketone **1a** (24 mmol), DMSO (50 mL), freshly machine-powdered KOH·0.5H_2_O (10.00 g, 154 mmol), and H_2_O (0.38 g, 21 mmol) and flushed with argon from the balloon. The flask was sunk into a preliminarily heated oil bath and the reaction mixture was stirred (500–700 rpm) for 1.5 h at 84–85 °C. After the reaction completion, the flask was cooled down to r.t., one part of water (25 mL) was added, and the reaction mixture was transferred into a separation funnel. A separated aqueous basic layer (~18 g), consisting of inorganic phosphates and KOH, was discarded. To the remaining aqueous organic layer, another portion of water (25 mL) and CHCl_3_ (25 mL) were added. The CHCl_3_ layer was separated and the aqueous organic layer was further extracted twice with CHCl_3_ (2 × 25 mL). Therefore, the CHCl_3_ extract contains DMSO, the target product **2a**, carbinol **3a**, and diphenylmethane **4a**. After the evaporation of CHCl_3_ and DMSO (3 mmHg, 50 °C), **3a** and **4a** are consequentially washed out by the diethyl ether (3 × 15 mL) and chloroform (2 × 2 mL). The residue was dried in vacuum to give 2.53 g (45%) of the phosphate **2a**.

*Potassium bis(diphenylmethyl)phosphate* (**2a**). Yield: 2.53 g (45%), clear crystals, mp 293 °C (hexane/DMF). ^1^H NMR (400.13 MHz, DMSO_d_6_): δ = 6.08 (d, ^3^*J*_POCH_ = 10.1 Hz, 2H, POCH), 7.13–7.20 (m, 20 H, H*_o,m,p_* in Ph) ppm. ^13^C NMR (100.62 MHz, DMSO_d_6_): δ = 77.1 (d, ^2^*J*_PC_ = 5.2 Hz, POCH), 126.3 (C*_p_* in Ph), 126.5 (C*_o_* in Ph), 127.7 (C*_m_* in Ph), 144.5 (d, ^3^*J*_PC_ = 4.2 Hz, C*_i_* in Ph) ppm. ^31^P NMR (161.98 MHz, DMSO_d_6_): δ = −0.17 (t, ^3^*J*_POCH_ = 10.1 Hz) ppm. IR (KBr): 3435, 3028, 3058, 2932, 1657, 1599, 1493, 1453, 1261, 1188, 1103, 1008, 884, 859, 707, 573, 487 сm^−1^. Raman (785 nm): 219, 272, 620, 648, 748, 841, 1004, 1189, 1352, 1604, 3060 сm^−1^. Anal. Calcd. for C_26_H_22_ KO_4_P (m.w. 468.52): C, 66.65; H, 4.73. Found: C, 64.68; H, 5.09. This salt is very soluble in DMSO, DMF, MeOH, EtOH, and ^i^PrOH, but sparingly soluble in MeCN, dioxane, THF, DME, and C_6_H_6_ and very poorly soluble in CHCl_3_, DCM, ethyl acetate, acetone, H_2_O, Et_2_O, and CCl_4_.

#### 3.2.2. General Procedure for the Preparation of the Potassium Phosphates **2m**,**n**

A mixture of red phosphorus (2.25 g, 73 mmol), freshly machine-powdered KOH·0.5H_2_O (10.00 g, 154 mmol), H_2_O (0.38 g, 21 mmol), and phenylpyridylketone (**1m**,**n**, 24 mmol) in 50 mL of DMSO was stirred for 1.5 h at 84–85 °C (oil bath). After the reaction completion, one portion of water (25 mL) was added, and the aqueous basic layer (~13–18 g) was separated and discarded (consisting of non-organic phosphates and KOH). The reaction mixture was diluted with another portion of water (25 mL) and extracted with chloroform (3 × 25 mL); chloroform was dried on CaCl_2_ and removed to obtain the corresponding phenylpyridylcarbinol (48–52%). The remaining aqueous layer was carefully acidified with aq. HCl (18%) up to pH ~7, and water and DMSO were removed in vacuum. The resulting solid mass was washed with CHCl_3_ (3 × 15 mL), chloroform was removed, and the residue was dried in vacuum to give phosphate **2m**,**n** as wax masses in a yield of 35–38%.

*Potassium bis[(2-methylphenyl)(phenyl)methyl]phosphate* (**2b**). Following the general procedure, **2b** was prepared from **1b** (24 mmol, 4.71 g). **2b** was isolated as white powder (1.43 g, 24% yield), mp 264–266 °C. ^1^H NMR (400.13 MHz, DMSO_d_6_): δ = 2.03, 2.04 (s, 6 H, CH_3_), 6.20, 6.22 (d, ^3^*J*_POCH_ = 10.2 Hz, 2 H, POCH), 6.98–7.02 (m, 2 H, H-3 in C_6_H_4_), 7.07–7.17 (m, 6 H, H-4,5,6 in C_6_H_4_ and 8 H, H*_o_*_,*m*_ in Ph), 7.31–7.36 (m, 2 H, H*_p_* in Ph) ppm. ^13^C NMR (100.62 MHz, DMSO_d_6_): δ = 19.2 (CH_3_), 74.6 (d, ^2^*J*_PC_ = 4.7 Hz, POCH), 125.5 (C-5 in C_6_H_4_), 126.4, 126.5 (C-4 in C_6_H_4_), 126.5 (C*_p_*, in Ph), 127.1, 127.2 (C*_o_* in Ph), 127.1, 127.2 (C-6 in C_6_H_4_), 127.6 (C*_m_* in Ph), 129.8 (C-3 in C_6_H_4_), 134.4 (d, ^4^*J*_PC_ = 1.6 Hz, C-2 in C_6_H_4_), 142.0, 142.1 (d, ^3^*J*_PC_ = 3.3 Hz, C-1 in C_6_H_4_), 143.4, 143.5 (d, ^3^*J*_PC_ = 4.6 Hz, C*_i_* in Ph) ppm. ^31^P NMR (161.98 MHz, DMSO_d_6_): δ = −0.05 (t, ^3^*J*_POCH_ = 10.1 Hz) ppm. IR (KBr): 3435, 3027, 2921, 1608, 1493, 1453, 1244, 1156, 1103, 1019, 933, 875, 710, 625, 578, 509 сm^−1^. Raman (1064 nm): 339, 624, 795, 847, 1000, 1028, 1102, 1185, 1611, 3059 cm^−1^. Anal. Calcd for C_28_H_26_KO_4_P (m.w. 496.58): C, 67.72; H, 5.28. Found: C, 67.38; H, 5.18.

*Potassium bis[(3-methylphenyl)(phenyl)methyl]phosphate* (**2c**). Following the general procedure, **2c** was prepared from **1c** (24 mmol, 4.71 g). **2c** was isolated as white powder (2.09 g, 35% yield), mp 220–222 °C. ^1^H NMR (400.13 MHz, DMSO_d_6_): δ = 2.20 (s, 6 H, CH_3_), 6.03 (d, ^3^*J*_POCH_ = 9.9 Hz, 2 H, POCH), 6.94–7.20 (m, 18 H, H-2,4,5,6 in C_6_H_4_ and H*_m,o,p_* in Ph) ppm. ^13^C NMR (100.62 MHz, DMSO_d_6_): δ = 21.2 (CH_3_), 77.24 (d, ^2^*J*_PC_ = 5.0 Hz, POCH), 123.7, 123.8 (C-6 in C_6_H_4_), 126.4 (C*_p_*, in Ph), 126.5, 126.5 (C*_o_* in Ph), 127.1 (C-4 in C_6_H_4_), 127.1 (C-2 in C_6_H_4_), 127.7 (C-5 in C_6_H_4_), 127.8 (C*_m_* in Ph), 136.7 (C-3 in C_6_H_4_), 144.3 (d, ^3^*J*_PC_ = 3.8 Hz, C-1 in Ph) 144.4 (d, ^3^*J*_PC_ = 4.1 Hz, C*_i_* in Ph) ppm. ^31^P NMR (161.98 MHz, DMSO_d_6_): δ = −0.32 (t, ^3^*J*_POCH_ = 9.8 Hz) ppm. IR (KBr): 3434, 3059, 3060, 3027, 2920, 1606, 1491, 1452, 1243, 1194, 1155, 1100, 1057, 1018, 933, 914, 875, 795, 772, 709, 654, 624, 578, 511, 452 сm^−1^. Raman (785 nm): 224, 273, 527, 618, 652, 736, 780, 847, 1002, 1107, 1195, 1238, 1380, 1448, 1604, 3061 cm^−1^. Anal. Calcd for C_28_H_26_KO_4_P (m.w. 496.58): C, 67.72; H, 5.28. Found: C, 67.61; H, 4.97.

*Potassium bis[(4-methylphenyl)(phenyl)methyl]phosphate* (**2d**). Following the general procedure, **2d** was prepared from **1d** (24 mmol, 4.71 g). **2d** was isolated as white powder (1.73 g, 29% yield), mp 234–236 °C. ^1^Н (DMSO_d_6_): δ = 2.23 (s, 6 H, CH_3_), 6.03 (d, ^3^*J*_POCH_ = 10.0 Hz, 2 H, POCH), 6.96–6.98 (m, 4 H, H-3,5 in C_6_H_4_), 7.05–7.07 (m, 4 H, H-2,6 in C_6_H_4_), 7.12–7.18 (m, 10 H, H*_o_,_m_,_p_* in Ph) ppm. ^13^C NMR (100.62 MHz, DMSO_d_6_): δ = 20.8 (CH_3_), 77.0 (d, ^2^*J*_PC_ = 5.1 Hz, POCH), 126.3 (C*_p_* in Ph), 126.5 (C*_o_* in Ph), 126.6 (d, ^4^*J*_PC_ = 2.6 Hz, C-2,6 in C_6_H_4_), 127.7 (d, ^5^*J*_PC_ = 0.9 Hz, C*_m_* in Ph), 128.3 (d, ^5^*J*_PC_ = 1.3 Hz, C-3,5 in C_6_H_4_), 135.3 (d, ^6^*J*_PC_ = 1.8 Hz, C-4 in C_6_H_4_), 141.5 (d, ^3^*J*_PC_ = 4.1 Hz, C-1 in C_6_H_4_), 144.7 (d, ^3^*J*_PC_ = 4.5 Hz, C*_i_* in Ph) ppm. ^31^P NMR (161.98 MHz, DMSO_d_6_): δ = −0.51 (t, ^3^*J*_POCH_ = 9.9 Hz) ppm. IR (KBr): 3435, 3030, 2923, 1631, 1513, 1453, 1255, 1190, 1098, 1004, 881, 897, 572 сm^−1^. Raman (785 nm): 337, 497, 645, 798, 849, 1004, 1029, 1105, 1187, 1382, 1454, 1611, 3061 cm^−1^. Anal Calcd for C_28_H_26_KO_4_P (m.w. 496.58): C, 67.72; H, 5.28. Found: C, 67.62; H, 5.17.

*Potassium bis[(4-tert-butylphenyl)(phenyl)methyl]phosphate* (**2e**). Following the general procedure, **2e** was prepared from **1e** (24 mmol, 5.72 g). **2e** was isolated as white powder (2.51 g 36% yield), mp 263–265 °C. ^1^H NMR (400.13 MHz, DMSO_d_6_): δ = 1.24 (s, 18 H, CH_3_), 6.03 (d, ^3^*J*_POCH_ = 10.0 Hz, 2 H, POCH), 7.10–7.21 (m, 8 H in C_6_H_4_ and 10 H in Ph) ppm. ^13^C NMR (100.62 MHz, DMSO_d_6_): δ = 31.3 (CH_3_), 34.1 [*C*(CH_3_)_3_], 77.0 (d, ^2^*J*_PC_ = 4.8 Hz, POCH), 124.5 (C-2,6 in C_6_H_4_), 126.29 (C*_p_* in Ph), 126.3 (C-3,5 in C_6_H_4_), 126.5 (C*_o_* in Ph), 127.7 (C*_m_* in Ph), 141.6 (d, ^3^*J*_PC_ = 3.6 Hz, C*_i_* in Ph), 144.7 (d, ^3^*J*_PC_ = 3.7 Hz C-1 in C_6_H_4_), 148.6 (C-4 in C_6_H_4_) ppm. ^31^P NMR (161.98 MHz, DMSO_d_6_): δ = −0.15 (t, ^3^*J*_POCH_ = 9.9 Hz) ppm. IR (KBr): 3421, 3061, 2983, 1639, 1513, 1454, 1410, 1363, 1231, 1100, 1007, 891, 861, 804, 704, 626, 580, 528 сm^−1^. Anal. Calcd for C_34_H_38_KO_4_P (m.w. 580.73): C, 70.32; H, 6.60. Found: C, 69.96; H, 6.64.

*Potassium bis[(4-methoxyphenyl)(phenyl)methyl]phosphate* (**2f**). Following the general procedure, **2f** was prepared from **1f** (24 mmol, 5.09 g). **2f** was isolated as white powder (1.00 g, 16% yield), mp 195–196 °C. ^1^H NMR (400.13 MHz, DMSO_d_6_): δ = 3.69 (s, 6 H, OCH_3_), 6.05 (d, ^3^*J*_POCH_ = 10.0 Hz, 2 H, POCH), 6.72–6.75 (m, 4 H, H-3,5 in C_6_H_4_), 7.08–7.11 (m, 4 H, H-2,6 in C_6_H_4_), 7.13–7.19 (m, 10 H in Ph) ppm. ^13^C NMR (100.62 MHz, DMSO_d_6_): δ = 55.2 (OCH_3_), 77.2 (d, ^2^*J*_PC_ = 4.4 Hz, POCH), 113.4 (C-3,5 in C_6_H_4_), 126.7 (C*_p,o_* in Ph), 128.0 (C-2,6 in C_6_H_4_), 128.1 (C*_m_* in Ph), 136.5 (C-1 in C_6_H_4_), 144.6 (C*_i_* in Ph), 158.2 (C-4 in C_6_H_4_) ppm. ^31^P NMR (161.98 MHz, DMSO_d_6_): δ = −0.69 (t, ^3^*J*_POCH_ = 9.9 Hz) ppm. IR (KBr): 3434, 3029, 2934, 2837, 1611, 1512, 1453, 1252, 1175, 1098, 1003, 926, 887, 785, 703, 576 сm^−1^. Anal Calcd for C_28_H_26_KO_6_P (m.w. 528.57): C, 63.62; H, 4.96. Found: C, 63.42; H, 4.93.

*Potassium bis[(3-methoxyphenyl)(phenyl)methyl]phosphate* (**2g**). Following the general procedure, **2g** was prepared from **1g** (24 mmol, 5.09 g). **2g** was isolated as white powder (0.70 g, 11% yield), mp 156–157 °C. ^1^H NMR (400.13 MHz, DMSO_d_6_): δ = 3.65 (s, 6 H, OCH_3_), 6.01 (d, ^3^*J*_POCH_ = 10.1 Hz, 2 H, POCH), 6.68 (dt, ^3^*J*_4–5_ = 8.4 Hz, ^4^*J*_4–2_~^4^*J*_4–6_ = 2.5 Hz, 2H, H-4 in C_6_H_4_), 6.74 (d, ^3^*J*_6–5_ = 7.7 Hz, 2H, H-6 in C_6_H_4_), 6.79 (br.s, 2 H, H-2 in C_6_H_4_), 7.08 (ddd, ^3^*J*_5–6_ = 7.7 Hz, ^3^*J*_5–4_ = 8.4 Hz, ^5^*J*_5–2_ = 2.1 Hz, 2H, H-5 in C_6_H_4_), 7.10–7.18 (m, 10 H in Ph) ppm. ^13^C NMR (100.62 MHz, DMSO_d_6_): δ = 54.9 (OCH_3_), 77.1 (d, ^2^*J*_PC_ = 4.8 Hz, POCH), 111.8 (C-4 in C_6_H_4_), 112.2 (d, ^4^*J*_PC_ = 4.3 Hz, C-2), 118.8 (C-6 in C_6_H_4_), 126.4 (C*_p_*, in Ph), 126.5 (C*_o_* in Ph), 127.7 (C*_m_* in Ph), 128.8 (C-5 in C_6_H_4_), 144.4 (d, ^3^*J*_PC_ = 4.5 Hz, C*_i_* in Ph), 146.0 (d, ^3^*J*_PC_ = 4.5 Hz, C-1 in C_6_H_4_), 158.9 (C-3 in C_6_H_4_) ppm. ^31^P NMR (161.98 MHz, DMSO_d_6_): δ = −0.64 (t, ^3^*J*_POCH_ = 10.1 Hz) ppm. IR (KBr): 3432, 3027, 2920, 2835, 1601, 1490, 1242, 1157, 1103, 1021, 879, 776, 694, 599, 519 сm^−1^. Anal. Calcd for C_28_H_26_KO_6_P (m.w. 528.57): C, 63.62; H, 4.96. Found: C, 63.22; H, 4.92.

*Potassium bis{[4-(methylthio)phenyl](phenyl)methyl}phosphate* (**2h**). Following the general procedure, **2h** was prepared from **1h** (24 mmol, 5.48 g). **2h** was isolated as white powder (2.01 g, 30% yield), mp 158–159 °C. ^1^H NMR (400.13 MHz, CDCl_3_): δ = 2.39, 2.40 (s, 6 H, SCH_3_), 6.00, 6.02 (d, ^3^*J*_POCH_ = 9.3 Hz, 2 H, POCH), 7.04–7.21 (m, 8 H in C_6_H_4_, 10 H in Ph) ppm. ^13^C NMR (100.62 MHz, CDCl_3_): δ = 15.6 (SCH_3_), 78.3 (d, ^2^*J*_PC_ = 4.1 Hz, POCH), 126.1, 126.2 (C*_o_* in Ph), 126.6, 126.7 (C-3,5 in C_6_H_4_), 127.0, 127.1 (C-2,6 in C_6_H_4_), 127.3, 127.3 (C*_p_* in Ph), 128.4, 128.4 (C*_m_* in Ph), 137.3 (C-4 in C_6_H_4_), 139.1, 139.3 (C-1 in C_6_H_4_), 142.8, 143.0 (C*_i_* in Ph) ppm. ^31^P NMR (161.98 MHz, CDCl_3_): δ = −0.61 (br.t) ppm. IR (KBr): 3436, 3027, 2918, 1959, 1905, 1700, 1600, 1493, 1403, 1242, 1100, 1006, 886, 795, 701, 623, 571 сm^−1^. Anal. Calcd for C_28_H_26_KO_4_PS_2_ (m.w. 560.71): C, 59.98; H, 4.67. Found: C, 60.42; H, 4.91.

*Potassium bis[[4-(benzyloxy)phenyl](phenyl)methyl]phosphate* (**2i**). Following the general procedure, **2i** was prepared from **1i** (24 mmol, 6.92 g). **2i** was isolated as white powder (0.90 g, 16% yield), mp 244 °C. ^1^H NMR (400.13 MHz, DMSO_d_6_): δ = 5.03 (s, 4 H, OCH_2_), 6.00 (d, ^3^*J*_POCH_ = 10.1 Hz, 2 H, POCH), 6.80 (d, ^3^*J*_HH_ = 8.4 Hz, 4 H, H-3,5 in C_6_H_4_), 7.05 (d, ^3^*J*_HH_ = 8.0 Hz, 4 H, H-2,6 in C_6_H_4_), 7.11–7.16 (m, 10 H, H*_o,m,p_* in PhCH), 7.31–7.43 (m, 10 H, H*_o,m,p_* in PhCH_2_) ppm. ^13^C NMR (100.62 MHz, DMSO_d_6_): δ = 69.2 (OCH_2_), 76.8 (d, ^2^*J*_PC_ = 5.1 Hz, POCH), 114.0 (C-3,5 in C_6_H_4_), 126.3 (C*_p_* in PhCH), 126.5 (C*_o_* in PhCH), 127.6 (C*_o_* in PhCH_2_), 127.7 (C*_m_* in PhCH), 127.8 (C*_p_* in PhCH_2_), 127.9 (C-2,6 in C_6_H_4_), 128.5 (C*_m_* in PhCH_2_), 136.9 (C-1 in C_6_H_4_), 137.3 (C*_i_* in PhCH_2_), 144.8 (C*_i_* in PhCH), 157.1 (C-4 in C_6_H_4_) ppm. ^31^P NMR (161.98 MHz, DMSO_d_6_): δ = −0.21 (t, ^3^*J*_POCH_ = 10.0 Hz) ppm. IR (KBr): 3430, 2301, 1613, 1512, 1454, 1399, 1247, 1175, 1099, 1001, 888, 897, 574 сm^−1^. Anal. Calcd for C_40_H_34_ O_6_P (m.w. 468.52): C, 70.57; H, 5.03. Found: C, 70.28; H, 5.09.

*Potassium bis[(3-chlorophenyl)(phenyl)methyl]phosphate* (**2j**). Following the general procedure, **2j** was prepared from **1j** (24 mmol, 5.20 g). **2j** was isolated as white powder (0.32 g, 5% yield), mp 198 °C. ^1^H NMR (400.13 MHz, DMSO_d_6_): δ = 6.07 (d, ^3^*J*_POCH_ = 9.9 Hz, 2 H, POCH), 7.14–7.26 (m, 10 H, in Ph and 8 H in C_6_H_4_) ppm. ^13^C NMR (100.62 MHz, DMSO_d_6_): δ = 76.5 (d, ^2^*J*_PC_ = 4.7 Hz, POCH), 125.0, 125.1 (C-6 in C_6_H_4_), 126.1 (C*_p_*, in Ph), 126.5 (C*_o_* in Ph), 126.7 (C-2 in C_6_H_4_), 127.8 (C-4 in C_6_H_4_), 127.9 (C*_m_* in Ph), 129.7 (C-5 in C_6_H_4_), 132.7 (C-3 in C_6_H_4_), 143.6 (d, ^3^*J*_PC_ = 4.3 Hz, C*_i_* in Ph), 146.8 (d, ^3^*J*_PC_ = 4.2 Hz, C-1 in C_6_H_4_) ppm. ^31^P NMR (161.98 MHz, DMSO_d_6_): δ = −1.37 (t, ^3^*J*_POCH_ = 10.1 Hz) ppm. IR (KBr): 2922, 2833, 2258, 2129, 1651, 1495, 1454, 1292, 1229, 1029, 1025, 886, 826, 764, 701, 574 сm^−1^. Anal. Calcd for C_26_H_20_Cl_2_KO_4_P (m.w. 537.41): C, 58.11; H, 3.75. Found: C, 58.02; H, 3.71.

*Potassium bis[(3-fluorophenyl)(phenyl)methyl]phosphate* (**2k**). Following the general procedure, **2k** was prepared from **1k** (24 mmol, 4.80 g). **2k** was isolated as white powder (1.57 g, 26% yield), mp 236–238 °C. ^1^H NMR (400.13 MHz, DMSO_d_6_): δ = 6.07 (d, ^3^*J*_POCH_ = 10.0 Hz, 2 H, POCH), 6.92 (t, ^3^*J*_4–5_~^3^*J*_4-F_ = 8.5 Hz, 2 H, H-4 in C_6_H_4_), 7.00 (d, 4 H, ^3^*J*_2-F_~^3^*J*_6–5_ = 7.8 Hz, H-2,6 in C_6_H_4_), 7.15–7.20 (m, 12 H, *H_o,m,p_* in Ph, H-5 in C_6_H_4_) ppm. ^13^C NMR (100.62 MHz, DMSO_d_6_): δ = 76.6 (d, ^2^*J*_PC_ = 4.6 Hz, POCH), 113.0, 113.2 (d, ^2^*J*_CF_ = 20.2 Hz, C-2,4 in C_6_H_4_), 122.4 (C-6 in C_6_H_4_), 126.5 (C*_o_* in Ph), 126.8 (C*_p_*, in Ph), 127.9 (C*_m_* in Ph), 129.7 (d, ^3^*J*_CF_ = 8.2 Hz, C-5 in C_6_H_4_), 143.8 (C*_i_* in Ph), 147.3 (C-1 in C_6_H_4_), 162.0 (d, ^1^*J*_CF_ = 242.9 Hz, C-3 in C_6_H_4_) ppm. ^31^P NMR (161.98 MHz, DMSO_D6): δ = −0.96 (t, ^3^*J*_POCH_ = 9.9 Hz) ppm. ^19^F NMR (376.50 MHz, DMSO_d_6_): δ = −113.42 ppm. IR (KBr): 3031, 2919, 2850, 1592, 1450, 1245, 1103, 1018, 941, 874, 791, 706, 522 сm^−1^. Anal. Calcd for C_26_H_20_F_2_KO_4_P (m.w. 504.50): C, 61.90; H, 4.00. Found: C, 61.28; H, 3.99.

*Potassium bis(naphthalen-1-yl(phenyl)methyl)phosphate* (**2l**). Following the general procedure, **2l** was prepared from **1l** (24 mmol, 5.57 g). **2l** was isolated as light beige crystalline solids (0.48 g, 7% yield), mp 176–178 °C. ^1^H NMR (400.13 MHz, DMSO_d_6_): δ = 6.73, 6.72 (d, ^3^*J*_POCH_ = 10.1 Hz, 2 H, POCH), 7.06–7.09 (m, 6 H, *H_m,p_* in Ph), 7.11–7.16 (m, 4 H, *H_o_* in Ph), 7.31 (br.t, ^3^*J*_3–2_~^3^*J*_3–4_ = 7.5 Hz, 2 H, H-3 in C_5_H_4_), 7.32 (t, ^3^*J*_6–7_~^3^*J*_6–5_ = 8.2 Hz, 2 H, H-6 in C_5_H_4_), 7.41 (t, ^3^*J*_7–6_~^4^*J*_7–8_ = 8.2 Hz, 2 H, H-6 in C_5_H_4_), 7.49 (d, ^3^*J*_2–4_ = 7.5 Hz, 2 H, H-2 in C_5_H_4_), 7.86 (d, ^3^*J*_8–7_ = 8.2 Hz, 2 H, H-8 in C_5_H_4_), 7.88, 7.90 (d, ^3^*J*_8–7_ = 8.2 Hz, 2 H, H-5 in C_5_H_4_), ppm. ^13^C NMR (100.62 MHz, DMSO_d_6_): δ = 75.5 (POC), 124.7 (C-5 in Napht.), 125.0 (C-2 in Napht.), 125.3 (C 3, 7 in Napht.), 125.5 (C-6 in Napht.), 126.6 (C*_p_* in Ph), 127.1 (C*_o_*, in Ph), 127.4 (C-4 in Napht.), 127.7 (C*_m_* in Ph), 128.4 (C-8 in Napht.), 130.1 (C-9 in Napht.), 133.5 (C-10 in Napht.), 139.0, 143.6 (C*_i_* in Ph) ppm. ^31^P NMR (161.98 MHz, DMSO_d_6_): δ = −0.22 (t, ^3^*J*_POCH_ = 9.7 Hz) ppm. IR (KBr): 509, 607, 696, 779, 798, 884, 921, 989, 1048, 1082, 1101, 1185, 1248, 1268, 1453, 1494, 1510, 1597, 1634, 2924, 3030, 3058, 3086. Anal. Calcd for C_34_H_26_KO_4_P (m.w. 568.64): C, 71.81; H, 4.61; Found: C, 71.79; H, 4.60.

*Potassium bis[phenyl(pyridin-4-yl)methyl]phosphate* (**2m**). Following the general procedure, **2m** was prepared from **1m** (24 mmol, 4.40 g). **2m** was isolated as white powder (2.13 g, 38% yield), mp 125–127 °C. ^1^H NMR (400.13 MHz, CDCl_3_): δ = 6.23 (d, ^3^*J*_POCH_ = 9.6 Hz, 2 H, POCH), 7.14–7.27 (m, 10 H, *H_o,m,p_* in Ph), 7.36, 7.40 (d, ^3^*J*_PH_ = 5.4 Hz, 4 H, H-3,5 in C_5_H_4_), 8.28 (br.s, 4 H, H-2,6 in C_5_H_4_) ppm.^13^C NMR (100.62 MHz, CDCl_3_): δ = 76.8 (POCH), 122.3 (C-5 in C_5_H_4_), 126.6 (C*_o_* in Ph), 127.6 (C*_p_*, in Ph), 128.4 (C*_m_* in Ph), 134.9 (C-4 in C_5_H_4_), 138.6 (C-3 in C_5_H_4_), 141.4 (C*_i_* in Ph), 147.4 (C-2 in C_5_H_4_), 167.6 (C-6 in C_5_H_4_) ppm. ^31^P NMR (161.98 MHz, CDCl_3_): δ = −1.10 (br.t) ppm. IR (KBr): 3405, 3063, 2923, 2852, 1638, 1495, 1554, 1232, 1048, 889, 796, 700, 563 сm^−1^. Anal. Calcd for C_24_H_20_KN_2_O_4_P (m.w. 470.50): C, 61.27; H, 4.28; N, 5.95. Found: C, 61.20; H, 4.27; N, 5.15.

*Potassium bis[phenyl(pyridin-3-yl)methyl]phosphate* (**2n**). Following the general procedure, **2n** was prepared from **1n** (24 mmol, 4.40 g). **2n** was isolated as white powder (1.98 g, 35% yield), mp 195–197 °C. ^1^H NMR (400.13 MHz, CDCl_3_): δ = 6.15 (d, ^3^*J*_POCH_ = 9.2 Hz, 2 H, POCH), 6.94–6.96 (m, 2 H, H-5 in C_5_H_4_), 7.09–7.16 (m, 10 H, *H_o,m,p_* in Ph), 7.38–7.40 (m, 2 H, H-5 in C_5_H_4_), 8.24 (d, ^3^*J*_6–5_ = 5.0 Hz, 2 H, H-6 in C_5_H_4_), 8.57 (br.s, 2 H, H-2 in C_5_H_4_) ppm. ^13^C NMR (100.62 MHz, CDCl_3_): δ = 76.8 (POCH), 123.3 (C-5 in C_5_H_4_), 126.6 (C*_o_* in Ph), 127.6 (C*_p_*, in Ph), 128.4 (C*_m_* in Ph), 134.9 (C-4 in C_5_H_4_), 138.6 (C-3 in C_5_H_4_), 141.4 (C*_i_* in Ph), 147.4 (C-2 in C_5_H_4_), 147.6 (C-6 in C_5_H_4_) ppm. ^31^P NMR (161.98 MHz, CDCl_3_): δ = −1.06 (t, ^3^*J*_POCH_ = 9.1 Hz) ppm. ^15^N NMR (40.5 MHz, CDCl_3_): δ = −77.6 ppm. IR (KBr): 3429, 3030, 2922, 2853, 2388, 2300, 1632, 1579, 1495, 1426, 1192, 1049, 881, 700, 565 сm^−1^. Anal. Calcd for C_24_H_20_KN_2_O_4_P (m.w. 470.50): C, 61.27; H, 4.28; N, 5.95. Found: C, 61.19; H, 4.26; N, 5.35.

*Potassium bis[phenyl(2-thienyl)methyl]phosphate* (**2o**). Following the general procedure, **2o** was prepared from **1o** (24 mmol, 4.52 g). **2o** was isolated as white powder (0.46 g, 8% yield), mp 180 °C. ^1^H NMR (400.13 MHz, DMSO_d_6_): δ = 6.30 (d, ^3^*J*_POCH_ = 9.8 Hz, 2 H, POCH), 6.77–6.78 (m, 2 H, H-3 in C_4_H_3_S), 6.82–6.84 (m, 2 H, H-4 in C_4_H_3_S), 7.21–7.24 (m, 10 H in Ph), 7.30 (dd, ^3^*J*_5–4_ = 5.0 Hz, ^4^*J*_5–3_ = 1.8 Hz, 2 H, H-5 in C_4_H_3_S) ppm. ^13^C NMR (100.62 MHz, DMSO_d_6_): δ = 73.5, 73.6 (d, ^2^*J*_PC_ = 5.0 Hz, ^2^*J*_PC_ = 4.9 Hz, POCH), 124.4, 124.5 (C-3 in C_4_H_3_S), 124.9, 125.0 (C-5 in C_4_H_3_S), 126.1, 126.2 (C-4), 126.2, 126.3 (C*_o_* in Ph), 126.8, 126.9 (C*_p_* in Ph), 127.8 (C*_m_* in Ph), 143.8 (d, ^3^*J*_PC_ = 3.5 Hz, C*_i_* in Ph), 148.5, 148.6 (d, ^3^*J*_PC_ = 4.5 Hz, C-2 in C_4_H_3_S) ppm. ^31^P NMR (161.98 MHz, DMSO_d_6_): δ = −1.03 (t, ^3^*J*_POCH_ = 9.9 Hz) ppm. IR (KBr): 3430, 3062, 2931, 2301, 1613, 1512, 1454, 1399, 1247, 1175, 1099, 1001, 888, 833, 697, 574, 468 сm^−1^. Anal. Calcd for C_22_H_18_KO_4_PS_2_ (m.w. 480.58): C, 54.98; H, 3.78. Found: C, 44.51; H, 3.75.

*Furan-2-yl(phenyl)methylhypophosphite* (**2p**). Yield: (traces); oil. ^1^H NMR (400.13 MHz, DMSO_d_6_): δ = 6.06 (m), 6.07 (d, ^3^*J*_PH_ = 11.9 Hz), 6.29 (dt, ^4^*J*_3,5_ = 1.5, ^3^*J*_3,4_ = 3.1 Hz), 7.05 (d, ^1^*J*_PH_ = 454.2 Hz), 7.19–7.25 (m, 5H), 7.45 (ddd, J = 0.8, 1.63, 4.3 Hz) ppm. ^31^P NMR (161.98 MHz, DMSO___d_6_): δ = −2.82 (t, ^1^*J*_PH_ = 460 Hz) ppm. There was too little substance to record ^13^C NMR. Anal. Calcd for C_11_H_11_O_3_P (m.w. 222.18): C, 59.47; H, 4.99. Found: C, 59.31; H, 5.09.

The structure of furan-2-yl(phenyl)methylhypophosphite **2p** follows from ^1^H and ^31^P NMR spectra of the reaction mixture and their comparison with the known spectra of similar compounds [92].

*Diphenylmethanol* (**3a**) [93]. Following the general procedure, **3a** was prepared from **1a** (24 mmol, 4.71 g). **3a** was isolated as light beige crystals (1.64 g 37% yield), mp 60–62 °C (hexane). ^1^H NMR (400.13 MHz, DMSO_d_6_): δ = 5.71 (s, 1 H, CH), 5.90 (s, 1 H, OH), 7.18–7.40 (m, 10 H, H*_o,m,p_* in Ph) ppm. ^13^C NMR (100.62 MHz, DMSO_d_6_): δ = 74.4 (CH), 126.3 (C*_o_* in Ph), 126.8 (C*_p_* in Ph), 128.1 (C*_m_* in Ph), 145.8 (C*_i_* in Ph) ppm. IR (KBr): 1960, 1896, 1628, 1597, 1492, 1454, 1316, 1274, 1184, 1084, 1018, 917,830, 740, 701, 601, 548 сm^−1^. Anal. Calcd for C_13_H_12_O (m.w. 184.23): C, 84.75; H, 6.57. Found: C, 83.14; H, 6.55.

*Synthesis of the Dibenzhydryl Methyl Phosphate* (**5a**). The mixture of **2a** (100 mg) and MeI (300 mg, 10 eq.) in MeCN (2 mL) was stirred for 24 h at 50 °C. The solid part was then separated by centrifugation and the solvent was evaporated under vacuum, and 78 mg of **5a** (82%) as orange oil was obtained. ^1^H NMR (400.13 MHz, CDCl_3_): 3.39 (d, 3H, OСН_3_, ^3^*J*_Р-О-С-Н_ = 11.6 Hz), 6.34 (d, 2Н, РОСН, ^3^*J*_Р-О-С-Н_ = 8.4 Hz), 7.15–7.30 (m, 20Н, *Н_o_*, Н*_m_*, Н*_p_*). ^13^C NMR (100.62 MHz, CDCl_3_): δ = 54.11 (ОСН_3_, ^2^*J*_Р-О-С_ = 5.9 Hz), 81.25 (НС-О-Р, *^2^J_Р_*_-О-С_ = 5.1 Hz), 126.85 and 127.10 (C*_o_*), 128.02 and 128.20 (С*_p_*), 128.47 and 128.54 (C*_m_*), 140.27 and 140.45 (С*_i_*, ^3^*J*_Р-O-С-C_ = 4.9 and 5.4 Hz). ^31^P NMR (161.98 MHz, CDCl_3_): δ = 0.10 (qt ^3^*J*_Р-О-СН3_ = 11.6 Hz, ^3^*J*_Р-О-С-Н_ = 8.4 Hz). IR (microlayer): 3090, 3064, 3031, 3008, 2953, 2922, 2852, 1495, 1454, 1267, 1188, 1032, 1007, 990, 741, 697, 604, 558 сm^−1^. Anal. Calcd for C_27_H_25_O_4_P (m.w. 444.47): C, 72.96; H 5.67. Found: C 72.49; H 5.42.

## 4. Conclusions

In conclusion, diaryl(hetaryl)ketones are directly phosphorylated (85 °C, 1.5 h) with red phosphorus in the KOH/DMSO(H_2_O) suspension to give potassium bis(diaryl(hetaryl)methylphosphates in yields up to 45%.

Unlike the previously developed phosphorylation of various electrophiles with elemental phosphorus in strongly basic media proceeding as nucleophilic addition or substitution reactions to form in all of the cases only C-P bonds, the phosphorylation studied here leads to the formation of exclusively C-О-P bond systems.

Despite the modest yield of the diester of phosphoric acid, this synthesis may be rated as practically attractive, because it is based on cheap and available starting materials, is operationally simple and environmentally neutral. A major competitive advantage of this approach is that it allows avoiding toxic and irritating phosphorus chlorides or pentoxide and polyphosphoric acids. Also, this methodology enables the selective synthesis of diesters of phosphoric acid free of admixtures of mono- and triphosphates. Another beneficial feature of this chemistry is the possibility of preparing asymmetrical bis(diarylmethyl)phosphates, including those combining aromatic and heteroaromatic substituents.

## Figures and Tables

**Table 1:** Influence of the phosphorylation conditions on the conversion of **1a**, the ratio, and yields of products **2a**, **3a**, and **4a** ^a^.

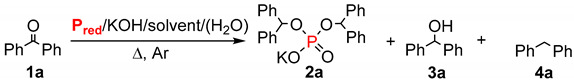									
Entry	1a:P_red_:H_2_ OMolar Ratio	Base	Solvent ^b^	T, °C	*t*, h	Conversion of 1a, %	Yield of, (%)		
							2a ^c^	3a ^d^	4a ^d^
1	1:1:3.5	KOH	DMSO	85	3.0	74	24	9	7
2	1:1:1.7	KOH	DMSO	85	3.0	86	31	23	^e^
3	1:1:0.9	KOH	DMSO	85	3.0	86	34	19	11
4	1:1:1.7	KOH	DMSO	60	3.0	87	29	27	12
5	1:1.5:1.7	KOH	DMSO	75	3.0	98	33	48	14
6	1:1:1.7	KOH	DMSO	96	6.0	~100	32	36	2
7	1:2:3.5	KOH	DMSO	85	3.0	81	36	44	5
8	1:4:3.5	KOH	DMSO	85	1.5	100	31	49 ^c^	^e^
9	1:2:1.7	KOH	DMSO	85	1.5	100	38	37 ^c^	^e^
10	1:3:0.9	KOH	DMSO	85	1.5	100	45	18	4
11 ^f^	1:3:0.9	KOH	DMSO	85	1.5	100	11	84 ^c^	e
12	1:3:0.5	KOH	DMSO	85	1.5	100	41	15	7
13	1:3:0	KOH	DMSO	85	1.5	100	39	9	7
14	1:3:0	KOH	DMSO ^g^	85	1.5	100	31	26	12
15	1:3:0.9	KOH	HMPA	85	1.5	100	30 ^dh^	29 ^c^	^e^
16	1:3:0.9	KOH	NMP	85	1.5	100	14	68 ^c^	^e^
17	1:3:0.9	KOH	Sulfolane	85	1.5	100	20 ^dh^	29 ^c^	^e^
18	1:3:0.9	KOH	DMF	85	1.5	42	4	5	2

^a^ Reagents and conditions: red phosphorus (50–100 mmol), benzophenone (24–48 mmol), base (154 mmol), water (0–167 mmol), and Ar. ^b^ Commercial solvent (50 mL) without further purification was used. ^с^ Isolated yield. ^d^ Yield determined by ^1^H NMR using durene as an internal standard. ^e^ Traces. ^f^ **1a** was added dropwise for 1.5 h. ^g^ Dry DMSO (over MS 3 Å) was used instead of commercial DMSO. ^h^ Contaminated with the solvent.

## Data Availability

The data presented in this study are available in the Supplementary Materials.

## Supplementary Materials

The following supporting information can be downloaded at https://www.mdpi.com/article/10.3390/molecules30061367/s1: The synthesis of initial ketones (S4–S6); Table S1: Screening of bases (S7); Control experiments: Radical-probe experiments (S9–S13); ^31^P NMR spectra of the reaction mixtures (Figures S1–S6); Reactions of (2- or 4-halo)benzophenones with red phosphorus (S13–S14); EPR experiments (S15–S18, Figures S7–S12); Evaluation of **2a** stability (S19); Crystal data and details of experiments for **2a** (S20, Figures S13–S16); Unsuccessful substrates (S25, Figure S17), Copy of ^1^H NMR, ^13^C NMR, ^31^P NMR, Raman, and FT-IR spectra of compounds **2a**–**p** and **3a** (S26–S89). Refs. [90,91,94,95,96,97,98,99,100,101,102,103,104,105,106,107,108,109,110,111,112,113,114,115,116,117,118,119,120,121,122,123] are cited in the Supplementary Materials.
