# Supplementary material for: Straightforward Superbase-Mediated Reductive O-Phosphorylation of Aromatic and Heteroaromatic Ketones with Red Phosphorus in the Superbase Suspension KOH/DMSO(H2O)
Source: Molecules. 2025 Mar 18;30(6):1367. doi: 10.3390/molecules30061367 (PMC11946803; doi:10.3390/molecules30061367)
Supplement: Supplementary file 1 [file molecules-30-01367-s001.zip › molecules-3510186-supplementary.pdf]

Supporting Information

Straightforward superbase-mediated phosphorylation of aromatic and heteroaromatic ketones with red phosphorus in the superbase suspension KOH/DMSO(H_2O)

Vladimir A. Kuimov,[a] Svetlana F. Malysheva,[a] Natalia A. Belogorlova,[a] Ruslan I. Fattakhov,[a] Alexander I. Albanov,[a] Irina Yu. Bagryanskaya,[b] Nikolay I. Tikhonov,[a] Boris A. Trofimov[a]

[a]A.E. Favorsky Irkutsk Institute of Chemistry, Siberian Branch, Russian Academy of Sciences, 664033, Irkutsk, 1 Favorsky st., Russia

[b]N. N. Vorozhtsov Novosibirsk Institute of Organic Chemistry, Siberian Branch, Russian Academy of Sciences, Novosibirsk 630090

Table of Contents

1.	General consideration	S3
2.	Experimental procedure	S4
2.1.	Synthesis of starting ketones	S4
2.2.	Optimization of the reaction conditions	S7
2.3.	Procedure for the preparation of the potassium phosphate **2a** in three parallel	S8
3.	Control experiments	S9
3.1.	Radical-probe experiments	S9
3.2.	Reactions of (2- or 4-halo)benzophenones with red phosphorus	S13
3.3.	EPR experiments	S15
3.4	Evaluation of **2a's** stability	S19
4.	X-Ray diffraction analysis of **2a**	S20
5.	Unsuccessful Substrates	S25
6.	Spectral data of the synthesized products (NMR spectra)	S26
7.	References	S93

1. General consideration

All manipulations were carried out under argon atmosphere. ^{1}H, ^{13}C, ^{31}P, and ^{19}F NMR spectra were recorded at 400.13, 100.62, 161.98 and 376.50 MHz, respectively, in DMSO or $CDCl_3$ solutions with a Bruker DPX-400 spectrometer. Chemical shifts were reported in δ (ppm) relative to DMSO_d_6 or $CDCl_3$ (^{1}H, ^{13}C) as internal standard or H_3PO_4 (^{31}P) as external standard. The assignment of signals in the ^{1}H NMR spectra was made using COSY experiments. Resonance signals of carbon atoms were assigned based on ^{1}H-^{13}C HSQC and ^{1}H-^{13}C HMBC experiments. IR spectra were run on a Bruker IFS 25 spectrometer. The EI mass spectra were obtained on a Shimadzu GSMS-QP5050 mass spectrometer. Melting points were recorded on a Stuart melting point apparatus and are uncorrected. Commercially available red phosphorus (KSAN Sia, Riga, Latvia) was purified by consecutive washing with aq. NaOH (1–2%), H_2O, EtOH, and Et_2O to remove all acidic impurities, dried in vacuum at 25–30 °C to constant weight, and stored under inert atmosphere (N_2). Commercially available undried DMSO (~1% of water) was used (Vekton). Commercially available potassium hydroxide (from Sigma-Aldrich) of composition KOH (~85%){Abdel-Magid, 2001 #8178} and H_2O (≤15%) that almost exactly corresponds to the formula $KOH{\cdot}0.5H_2O$[(1)] was used without further purification. The water content in $KOH{\cdot}0.5H_2O$ was determined using HB43-S Halogen METTLER TOLEDO. Most of the diaryl(hetaryl)ketones are commercial products: **1a-f,j,l-o,** 9H-fluoren-9-one, anthracen-9(10H)-one. Other ketones (3-Methoxybenzophenone (**1g**), (4-(methylthio)phenyl)(phenyl)methanone (**1h**), benzyloxybenzophenone (**1i**), 3-fluorobenzophenone (**1k**), 1-naphthyl(phenyl)methanone (**1l**), 2-benzoylfurane (**1p**) and 2-methoxynaphthalen-1-yl(phenyl)methanone) were synthesized according to the literature methods.

EPR spectra of the samples were recorded with the ELEXSYS E-580 spectrometer (Bruker Corporation, Billerica, MA), X-band 9.7 GHz.

Raman spectra were obtained on an ATP 8900Ad Raman vacuum Fourier transform infrared spectrometer (Optosky Photonics, China), на 785 and 1064 nm.

2. Experimental procedure

2.1. Synthesis of starting ketones

***2.1.1.** 3-Methoxybenzophenone (**1g**) synthesis*

3-Methoxybenzophenone (58%) was prepared as described in ref.[2] from 3-methoxybenzoyl chloride and benzene in the presence of $AlCl_3$. Physical-chemical characteristics of the ketone **1k** were identical to the literature data.[3]

***2.1.2.** 4-Methylthiobenzophenone (**1h**) synthesis*

4-Methylthiobenzophenone (48%) was prepared according to known method[4] by reaction of methylthioanisole and benzoyl chloride in the presence of $AlC1_3$. Physical-chemical characteristics of the ketone **1k** were identical to the literature data.[4]

***2.1.3.** 4-benzyloxybenzophenone (**1i**) synthesis*

A mixture of 4-hydroxybenzophenone (4.76 g, 0.024 mol) and benzyl chloride (3.29 g, 0.026 mol) were dissolved in 35 mL of DMSO and 1.20 g of NaOH (0.03 mol). The mixture was stirred at room temperature overnight. Water (50 mL) was added and the resulting solution was extracted with (3×30 mL) of Et_2O. The combined organic phases were washed twice with 30 mL of aqueous NaOH (10%) and water (3×20 mL), dried over Na_2SO_4, and the solvent was evaporated. The resulting yellowish product was purified from methanol to yield 6.86 g (99%) of pure product as confirmed by NMR.[5] ^{1}H NMR ($CDCl_3$/CCl_4, TMS), δ (ppm): 5.12 (s, 2H), 7.00 (d, 2H, J_{HH} 8.8 Hz), 7.29-7.45 (m, 7H), 7.52 (t, 1H, J_{HH} 7.4 Hz), 7.72 (d, 2H, J_{HH} 7.4 Hz), 8.74 (d, 2H, J_{HH} 8.7 Hz). ^{13}C NMR ($CDCl_3$/CCl_4, TMS), δ (ppm): 194.8, 162.2, 138.1, 136.1, 132.3, 131.6, 130.2, 129.5, 128.5, 128.0, 127.9, 127.3, 114.2, 69.9.

***2.1.4.** 3-Fluorobenzophenone (**1k**) synthesis*

Ketone **1k** (81%) was synthesized according to known method[2a] from 3-fluorobenzoyl chloride and benzene in the presence of $AlCl_3$. Physical-chemical characteristics of the ketone **1k** were identical to the literature data.[6]

***2.1.5.** (1-Naphthyl)(phenyl)methanone (**1l**) synthesis*

Ketone **1l** was synthesized according to known method[7] in 28% yield.

^{1}H ($CDCl_3$): δ 8.06 (d, 1H, J_{HH} 7.9 Hz), 7.97 (d, 1H, J_{HH} 7.1 Hz), 7.88 (d, 1H, J_{HH} 7.5 Hz), 7.84 (d, 2H, J_{HH} 7.5 Hz), 7.55 (q, 2H, J_{HH} 8.0 Hz), 7.48 (t, 3H, J_{HH} 7.2 Hz), 7.43 (t, 2H, J_{HH} 7.7 Hz); ^{13}C ($CDCl_3$): δ 197.4, 138.3, 136.4, 133.7, 133.0, 131.1, 130.9, 130.3, 128.3, 127.6, 127.1, 126.4, 125.8, 124.2 (lit.[8]).

***2.1.6.** 3-Benzoylpyridine synthesis (**1n**)*

3-Benzoylpyridine was prepared as described in ref.[9] Physical-chemical characteristics of the ketone **1k** were identical to the literature data.[5]

***2.1.7.** 4-Benzoylpyridine synthesis (**1m**)*

4-Benzoylpyridine was prepared by the published procedure[9] with little modification. In a 250 mL three-necked flask, fitted with a glycerin-sealed mechanical stirrer, a reflux condenser protected with a calcium chloride tube, and a dropping funnel, a dry isonicotinic acid (12.3 g, 0.1 mole) dissolved in freshly distilled thionyl chloride (82 g, 0.69 mol) and reflux during 1 h. Then

the reflux condenser is replaced by downward condenser for distilling off excess $SOCl_2$ at reduced pressure. Then anhydrous benzene (50 mL) was added and the benzene/$SOCl_2$ azeotrope is distilled at reduced pressure. A new portion of benzene (50 mL) was added again to a brown suspension and the reaction mixture was cold down (0-2 °C), and anhydrous $AlCl_3$ is added in portions (6×5.5 g) over a period of 1 h. The reaction flask was reflux for 6 h. The dark red-brown reaction mixture is poured onto a mixture of 200 g of ice and 20 ml of concentrated hydrochloric acid. The dense brown organic layer is separated and discarded. The acid solution is extracted with Et_2O (3×50 mL), which are discarded; then it is treated with 50% aqueous sodium hydroxide (200 mL) until the aluminum hydroxide which first forms redissolves. After cooling, the ketone is extracted with $CHCl_3$ (20×50 mL). The combined extracts are washed with water (10×20 mL), the solvent is removed and purification was done by recrystallization from hexane to get 9.8 g (52%) of 4-benzoylpyridine as yellow wax. ^{1}H ($CDCl_3$/CCl_4, TMS): δ 7.50 (t, 2H, J_{HH} 7.6 Hz), 7.56 (d, 2H, J_{HH} 4.8 Hz), 7.62 (t, 1H, J_{HH} 7.4 Hz), 7.80 (d, 2H, J_{HH} 7.6 Hz), 8.79 (d, 2H, J_{HH} 4.9 Hz). ^{13}C ($CDCl_3$/CCl_4): δ 122.8, 128.6, 130.1, 133.4, 135.9, 144.3, 150.3, 194.8. lit.[6,10]

2.1.8. *2-Benzoylthiophene (**1o**) synthesis*

2-Benzoylthiophene (87% yield) was synthesized by published procedure[11] from thiophene and benzoyl chloride in the presence of $AlCl_3$. Physical-chemical characteristics of the ketone **1o** were identical to the literature data.[5]

2.1.9. *2-Benzoylfurane (**1p**) synthesis*

To a suspension of $AlCl_3$ (4.44g, 0.033 mol) in excess of dry benzene (16.00 g), 4.35 g of furan-2-carbonyl chloride (prepared from furan-2-carboxylic acid and $SOCl_2$) carefully adding dropwise for 0.5 h at room temperature. The reaction mixture was heated at 80 °C for 3.5 h and the black oily product obtained was purified on a Al_2O_3 (petroleum ether : ethyl acetate at 97:3 ratio) to give 4.20 g of brown oil (73%). ^{1}H NMR ($CDCl_3$/CCl_4, TMS), δ (ppm): 6.57 (dd, 1H, J_{HH} 1.5, 3.4 Hz), 7.22 (d, 1H, J_{HH} 3.5 Hz), 7.47 (t, 1H, J_{HH} 7.6 Hz), 7.56 (t, 1H, J_{HH} 7.1 Hz), 7.68 (m, 1H), 7.96 (d, 1H, J_{HH} 7.5 Hz). ^{13}C NMR ($CDCl_3$/CCl_4), δ (ppm): 111.6, 119.7, 127.7, 128.6, 131.9, 136.6, 146.4, 151.7, 181.2 (lit.[6]).

2.1.10. *4-vinyloxybenzophenone and (4-((methylthio)methoxy)phenyl)(phenyl)methanone synthesis*

A suspension of 4-hydroxybenzophenone (9.91 g, 0.05 mol) and freshly machine-powdered $KOH{\cdot}0.5H_2O$ (1.63 g, 0.025 mol) in DMSO (50 mL) was placed into a 0.25-L stirred reactor (Parr reactor, 240 rpm). The latter was fed with acetylene under pressure (initial pressure at ambient temperature was 12-14 atm and then decompressed to atmospheric pressure to remove air). The reactor was fed with acetylene again (3 times, until the acetylene adsorption stops) and heated (120 °C) for 3 h. The reaction mixture, after cooling to room temperature, was diluted with aq. NH_4Cl (1%, 100 mL) and extracted with diethyl ether (6×30 mL). The extract was washed with aq. NaOH (10%, 6×20 mL) to remove unreacted ketone (conversion 31%), and dried over Na_2SO_4 overnight. After removal of the solvent, a crude brown oil (3.986 g) was fractionalized in vacuum (3 bar) to give 4-vinyloxybenzophenone (1.12 g, 10%) at 158-160 °C and (4-((methylthio)methoxy)phenyl)(phenyl)methanone (1.41 g, 11%) at 195-198 °C.

4-vinyloxybenzophenone

^1H NMR ($CDCl_3/CCl_4$, TMS), δ (ppm): 4.56 (dd, 1H, J_{HH} 1.5, 6.0 Hz), 4.89 (dd, 1H, J_{HH} 1.5, 13.6 Hz), 6.67 (dd, 1H, J_{HH} 6.0, 13.6 Hz), 7.04 (d, 2H, J_{HH} 8.7 Hz), 7.44 (t, 2H, J_{HH} 6.1 Hz), 7.54 (m, 1H), 7.73 (d, J_{HH} 7.2 Hz), 7.81 (d, 2H, J_{HH} 8.7 Hz). ^{13}C NMR ($CDCl_3/CCl_4$), δ (ppm): 97.3, 115.8, 128.1, 129.6, 131.9, 132.0, 132.3, 137.7, 146.3, 159.9, 194.9.

(4-((Methylthio)methoxy)phenyl)(phenyl)methanone

^1H NMR ($CDCl_3/CCl_4$, TMS), δ (ppm): 2.25 (s, 3H), 5.18 (s, 2H), 6.97 (d, 2H, J_{HH} 8.6 Hz), 7.43 (t, 2H, J_{HH} 7.5 Hz), 7.52 (t, 1H, J_{HH} 7.2 Hz), 7.73 (d, 2H, J_{HH} 7.6 Hz), 7.79 (d, 2H, J_{HH} 8.6 Hz). ^{13}C NMR ($CDCl_3/CCl_4$), δ (ppm): 14.2, 71.8, 114.7, 127.8, 129.3, 130.5, 131.5, 131.9, 137.8, 160.2, 194.3.

2.1.11. *4-allyloxybenzophenone synthesis*

A mixture of 4-hydroxybenzophenone (4.76 g, 0.024 mol) and allyl chloride (2.00 g, 0.026 mol) were dissolved in 35 mL of DMSO and 1.20 g of powdered sodium hydroxide (0.03 mol) was added. The mixture was stirred at room temperature overnight. Water (50 mL) was added and the resulting solution was extracted with (3×30 mL) of diethyl ether. The combined organic phases were washed with 30 mL of aqueous NaOH (10%) and water (3×20 mL), dried over Na_2SO_4, and the solvent was evaporated. The resulting yellowish product was purified from methanol to yield 2.01 g (35%) of pure product as confirmed by NMR (lit.[(12)])

^1H NMR ($CDCl_3/CCl_4$, TMS), δ (ppm): 4.61 (d, 2H, J_{HH} 4.2 Hz), 5.31 (d, 1H, J_{HH} 10.4 Hz) and 5.42 (d, 1H, J_{HH} 17.2 Hz), 6.04 (qd, 1H, J_{HH} 4.9, 10.2 Hz), 6.94 (d, 2H, J_{HH} 8.6 Hz); 7.45 (t, 2H, J_{HH} 7.3 Hz), 7.53 (t, 1H, J_{HH} 7.0 Hz), 7.73 (d, 2H, J_{HH} 7.3 Hz), 7.79 (2H, J_{HH} 8.7 Hz). ^{13}C NMR ($CDCl_3$), δ (ppm): 68.8, 114.1, 118.1, 128.1, 129.6, 130.1, 1231.8, 132.4, 138.1, 162.1, 195.4.

2.1.12. *4-Hexyloxybenzophenone synthesis*

A mixture of 4-hydroxybenzophenone (4.76 g, 0.024 mol) and hexyl iodide (5.57 g, 0.026 mol) were dissolved in 35 mL of DMSO and 1.20 g of sodium hydroxide (0.03 mol). The mixture was stirred at room temperature overnight. Water (50 mL) was added and the resulting solution was extracted with (3×30 mL) of diethyl ether. The combined organic phases were washed twice with 30 mL of aqueous NaOH (10%) and water (3×20 mL), dried over Na_2SO_4, and the solvent was evaporated, to yield 6.14 g (91%) of pure product as confirmed by NMR.

^1H NMR ($CDCl_3/CCl_4$, TMS), δ (ppm): 0.86 (t, 3H), 1.29-1.32 (m, 4H), 1.42 (m, 2H), 1.75 (m, 2H), 3.96 (d, 2H, J_{HH} 6.5 Hz), 6.86 (d, 2H, J_{HH} 8.7 Hz), 7.39 (t, 2H, J_{HH} 7.5 Hz), 7.47 (t, 1H, J_{HH} 7.3 Hz), 7.67 (d, 2H, J_{HH} 7.7 Hz), 8.67 (d, 2H, J_{HH} 8.7 Hz). ^{13}C NMR ($CDCl_3/CCl_4$), δ (ppm): 14.0, 23.5, 25.6, 29.1, 31.5, 68.1, 113.9, 128.0, 129.6, 129.9, 131.6, 132.4, 138.4, 162.7, 194.8.

2.1.13. *2,4-difluorobenzophenone synthesis*

A mixture of 1,3-difluorobenzene (6.84 g, 0.06 mol), benzoyl chloride (7.03 g, 0.05 mol) and $AlCl_3$ (6.68 g, 0.05 mol) was refluxed 10 h. Then the cold mixture was treated with water (50 mL) and HCl (8%, 2 mL), mixed 40 min and extracted with $CHCl_3$ (3×30 mL). The organic layer was dried over K_2CO_3, filtrated and solvent removed under vacuum to get 2,4-difluorobenzophenone (8.52 g, 78%). ^1H NMR ($CDCl_3$, TMS), δ (ppm): 6.88 (ddd, 1H, J_{HF} 9.9 Hz, J_{HH} 8.9 Hz, J_{HF} 2.4 Hz), 6.98 (tdd, 1H, J_{HF} 8.7 Hz, J_{HH} 2.4 Hz, J_{HF} 0.8 Hz), 7.45 (t, 2H, J_{HH} 7.7 Hz), 7.53-7.61 (m, 2H), 7.79 (d, 2H, J_{HH} 8.8 Hz); ^{13}C NMR ($CDCl_3$), δ (ppm): 104.63 (t, J_{CF} 25.5 Hz), 111.82 (dd, J_{CF} 21.6, 3.6 Hz), 123.33 (dd, J_{CF} 14.7, 3.8 Hz), 128.47, 129.65, 132.50

(dd, J_{CF} 10.3, 4.4 Hz), 133.42, 137.36, 160.90 (dd, J_{CF} 256.0, 12.3 Hz), 164.86 (dd, J_{CF} 254.7, 11.6 Hz), 192.25. *m/z* 218 (M^+).

2.2. Optimization of reaction conditions

Table S1. Screening of bases.[a]

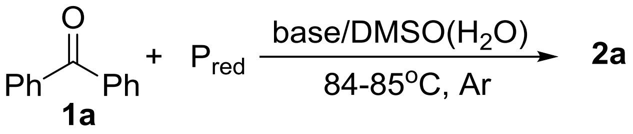

Entry	Bases	Time, h	Yield of **2a** (%)[b]	Comments
1	$LiOH·H_2O$	1.5	0	Predominantly **1a** and P_{red} were recovered. In NMR ^{31}P of the reaction mixture the signals of traces of lithium (hydroxydiphenylmethyl)-*H*-phosphinate were observed at 3.76 ppm, d, $^1J_{PH}$ 564 Hz.
2	$KOH·0.5H_2O$	1.5	47	The total conversion of P_{red} and **1a** was observed.
3[c]	*t*-BuOK	1.5	Traces	Predominantly unknown OPCs mainly were formed. The conversion of P_{red} was ~ 85%.
4	$Ca(OH)_2$[d]	4	0	P_{red} cleavage was not observed. **1a** was mostly recovered.
5	$Ba(OH)_2$[d]	4	0	P_{red} cleavage was not observed. **1a** was mostly recovered.
6	$[Et_3NBn]OH$[e]	4	0	Traces of primary phosphine oxide were observed in the reaction mixture, P_{red} and **1a** was mainly recovered.
7	$[Bu_4N]OH$[f]	4	2	The mixture of primary phosphine oxide, (hydroxydiphenylmethyl)-*H*-phosphinate and -H-phosphonate was isolated with total yield of <5%. The main product was **3a**. The conversion of P_{red} was ~ 50%.
8[g]	DABCO	4	0	In NMR ^{31}P of the reaction mixture only the signal of 1,4-diazabicyclo[2.2.2]octan-1-ium (hydroxydiphenylmethyl)-*H*-phosphinate was observed at -0.43 ppm, d, $^1J_{PH}$ 574 Hz. The conversion of P_{red} was ~ 12%.

[a] *Reaction conditions*: P_{red} (7.3 mmol), **1a** (2.4 mmol), base (15.4 mmol), water (2.2 mmol), DMSO (5 mL), Ar, 84-85 °C;
[b] Yields were determined by ^1H NMR using durene as internal; [c] Dry DMSO (over MS 3Å) was used instead of commercial DMSO; [d] Freshly prepared from MO and water (1:5). [e] $[Et_3NBn]OH$ was freshly prepared in advance from TEBAC and KOH in DMSO at 85 °C for 10 min before the addition of P_{red} and **1a**; [f] To 11 mL of commercial 40% aq. sln. of Bu_4NOH (4.41 g, 17 mmol, Alfa Aesar) in dry DMSO (20 mL), was added 23.4 g of molecular sieves (4 Å). The mixture was allowed to stand for 2 d. Then the sieves were removed quickly by filtration under vacuum. The content of water in the reaction mixture was ≈10%;
[g] *Reaction conditions*: P_{red} (0.73 mmol), **1a** (0.24 mmol), DABCO (1.54 mmol), DMSO (0.5 mL), Ar, 84-85 °C.

2.3. Procedure for the preparation of the potassium phosphate 2a in three parallel

1). A 250 mL round-bottom flask was sequentially charged with red phosphorus (2.25 g, 73 mmol), ketone **1a** (24 mmol), DMSO (50 mL), freshly machine-powdered $KOH·0.5H_2O$ (10.00 g, 154 mmol), H_2O (0.38 g, 21 mmol) and flushed with argon from the balloon. The flask was sunk into preliminary heated oil bath and the reaction mixture was stirred (500-700 rpm) for 1.5 hours at 84-85 ^oC. After the reaction completion, the flask was cold down to r.t., one part of water (25 mL) was added, and the reaction mixture was transferred into a separation funnel. Separated aqueous basic layer (~18 g) was discarded (consist of non-organic phosphates and KOH), and new portion of water (25 mL) added again to the reaction mixture. After extraction with $CHCl_3$ (3×25 mL), the organic layer was separated and $CHCl_3$ and DMSO were distilled under reduced pressure. The resulting product was washed with diethyl ether (3×15 mL) and $CHCl_3$ (2×2 mL) to remove diphenylcarbinol and diphenylmethane, dried in vacuum to give 2.53 g (45%) of the phosphate **2a**.

2). A mixture of red phosphorus (2.25 g, 73 mmol), benzophenone (4.37 g, 24 mmol), DMSO (50 mL), freshly machine-powdered $KOH·0.5H_2O$ (10.00 g) and H_2O (0.38 g) was gradually heated to 80-85 ^oC for 90-100 min under argon atmosphere. The color of the reaction mixture turned burgundy-red tones, gradually faded and became pastel brown. Then the reaction mixture was cooled to room temperature and diluted with 25 mL of water. After separation of the heavy aqueous-inorganic layer, water (25 mL) was added to the reaction mixture, and the latter was cooled to room temperature and extracted with chloroform (3×25 mL). If necessary, more water or 10% KCl solution (10-20 mL) can be added to better separate the aqueous alkaline and organic layers. After separation of the organic layer, chloroform, water, and DMSO were removed under reduced pressure. The paraffin residue (4.16 g) was thoroughly washed with ether (5×15 mL) to remove carbinol and diphenylmethane, and dried in vacuo to give white powder **2a** (2.42 g, 43%).

3). From red phosphorus (2.25 g, 73 mmol), benzophenone (4.37 g, 24 mmol), DMSO (50 mL), freshly powdered $KOH·0.5H_2O$ (10.00 g), H_2O (0.38 g) at 82-84 ^oC, 90 min, Ar, after similar treatment of the reaction mixture, **2a** (2.47 g, 44% yield) was obtained.

3. Control experiments

Other features, which shed light on the mechanism of this extraordinary cascade phosphorylation, are (i) bright purple color of the reaction mixture, (ii) decreasing the yield of phosphate **2a** as well as **3a** and **4a** in the presence of typical radical scavengers (hydroquinone, TEMPO, quinhydrone), (iii) characteristic ESR signals appeared during the reaction.

3.1. Radical-probe experiments:

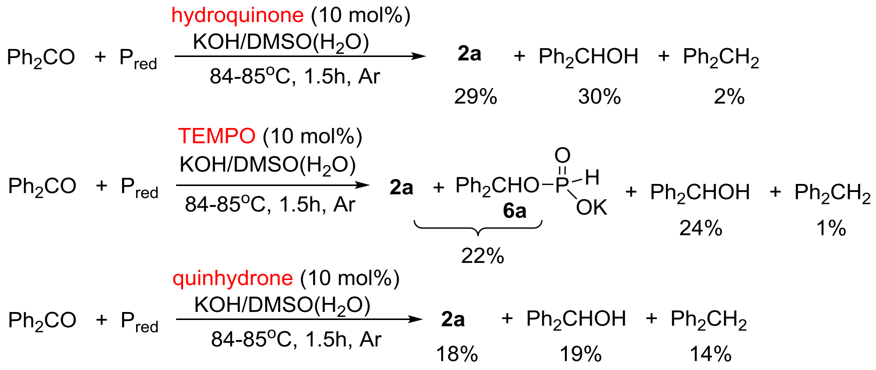

3.1.1. Hydroquinone experiment

A 250 mL round-bottom flask was sequentially charged with red phosphorus (73 mmol), benzophenone (24 mmol), freshly machine-powdered KOH·0.5H_2O (154 mmol), hydroquinone (2.4 mmol), H_2O (21 mmol) in DMSO (50 mL) and flushed with argon from the balloon. Reaction mixture was stirred (500-700 rpm) for 1.5 hours at 84-85 ^{o}C (oil bath). After the reaction completion, the ~0.6 mL of the reaction solution was directly used for ^{31}P NMR testing without any purification. In the ^{31}P spectrum of the reaction mixture we observed 5 signals, which could be interpreted as following organophosphorus molecules (Scheme S1): $Ph_2C(OH)P(O)H_2$ (t, -2.55 ppm, $^1J_{PH}$ = 467.4 Hz), **2a** (t, -0.5 ppm, $^3J_{PH}$ = 10.0 Hz), $Ph_2CHOP(O)H(OK)$ (dd, -0.23 ppm, $^1J_{PH}$ = 581.4 Hz, $^3J_{PH}$ = 10.5 Hz), $Ph_2C(OH)P(O)H(OK)$ (d, 0.04 ppm, $^1J_{PH}$ = 583.2 Hz), $(Ph_2CHO)_3P(OK)$-PH_2 (td, -173.38 ppm, $^1J_{PP}$ = 76.9 Hz, $^1J_{PH}$ = 189.0 Hz; dq, 16.14 ppm, $^1J_{PP}$ = 76.9 Hz (see lit.[13e]), $^3J_{PH}$ = 12.4 Hz) in a about 8 : 15 : 0.7 : 1.5 : 1 molar ratio. The phosphate **2a** was isolated by the method described in section 2 in 29% yield. This indicates that phosphate **2a** is formed via the phospha-Brook rearrangement. The assignment of signals can be made based on the chemical shifts of the ^{31}P NMR spectra, the nature of the splitting and the spin-spin coupling constants, authentic or similar to those of similar known OPCs.[13]

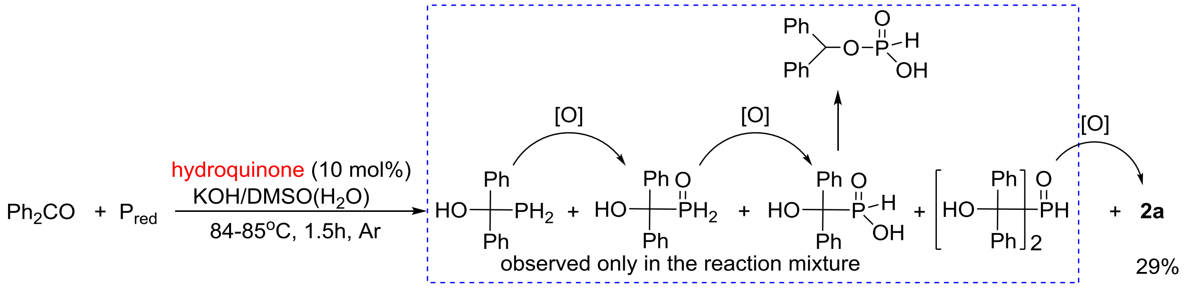

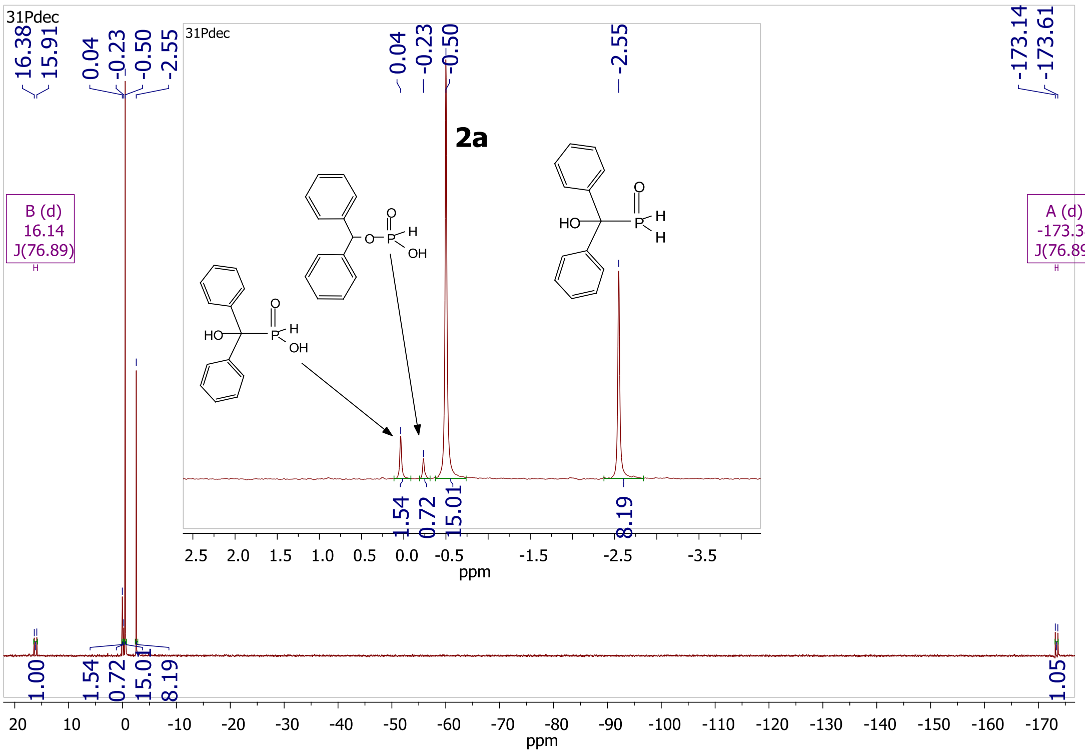

Figure S1. ^{31}P{H} NMR spectrum of the reaction mixture **1a**/P_{red}/KOH/DMSO(H_2O) in the presence of hydroquinone (10 mol%) at 85 °C in 1.5 h.

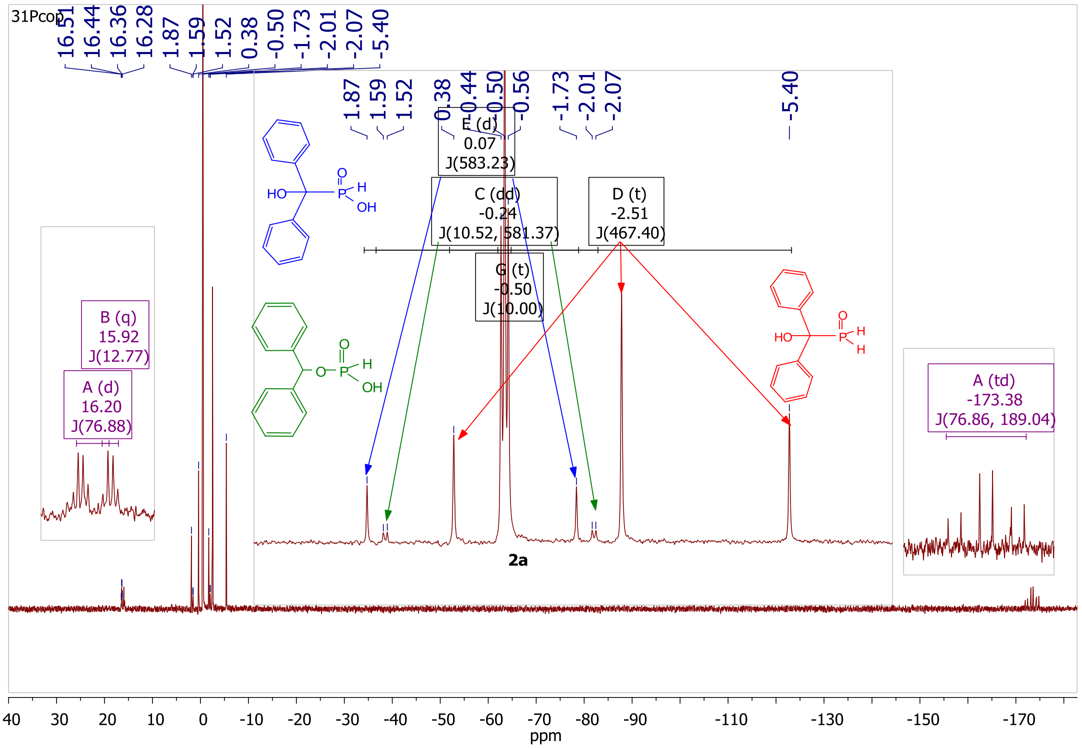

Figure S2. ^{31}P NMR spectrum of the reaction mixture **1a**/P_{red}/KOH/DMSO(H_2O) in the presence of hydroquinone (10 mol%) at 85 °C in 1.5 h.

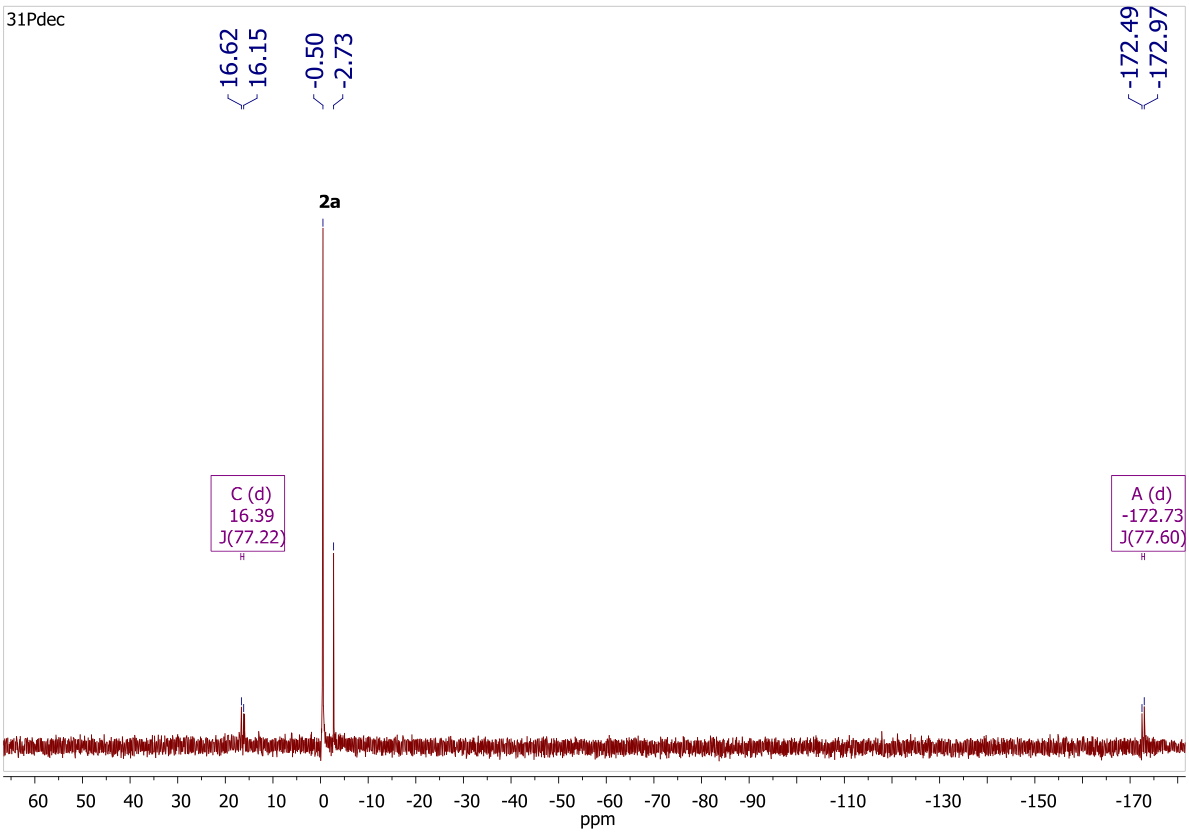

Figure S3. $^{31}P\{^1H\}$ NMR spectrum of the reaction mixture **1a**/P_{red}/KOH/DMSO(H_2O) in the presence of TEMPO (10 mol%) at 85 °C in 1.5 h.

Mix of potassium bis(diphenylmethyl)phosphate (**2a**) and potassium diphenylmethylphosphate (**6a**) as ~1:2 was isolated in the experiment with TEMPO.
^{1}H NMR (400.13 MHz, DMSO_d$_6$): δ = 6.05 (d, $^3J_{PH}$ = 10.1 Hz, 2 H, POCH) for **2a** and 6.13 (d, $^3J_{PH}$ = 10.5 Hz, 2 H, POCH) for **6a**, 6.63 (d, $^3J_{PH}$ = 586.6 Hz, 1 H, PH), 7.10-7.21 (m, $H_{o,m,p}$ in Ph) ppm. ^{13}C NMR (100.62 MHz, DMSO_d$_6$): δ = 77.2 (d, $^2J_{PC}$ = 5.2 Hz, POCH) for **2a** and 75.9 (d, $^2J_{PC}$ = 4.2 Hz, POCH) for **6a**, 126.4 (C_p), 126.6 (C_o), 127.7 (C_m) for **2a** and 126.58 (C_o), 126.7 (C_p), 128.0 (C_m) for **6a**, 144.4 (d, $^3J_{PC}$ = 4.3 Hz, C_i) for **2a** and 144.3 (d, $^3J_{PC}$ = 3.7 Hz, C_i) for **6a** ppm. ^{31}P NMR (161.98 MHz, DMSO_d$_6$): δ = –0.07 (**6a**, dd, $^1J_{PH}$ = 586.9 Hz, $^3J_{PH}$ = 10.4 Hz) and -0.55 (**2a,** t $^3J_{PH}$ = 9.9 Hz) ppm. IR (KBr): 3435, 3028, 3058, 2932, 1657, 1599, 1493, 1453, 1261, 1188, 1103, 1008, 884, 859, 707, 573, 487 cm^{-1}.

Since the radical scavengers were taken in amounts much less than equimolar compared to the ketones, they trap only a small part of forming radicals, while a main part of the starting compounds further participate in the major process. Besides, the free-radical species may not come out to the solution from anion-radical pairs.

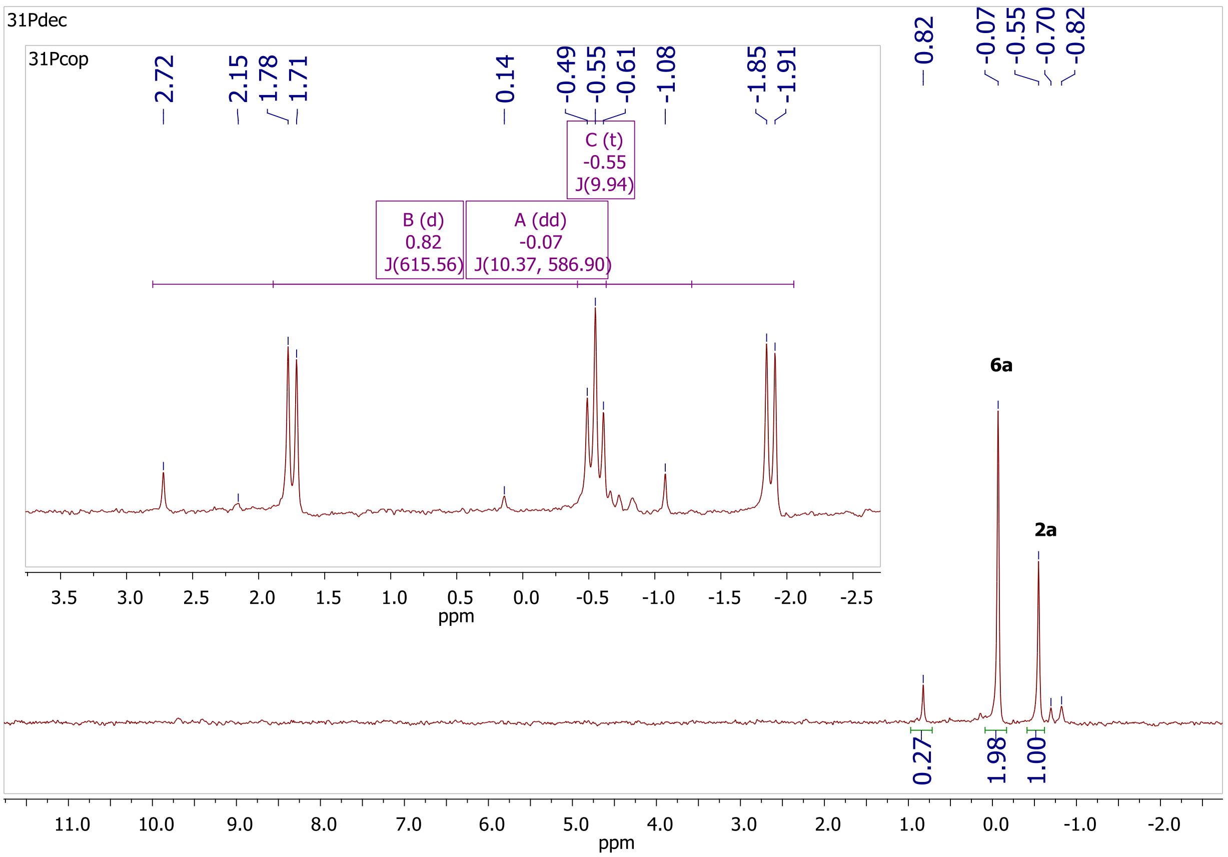

Figure S4. ^{31}P NMR spectrum of the mix **2a** and **6a** isolated in experiment with TEMPO (10 mol%).

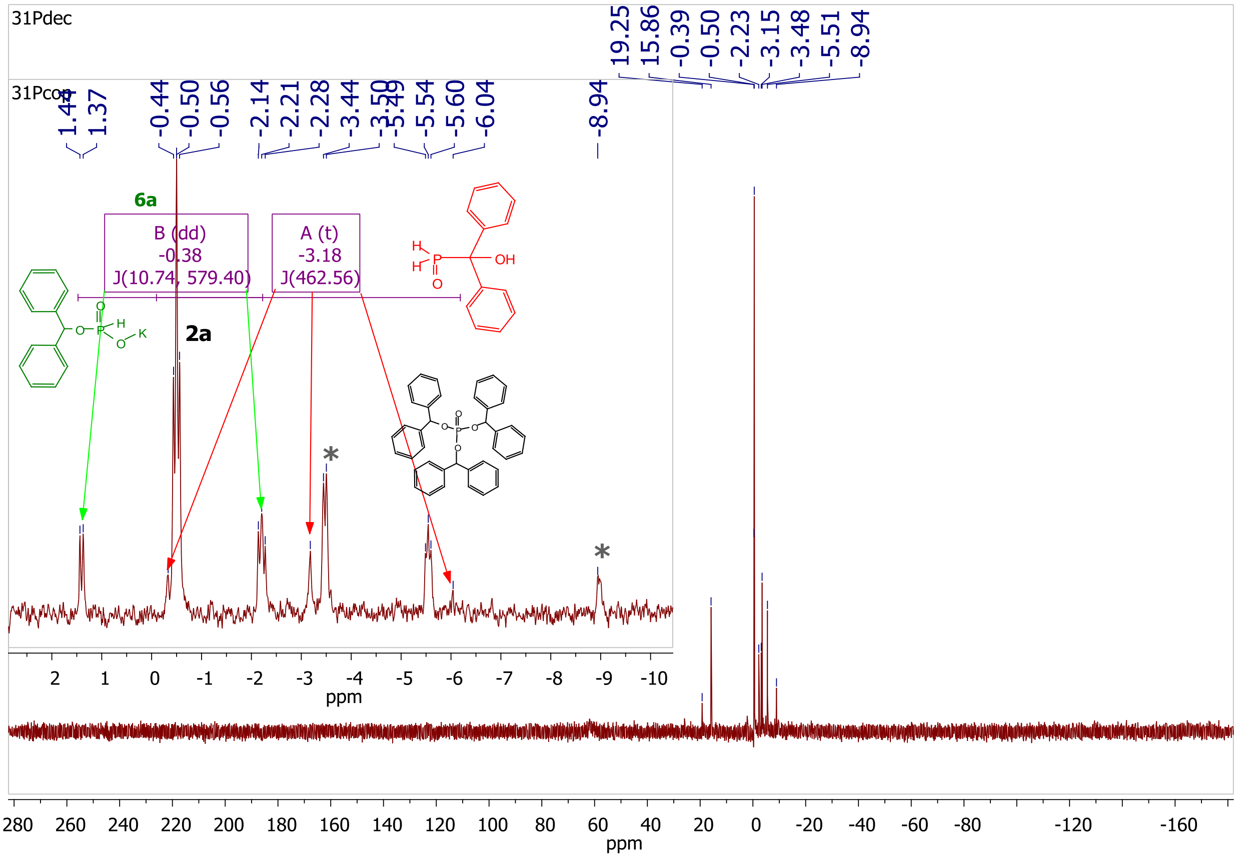

Figure S5. $^{31}P\{^1H\}$ NMR spectrum of the reaction mixture **1a**/P_{red}/KOH/DMSO(H_2O) in the presence of quinhydrone (10 mol%) at 85 °C in 1.5 h. Asterisks stands for unknown compounds.

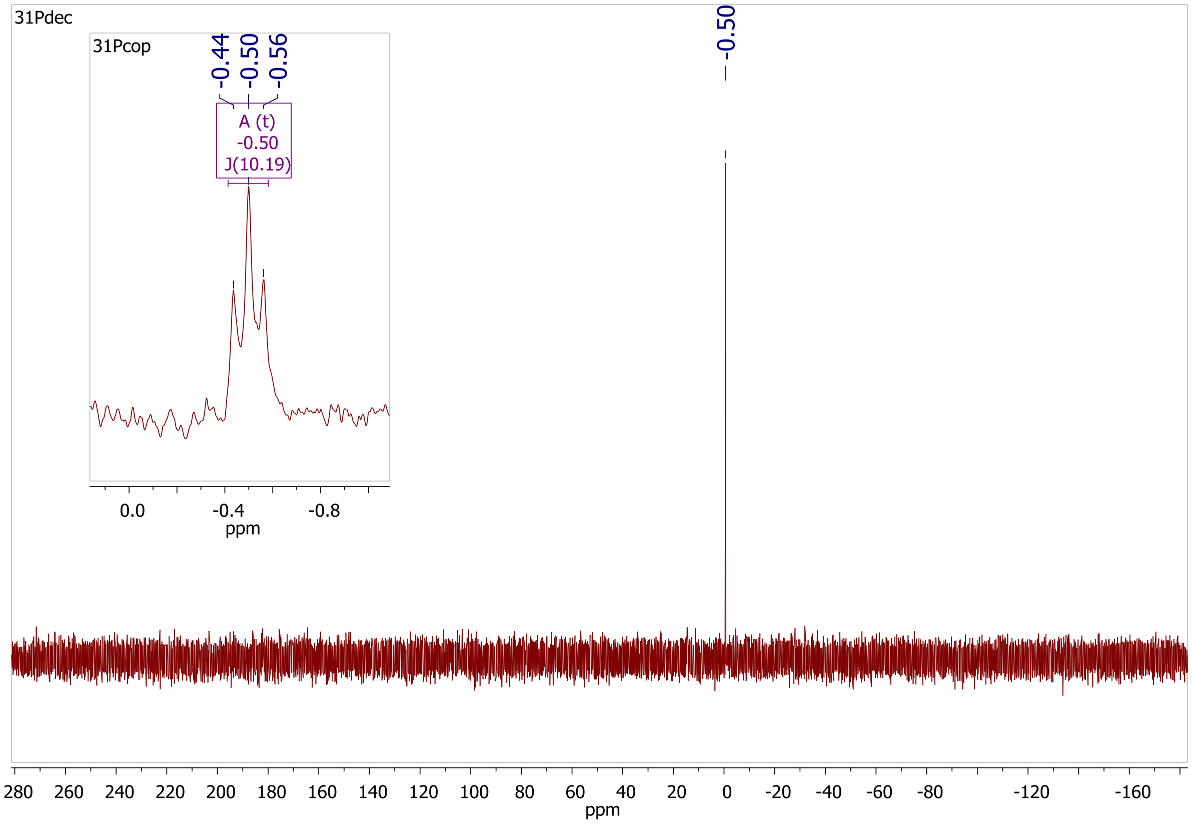

Figure S6. $^{31}P\{^{1}H\}$ NMR spectrum of the reaction mixture **1a**/P_{red}/KOH/DMSO(H_2O) at 85 °C in 1.5 h without any radical scavengers.

3.2. Reactions of (2- or 4-halo)benzophenones with red phosphorus

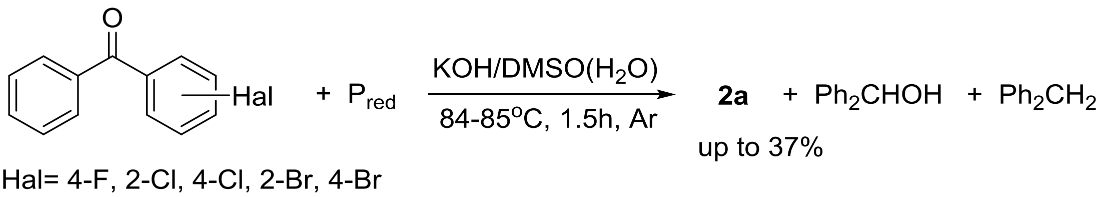

3.2.1. *Reaction of 2-chlorobenzophenone with red phosphorus*

A 250 mL round-bottom flask was sequentially charged with red phosphorus (73 mmol), 2-chlorobenzophenone (24 mmol), freshly machine-powdered KOH·0.5H_2O (154 mmol), H_2O (21 mmol) in DMSO (50 mL) and flushed with argon from the balloon. Reaction mixture was stirred (500-700 rpm) for 1.5 hours at 84-85 °C (oil bath). After the reaction completion, one part of water (25 mL) was added, and the reaction mixture was transferred into a separation funnel. Separated aqueous layer (~18 g) was discarded (which consists of inorganic phosphites and KOH), and new portion of water (25 mL) was added again to the reaction mixture. After extraction with $CHCl_3$ (3×25 mL) organic layer was separated and $CHCl_3$ and DMSO were distilled under reduced pressure. The resulting product was washed with diethyl ether (3×15 mL) and $CHCl_3$ (2×2 mL) to remove diphenylmethane and diphenylmethanol, dried in vacuum to give phosphate **2a** (2.08 g, 37%).

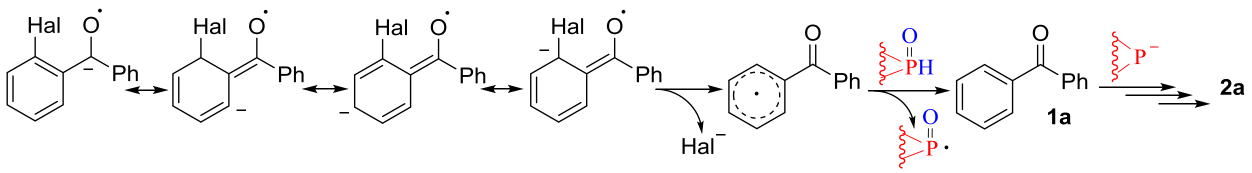

3.2.2. *Reaction of 2-bromobenzophenone with red phosphorus*

A 250 mL round-bottom flask was sequentially charged with red phosphorus (2.25 g, 73 mmol), 2-bromobenzophenone (6.27 g, 24 mmol), freshly machine-powdered KOH·0.5H_2O (10.00 g, 154 mmol), H_2O (0.38 g, 21 mmol) in DMSO (50 mL) and flushed with argon from the balloon. Reaction mixture was stirred (500-700 rpm) for 1.5 hours at 84-85 ^oC (oil bath). After the reaction completion, one part of water (25 mL) was added, and the reaction mixture was transferred into a separation funnel. Separated aqueous layer (~18 g) was discarded (which consists of KH_2PO_2, KH_2PO_3, K_2HPO_3 and KOH), and new portion of water (25 mL) was added again to the reaction mixture. After extraction with $CHCl_3$ (3×25 mL) organic layer was separated and $CHCl_3$ and DMSO were distilled under reduced pressure. The resulting product was washed with diethyl ether (5×15 mL) to remove diphenylcarbinol, the residue dried in vacuum to give phosphate **2a** (1.18 g, 21%). Product **2a** was determined on the corresponding spectra ^1H and ^{13}C NMR.

3.2.3. *Reaction of 4-bromobenzophenone with red phosphorus*

A 250 mL round-bottom flask was sequentially charged with red phosphorus (2.25 g, 73 mmol), 4-bromobenzophenone (6.27 g, 24 mmol), freshly machine-powdered KOH·0.5H_2O (10.00 g, 154 mmol), H_2O (0.38 g, 21 mmol) in DMSO (50 mL) and flushed with argon from the balloon. Reaction mixture was stirred (500-700 rpm) for 1.5 hours at 84-85 ^oC (oil bath). After the reaction completion, one part of water (25 mL) was added, and the reaction mixture was transferred into a separation funnel. Separated aqueous layer (~18 g) was discarded (which consists of inorganic phosphites and KOH), and new portion of water (25 mL) was added again to the reaction mixture. After extraction with $CHCl_3$ (3×25 mL) organic layer was separated and $CHCl_3$ and DMSO were distilled under reduced pressure. The resulting product was washed with diethyl ether (5×15 mL) to remove diphenylcarbinol, the residue dried in vacuum to give phosphate **2a** (0.96 g, 17%). Product **2a** was determined on the corresponding spectra ^1H and ^{13}C NMR.

3.2.4. *Reaction of 4-fluorobenzophenone with red phosphorus*

A 250 mL round-bottom flask was sequentially charged with red phosphorus (2.25 g, 73 mmol), 4-fluorobenzophenone (4.82 g, 24 mmol), freshly machine-powdered KOH·0.5H_2O (10.00 g, 154 mmol), H_2O (0.38 g, 21 mmol) in DMSO (50 mL) and flushed with argon from the balloon. Reaction mixture was stirred (500-700 rpm) for 1.5 hours at 84-85 ^oC (oil bath). After the reaction completion, one part of water (25 mL) was added, and the reaction mixture was transferred into a separation funnel. Separated aqueous layer (~18 g) was discarded (which consists of inorganic phosphites and KOH), and new portion of water (25 mL) was added again to the reaction mixture. After extraction with $CHCl_3$ (3×25 mL) organic layer was separated and $CHCl_3$ and DMSO were distilled under reduced pressure. The resulting product was washed with diethyl ether several times to remove diphenylcarbinol, the residue dried in vacuum to give

phosphate **2a** (0.79 g, 14%). Product **2a** was determined on the corresponding spectra ^{1}H and ^{13}C NMR.

3.3. EPR experiments

To get a deeper insight into the mechanism, electron paramagnetic resonance (EPR) experiments were performed (Fig. S8-10). The observed *g*-factors (Fig. S8) for the spin adducts were very close to 2.0023 for free electrons, which provided more conclusive evidence for the existence of radicals. The results of EPR experiments and the above radical trapping experiments showed the experimental mechanism may involve a free radical mechanism (see the paper).

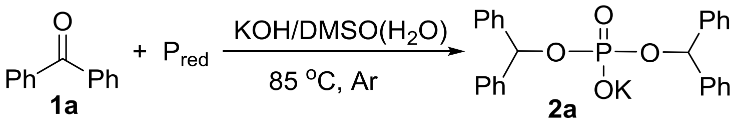

A 50 mL three-necked flask containing a mechanical stirrer, thermometer and small reflux condenser (for gas removal), was charged with benzophenone (0.87 g, 4.8 mmol), P_{red} (0.45 g, 14.5 mmol) and freshly machine-powdered $KOH{\cdot}0.5H_2O$ (2.00 g, 30.8 mmol) and water (0.08 g, 4.4 mmol) in DMSO (10 mL). After the flask was purged with argon, the resulting mixture was stirred under at 85 °C (oil bath) for 1.5 hours. The ~0.1 mL of the reaction solution was directly used for EPR testing without any purification. EPR spectra was recorded at room temperature on an ELEXSYS E-580 EPR spectrometer (Bruker Corporation, Billerica, MA) operated at 9.7 GHz. The EPR-spectra were recorded at the following conditions in thin glass capillaries (diameter of 1 mm): amplitude modulation 0.05 G, modulation frequency 100 kHz, receiver gain 50-60 dB, time constant 0.02 s, conversion time 0.06 s, field range 20 – 30 G / centre field 3350 G, averaged scans 5, microwave power 0.6325 mW.

A signal of the benzophenone anion-radical was detected in the reaction mixture using the ESR technique (Fig.S8). First, two overlapped ESR signals from the benzophenone anion-radical and, probably, from the polyphosphinyl radical ($>P_n\cdot$) were observed (Fig. S10). Using interval recording of the spectrum during a couple of hours, it was possible to separate the two signals from each other. The signal of the benzophenone anion-radical (Fig.S9) corresponded almost exactly to the simulated version (Fig. S11) and the known data.(14) In the latter work it was shown that almost 80% of the unpaired electron was delocalized in the phenyl rings. The maximum spin density of the unpaired electron is on the carbon atoms in the 4-position, somewhat less spin density is located in the 2-position and only an insignificant part in the 3-position. This is in good agreement with the fact that only potassium 3-halobenzhydrylphosphates **2j, 2k** from 3-halogenated benzophenones **1j,k** were obtained by our method, whereas for 2- and 4-halobenzophenones complete reductive dehalogenation occurred to form only phosphate **2a** (see 3.2.). The polyphosphinyl radical is presented in ESR spectrum by multiplets that corresponds to the interaction of several phosphorus atoms. Since P atom has spin ½ then according to multiplicity rules(15) the spectrum should look as shown in Fig. S9.

In the ^{31}P NMR spectra (Fig. S12, S13) of the reaction mixture one observed broad signals at -106 ppm together with a set of signals from 1.34 to -1.32 ppm range probably belong to polyphosphinyl radical ($>P_n\cdot$) (lit. data for some phosphorus clusters, δ_P ($^{-3}P_7$) = -117 (DMF), -120 (THF) ppm).(16)

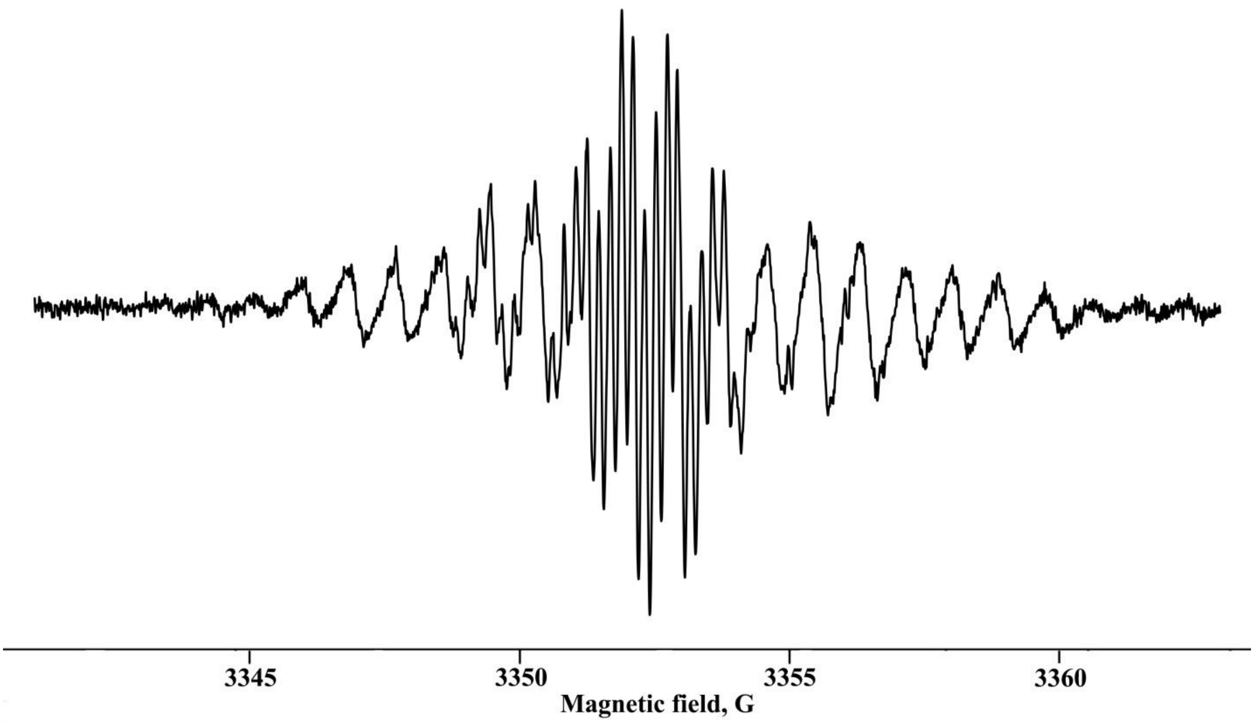

Figure S7. ESR spectrum of the mixed anion-radical **1a** and proposed polyphosphinyl radical (>P_n·)

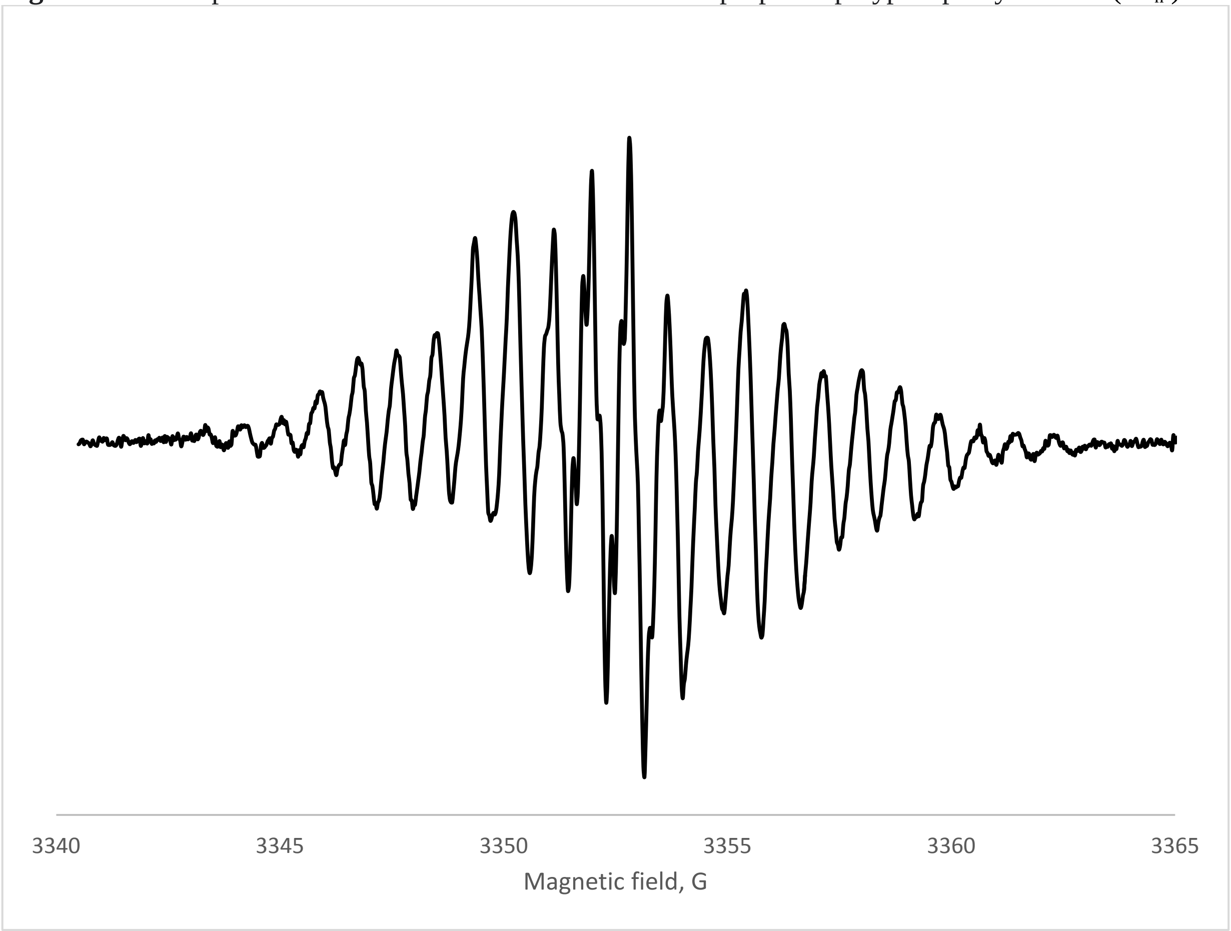

Figure S8. ESR spectrum of the anion-radical of benzophenone

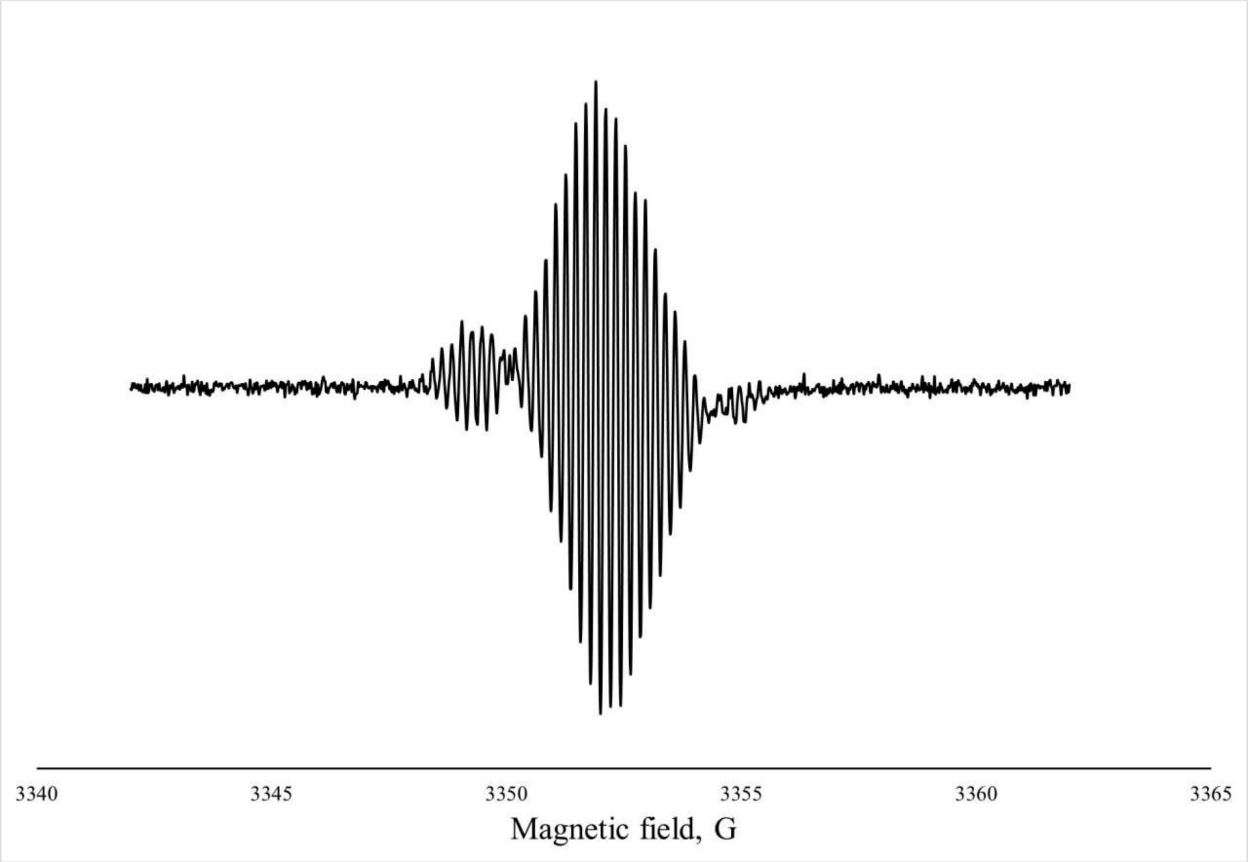

Figure S9. ESR spectrum of the proposed polyphosphinyl radical (>P_n·)

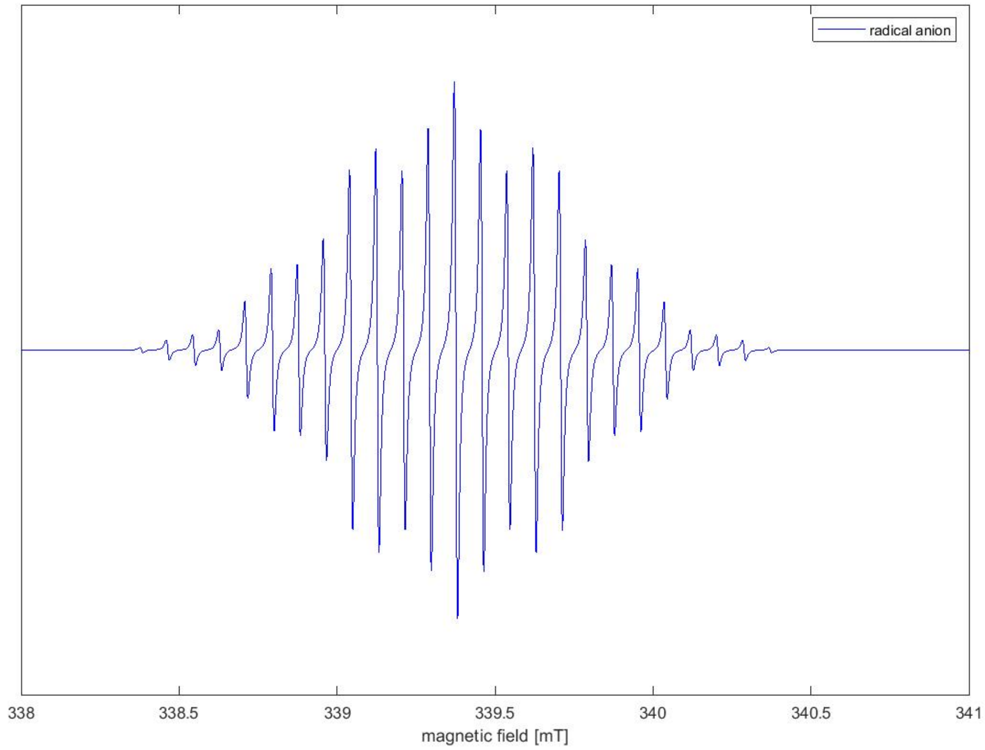

Figure S10. Simulation of the spectrum of the benzophenone anion-radical

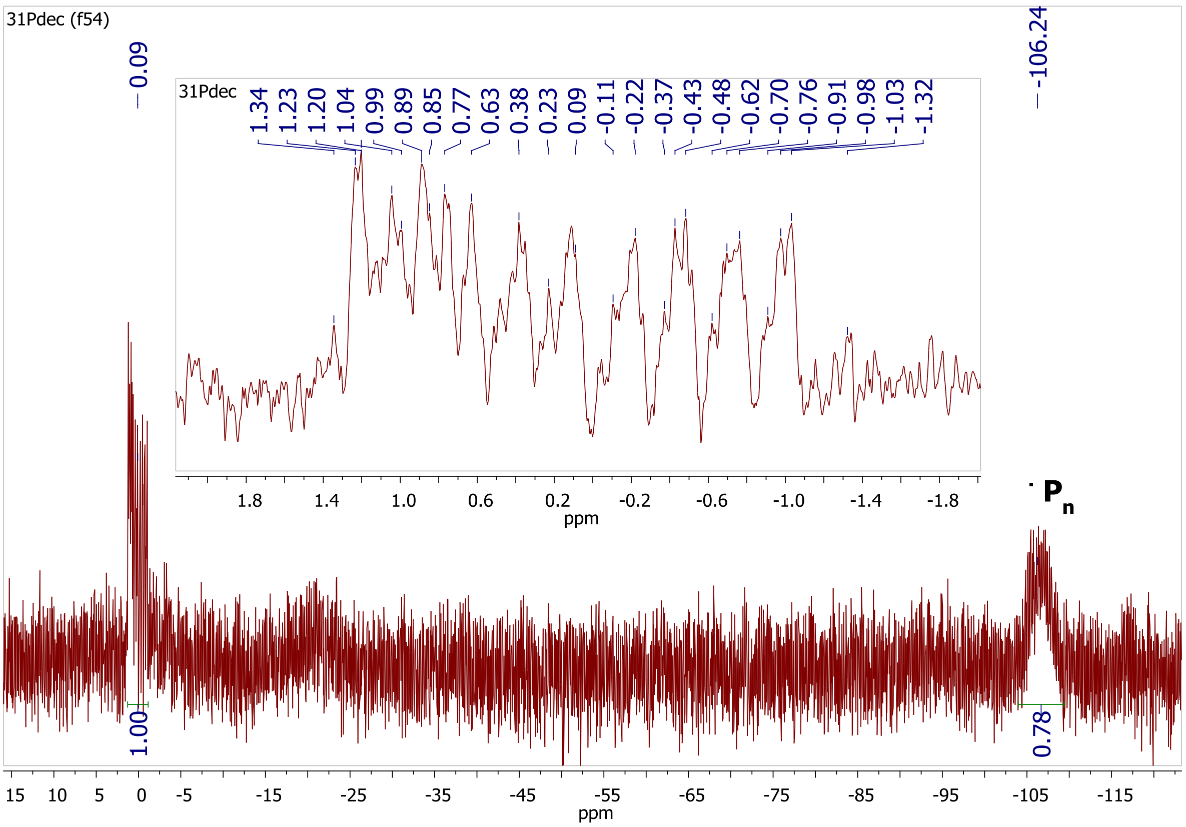

Figure S11. ^{31}P NMR {^{1}H} of the of the reaction mixture P_{red}/**1a**/KOH/DMSO/H_2O in 30 min after the reaction beginning.

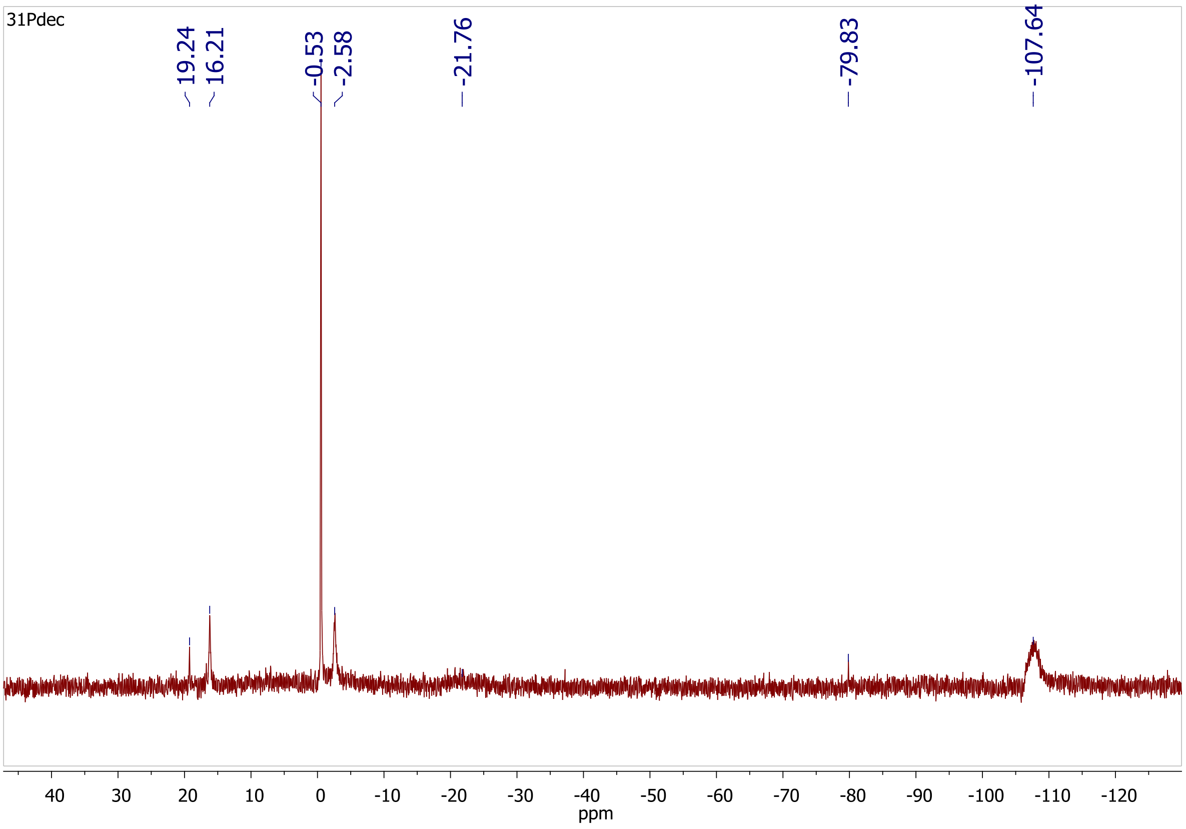

Figure S12. ^{31}P NMR {^{1}H} of the of the reaction mixture P_{red}/**1a**/KOH/DMSO/H_2O in 90 min after the reaction beginning.

3.4. Evaluation of 2a stability

A 250 mL round-bottom flask was sequentially charged with **2a** (0.201 g, 0.43 mmol), freshly machine-powdered $KOH \cdot 0.5H_2O$ (2.08 g, 32.00 mmol), H_2O (4 mmol) in DMSO (10 mL) and flushed with argon from the balloon. Reaction mixture was stirred (500 rpm) for 1.5 hours at 84-85 ^oC (oil bath). After the reaction completion, the flask was cold down to r.t., water (40 mL) was added. After extraction with $CHCl_3$ (3×20 mL), the organic layer was separated and $CHCl_3$ and DMSO were distilled under reduced pressure. The resulting product was washed with diethyl ether (3×5 mL) and dried under vacuum, and 0.128 g of **2a** was recovered (64%). From ether extract ketone **1a** (0.014 g) was isolated in 18% yield. Benzhydrol (0.019 g, 12%) was isolated after acidification of aq. layer.

4. X-Ray diffraction analysis of 2a

The X-ray diffraction data were obtained on a Bruker Kappa Apex II CCD diffractometer (Bruker Corp., USA) using φ-ω scans of narrow (0.5°) frames with MoKα radiation (λ = 0.71073 Å) and a graphite monochromator at room temperature (296 K). The structures was solved by direct methods using the SHELXT-2014/5(17) and refined by full-matrix least-squares method against all F2 in anisotropic approximation using the SHELXL-2018/3.(17) The hydrogen atoms positions were calculated with the riding model. Absorption corrections were applied using the empirical multiscan method with the SADABS program.(18) Compound **2a** is triclinic, space group P-1, *a* = 6.0255(4), b = 12.5562(10), c = 16.7667(14), α = 108.862(3), β = 94.573(3), γ = 103.728(3)°, V = 1149.10(15) $Å^3$, Z = 2, $C_{26}H_{22}O_4PK$, formula weight 468.50, crystal density Dc = 1.354 g/sm^3, μ = 0.331 mm-1, F(0 0 0) = 488, yellow crystal size 0.90 × 0.10 × 0.06 mm^3, independent reflections 5306 (Rint = 0.0462), wR_2 = 0.1090, goodness of fit S = 1.02 for all reflections and R = 0.0386 for 4249 I > 2σ, difference electron density max is 0.32 and min is -0.39 e-/$Å^3$. CCDC **2366416** contain the supplementary crystallographic data for this paper. These data can be obtained free of charge from The Cambridge Crystallographic Data Centre (CCDC) via www.ccdc.cam.ac.uk/data_request/cif. Molecular structure of the crystalographically independent part of compound **2a** is illustrated in Figure 2 in paper and in Fig.S11a. The obtained crystal structure were analyzed for the geometrical parameters and short contacts between non-bonded atoms using PLATON(19) and MERCURY programs.(20) The geometric parameters agreed within 3σ of the corresponding mean statistical values,(21) the all phenyl cycles are perfectly planar in the crystal.

Single crystal of potassium phosphate **2a** was grown by a method of slow liquid diffusion as follows. To a solution of potassium phosphate (70 mg) in DMF (0.5 ml), n-hexane (0.5 ml) was added to form two-phase system, which was allowed evaporating slowly through non-tightly closed cap during 4 weeks. The crystals formed were dried on air and several most perfect single crystals were selected for X-ray analysis.

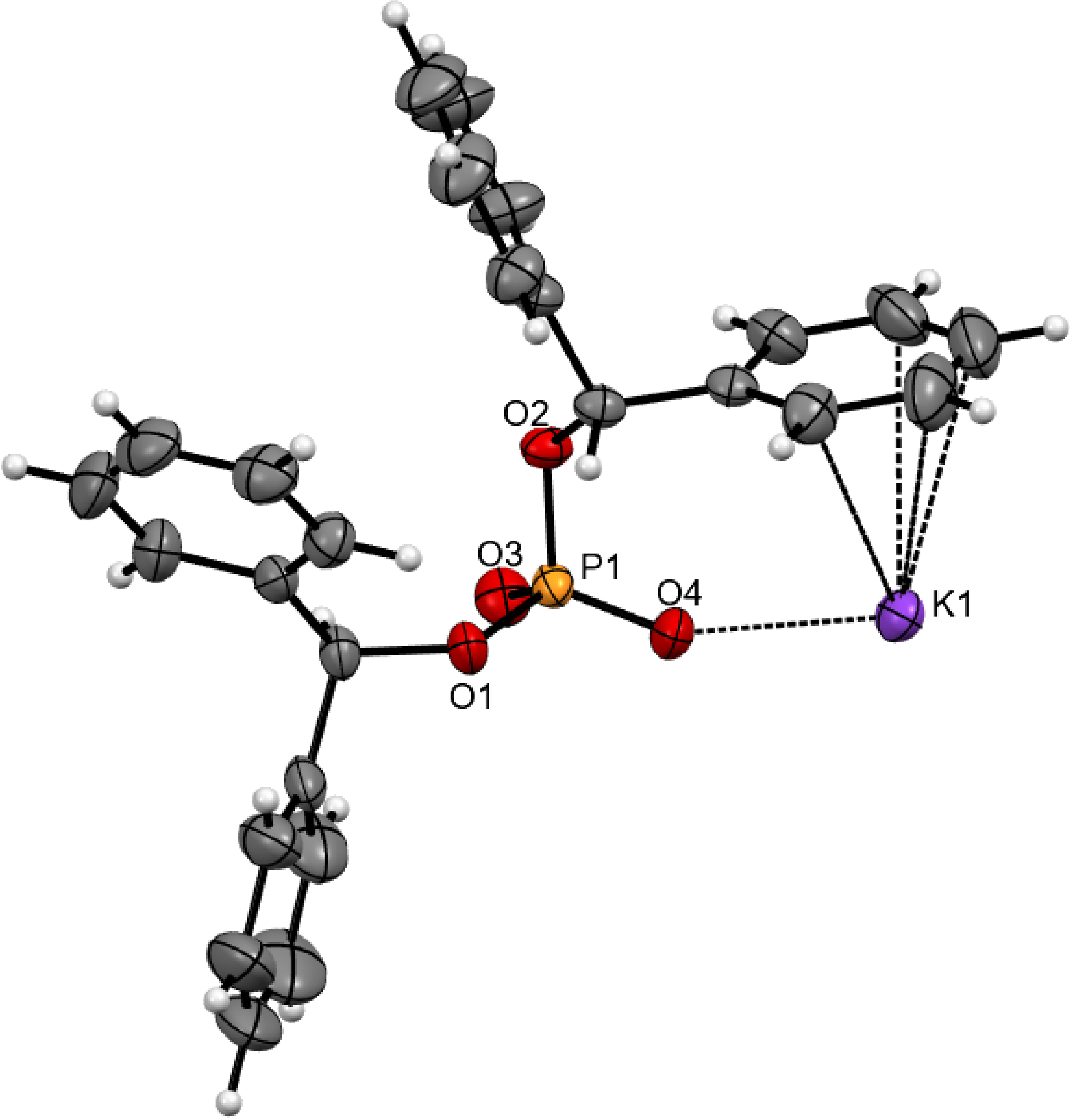

Figure S13. a) Molecular structure of the crystalographically independent part for **2a** (50% thermal ellipsoids are shown);

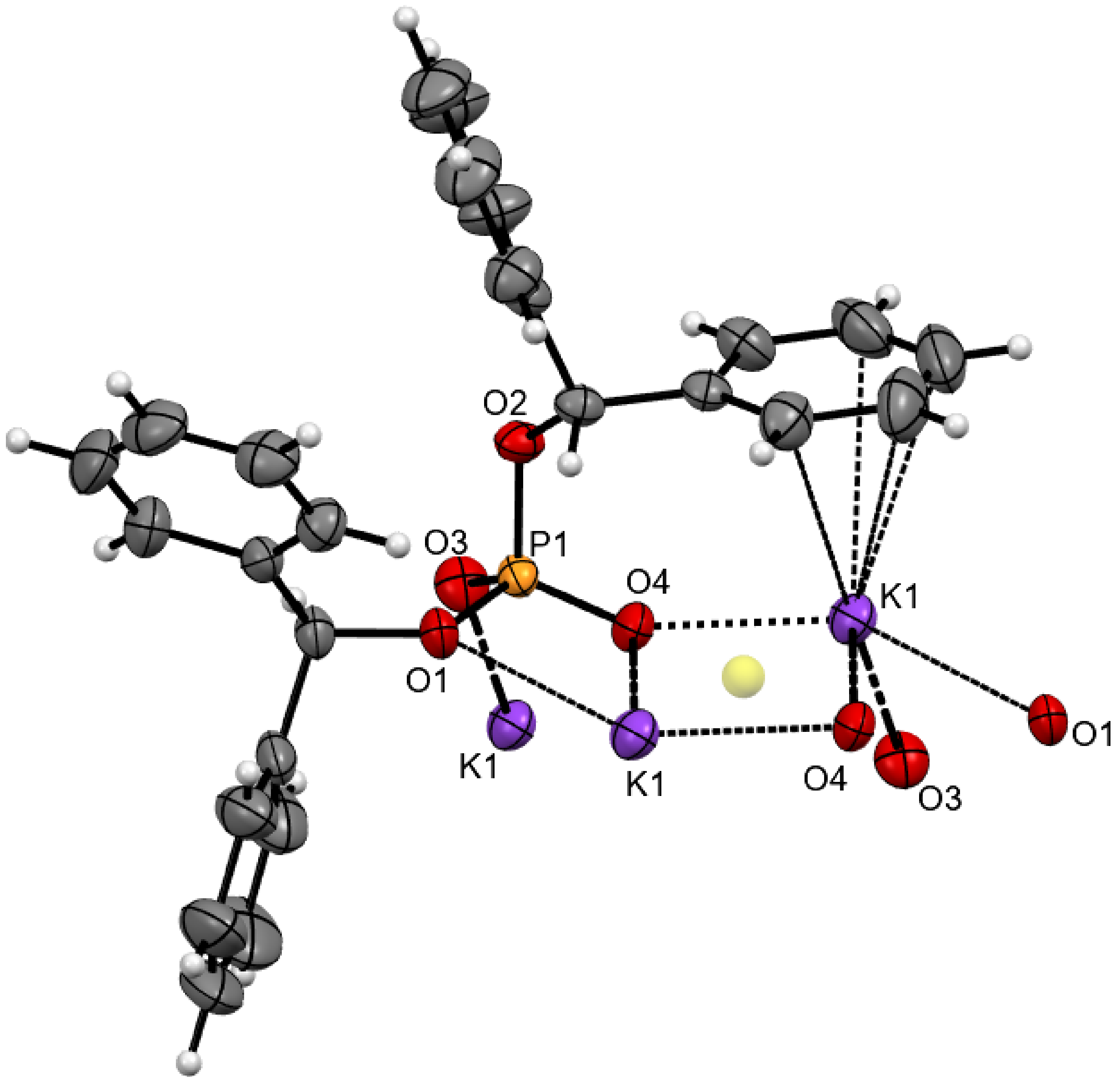

Figure S14. Repeating fragment of polymer chain. Center of inversion is shown (yellow color)

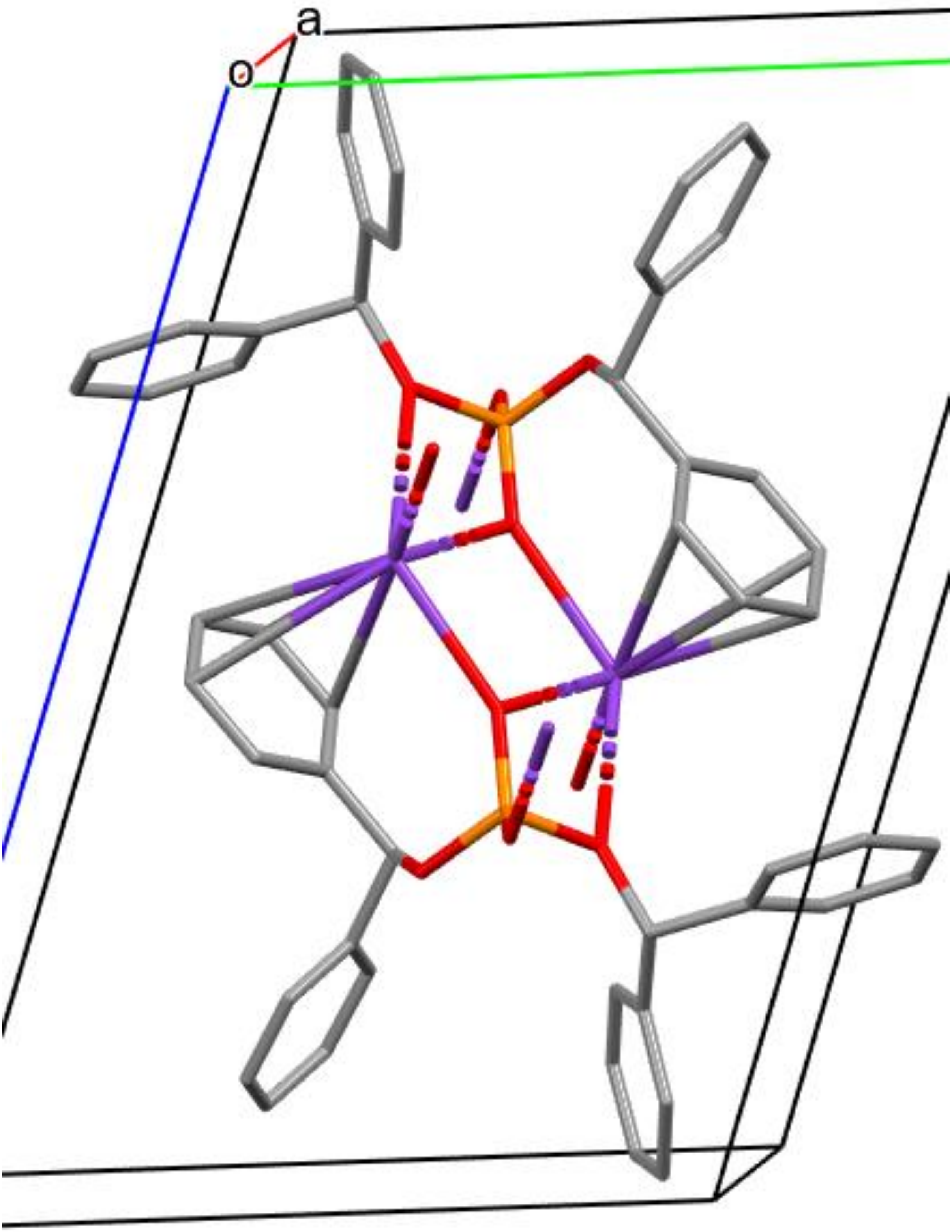

Figure S15. Structure of the coordination dimer for **2a** (hydrogen atoms are omitted for clarity)

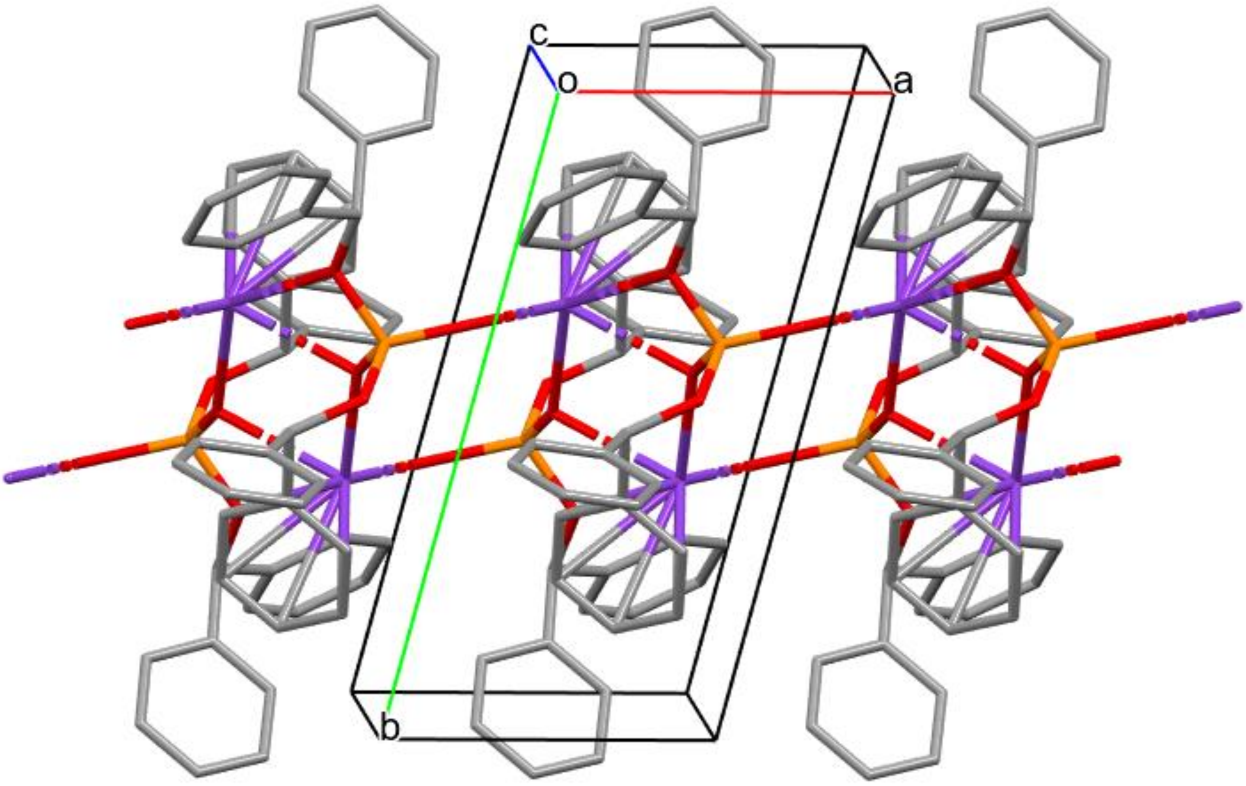

Figure S16. Structure of the chained coordination polymer **2a** along the crystallographic *a* (hydrogen atoms are omitted for clarity).

5. Unsuccessful Substrates

The ketones with stronger π-donors in a one of benzene ring such as 4-Me_2N-, 4-OH, 4-$C_6H_{13}O$, 4-$MeSCH_2O$ did not afford phosphorylation product and only in some cases, small amounts of the corresponding carbinols were detectable (^{1}H NMR) in the reaction mixtures, that is in consistent with the ion-radical character of the whole process. In the cases of 4-OVin, 4-OAll substituence in a one of benzene ring, the oligomerization over the double bonds likely prevails, since nether carbinols no phosphorylated products were formed. A condense aromatic substituents (1-naphthyl) directed the reaction mainly towards the reduction processes providing 51% yield of corresponding carbinol and diarylmethane whereas the phosphorylation product was formed only in 7% yield. This is results from the anticipated steric shielding of the radical anion center in the initial intermediate by the bulky naphthyl substituents, that almost prevents the attack by P-centered free-radicals. This is support by the fact that Ph-2-MeO-naphthyl ketone under the aforementioned conditions was not phosphorylated at all, giving the corresponding carbinol in 32% yield. Expectedly, fatty aromatic ketones under the same were not able to be phosphorylated owing to the domination of the aldol-krotone processes. The testing ketones there are: acetophenone, dibenzylketone, ([1,1'-biphenyl]-4-yl)(benzyl)ketone, anthrone, 9-fluorenone, 1,2,3,4-tetrafluoro-9-fluorenone.

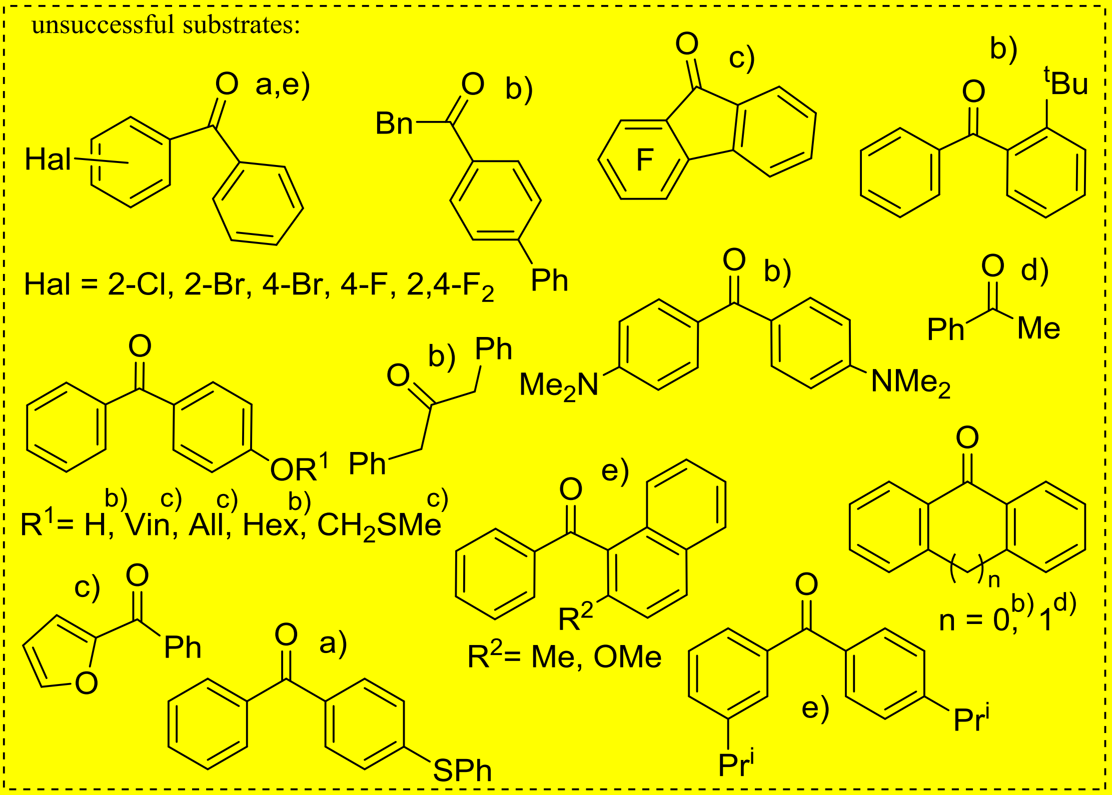

Figure S17. a) reductive elimination of substituent occurs; b) no conversion, the ketone is recovered almost completely; c) irreversible transformations occur, the ketone was not recovered; d) only partially recovered; e) reduction of ketone takes place.

6. Spectral data of the synthesized products

Potassium bis(diphenylmethyl)phosphate (2a).

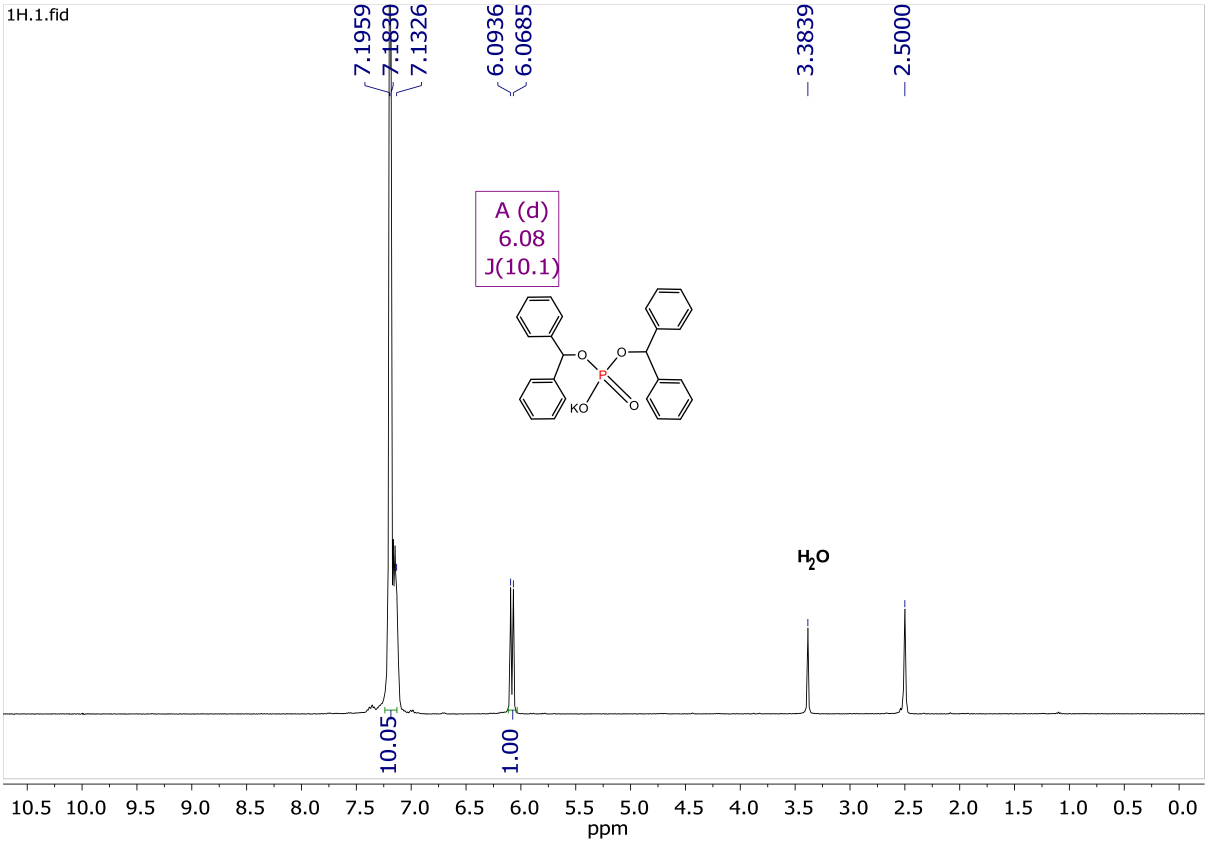

^{1}H NMR spectrum of **2a** (DMSO_d_6).

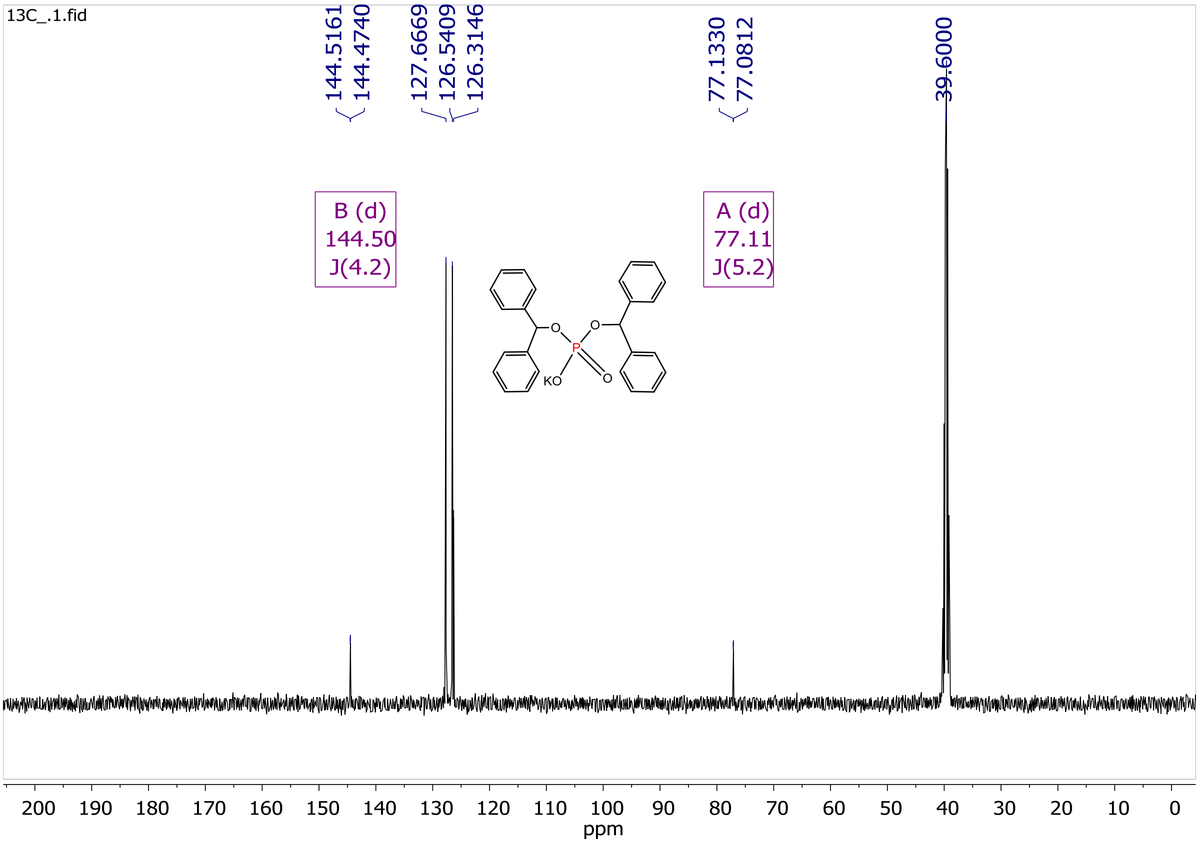

^{13}C NMR spectrum of **2a** (DMSO_d_6).

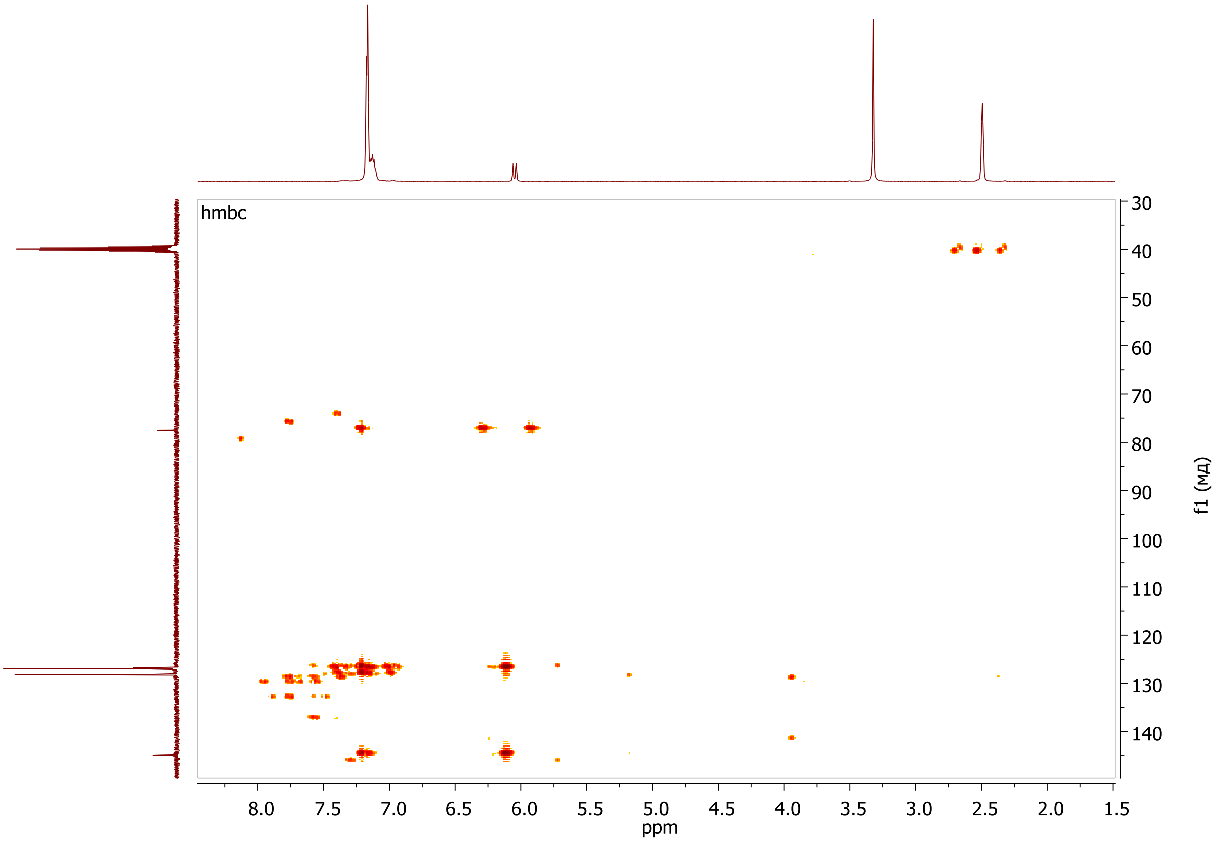

2D HMBC ^{13}C-^{1}H NMR spectrum of **2a** (DMSO_d_6).

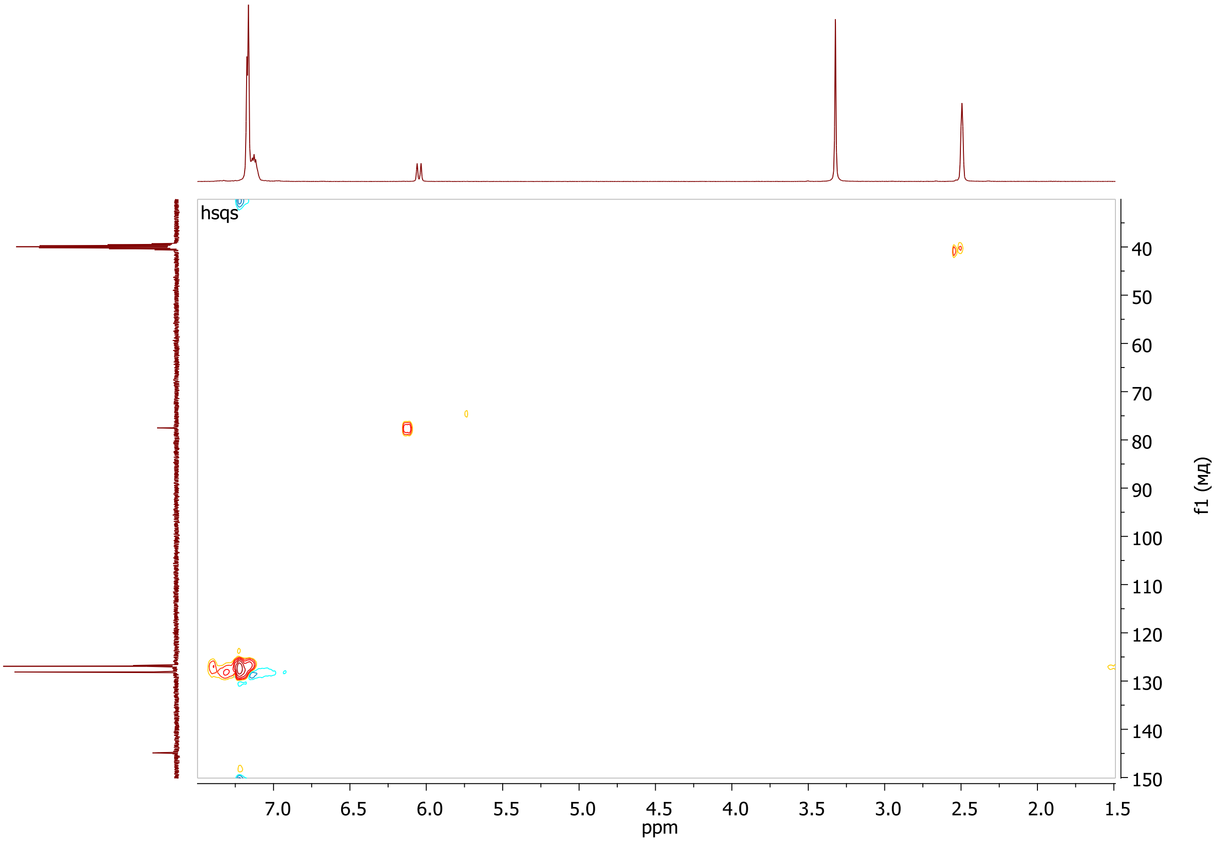

2D HSQC ^{13}C-^{1}H NMR spectrum of **2a** (DMSO_d_6).

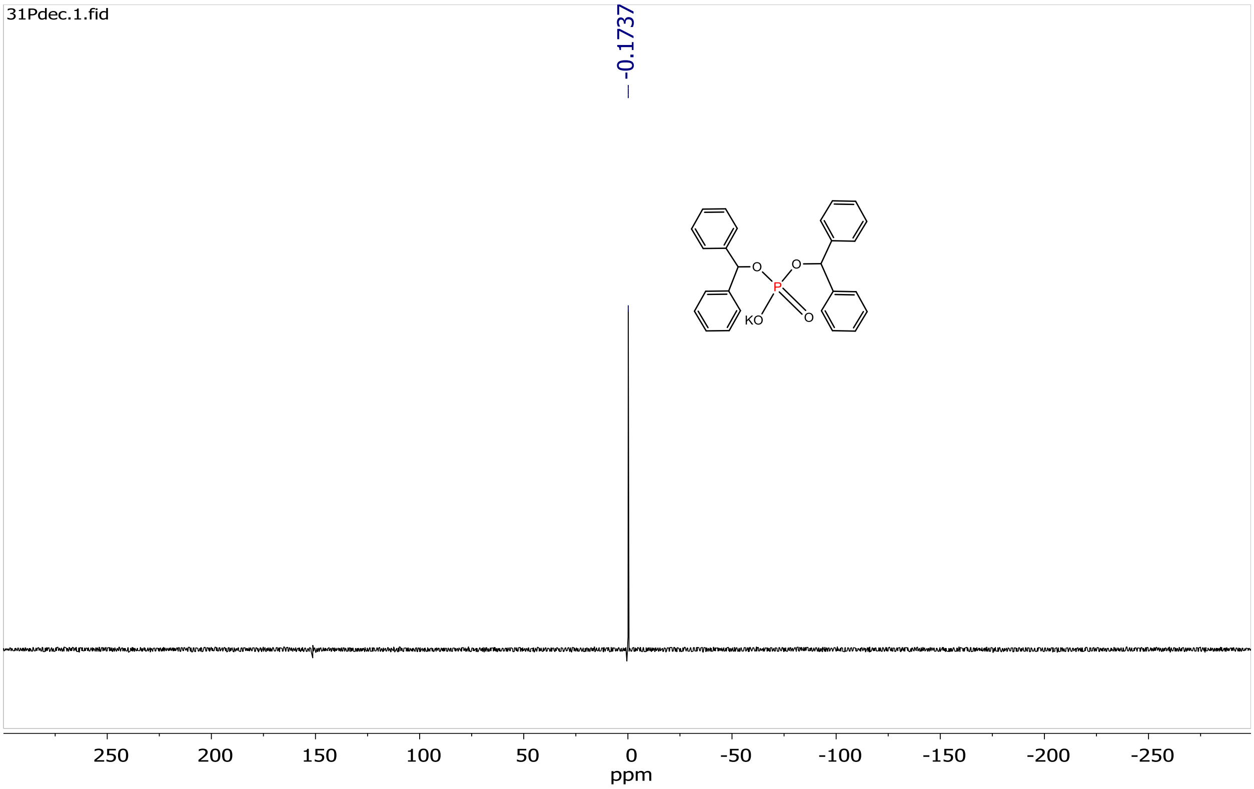

$^{31}P\{^{1}H\}$ NMR spectrum of **2a** (DMSO_d_6).

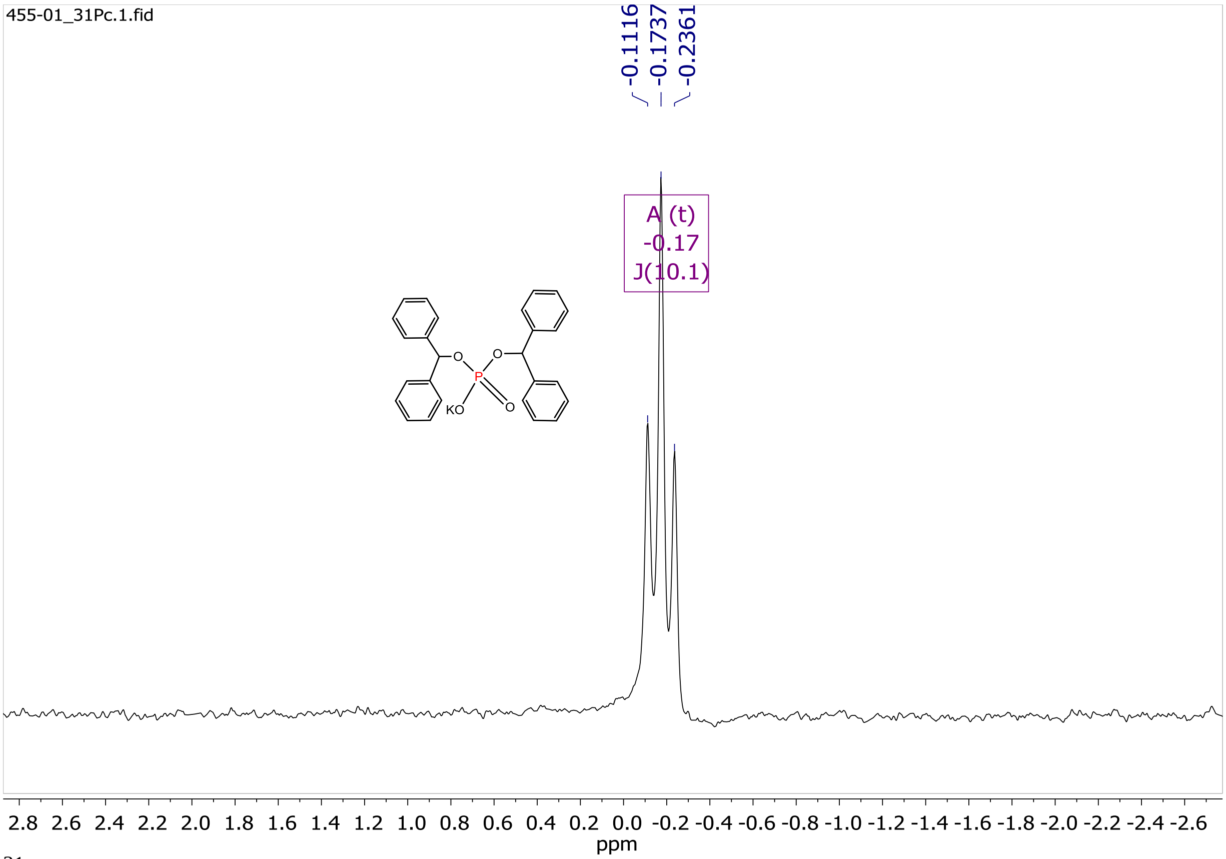

^{31}P NMR spectrum of **2a** (DMSO_d_6).

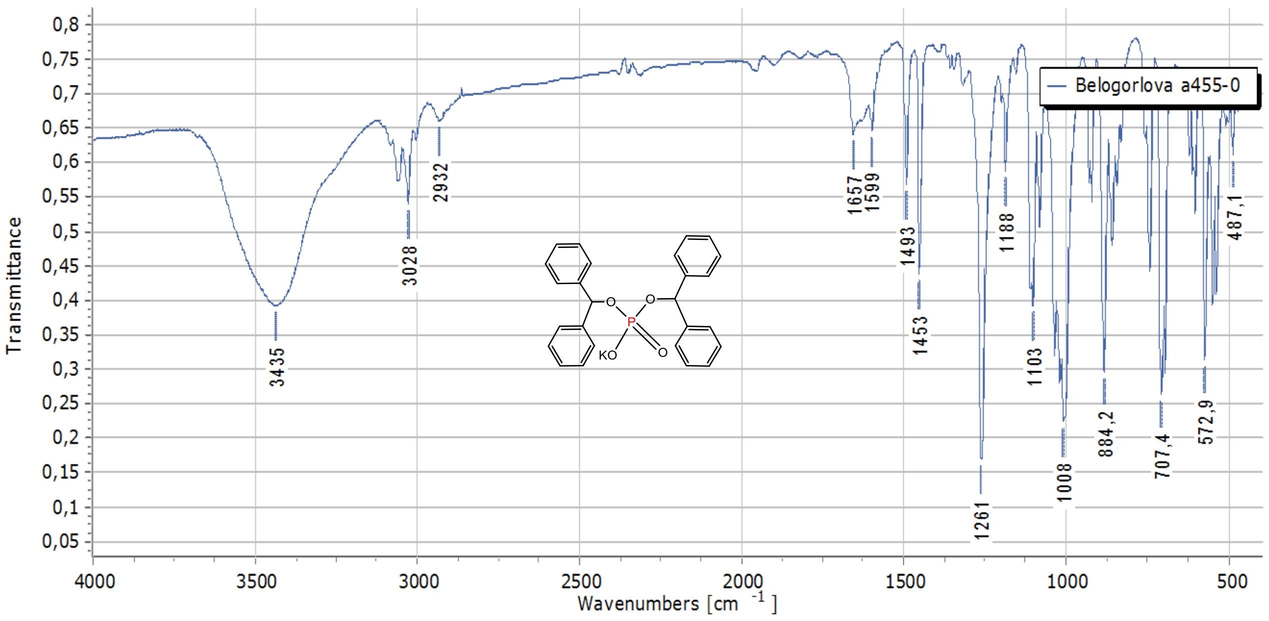

IR spectrum of **2a** (KBr, cm^{-1}).

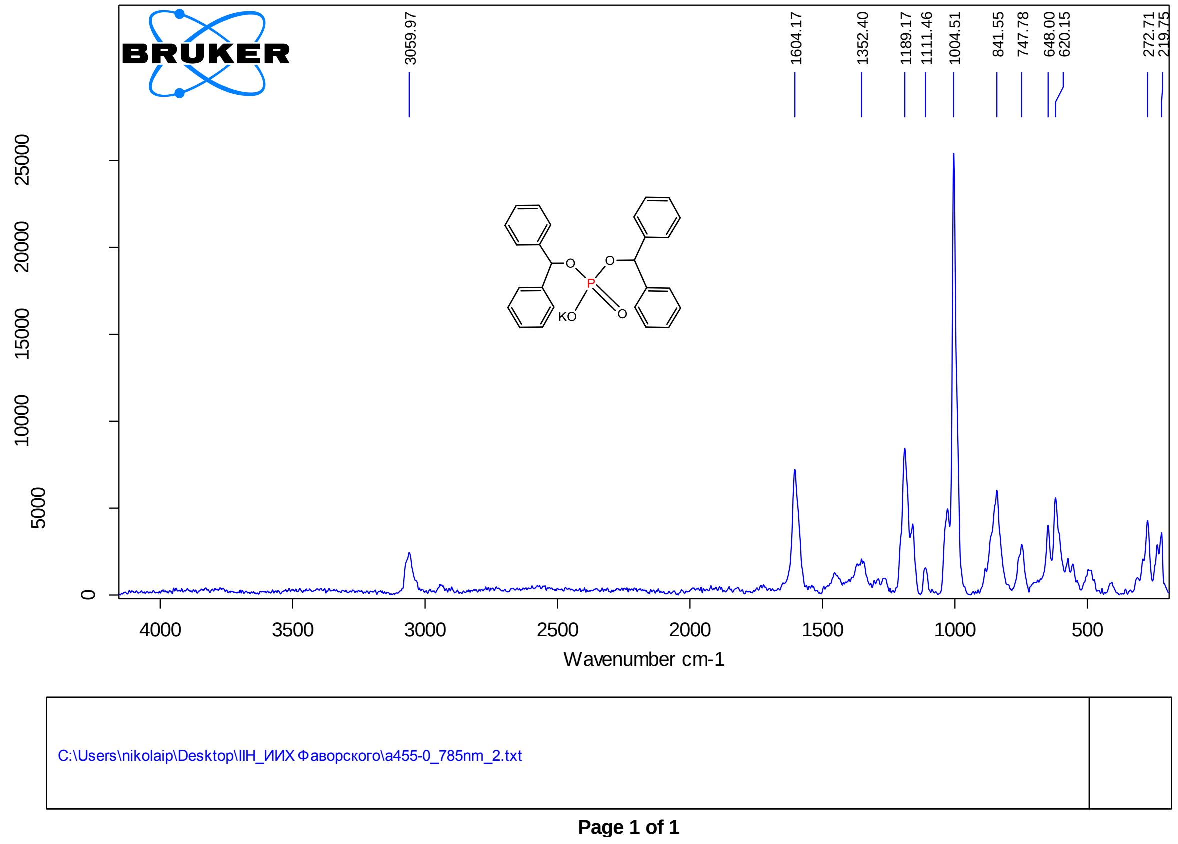

Raman spectrum of **2a** (785 nm).

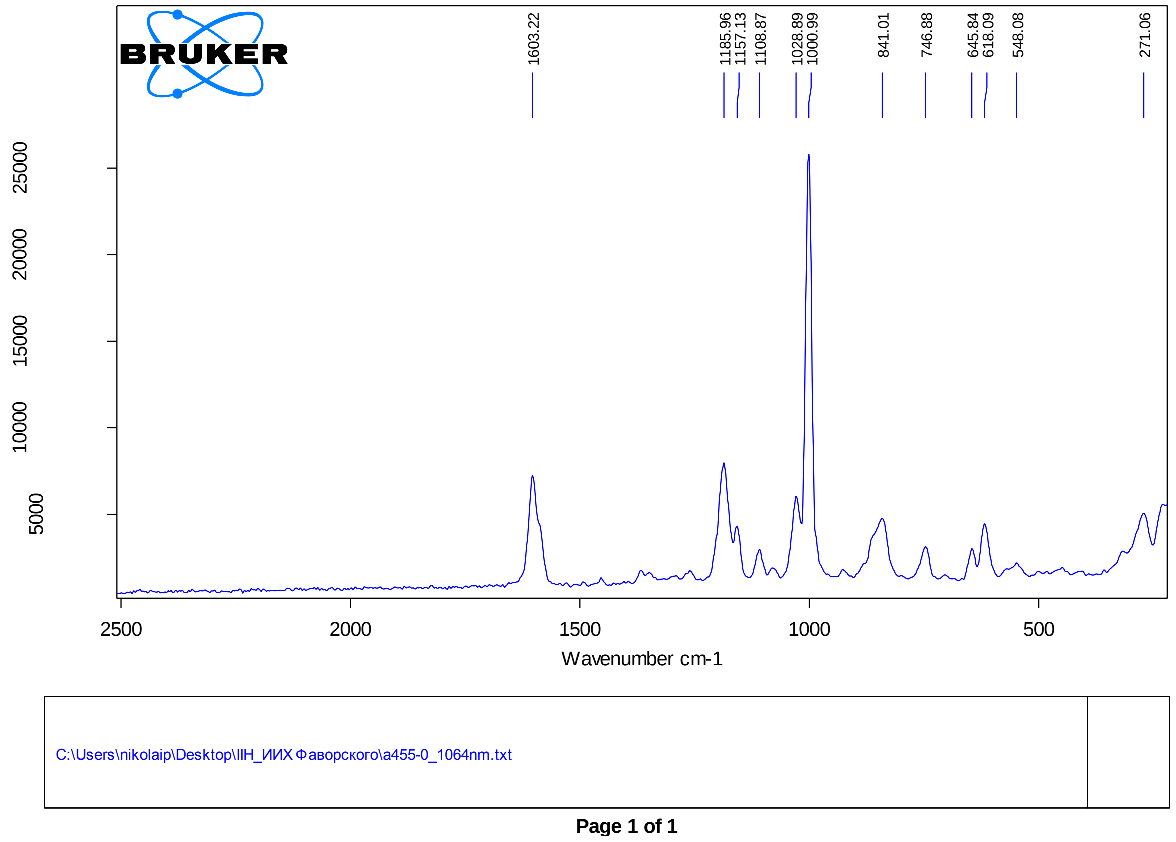

Raman spectrum of **2a** (1064 nm).

Potassium bis[(2-methylphenyl)(phenyl)methyl]phosphate (2b).

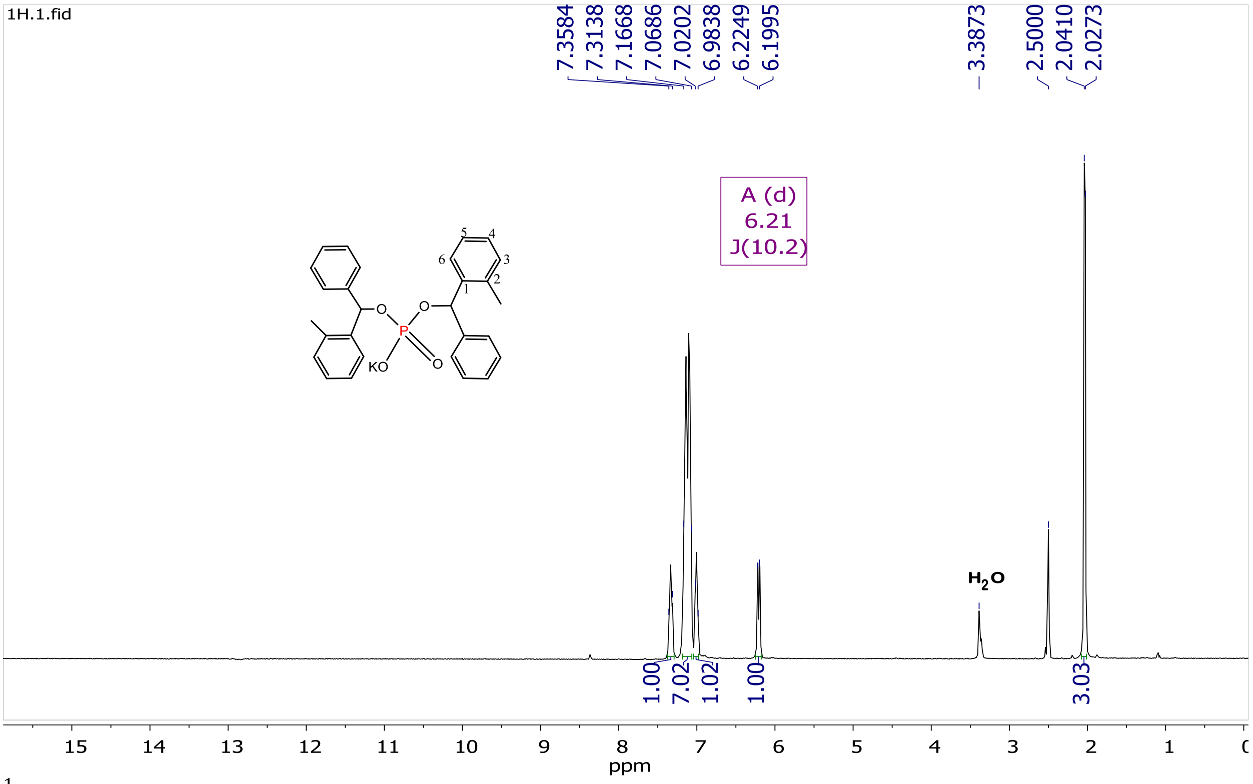

^{1}H NMR spectrum of **2b** ($DMSO\_d_6$).

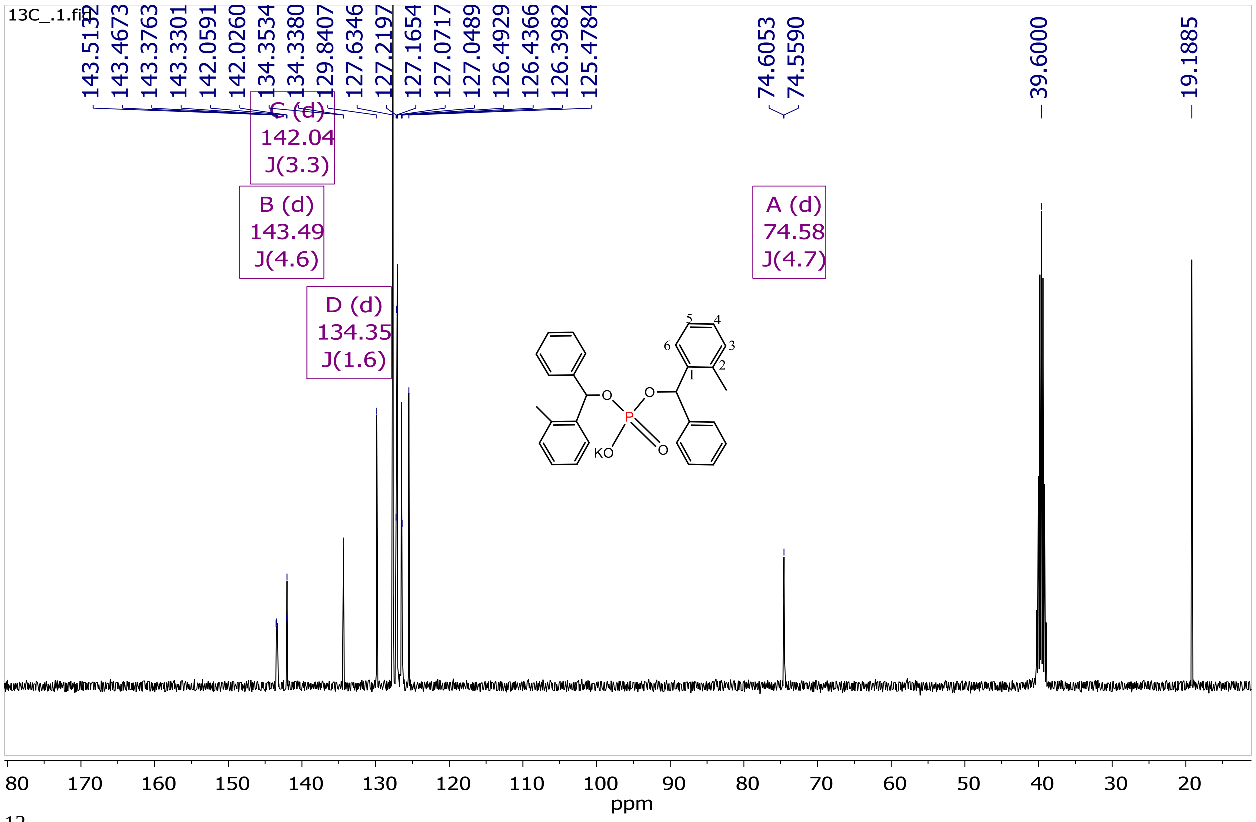

^{13}C NMR spectrum of **2b** (DMSO_d$_6$).

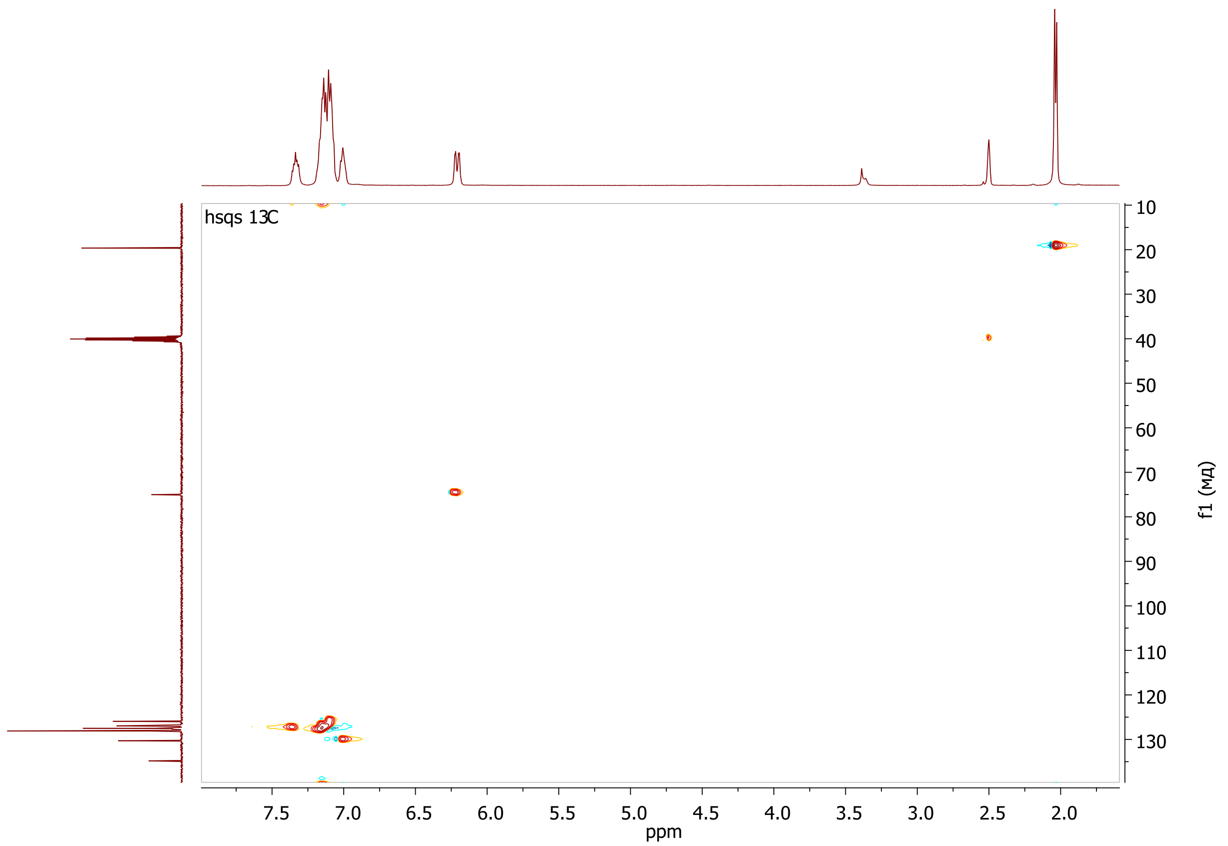

2D HSQC ^{13}C-^{1}H NMR spectrum of **2b** (DMSO_d_6).

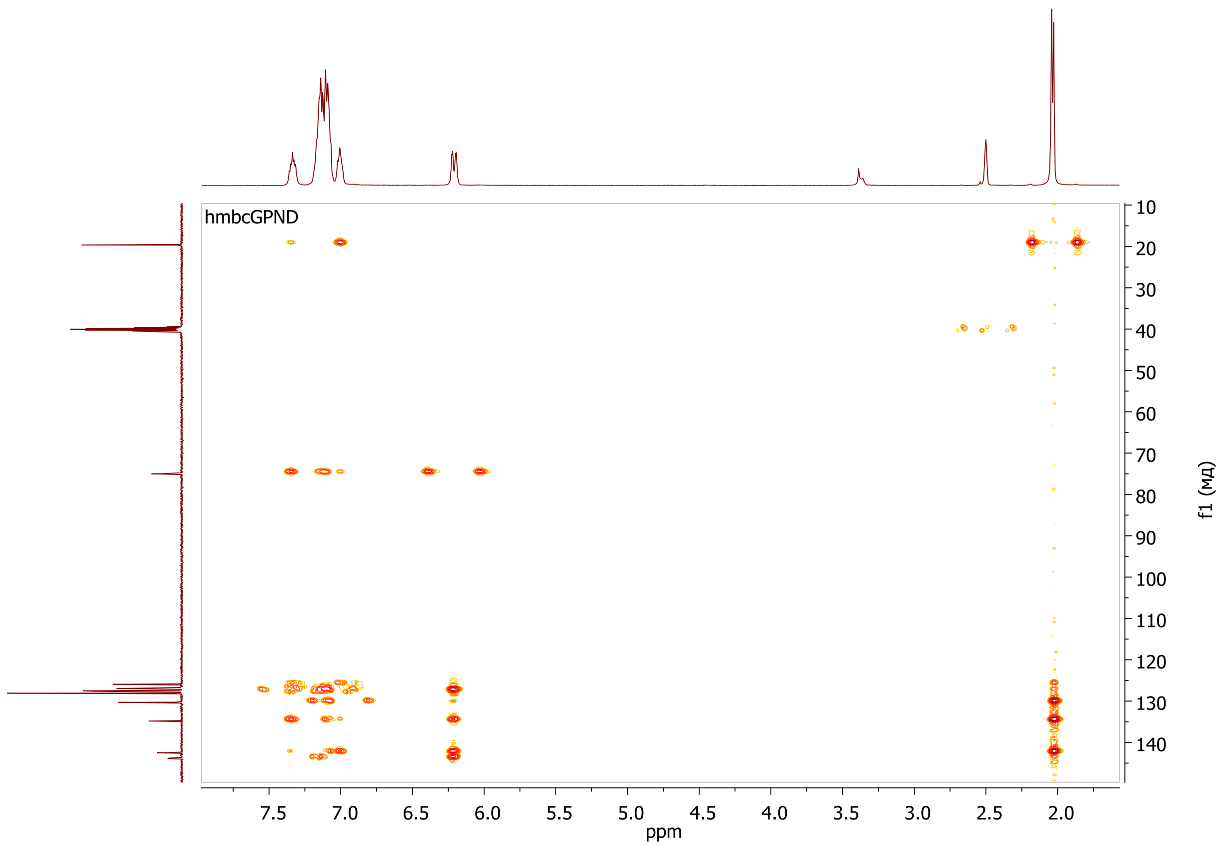

2D HMBC ^{13}C-^{1}H NMR spectrum of **2b** (DMSO_d_6).

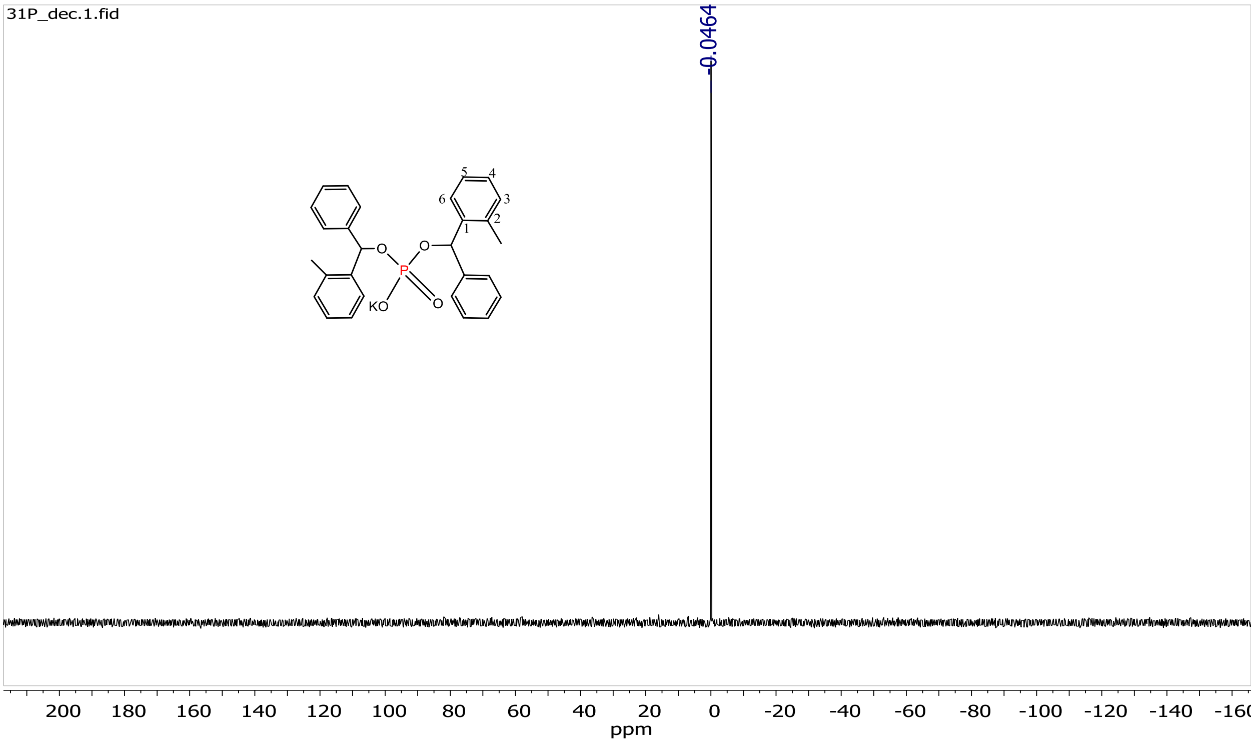

$^{31}P\{^1H\}$ NMR spectrum of **2b** (DMSO_d_6).

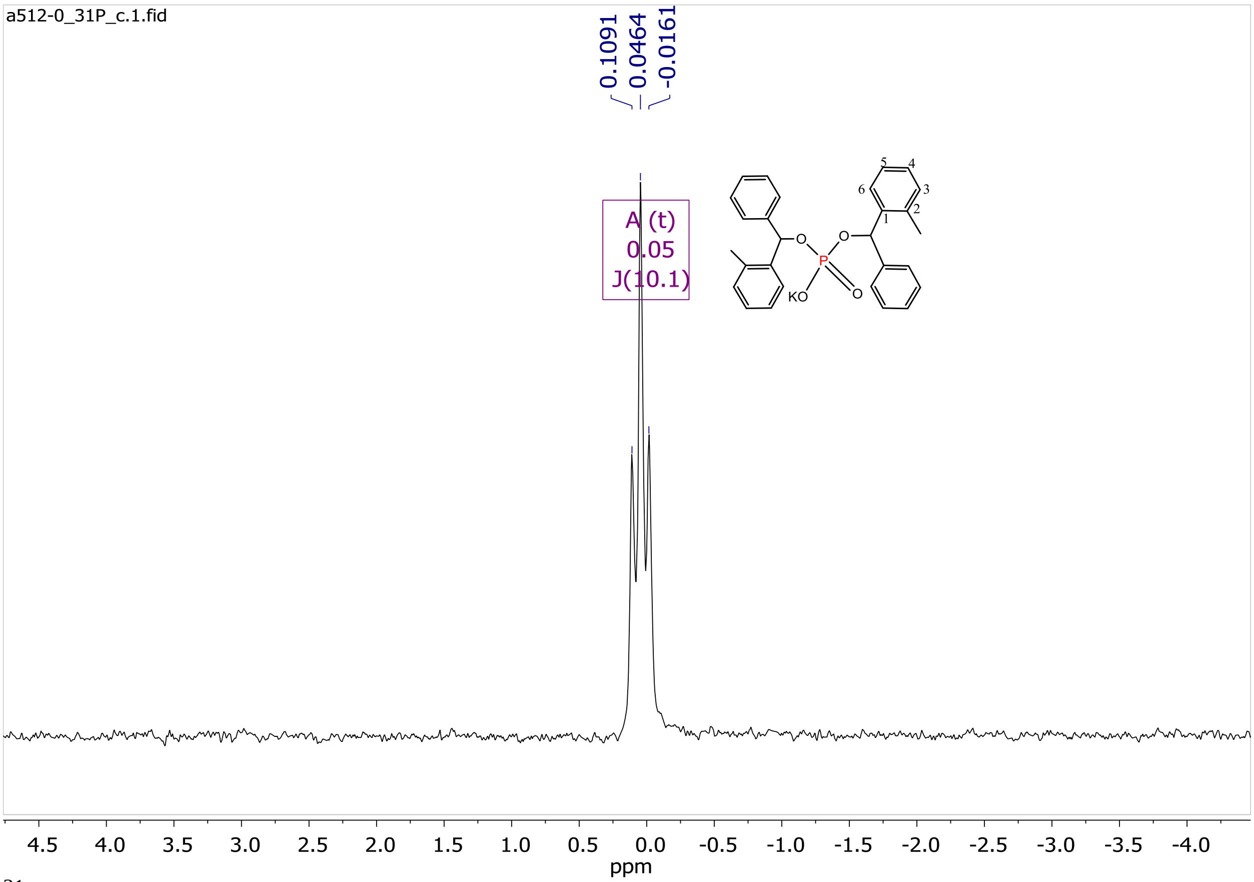

^{31}P NMR spectrum of **2b** (DMSO_d_6).

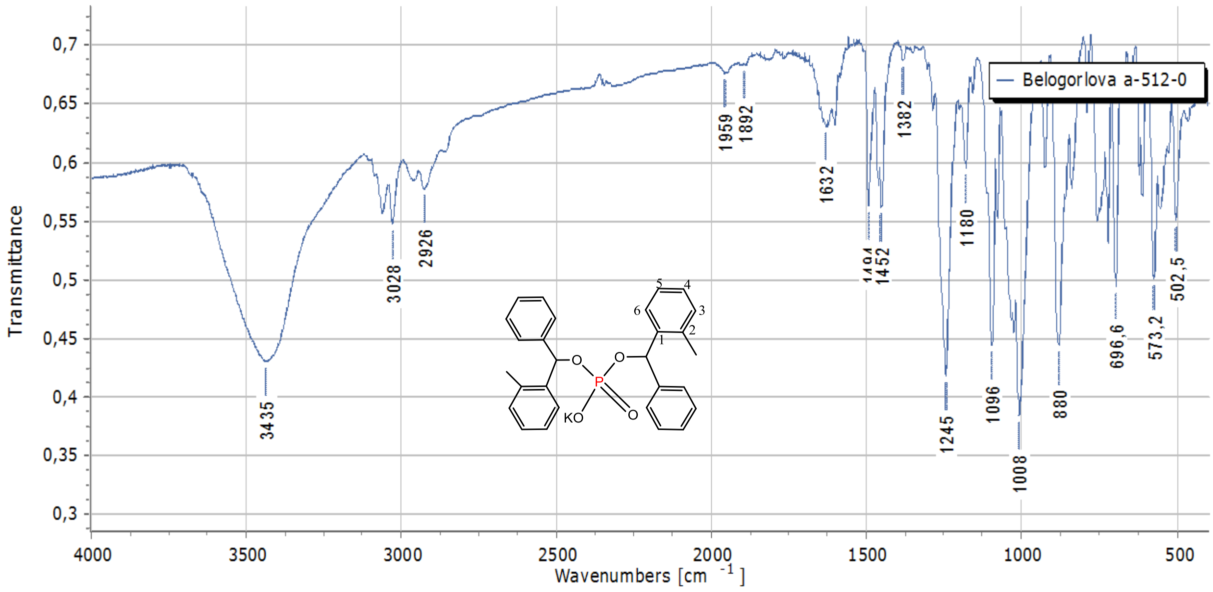

IR spectrum of **2b** (KBr, cm^{-1}).

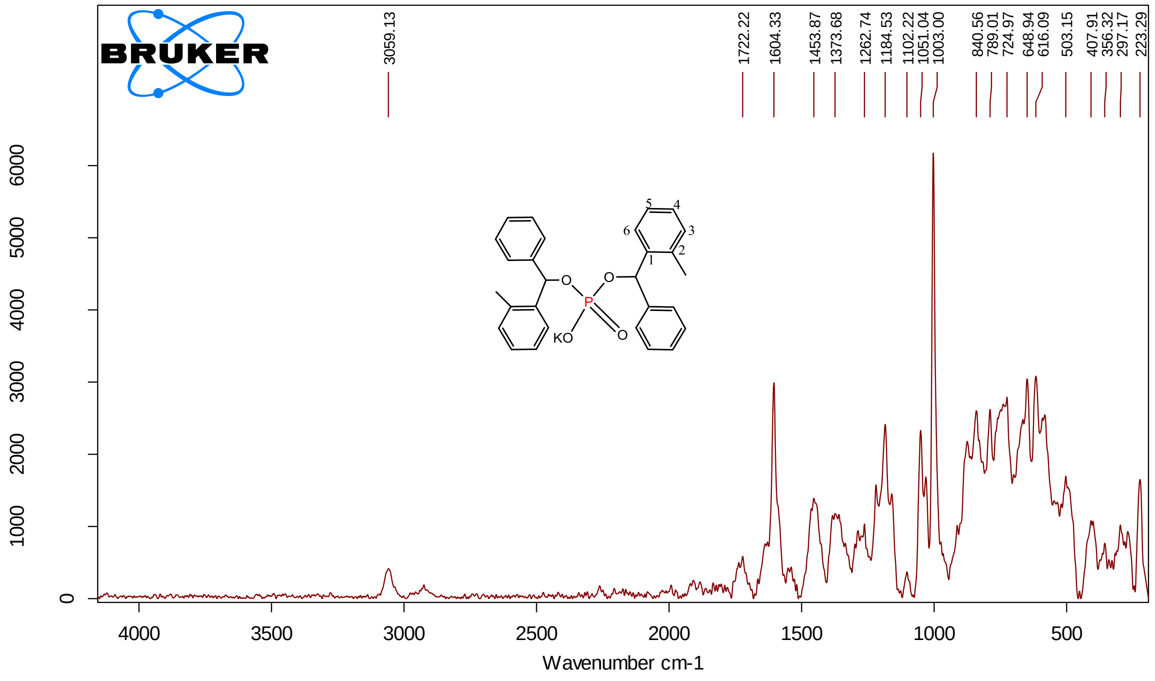

C:\Users\nikolaip\Desktop\IIH_ИИХ Фаворского\a512-0_785nm_2.txt

Page 1 of 1

Raman spectrum of **2b** (785 nm).

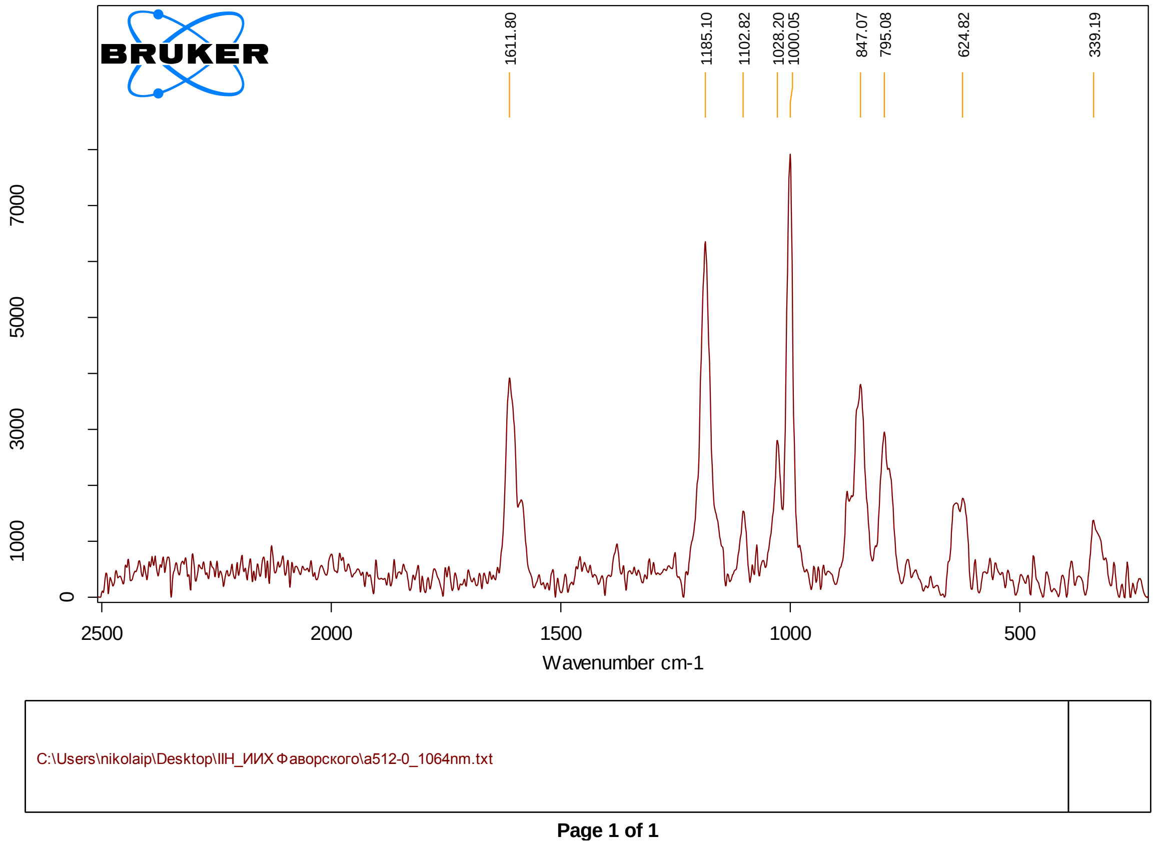

Raman spectrum of **2b** (1064 nm).

Potassium bis[(3-methylphenyl)(phenyl)methyl]phosphate (2c).

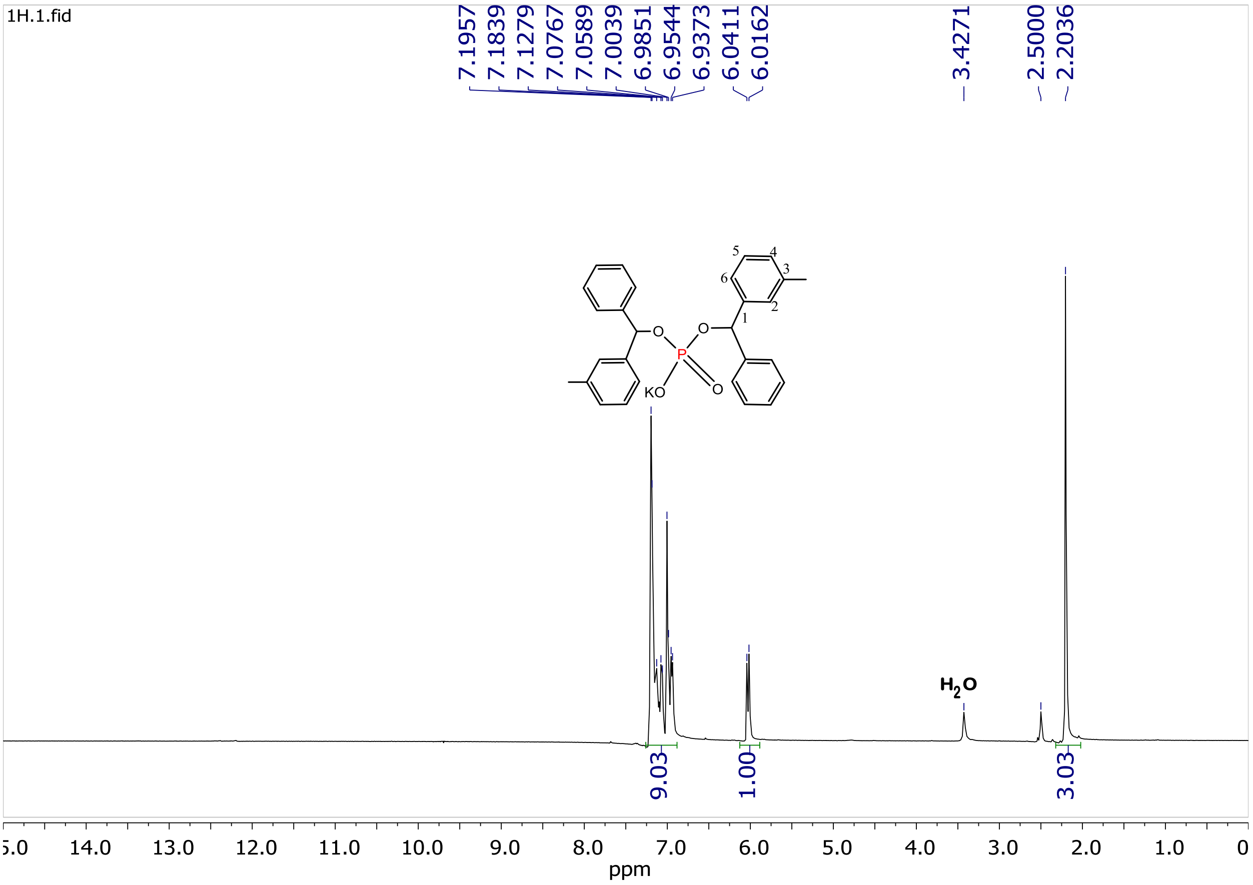

^{1}H NMR spectrum of **2c** (DMSO_d_6).

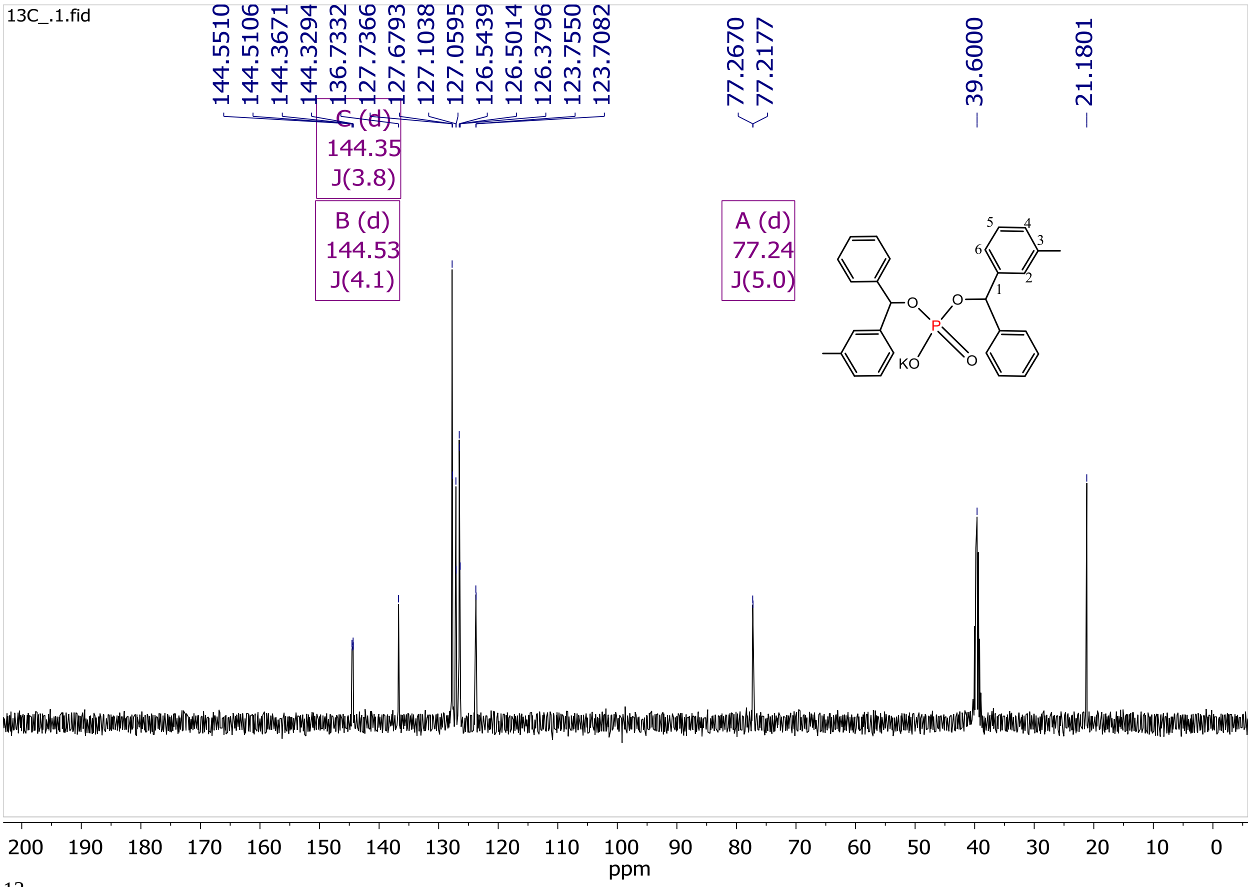

^{13}C NMR spectrum of **2c** (DMSO_d$_6$).

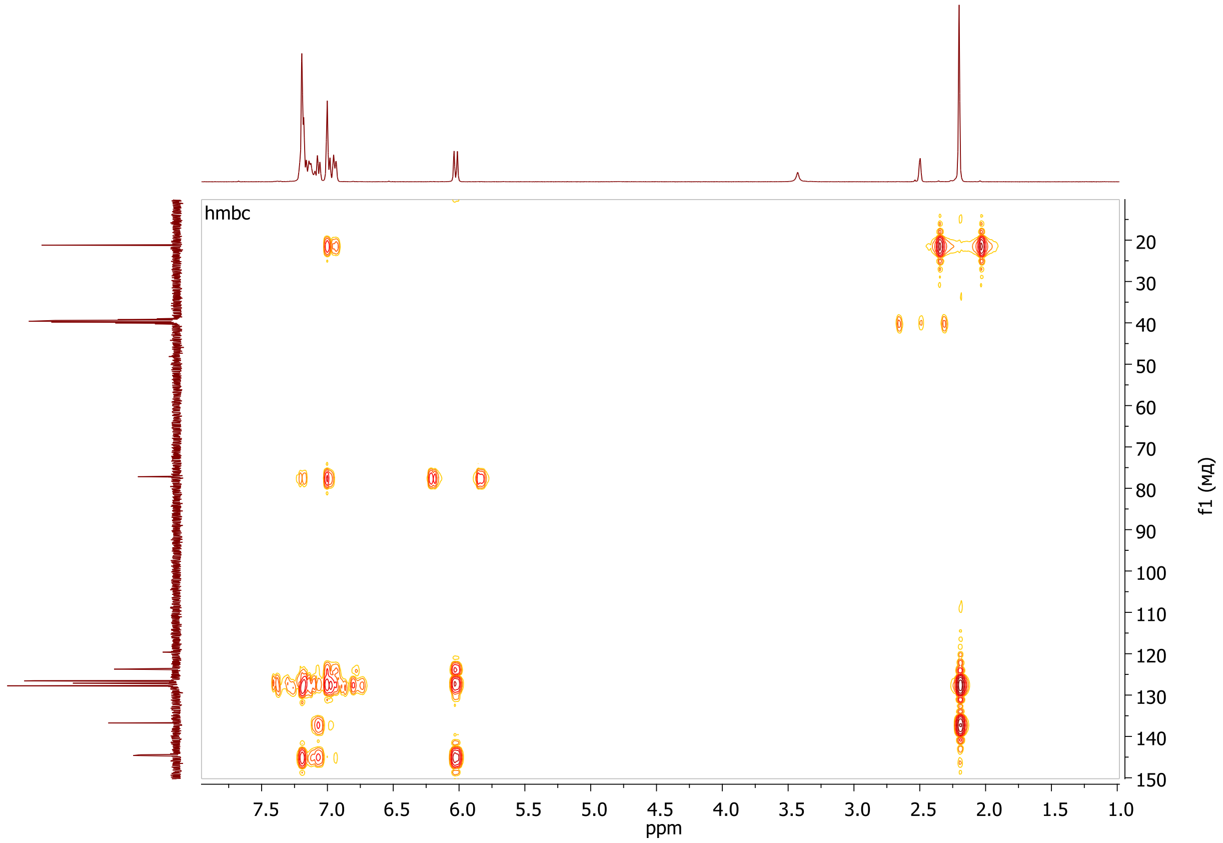

2D HMBC ^{13}C-^{1}H NMR spectrum of **2c** (DMSO_d$_6$).

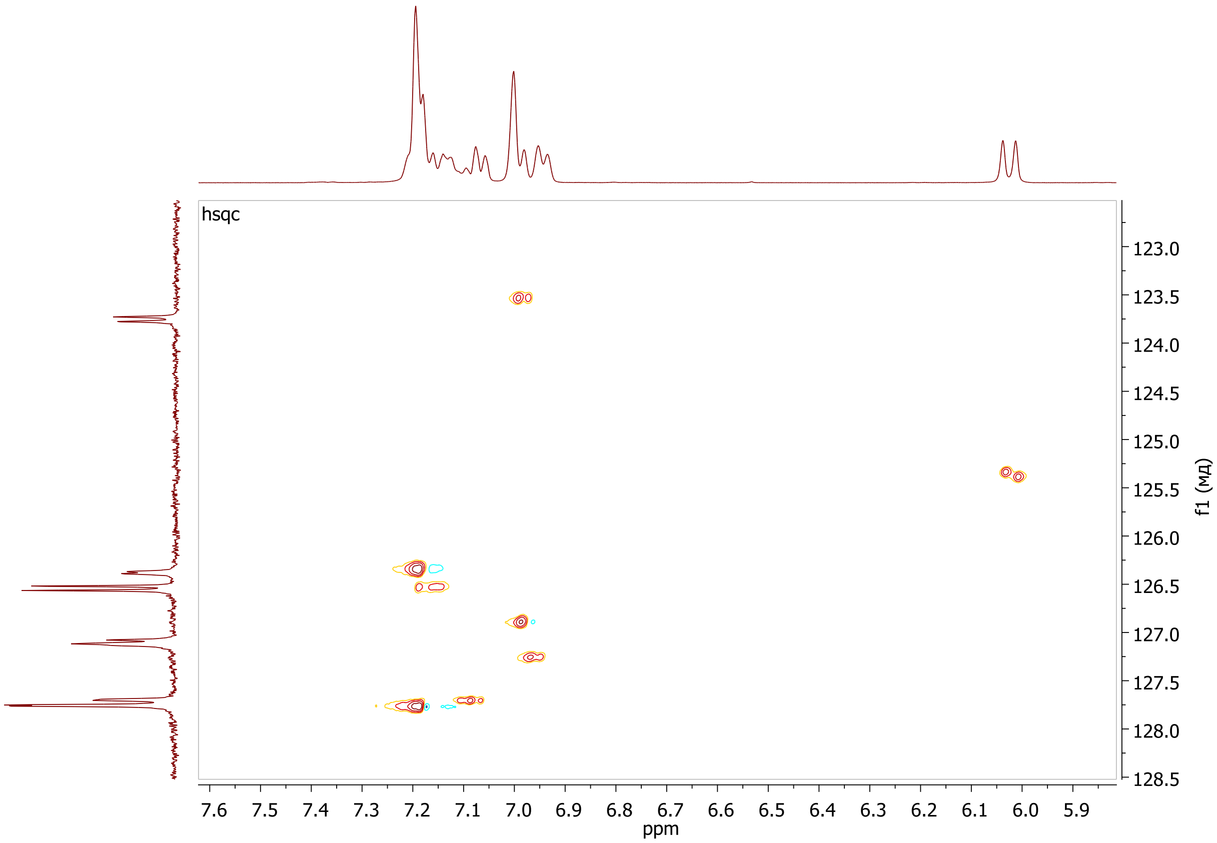

2D HSQC ^{13}C-^{1}H NMR spectrum of **2c** (DMSO_d_6).

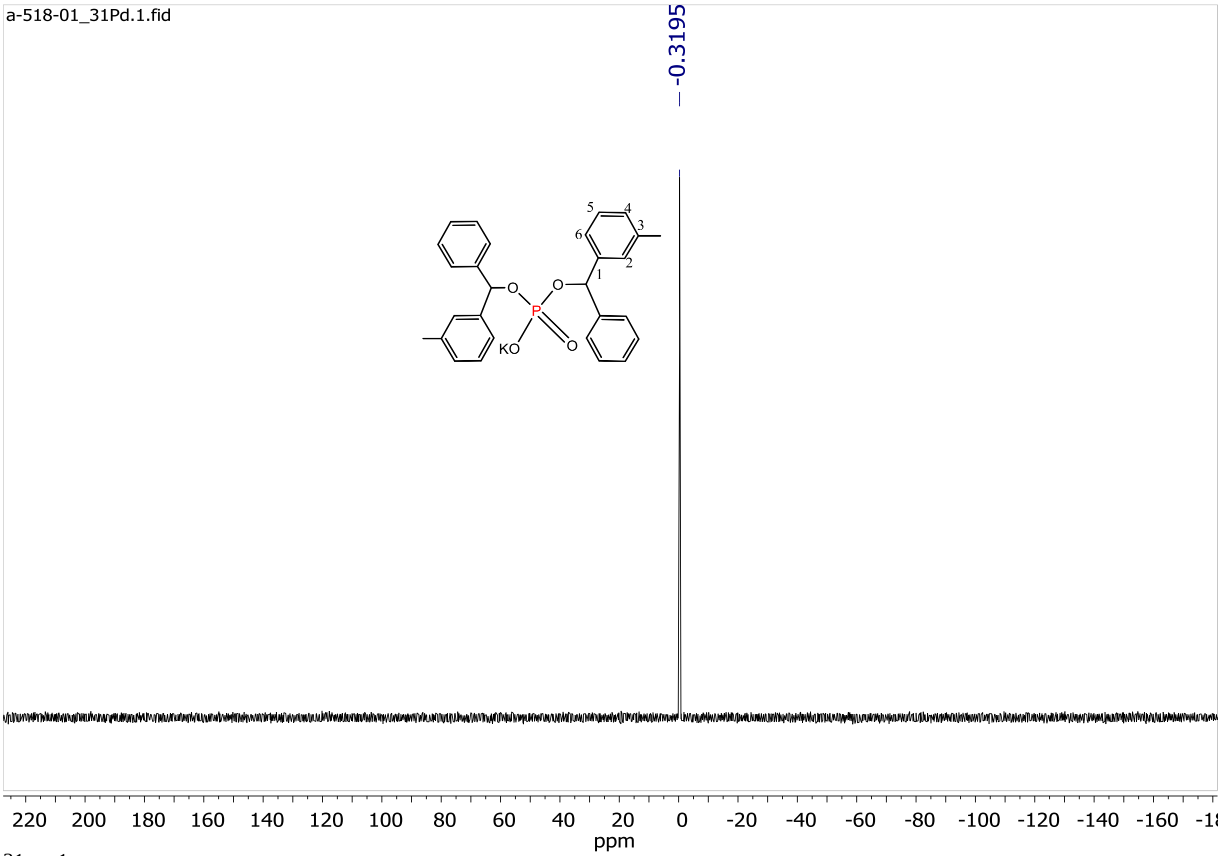

$^{31}P\{^{1}H\}$ NMR spectrum of **2c** (DMSO_d_6).

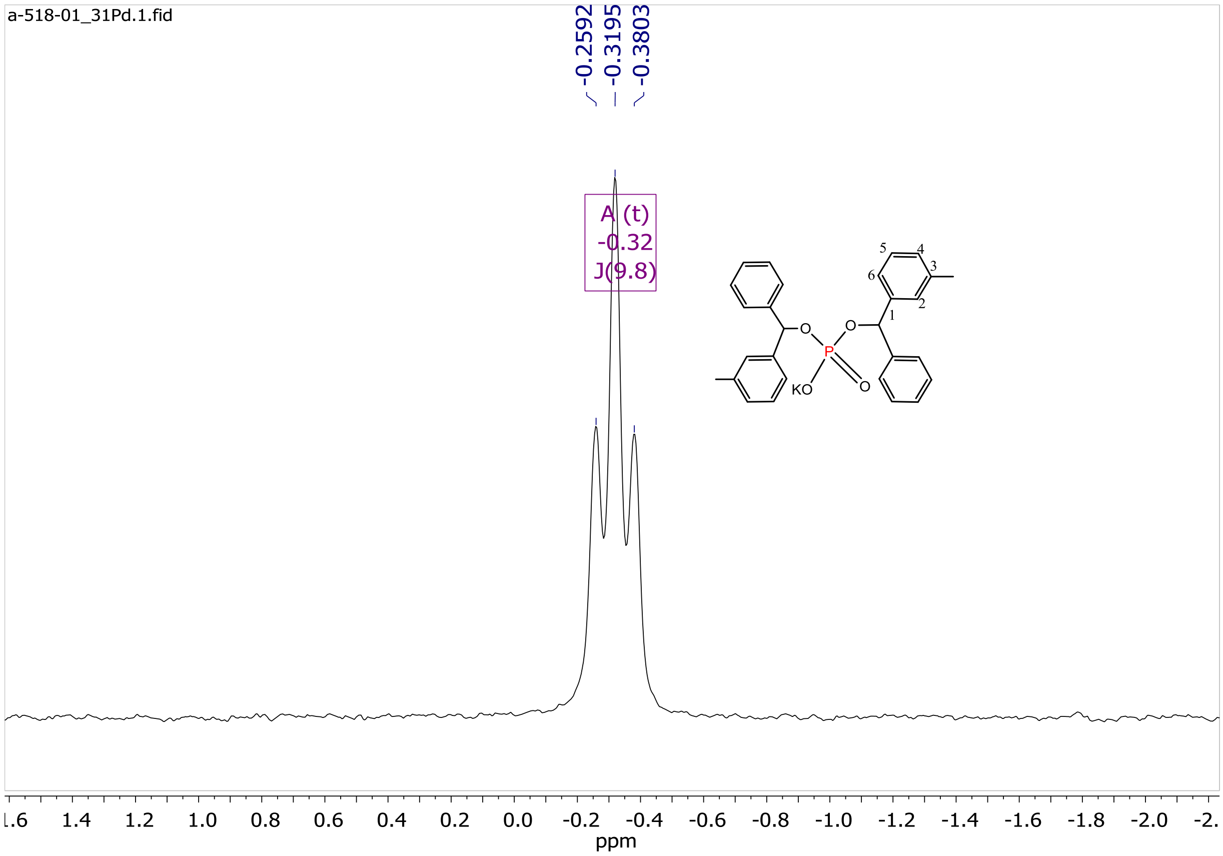

^{31}P NMR spectrum of **2c** (DMSO_d$_6$).

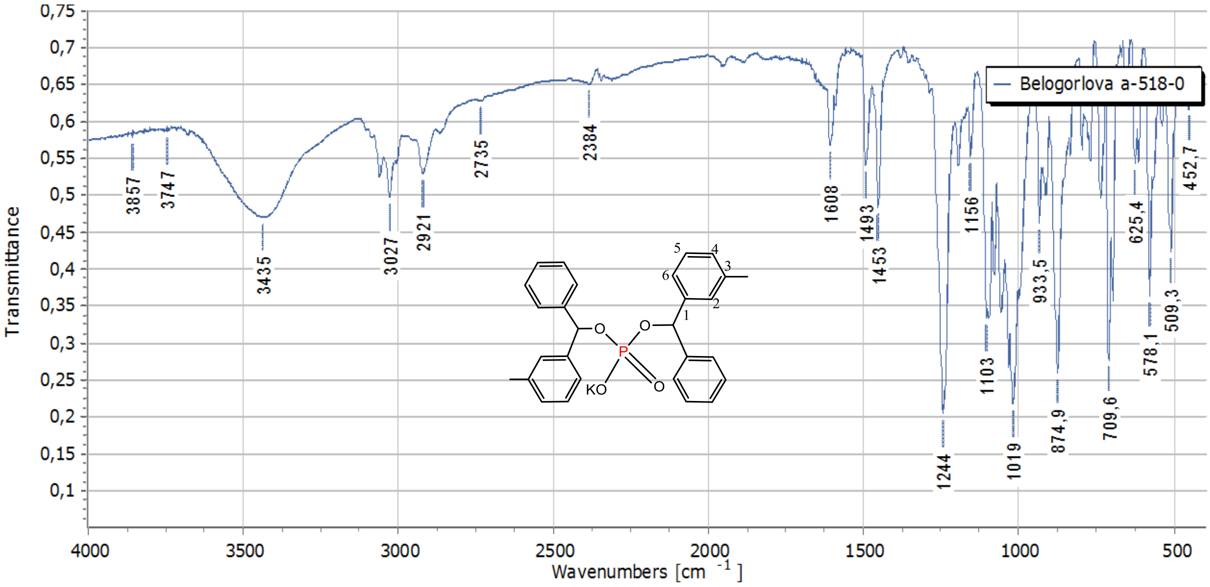

IR spectrum of **2c** (KBr, cm^{-1}).

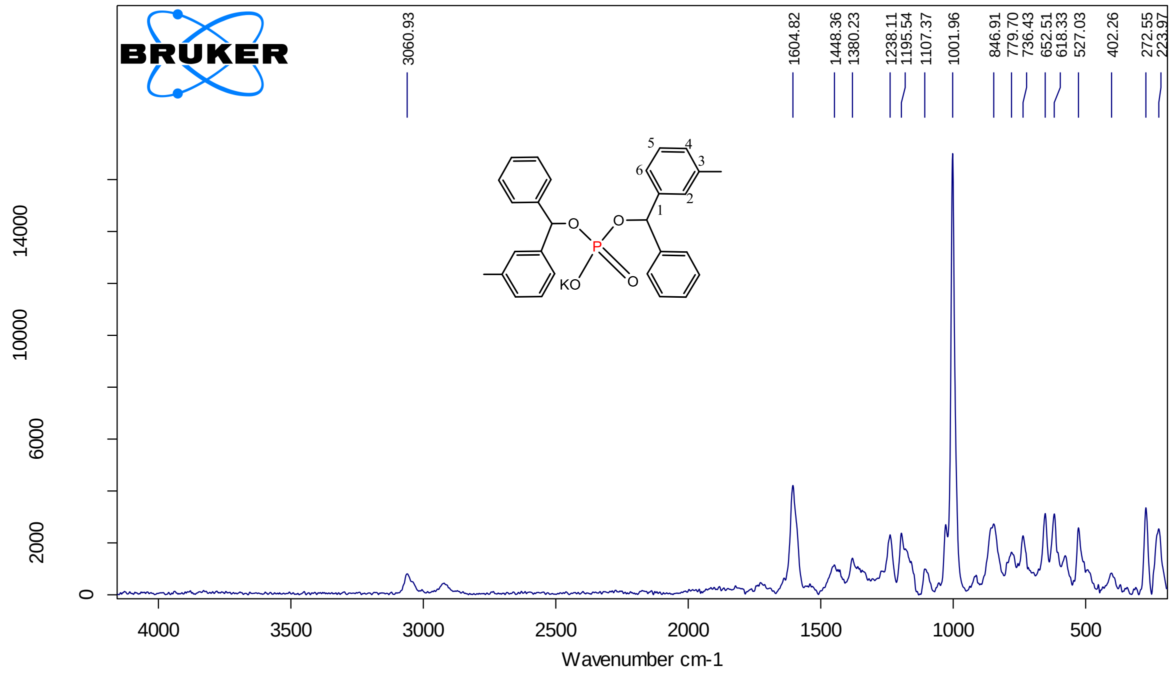

C:\Users\nikolaip\Desktop\IIH_ИИХ Фаворского\a518-0_785nm_1.txt

Page 1 of 1

Raman spectrum of **2c** (785 nm).

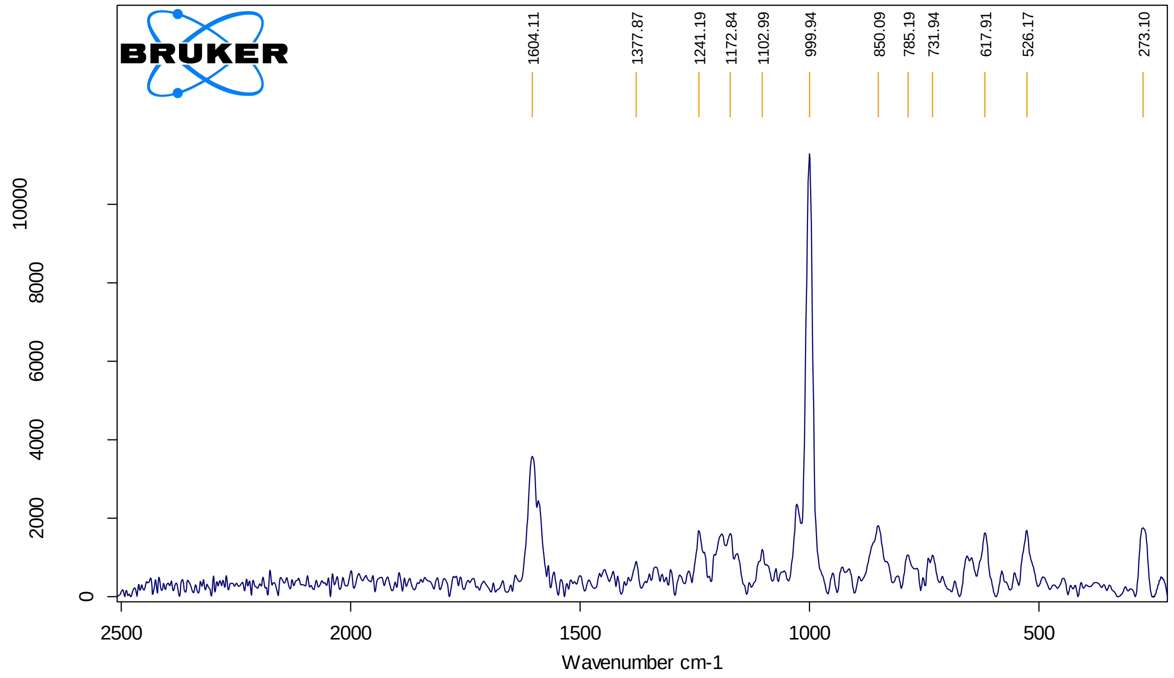

C:\Users\nikolaip\Desktop\IIH_ИИХ Фаворского\a518-0_1064nm.txt

Page 1 of 1

Raman spectrum of **2c** (1064 nm).

Potassium bis[(4-methylphenyl)(phenyl)methyl]phosphate (2d).

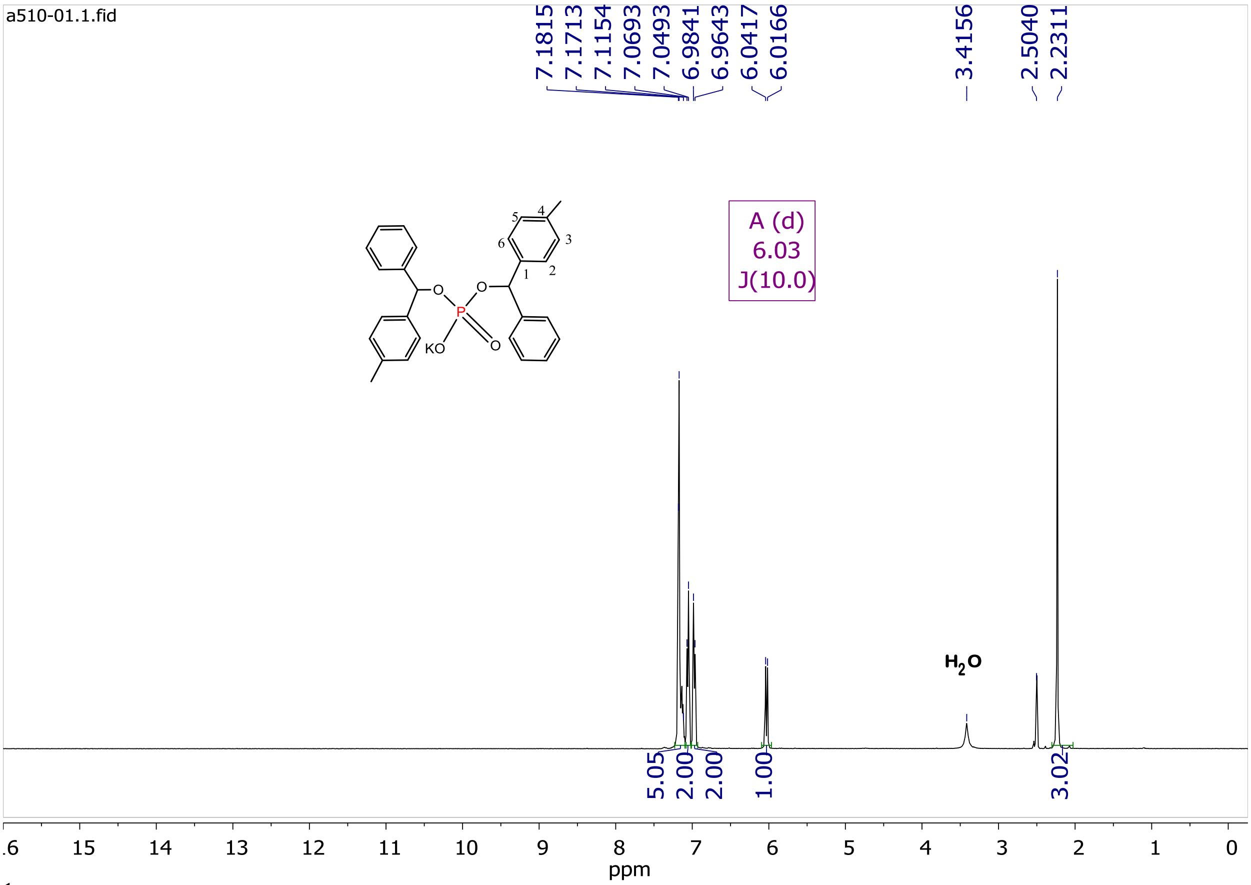

^{1}H NMR spectrum of **2d** (DMSO_d$_6$).

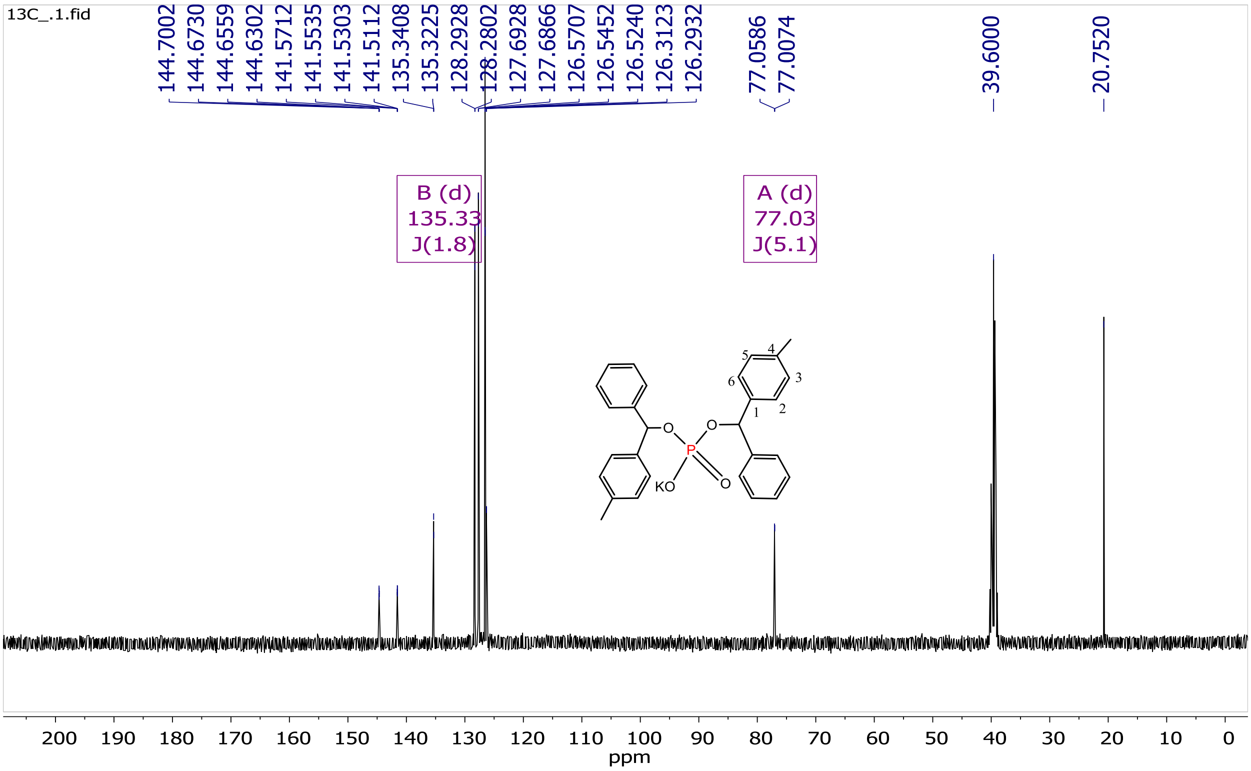

^{13}C NMR spectrum of **2d** (DMSO_d$_6$).

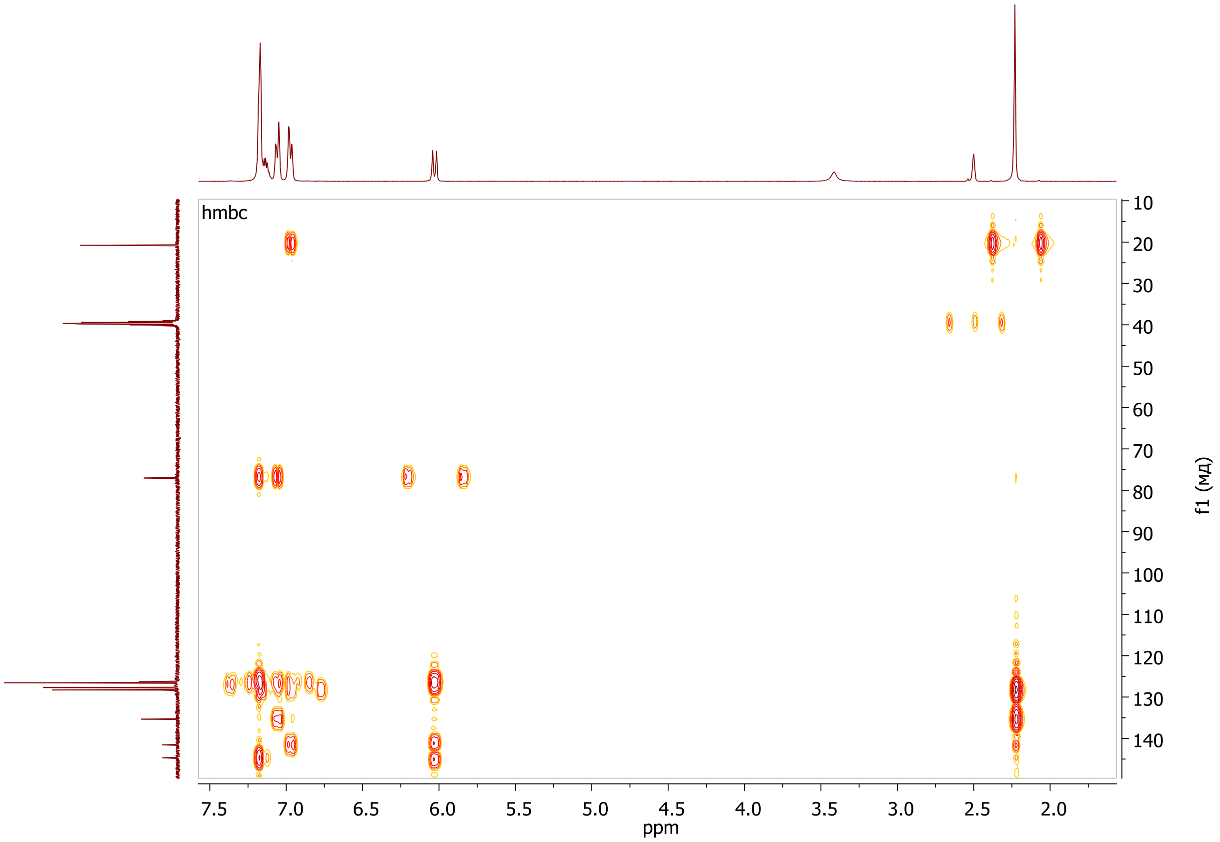

2D HMBC ^{13}C-^{1}H NMR spectrum of **2d** (DMSO_d$_6$).

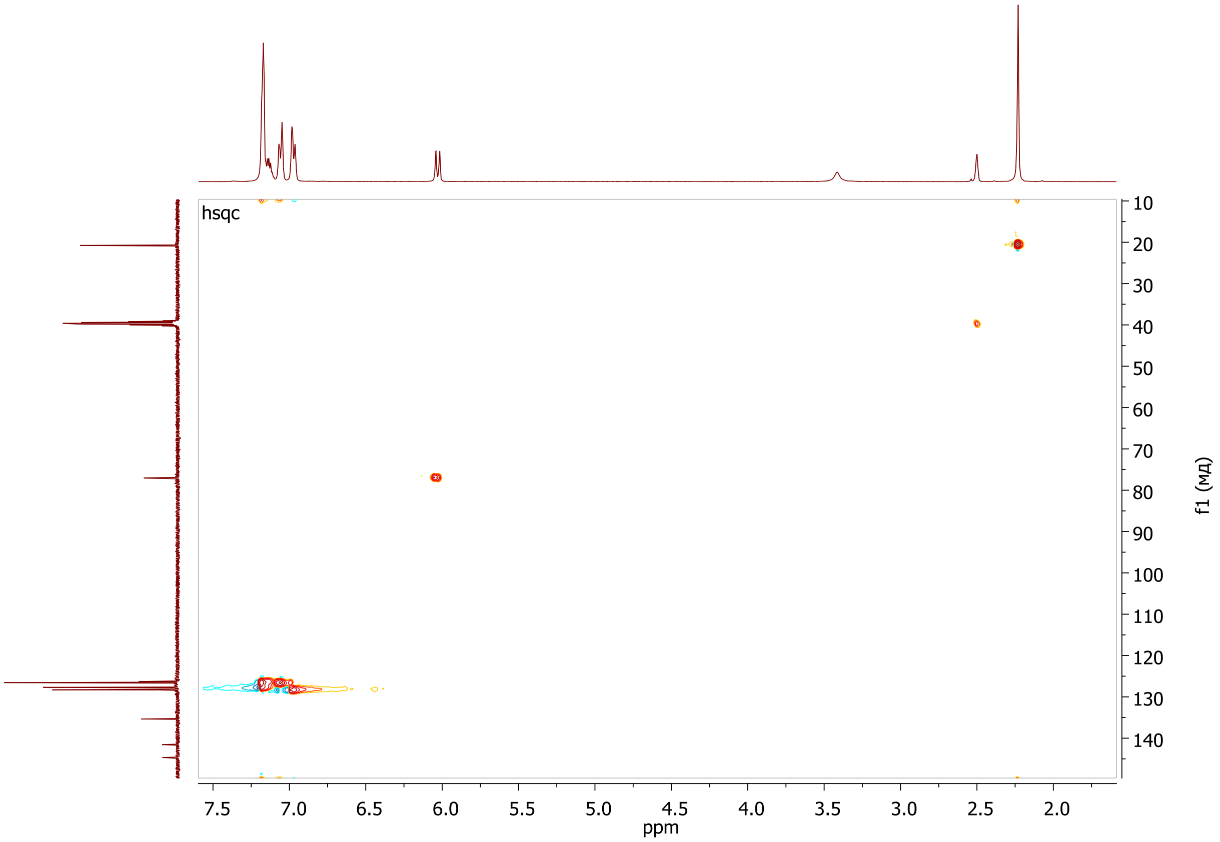

2D HSQC ^{13}C-^{1}H NMR spectrum of **2d** (DMSO_d$_6$).

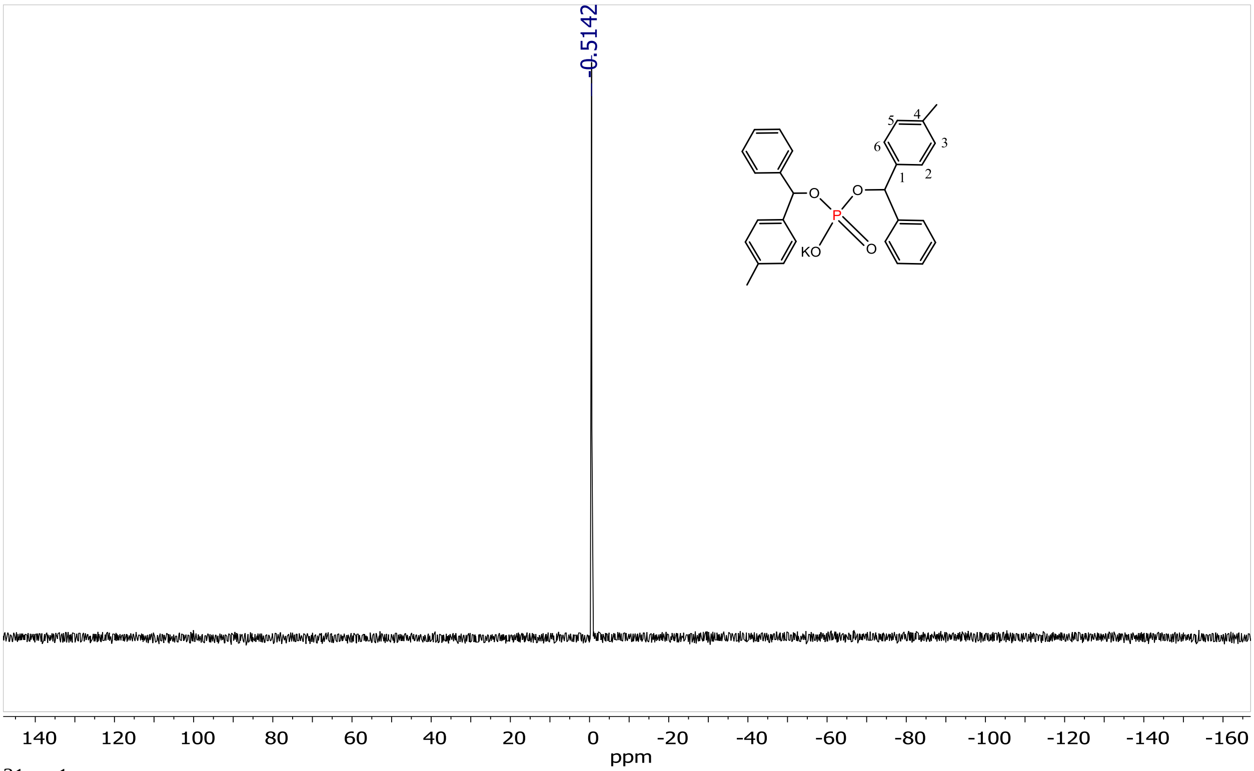

$^{31}P\{^{1}H\}$ NMR spectrum of **2d** (DMSO_d_6).

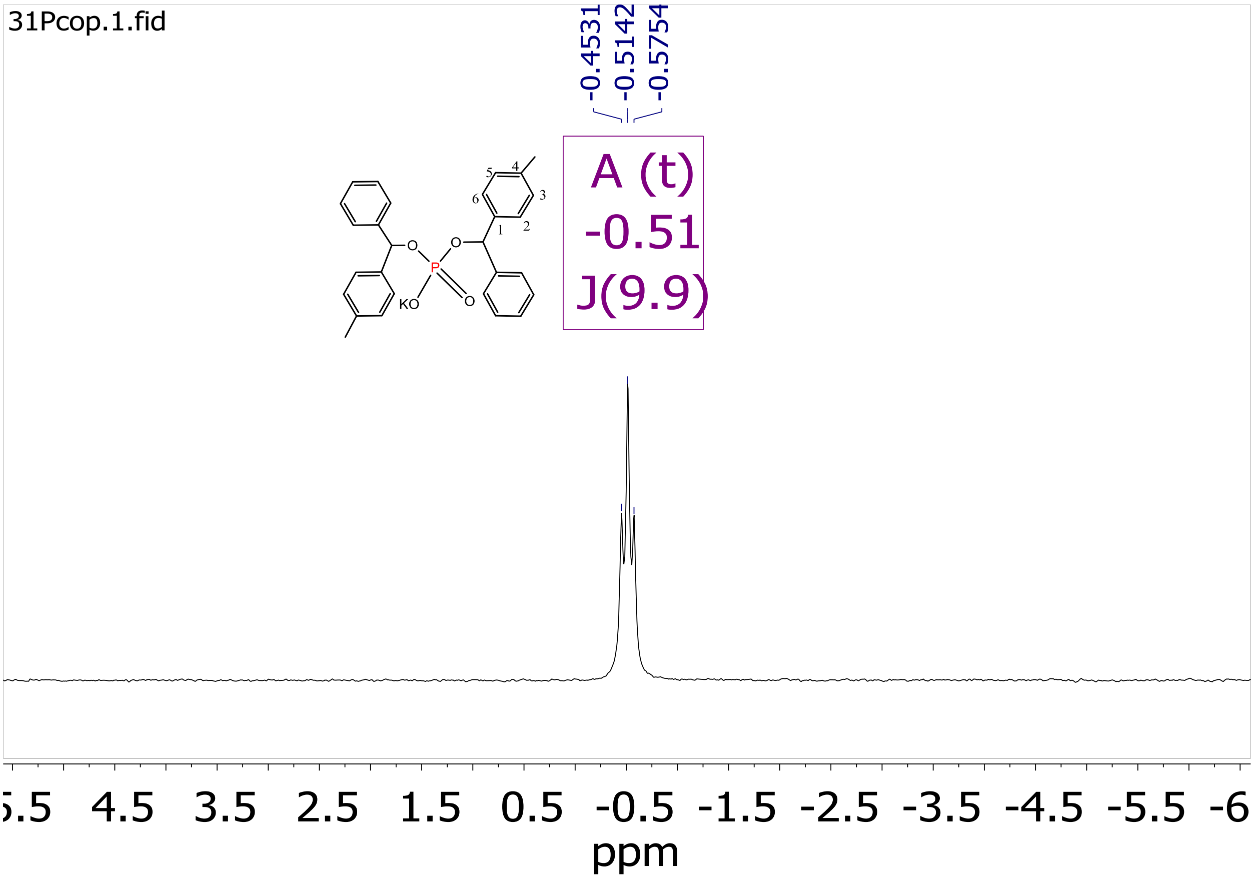

^{31}P NMR spectrum of **2d** (DMSO_d_6).

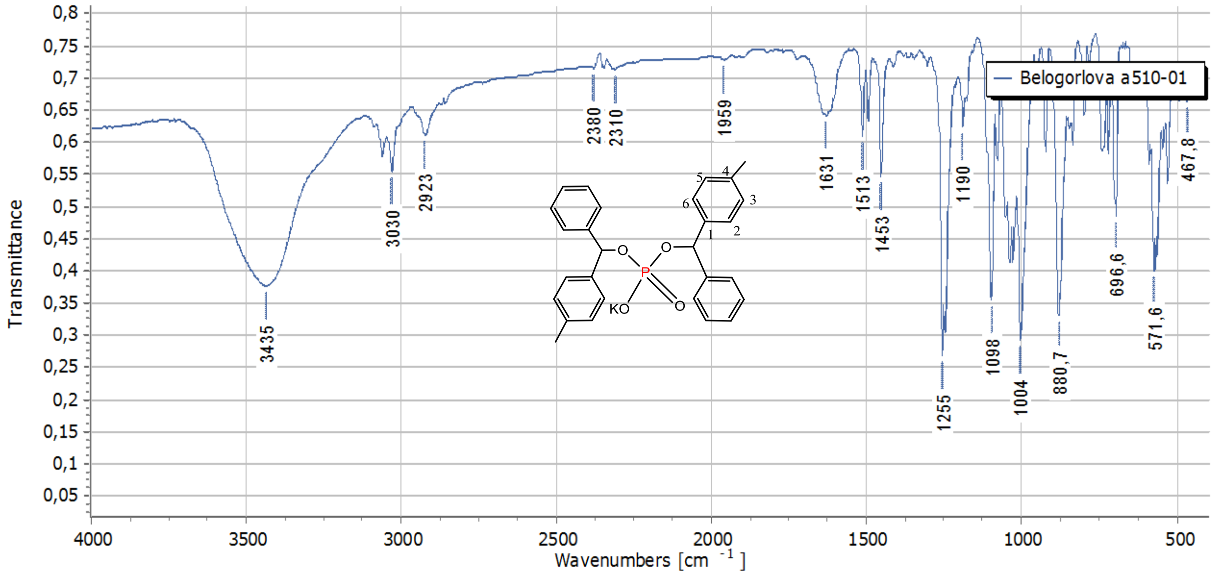

IR spectrum of **2d** (KBr, cm^{-1}).

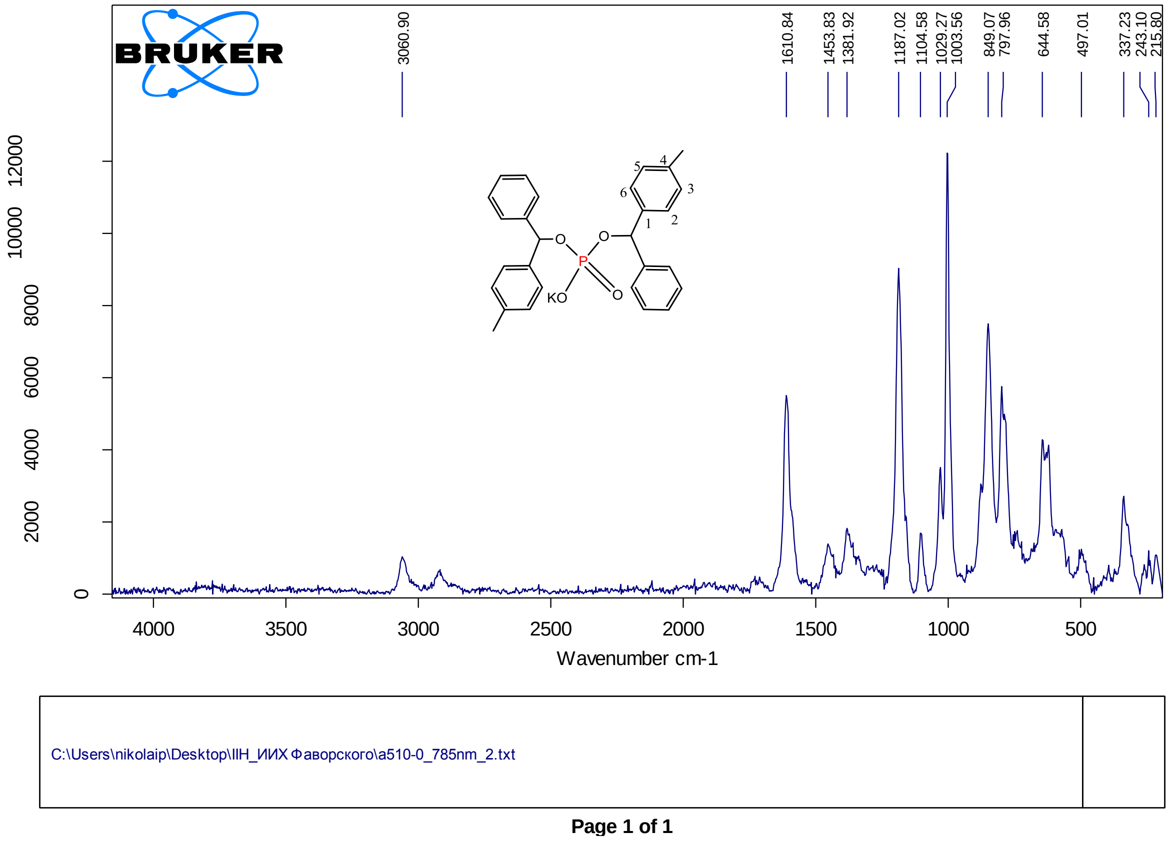

Raman spectrum of **2d** (785 nm).

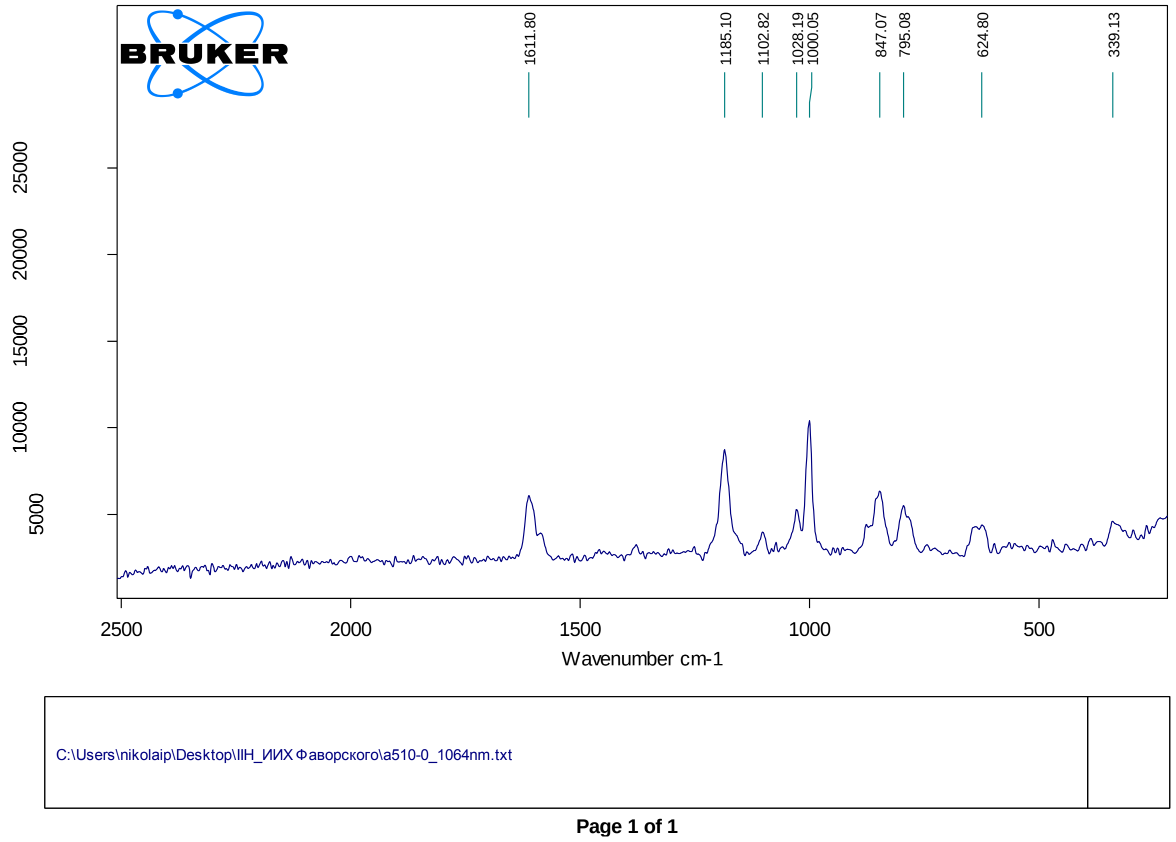

Raman spectrum of **2d** (1064 nm).

Potassium bis[(4-*tert*-butylphenyl)(phenyl)methyl]phosphate (2e).

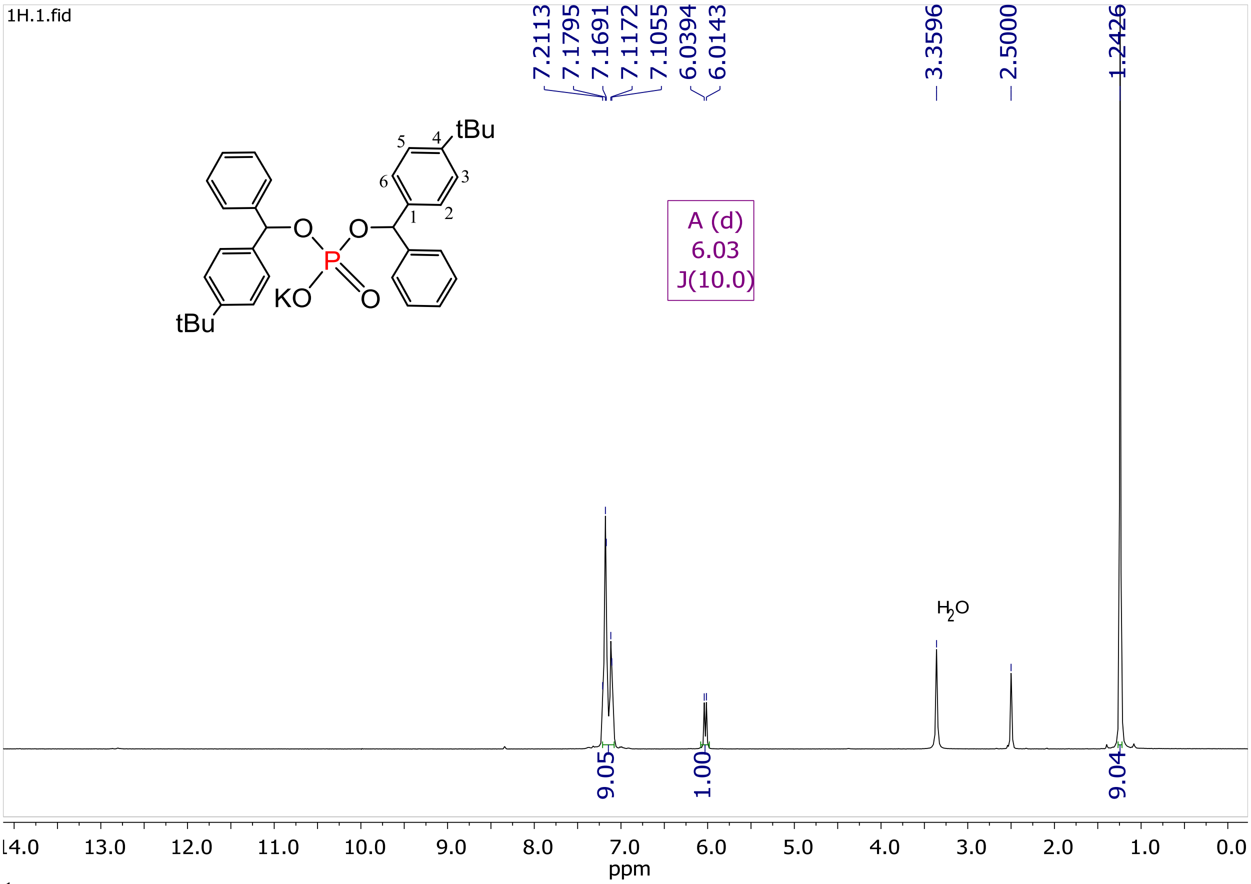

^{1}H NMR spectrum of **2e** (DMSO_d_6).

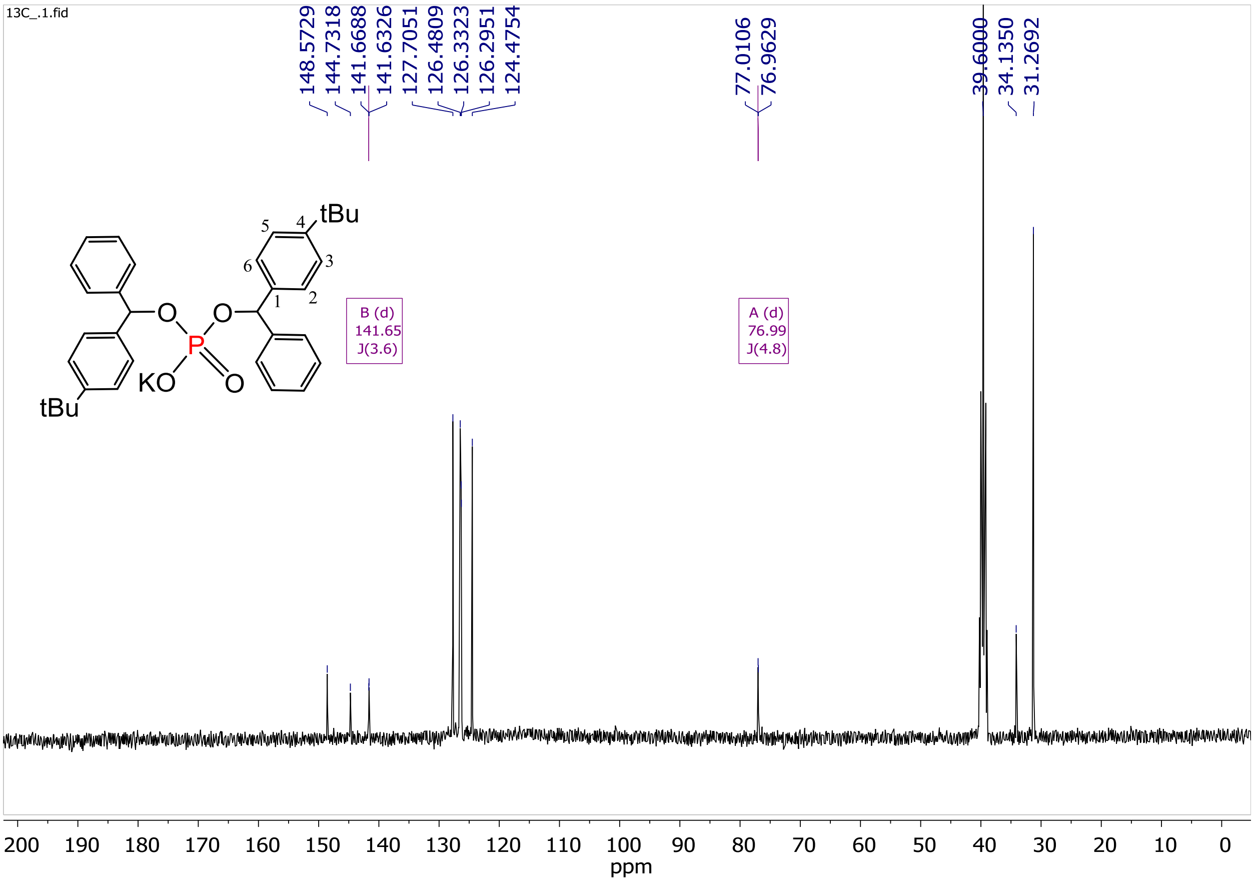

^{13}C NMR spectrum of **2e** (DMSO_d$_6$).

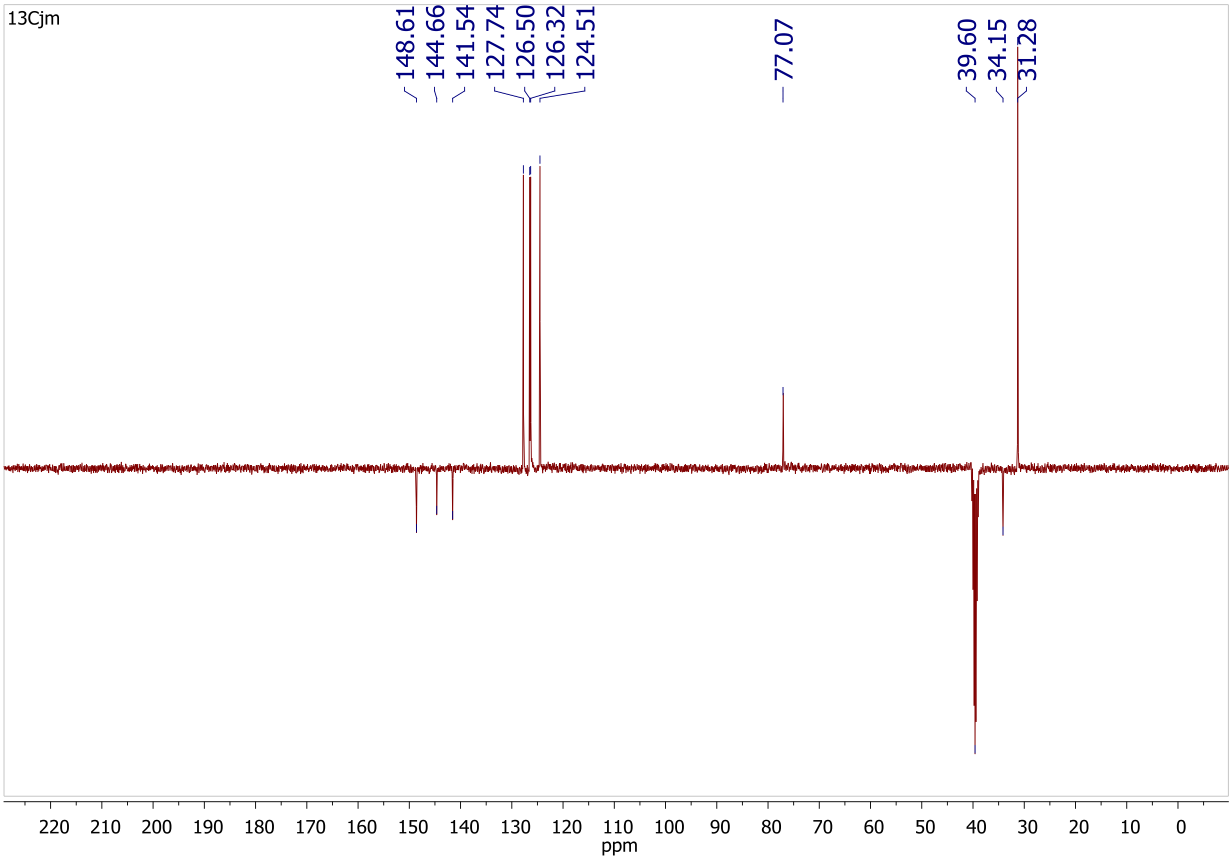

^{13}Cjm NMR spectrum of **2e** (DMSO_d$_6$).

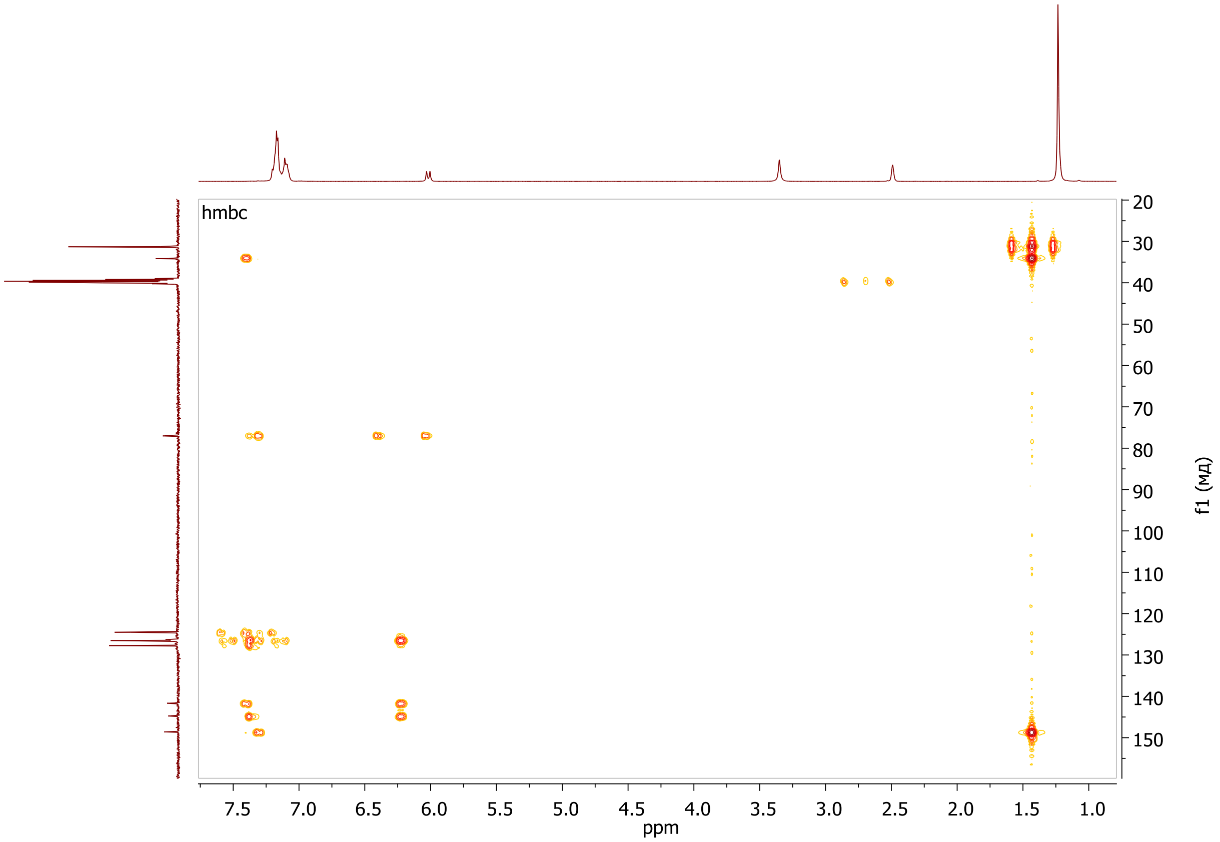

2D HMBC ^{13}C-^{1}H NMR spectrum of **2e** (DMSO_d_6).

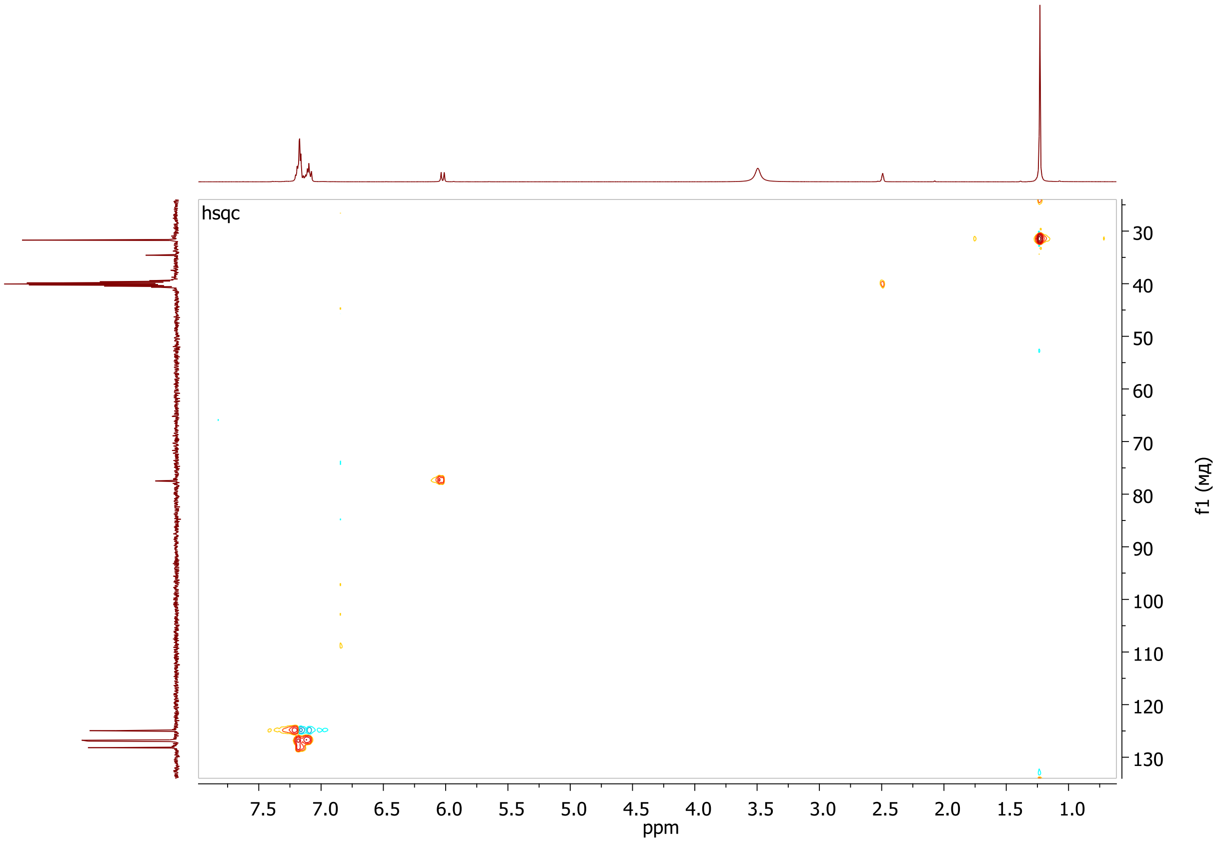

2D HSQC ^{13}C-^{1}H NMR spectrum of **2e** (DMSO_d_6).

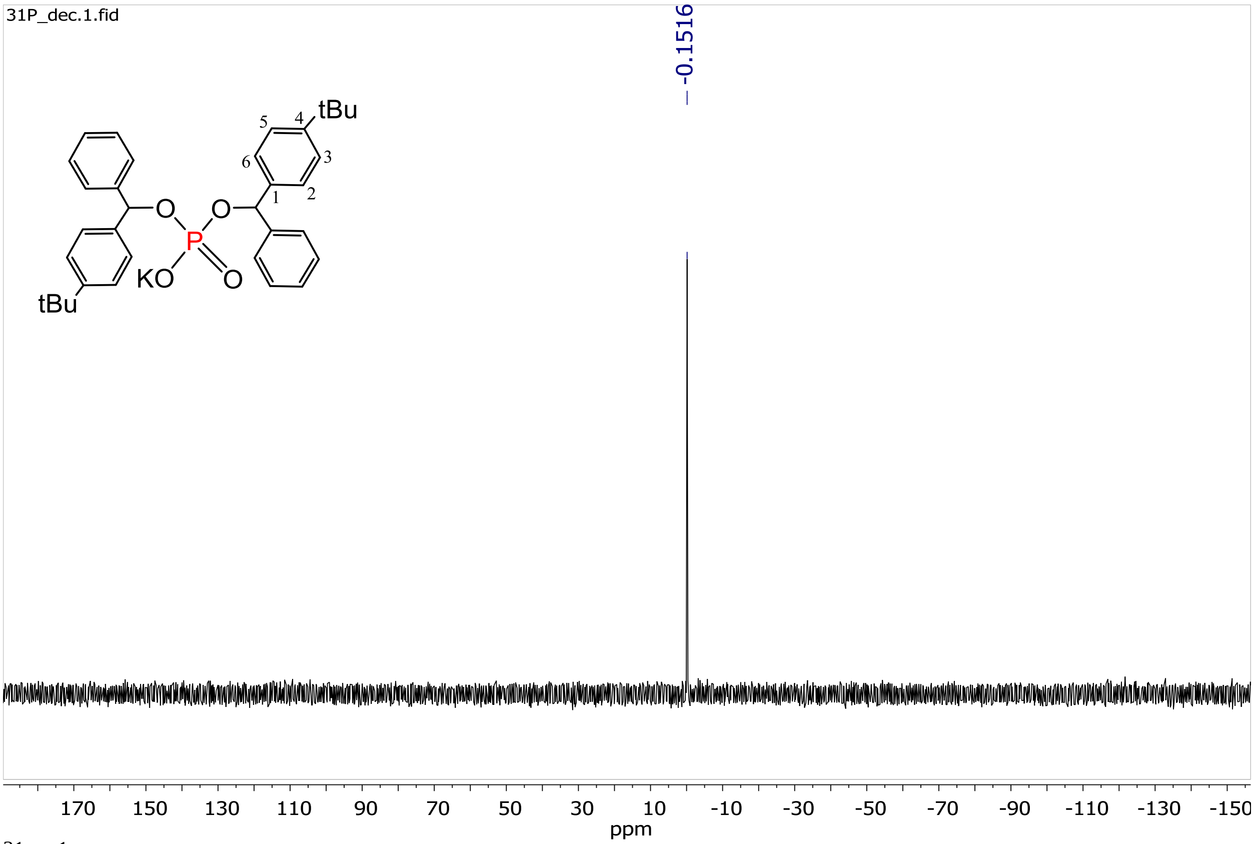

$^{31}P\{^1H\}$ NMR spectrum of **2e** (DMSO_d$_6$).

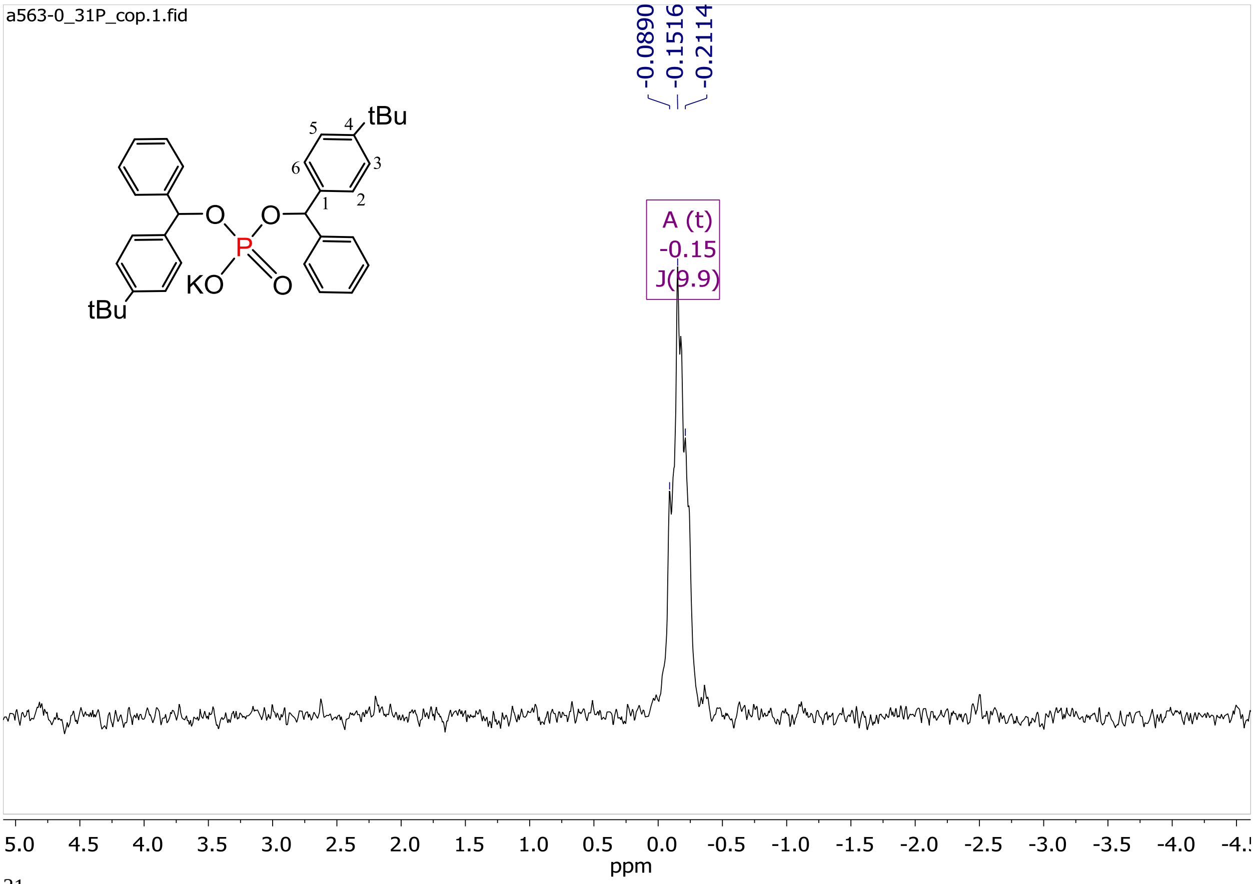

^{31}P NMR spectrum of **2e** (DMSO_d$_6$).

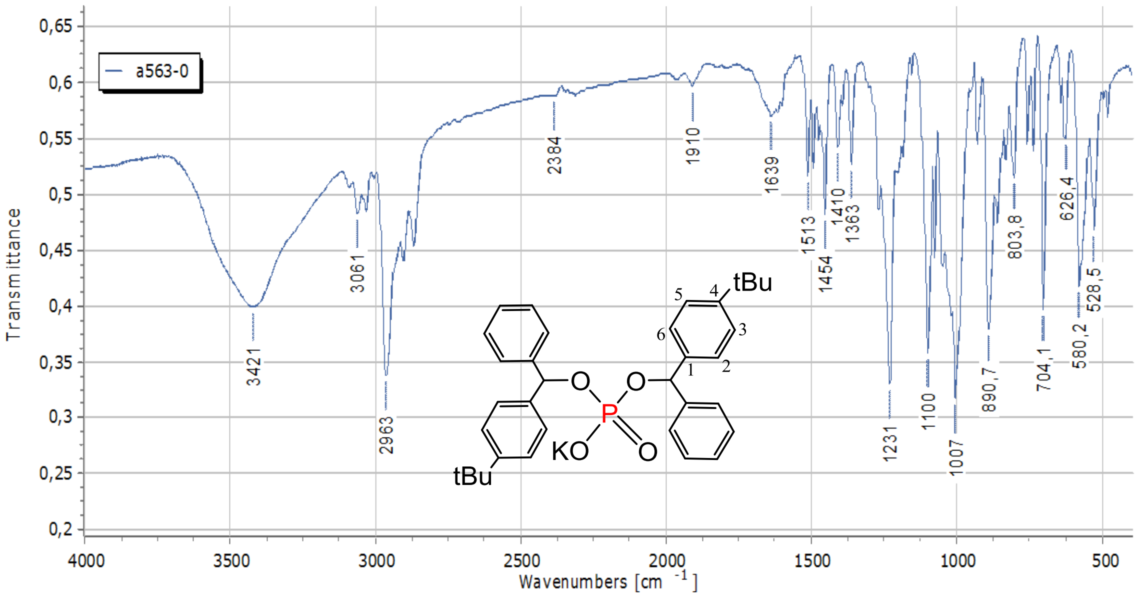

IR spectrum of **2e** (KBr, cm^{-1}).

Potassium bis[(4-methoxyphenyl)(phenyl)methyl]phosphate (2f).

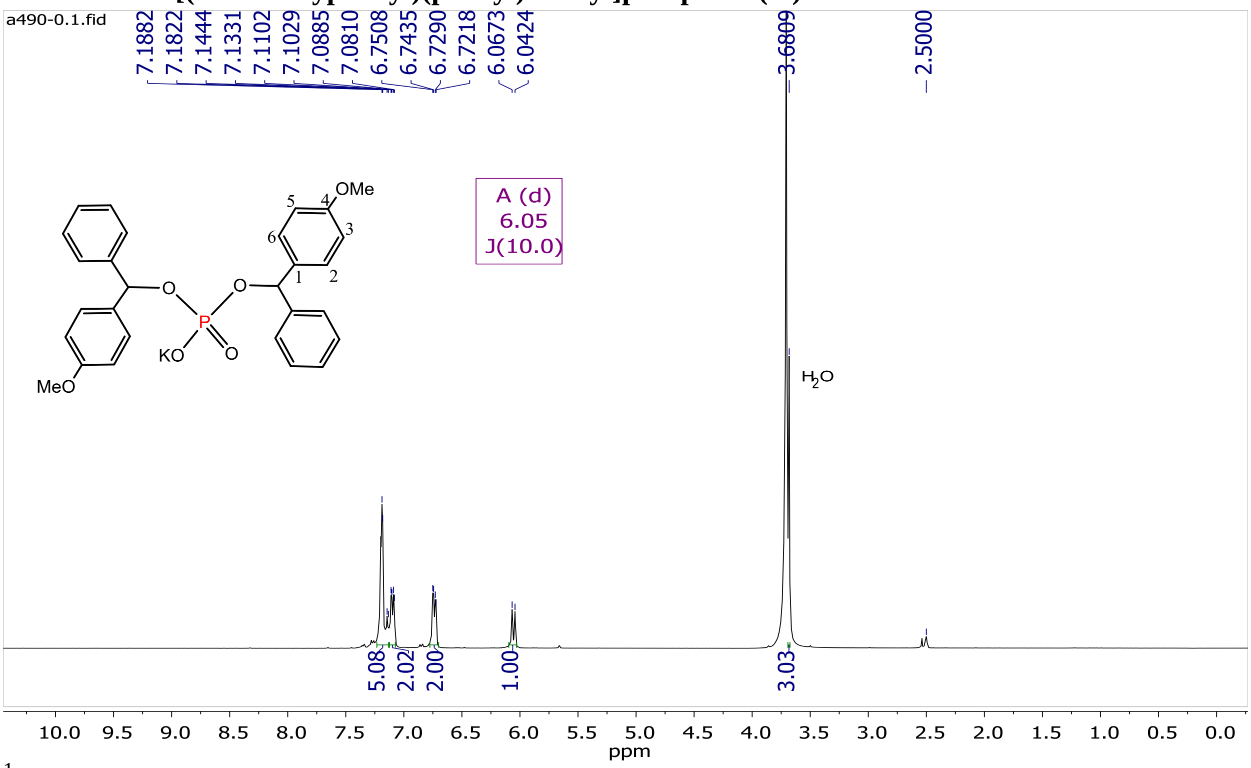

^{1}H NMR spectrum of **2f** (DMSO_d_6).

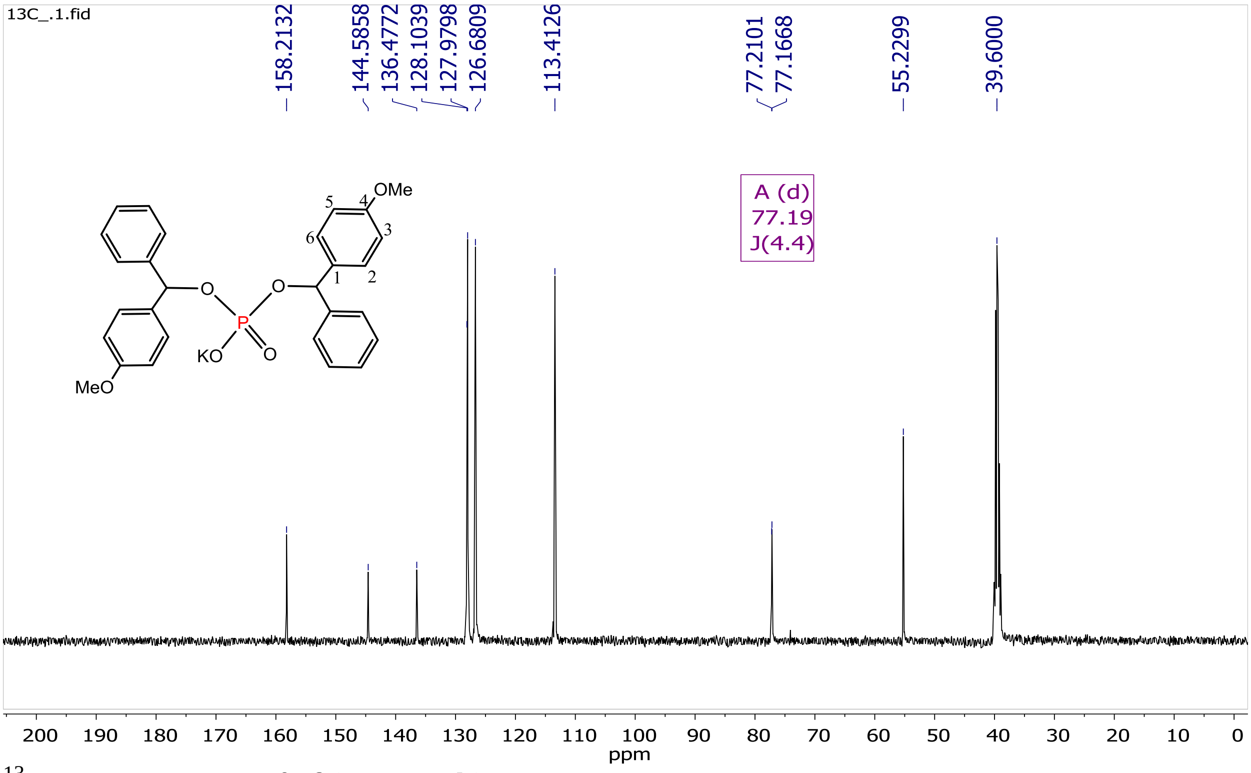

^{13}C NMR spectrum of **2f** (DMSO_d$_6$).

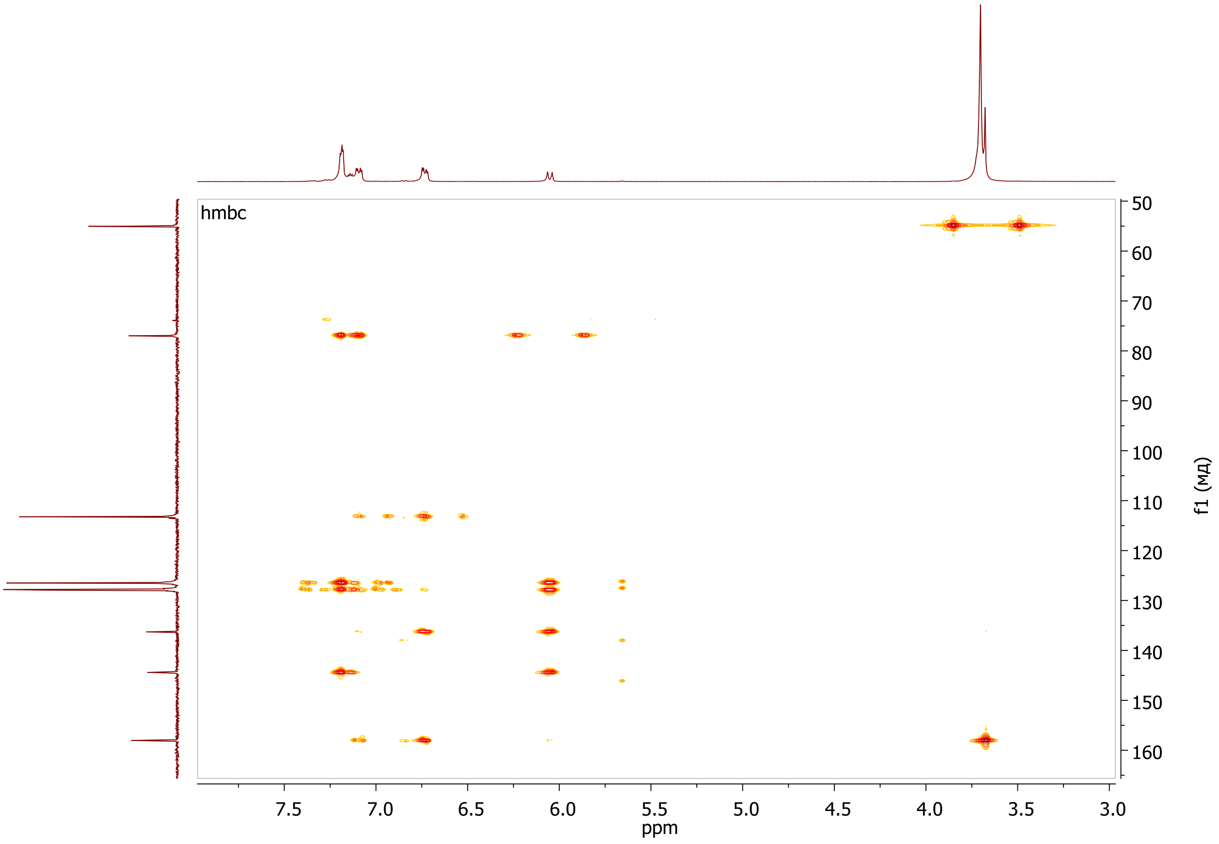

2D HMBC ^{13}C-^{1}H NMR spectrum of **2f** (DMSO_d$_6$).

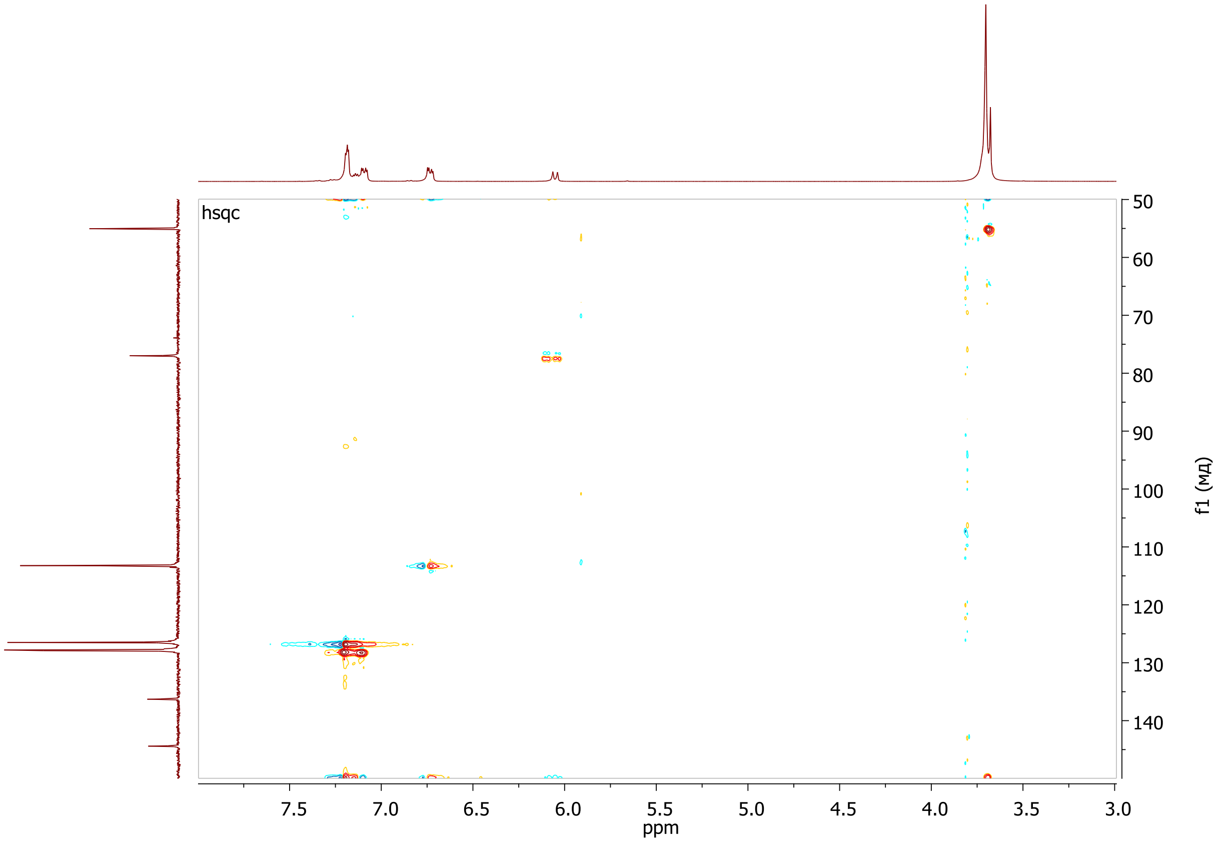

2D HSQC ^{13}C-^{1}H NMR spectrum of **2f** (DMSO_d_6).

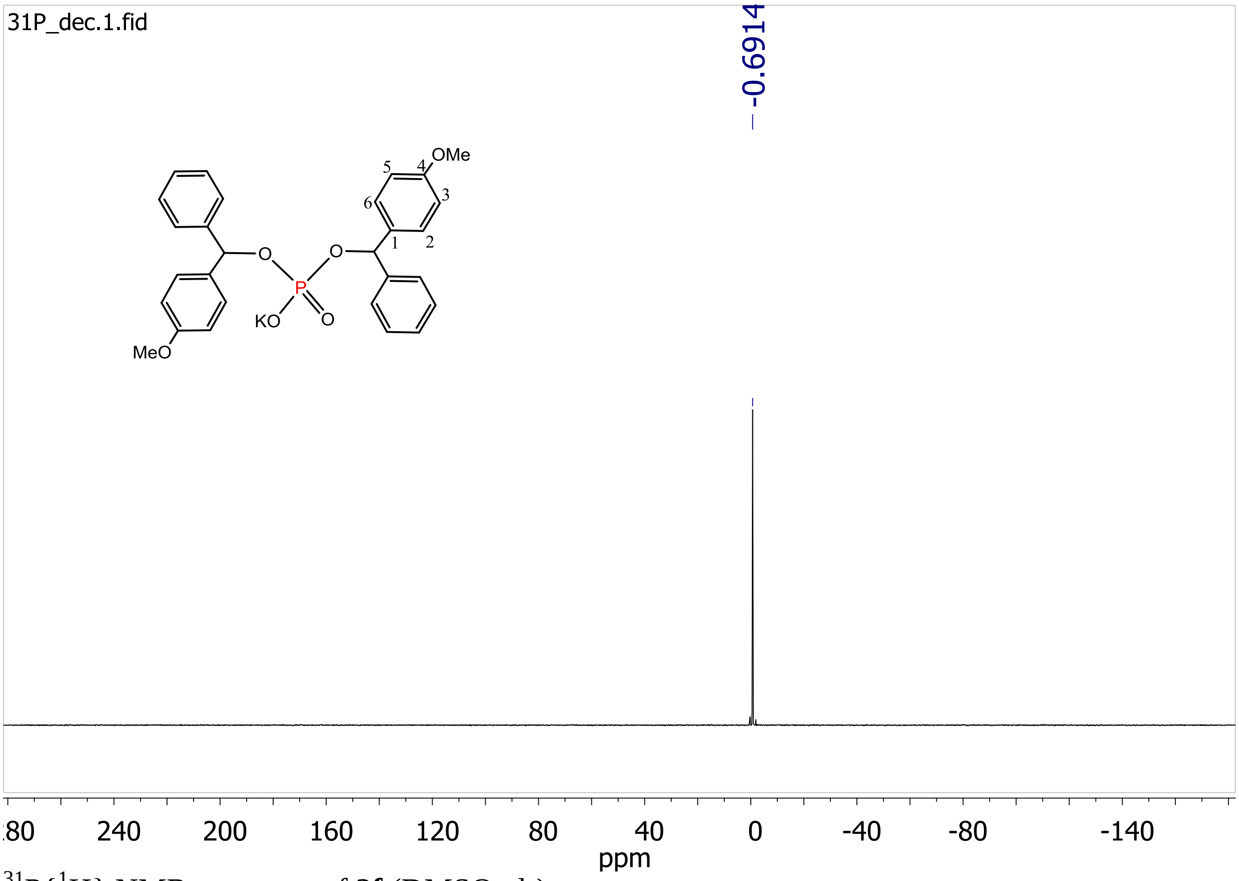

$^{31}P\{^{1}H\}$ NMR spectrum of **2f** (DMSO_d_6).

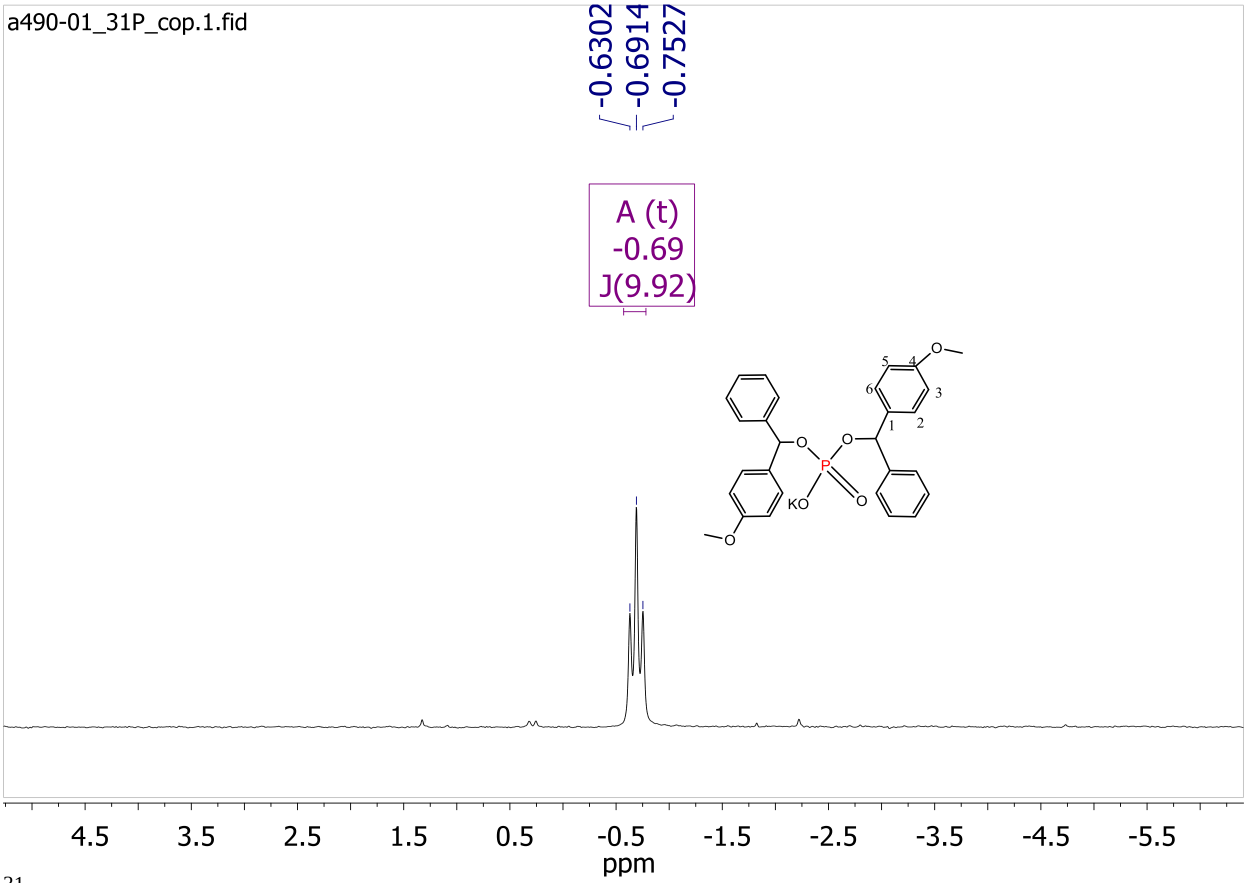

^{31}P NMR spectrum of **2f** (DMSO_d$_6$).

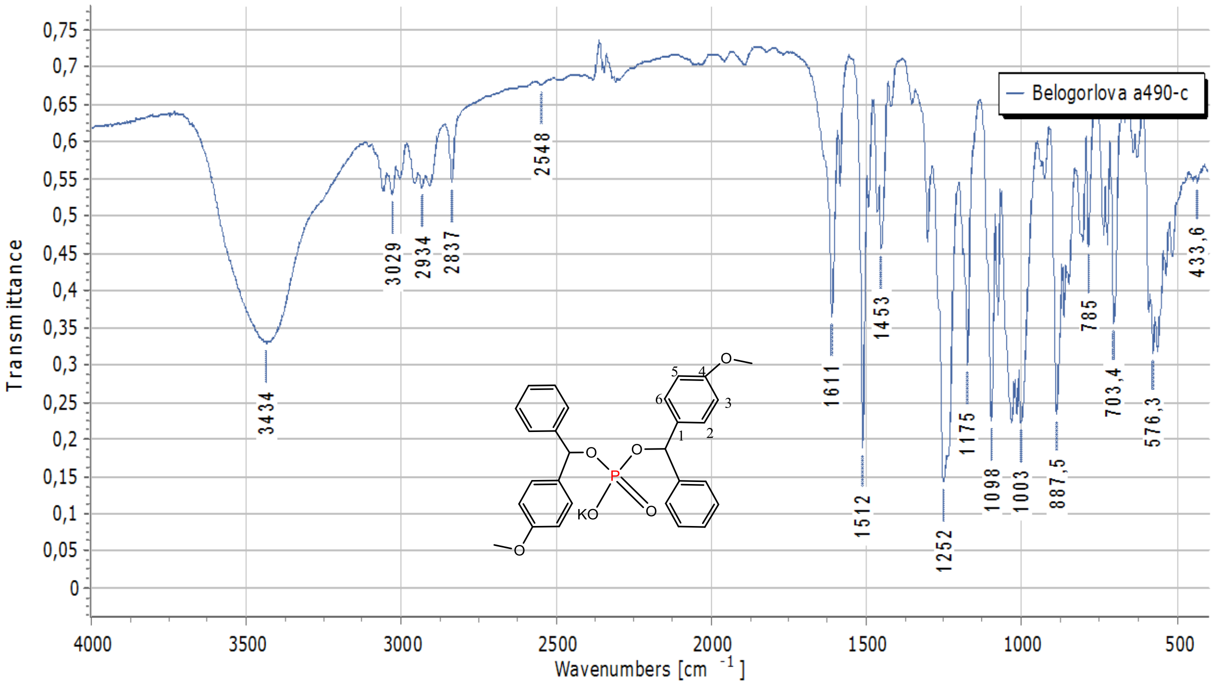

IR spectrum of **2f** (KBr, cm^{-1}).

Potassium bis[(3-methoxyphenyl)(phenyl)methyl]phosphate (2g)

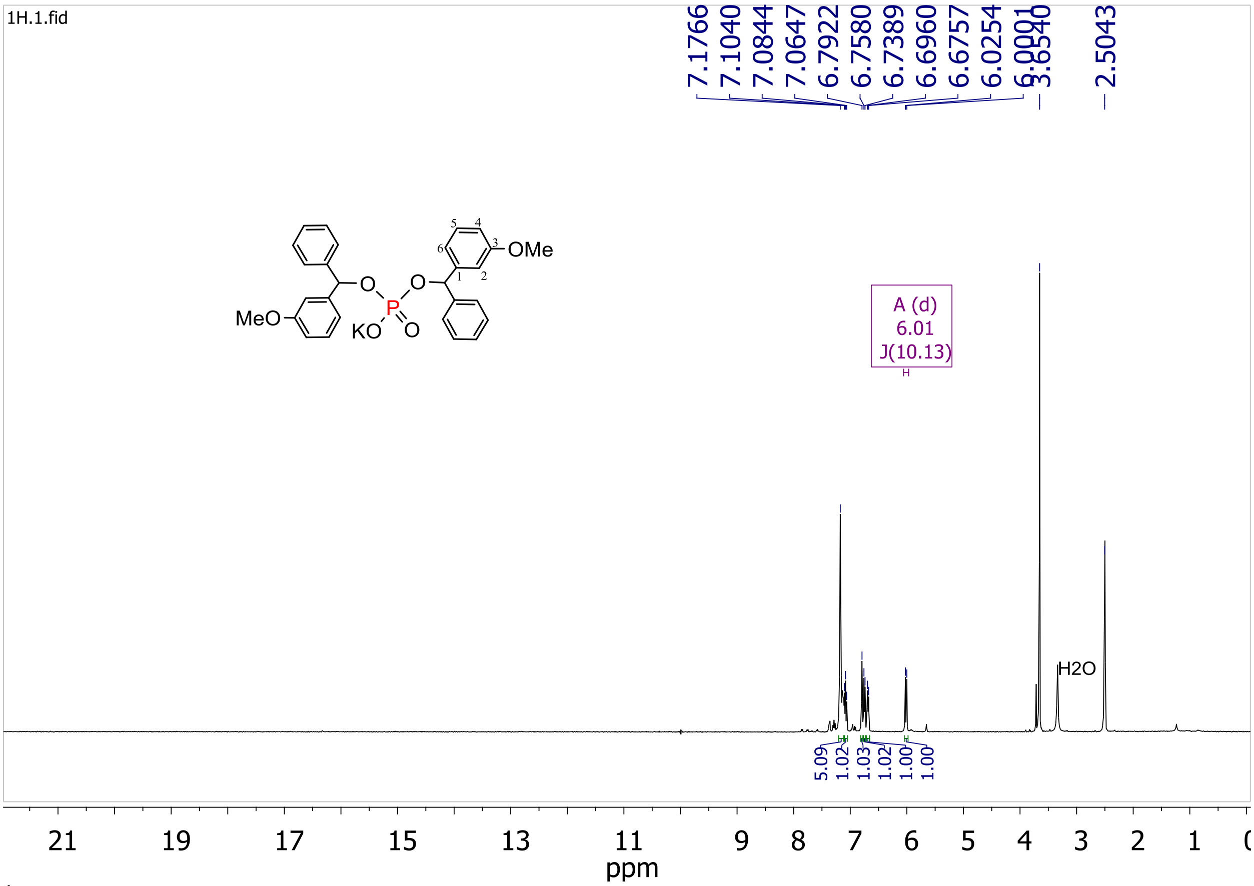

^{1}H NMR spectrum of **2g** (DMSO_d_6).

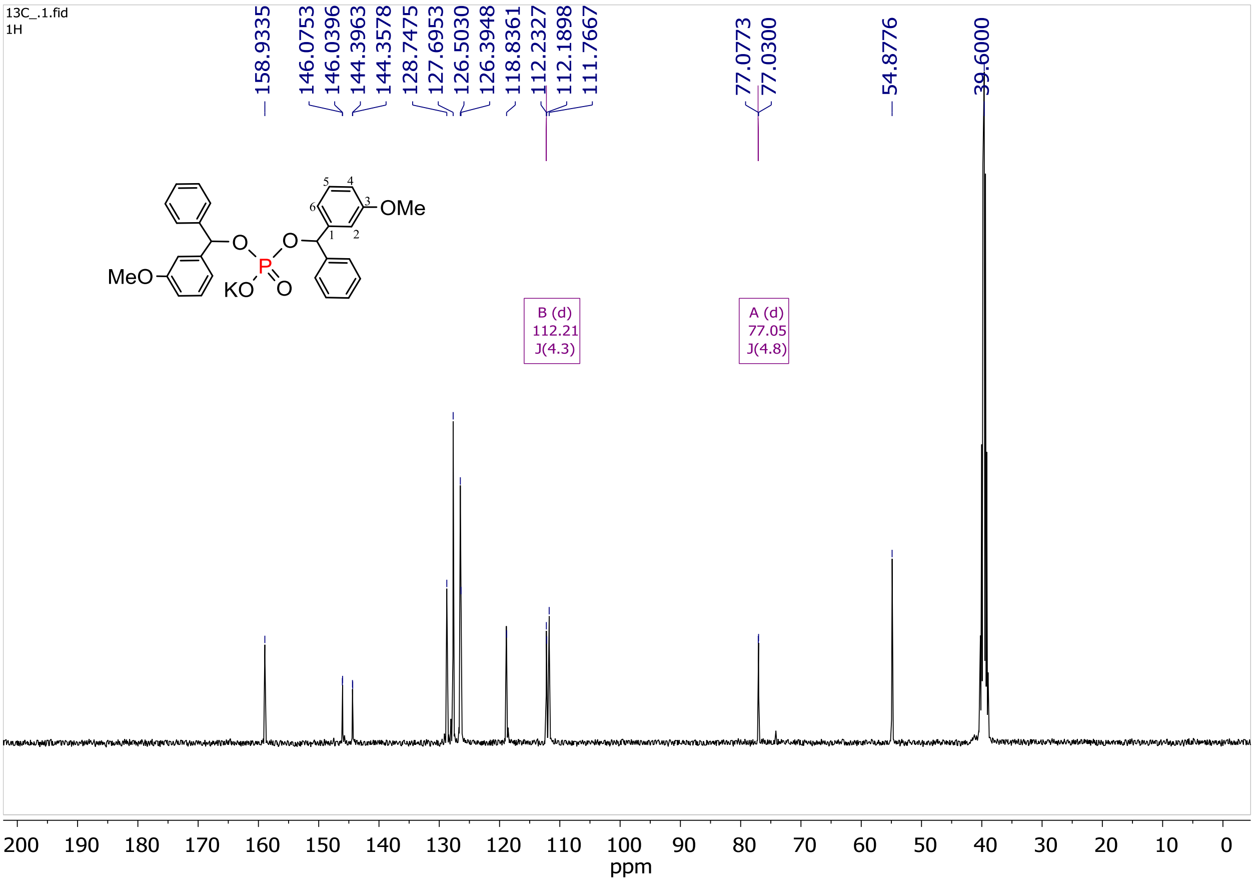

^{13}C NMR spectrum of **2g** (DMSO_d$_6$).

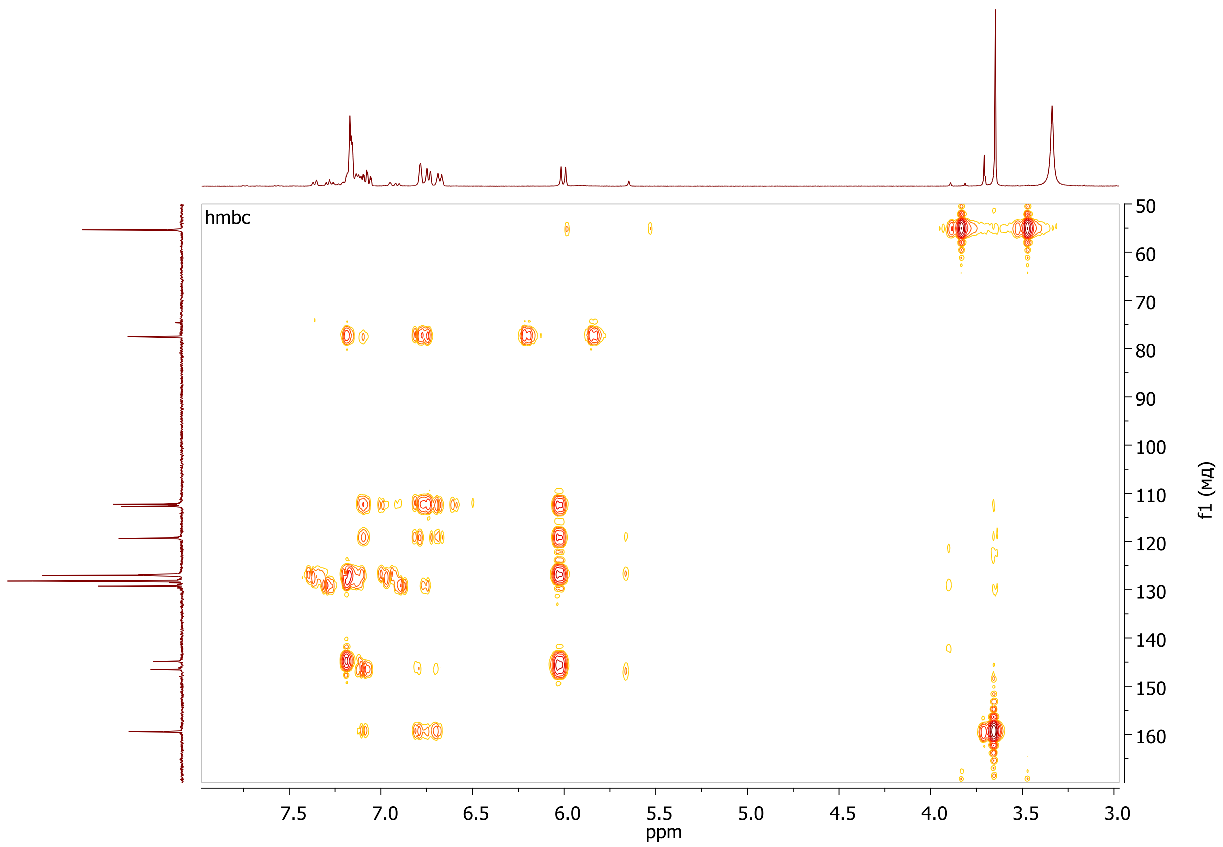

2D HMBC ^{13}C-^{1}H NMR spectrum of **2g** (DMSO_d$_6$).

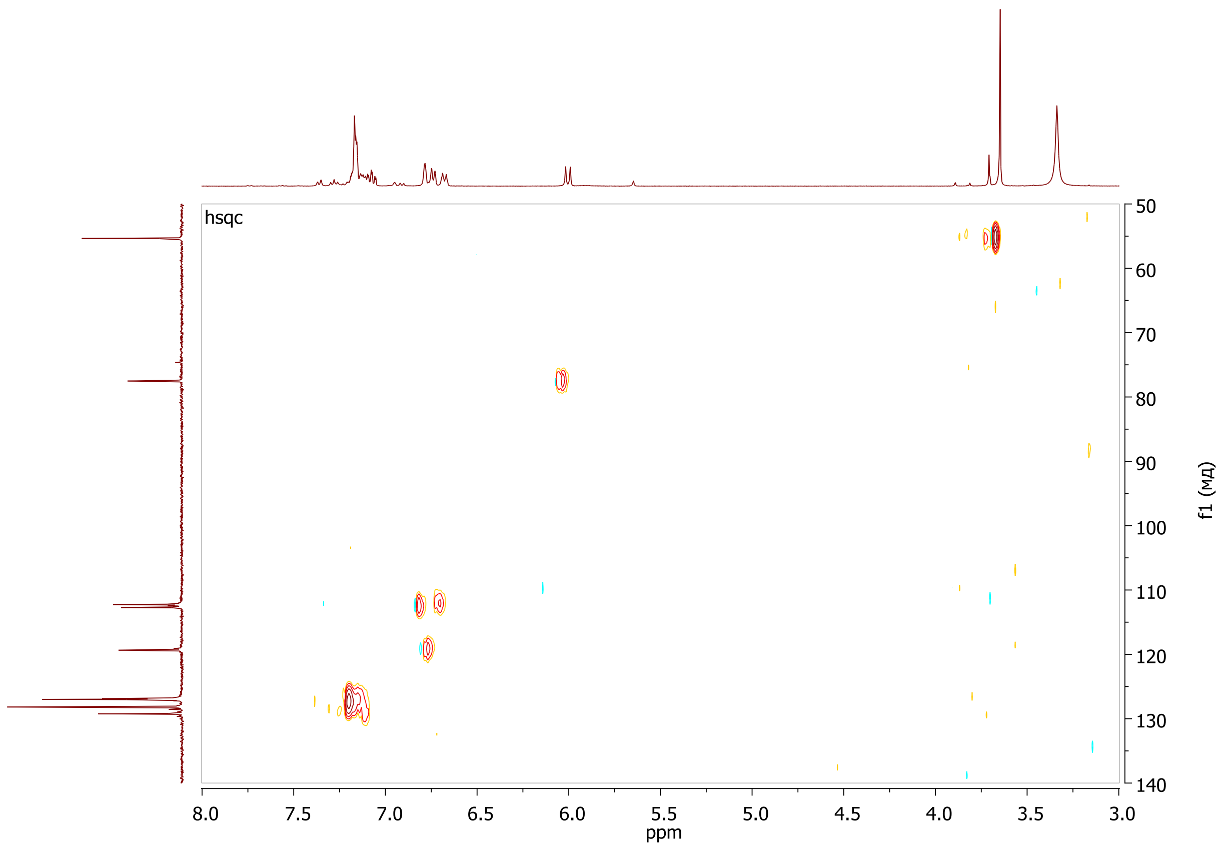

2D HSQC ^{13}C-^{1}H NMR spectrum of **2g** (DMSO_d$_6$).

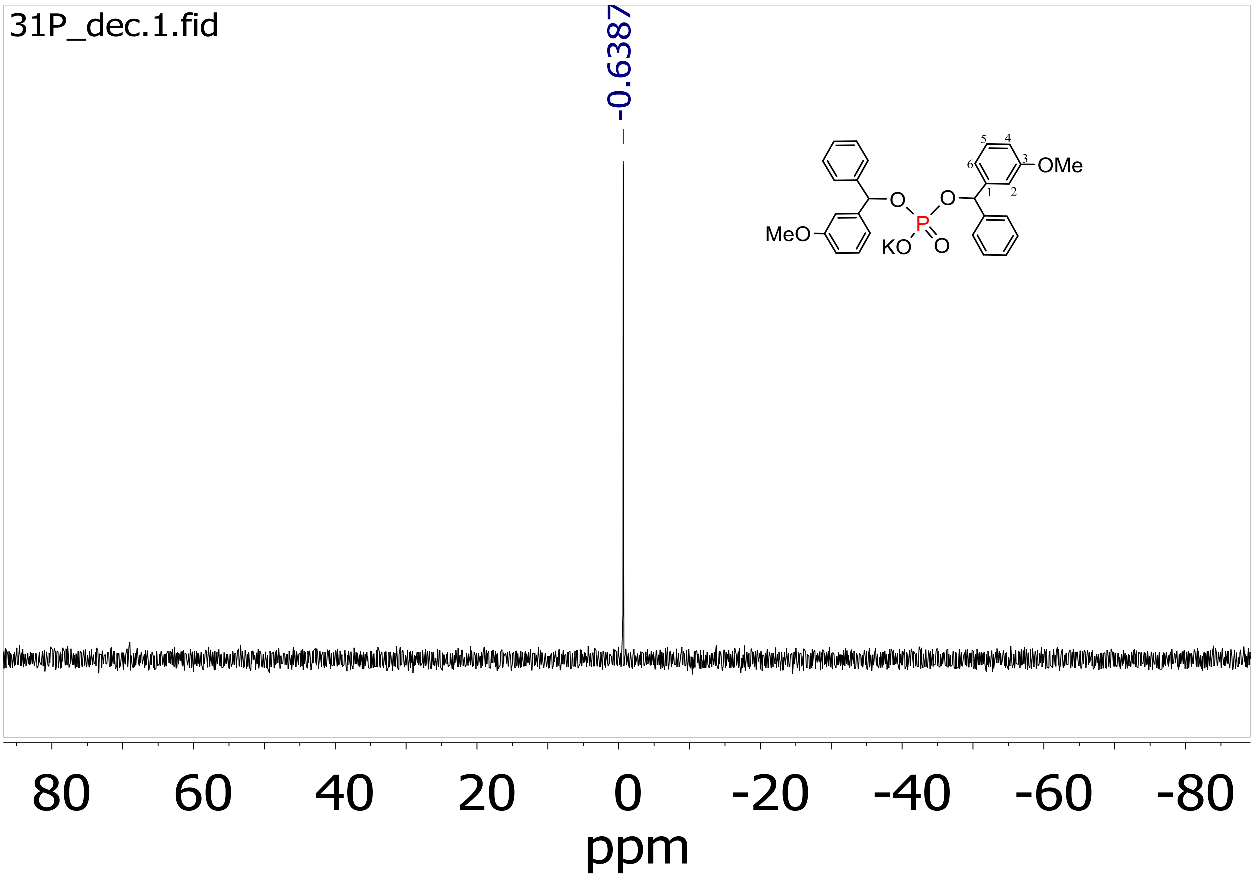

$^{31}P\{^{1}H\}$ NMR spectrum of **2g** (DMSO_d$_6$).

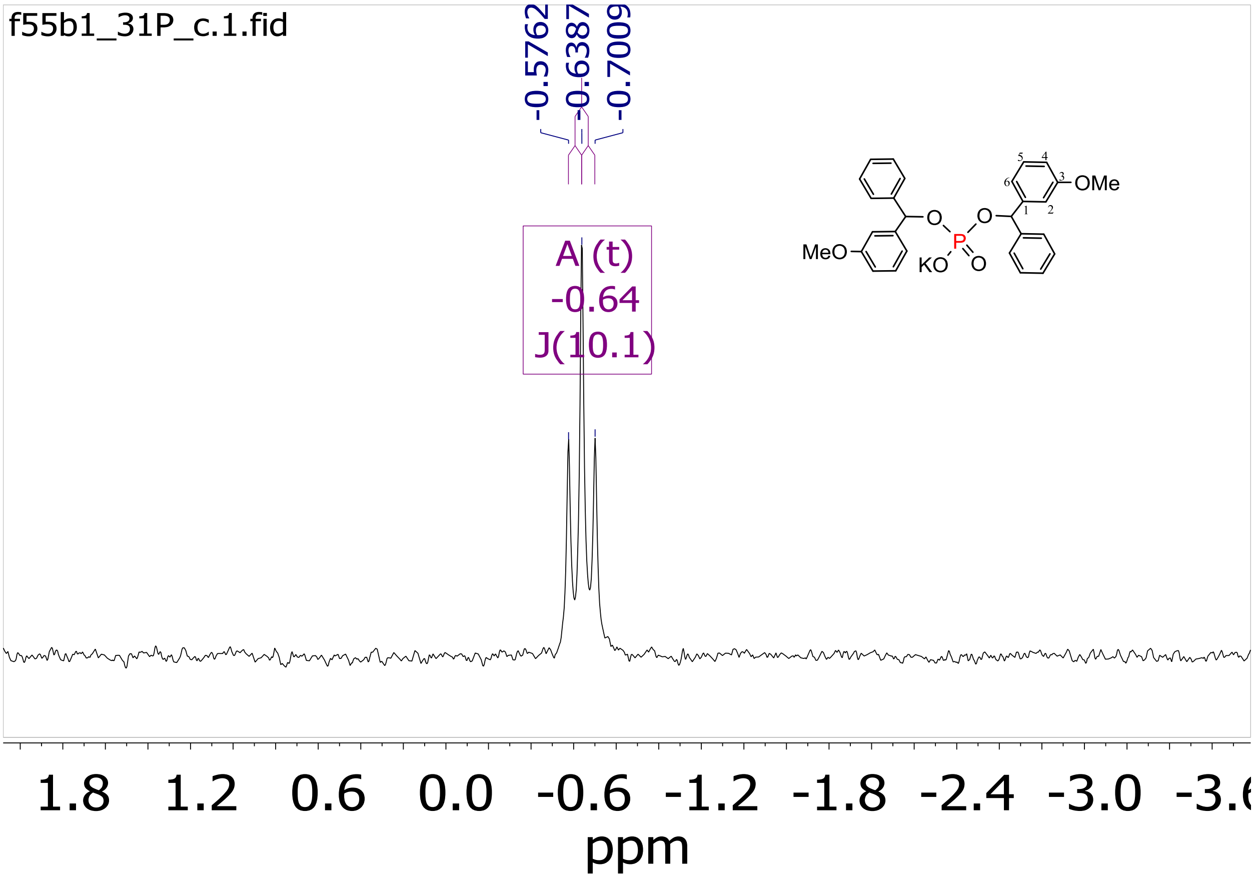

^{31}P NMR spectrum of **2g** (DMSO_d$_6$).

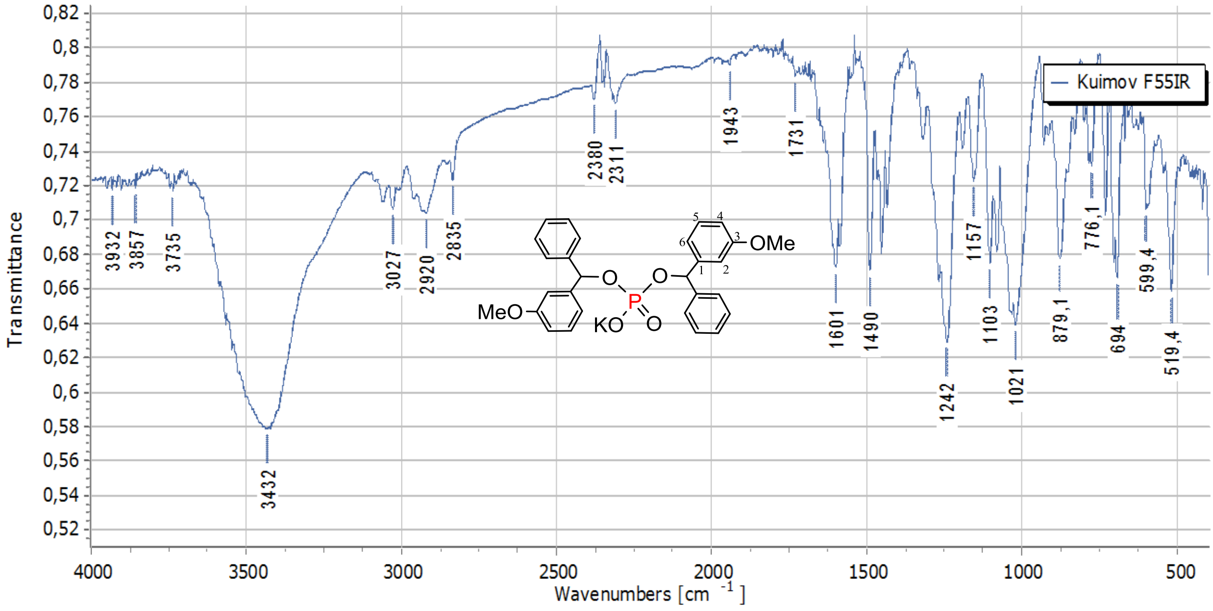

IR spectrum of **2g** (KBr, cm^{-1}).

Potassium bis[[4-(methylthio)phenyl](phenyl)methyl]phosphate (2h).

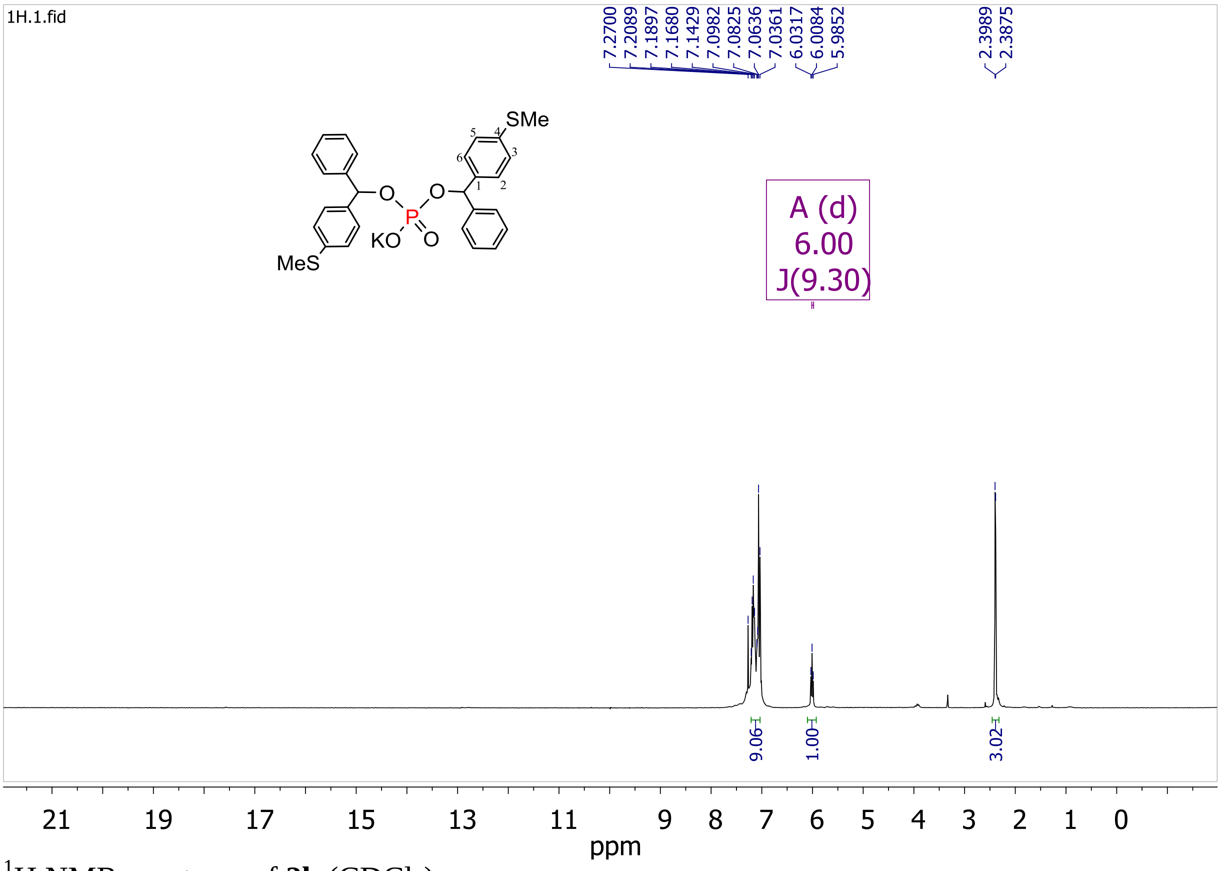

^{1}H NMR spectrum of **2h** ($CDCl_3$).

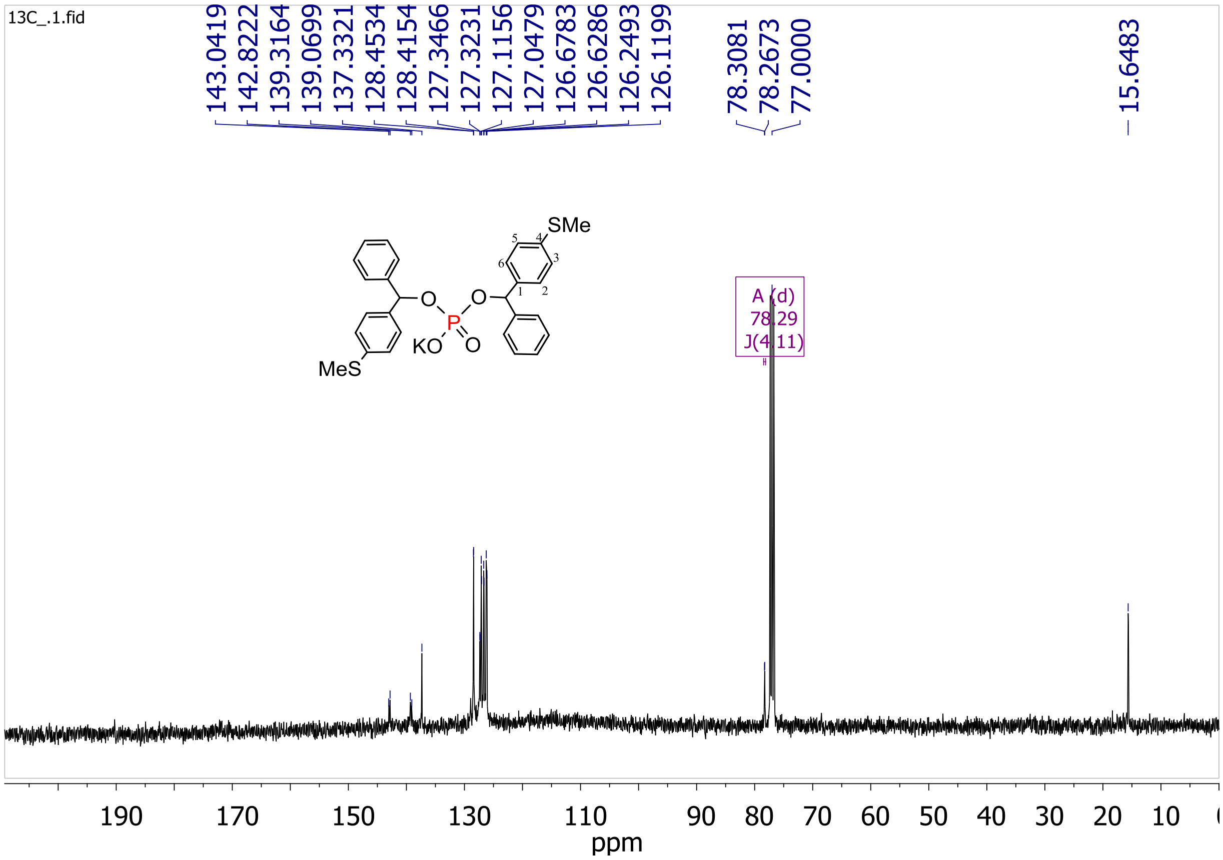

^{13}C NMR spectrum of **2h** ($CDCl_3$).

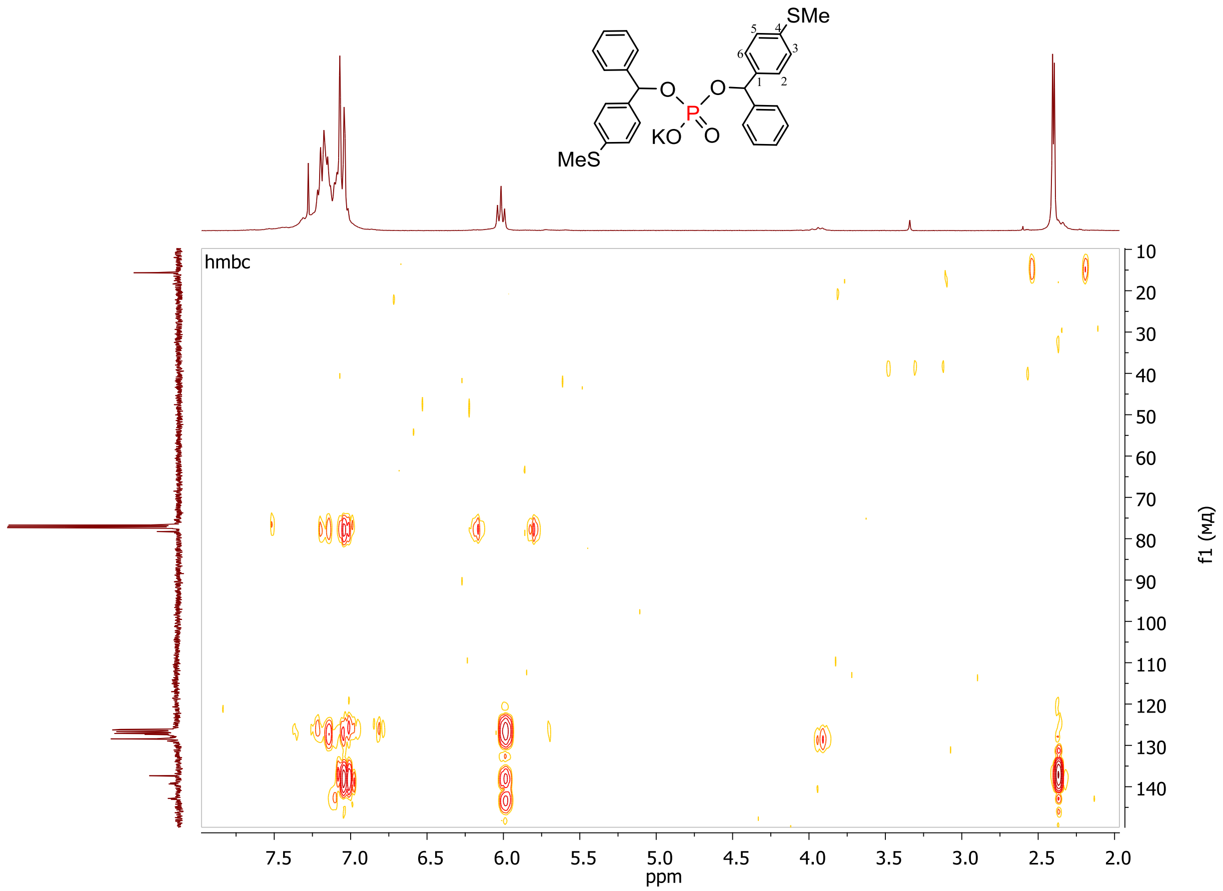

2D HMBC ^{13}C-^{1}H NMR spectrum of **2h** ($CDCl_3$).

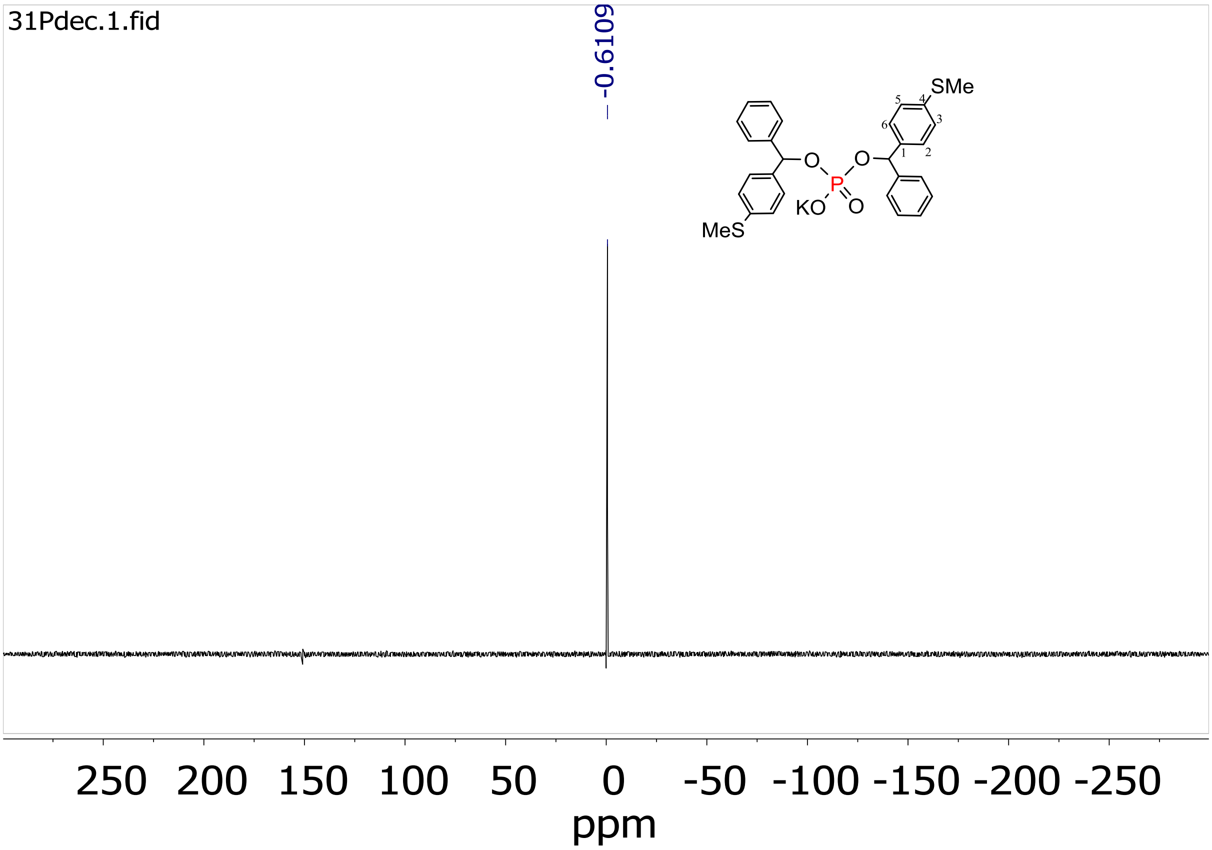

$^{31}P\{^1H\}$ NMR spectrum of **2h** ($CDCl_3$).

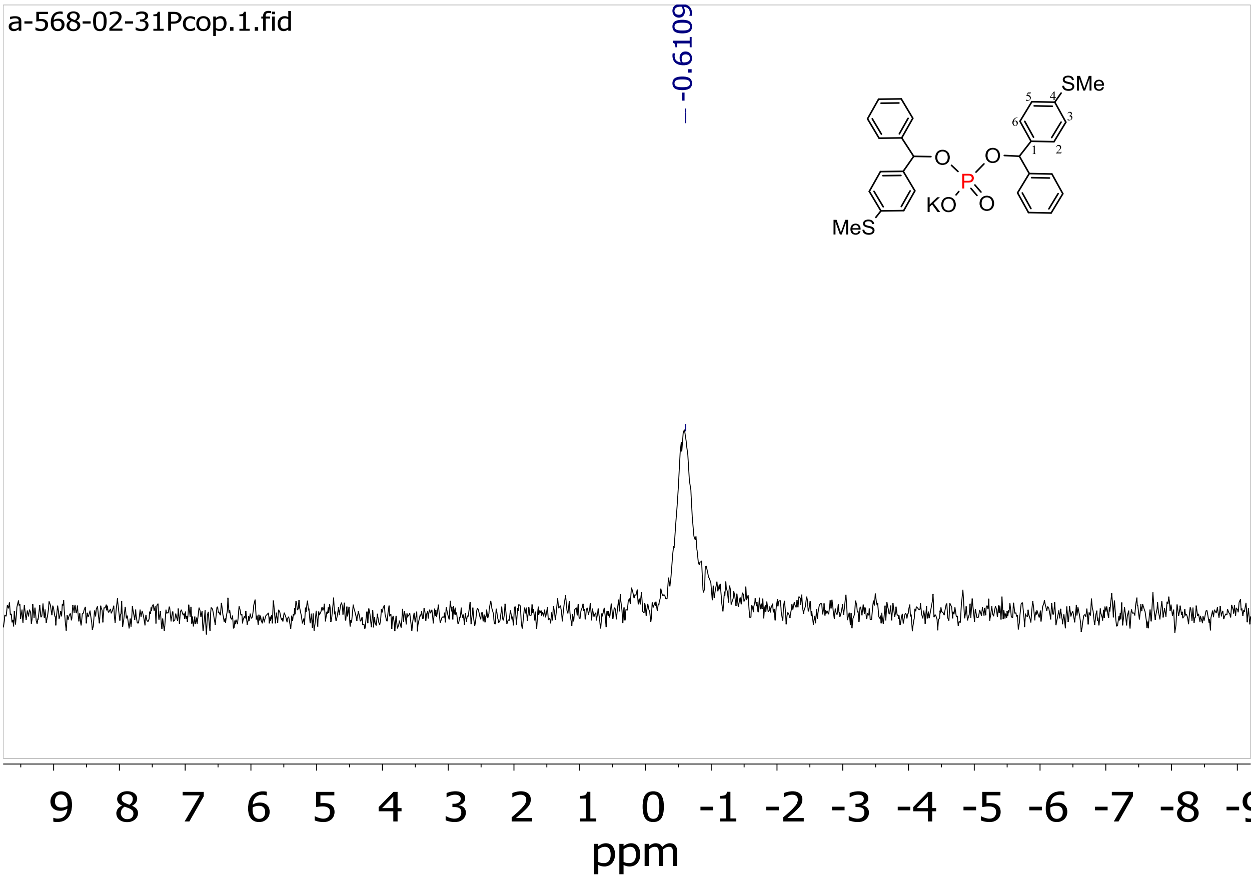

^{31}P NMR spectrum of **2h** ($CDCl_3$).

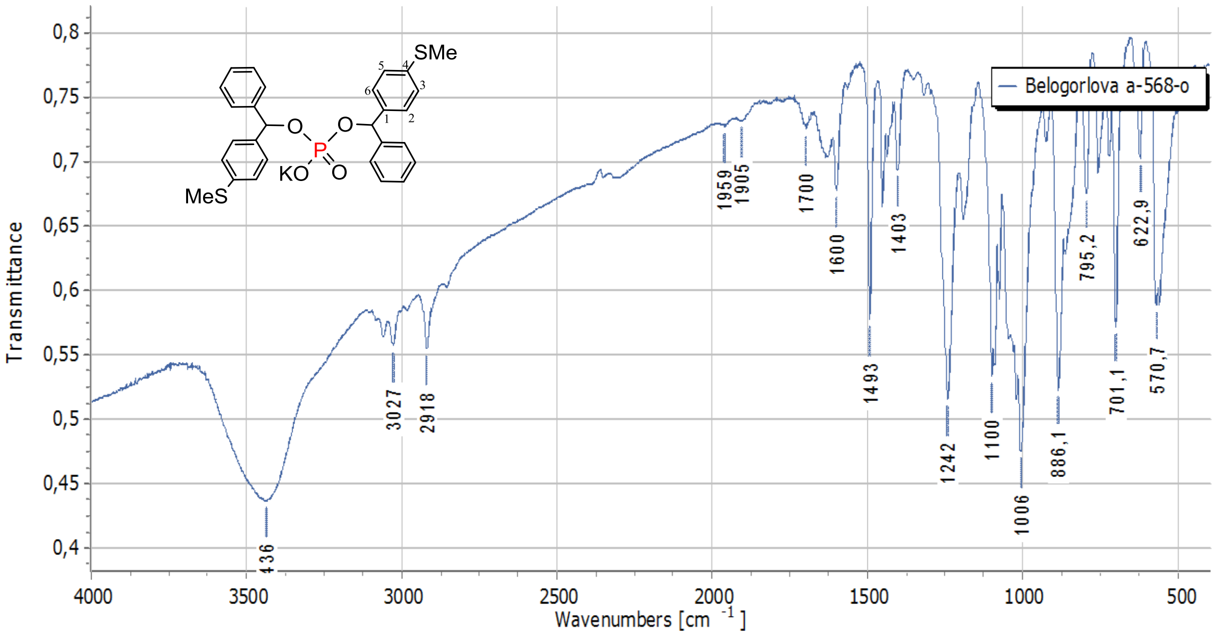

IR spectrum of **2h** (KBr, cm^{-1}).

Potassium bis[[4-(benzyloxy)phenyl](phenyl)methyl]phosphate (2i).

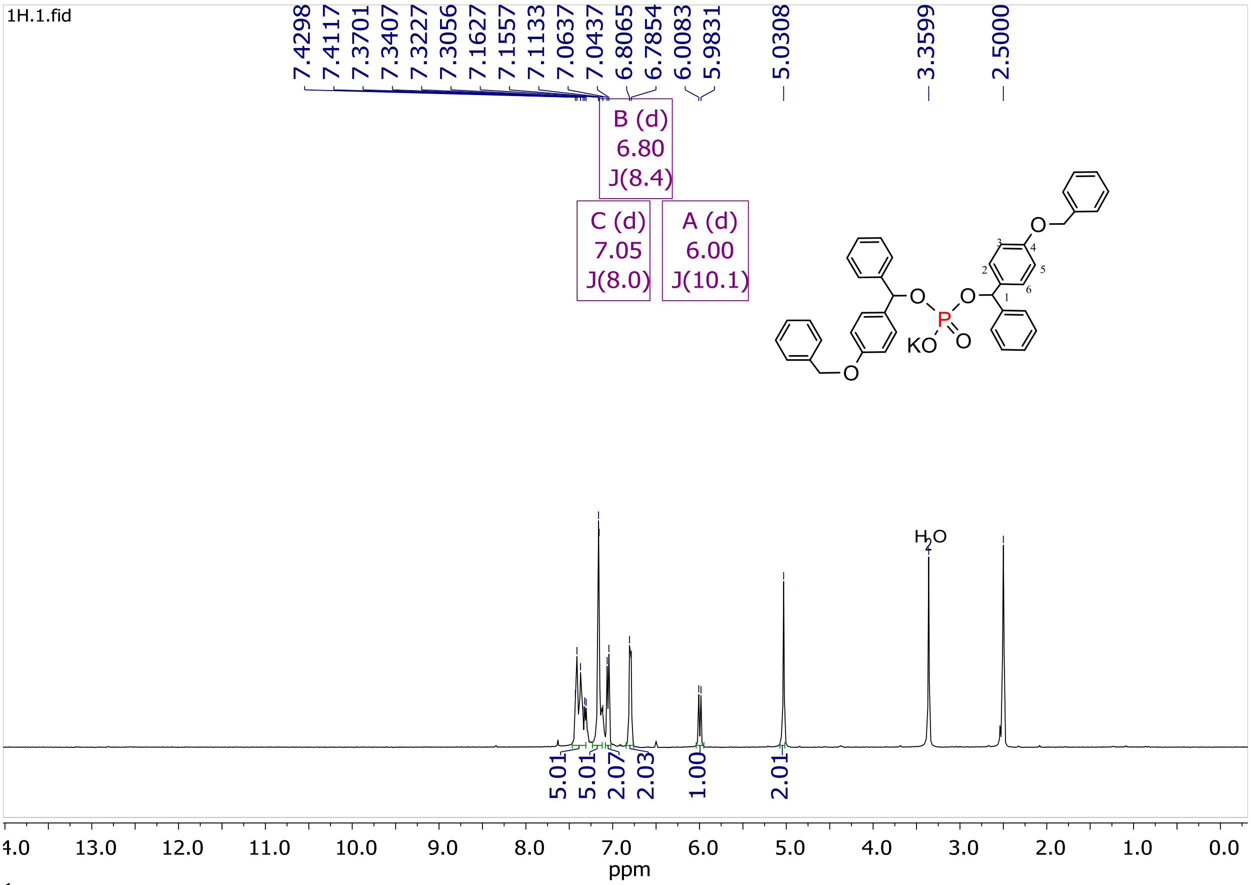

^{1}H NMR spectrum of **2i** (DMSO_d$_6$).

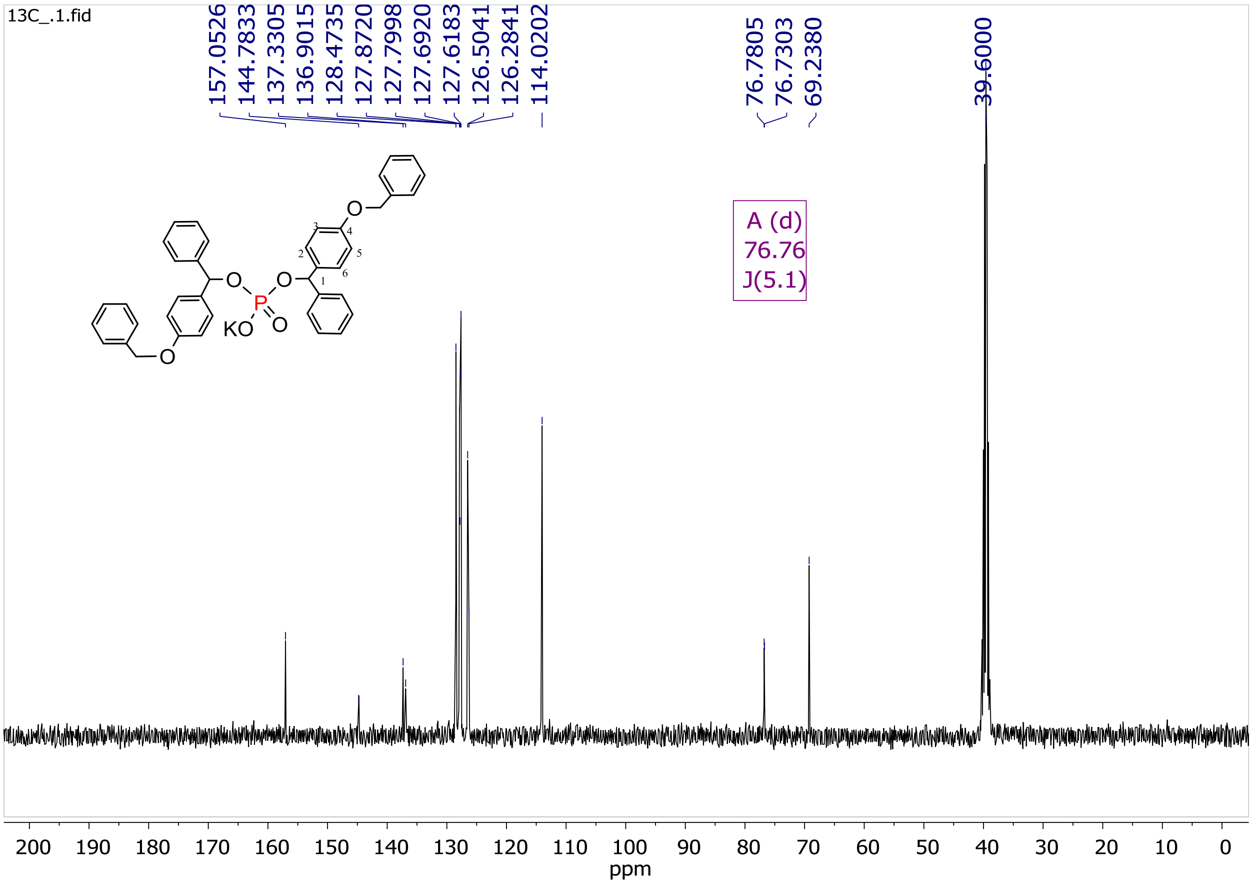

^{13}C NMR spectrum of **2i** (DMSO_d$_6$).

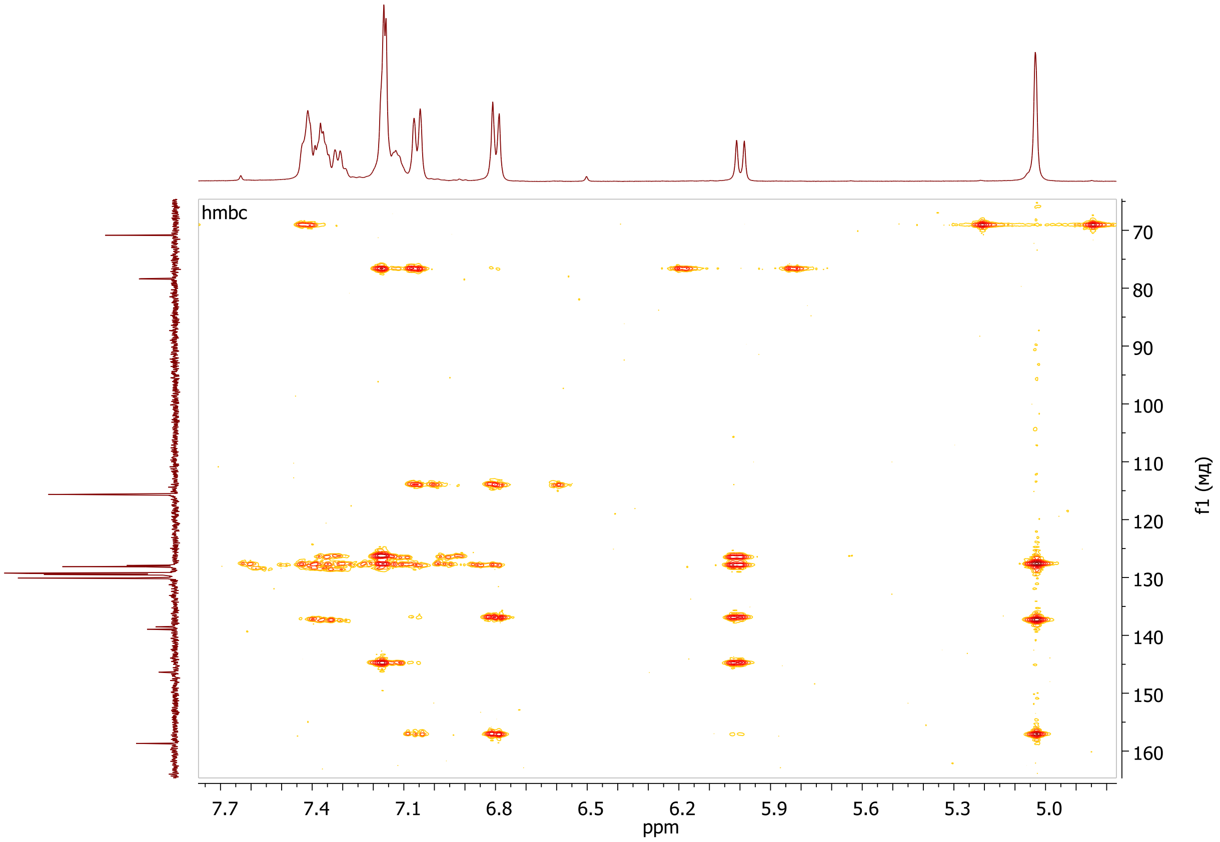

2D HMBC ^{13}C-^{1}H NMR spectrum of **2i** (DMSO_d$_6$).

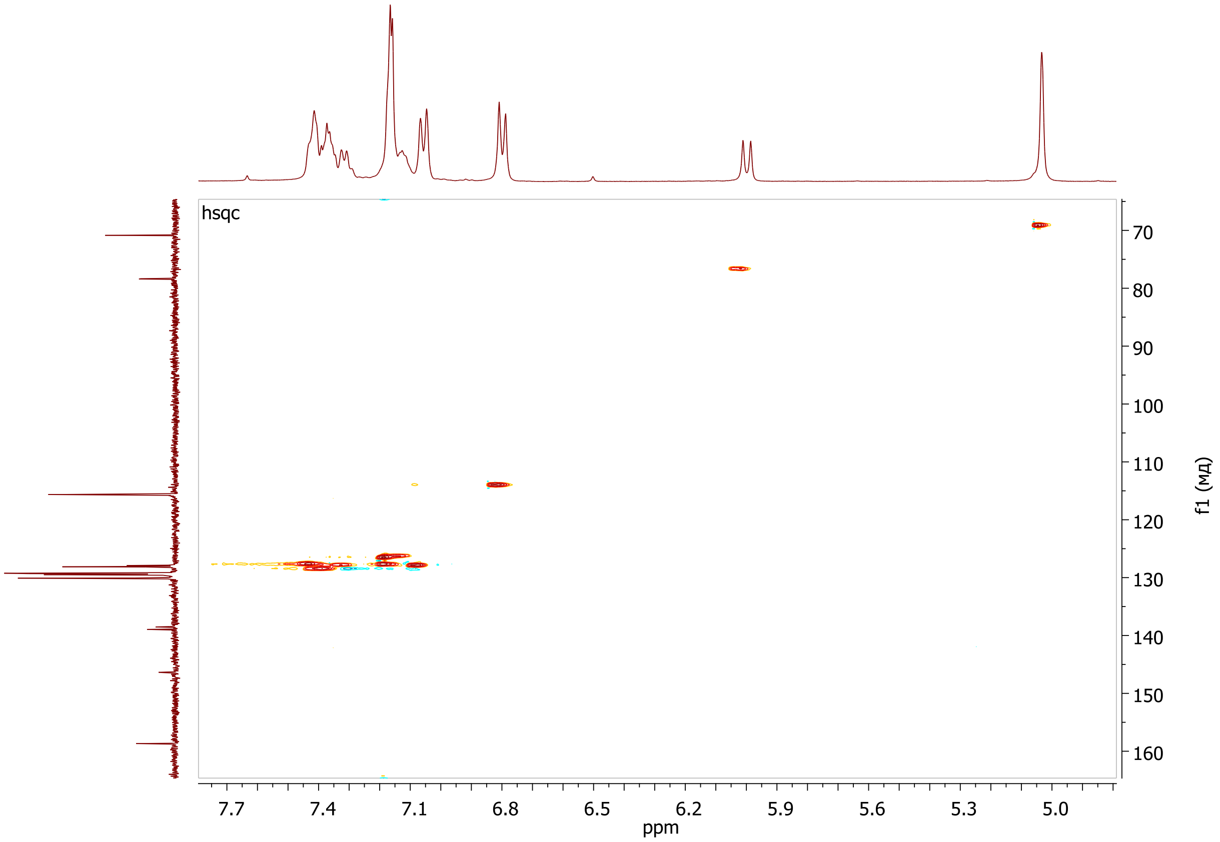

2D HSQC ^{13}C-^{1}H NMR spectrum of **2i** (DMSO_d_6).

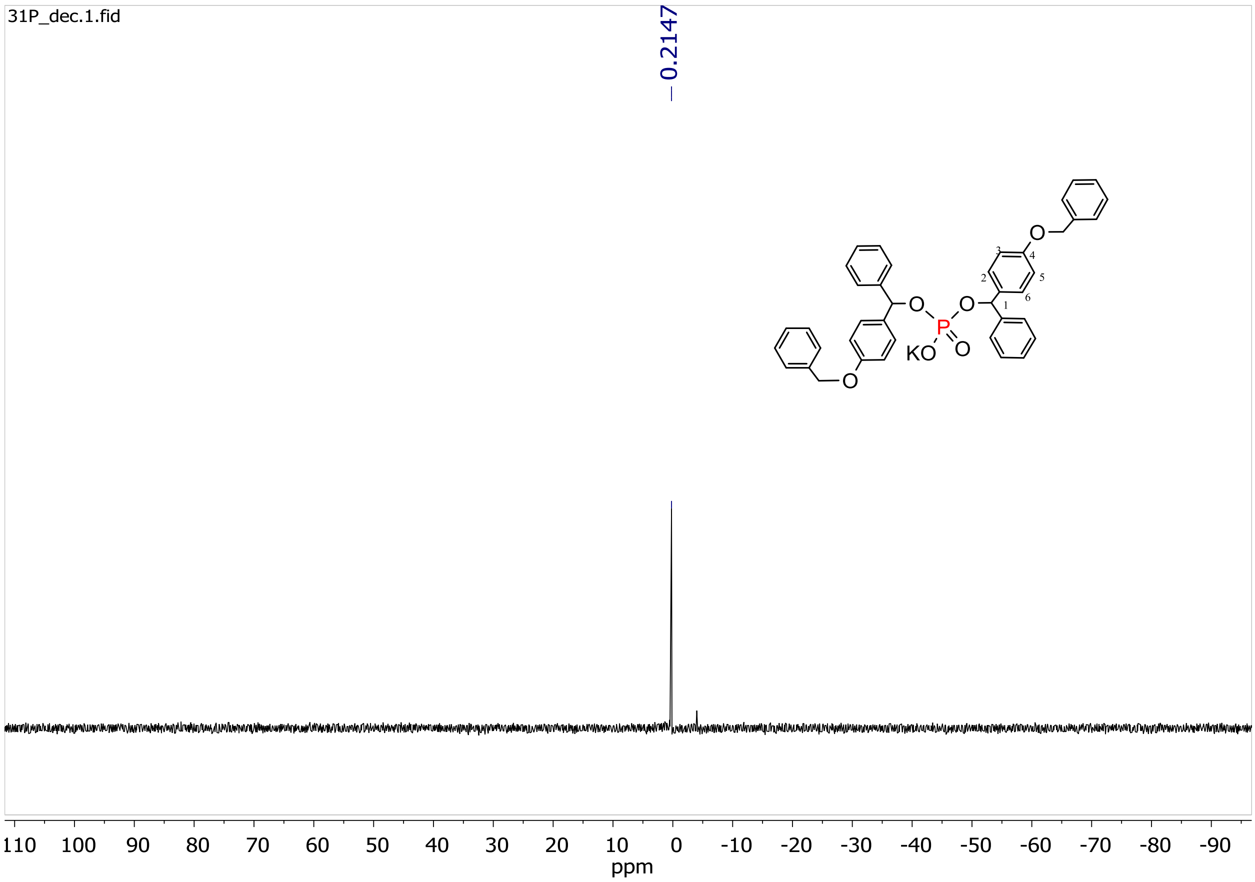

$^{31}P\{^{1}H\}$ NMR spectrum of **2i** (DMSO_d_6).

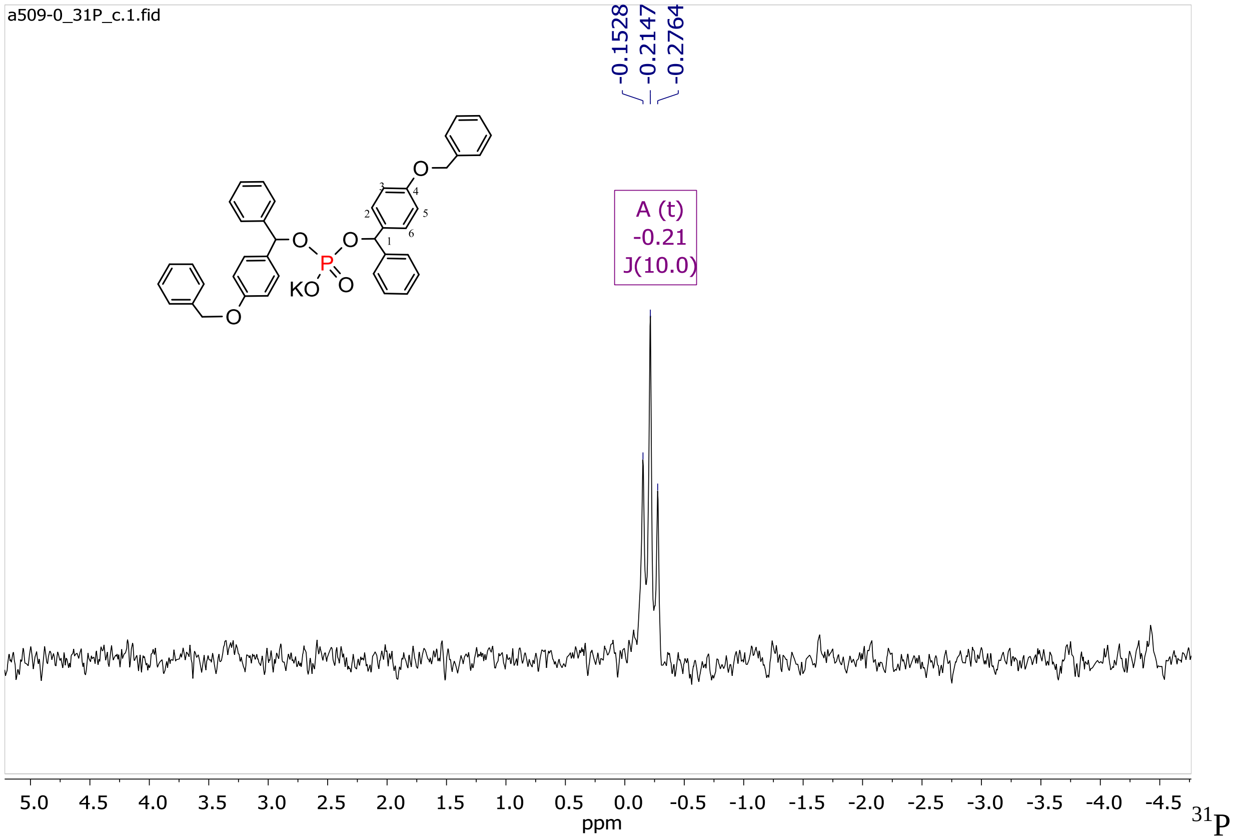

^{31}P NMR spectrum of **2i** (DMSO_d_6).

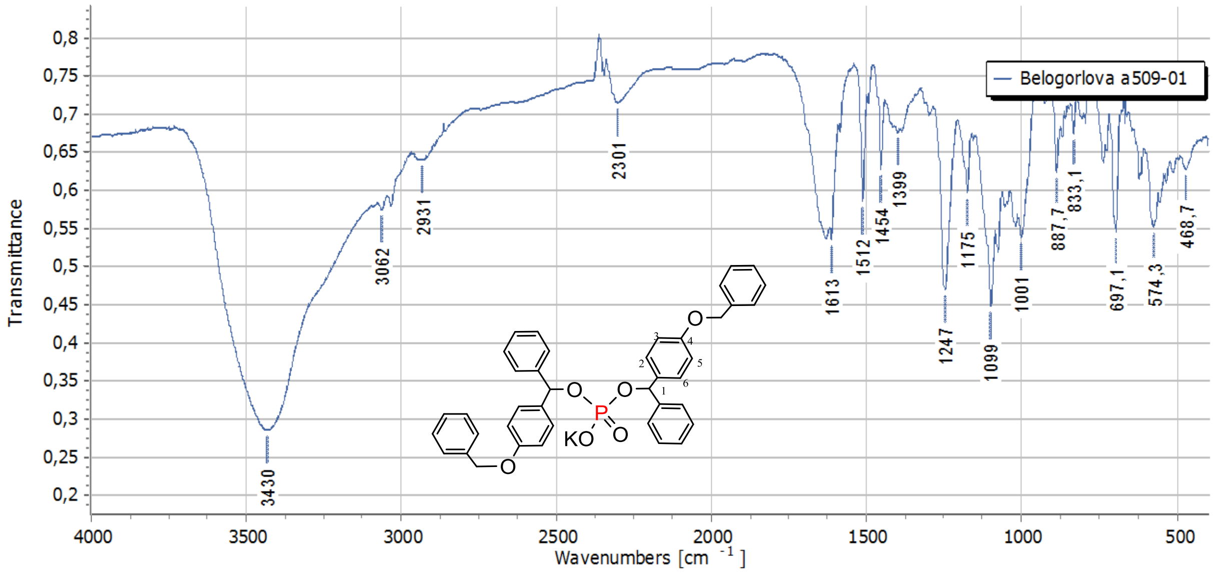

IR spectrum of **2i** (KBr, cm^{-1}).

Potassium bis[(3-chlorophenyl)(phenyl)methyl]phosphate (2j).

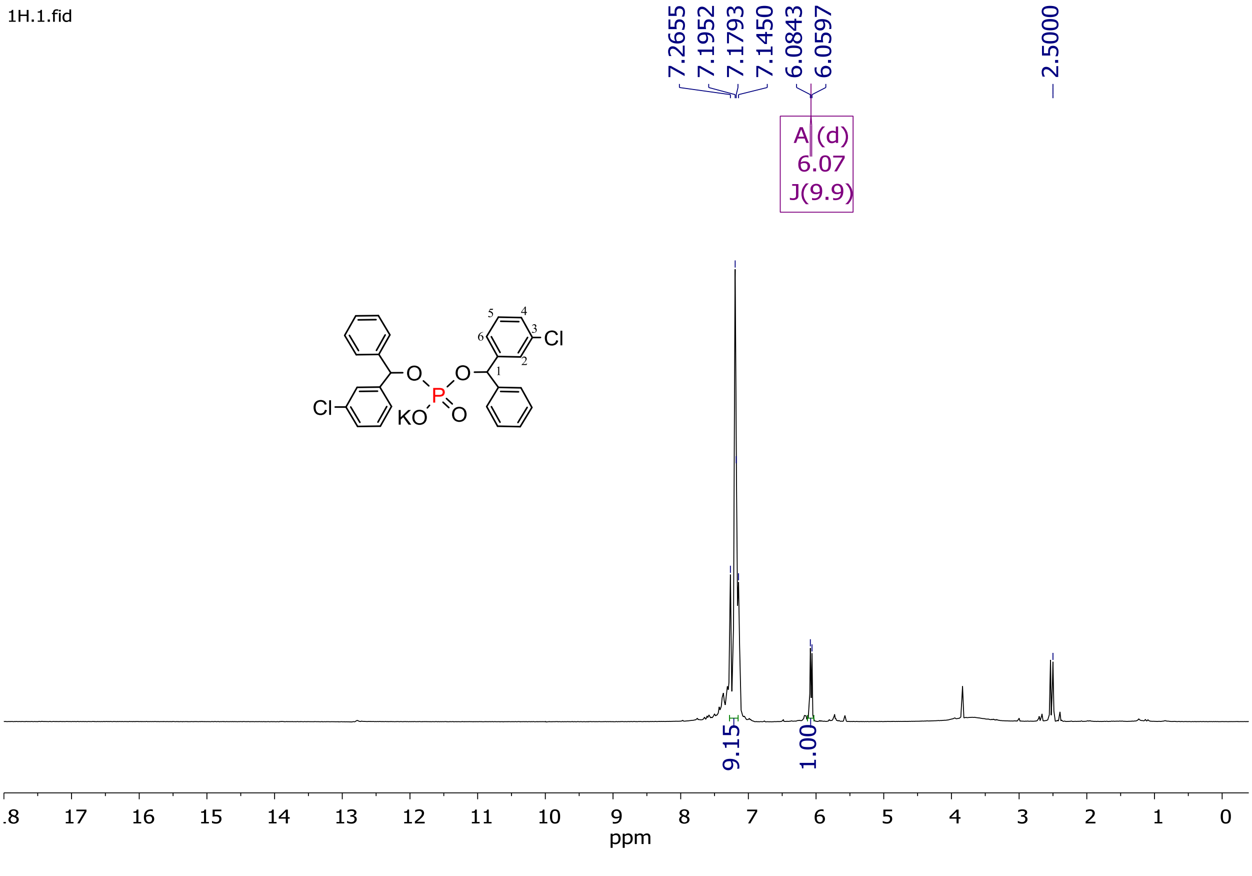

^{1}H NMR spectrum of **2j** (DMSO_d$_6$).

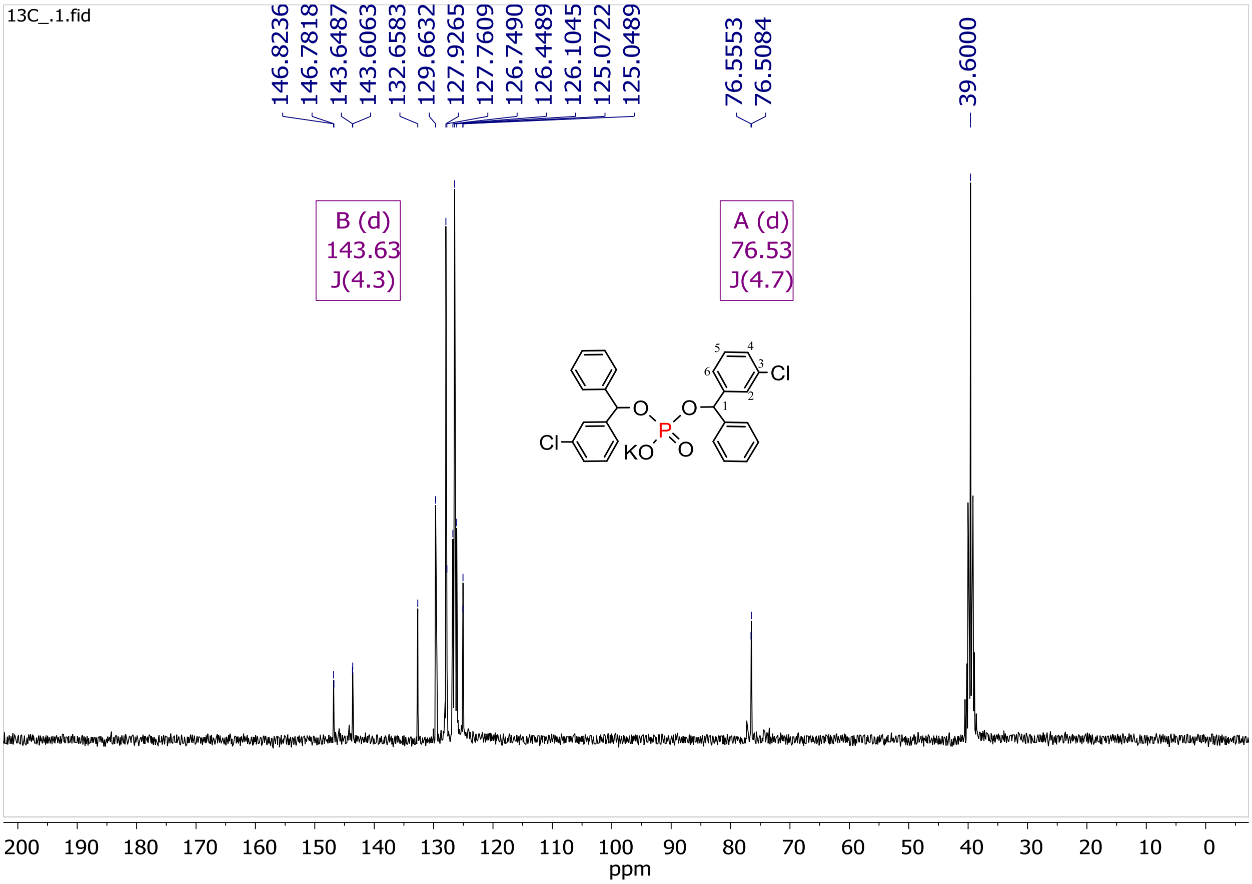

^{13}C NMR spectrum of **2j** (DMSO_d$_6$).

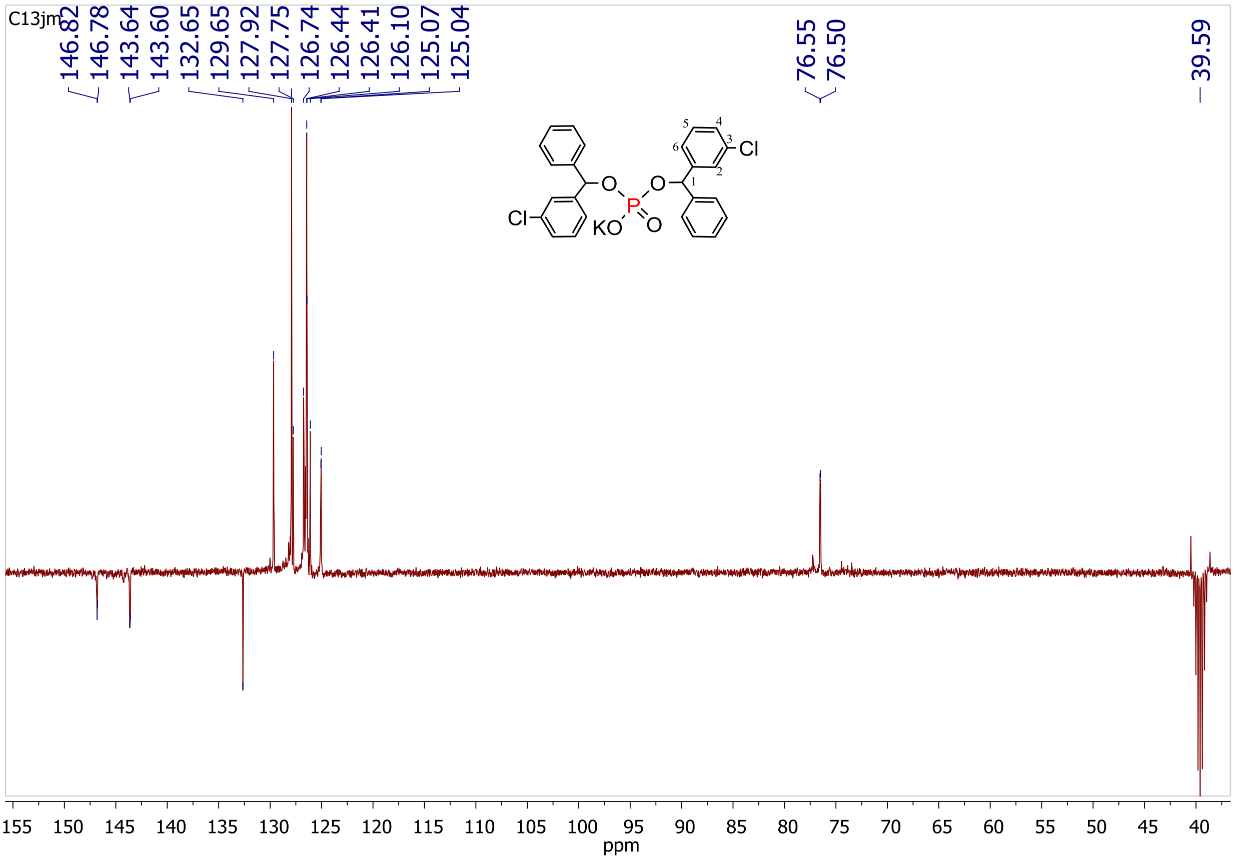

C^{13}jm NMR spectrum of **2j** (DMSO_d$_6$).

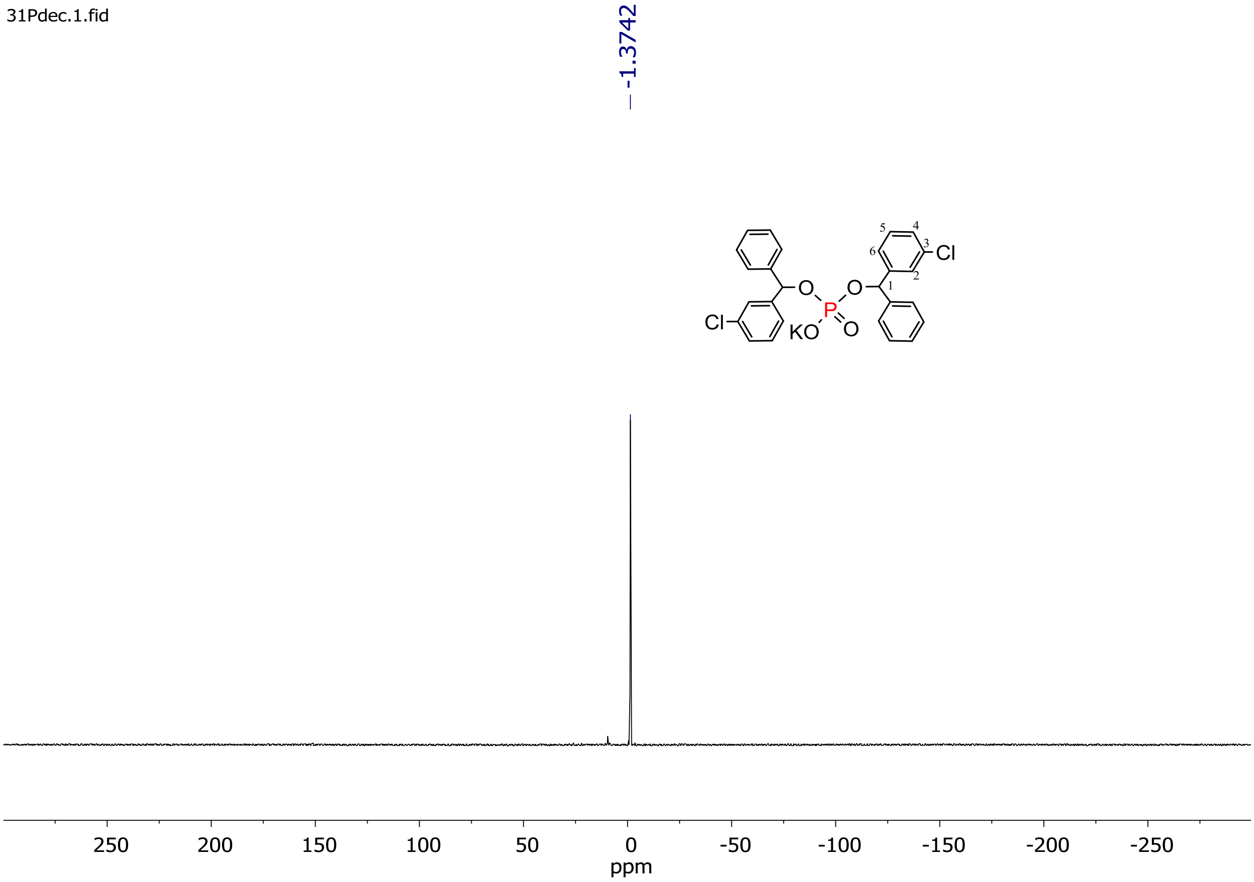

$^{31}P\{^1H\}$ NMR spectrum of **2j** (DMSO_d$_6$).

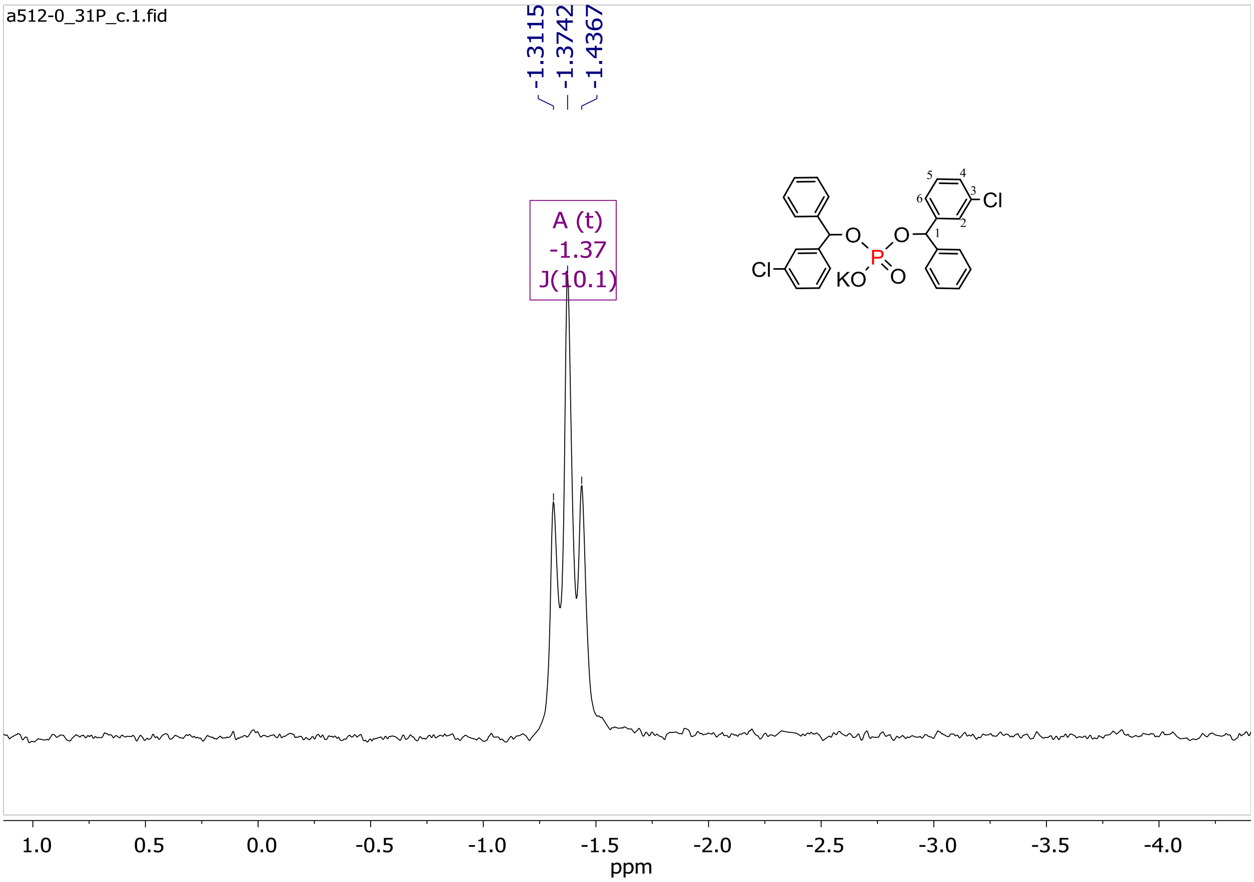

^{31}P NMR spectrum of **2j** (DMSO_d$_6$).

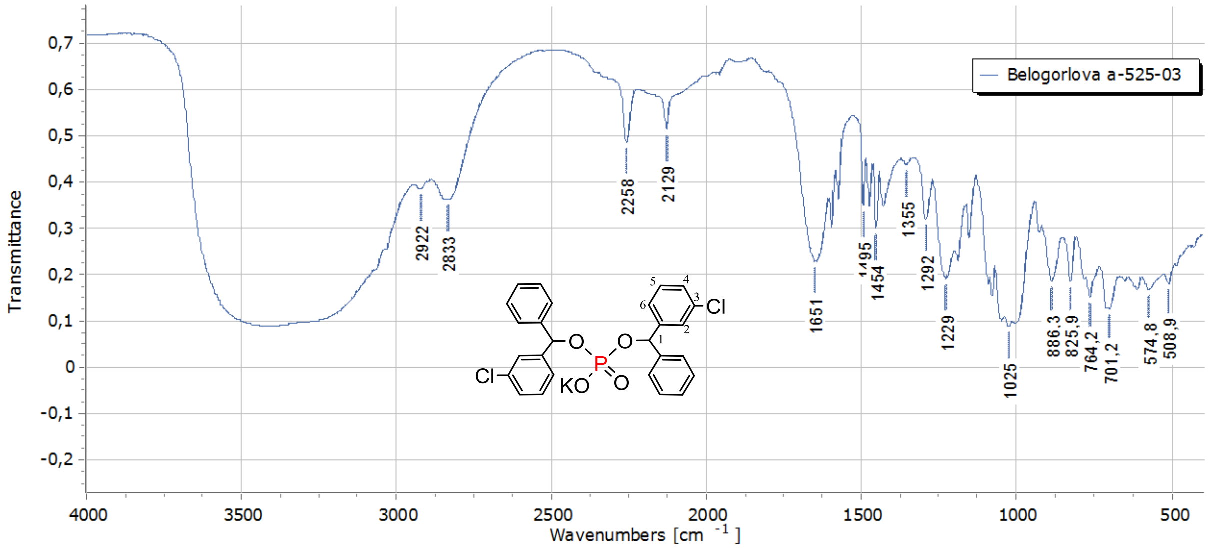

IR spectrum of **2j** (KBr, cm^{-1}).

Potassium bis[(3-fluorophenyl)(phenyl)methyl]phosphate (2k).

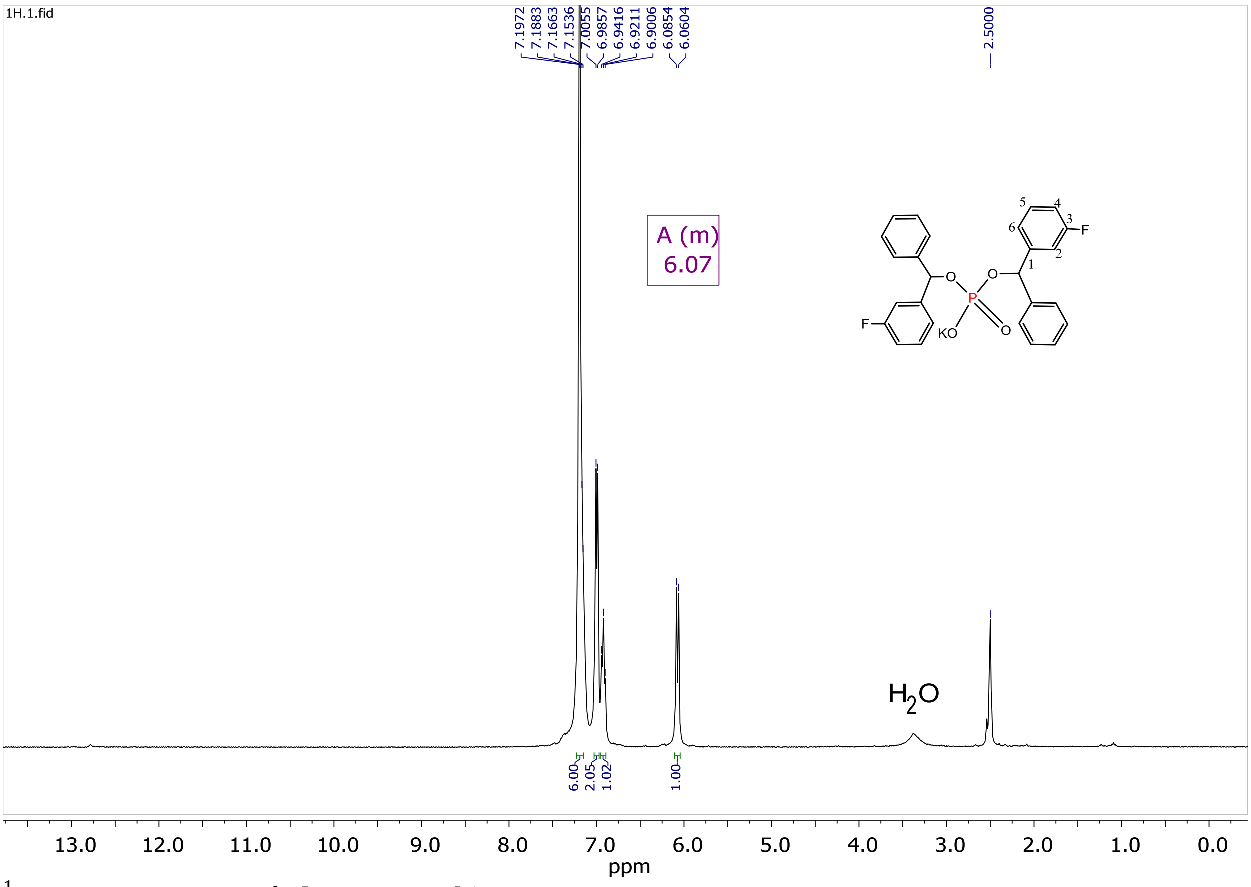

^{1}H NMR spectrum of **2k** (DMSO_d$_6$).

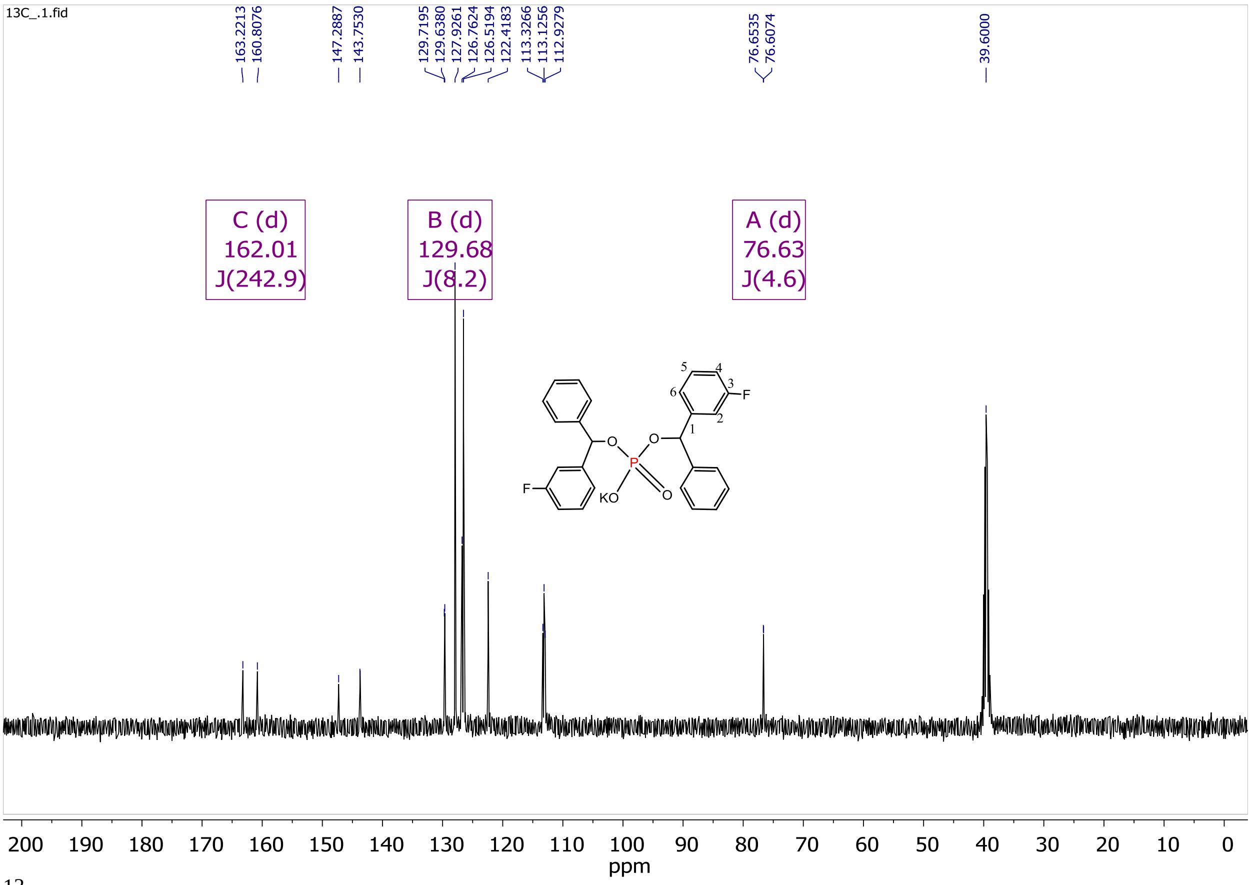

^{13}C NMR spectrum of **2k** (DMSO_d$_6$).

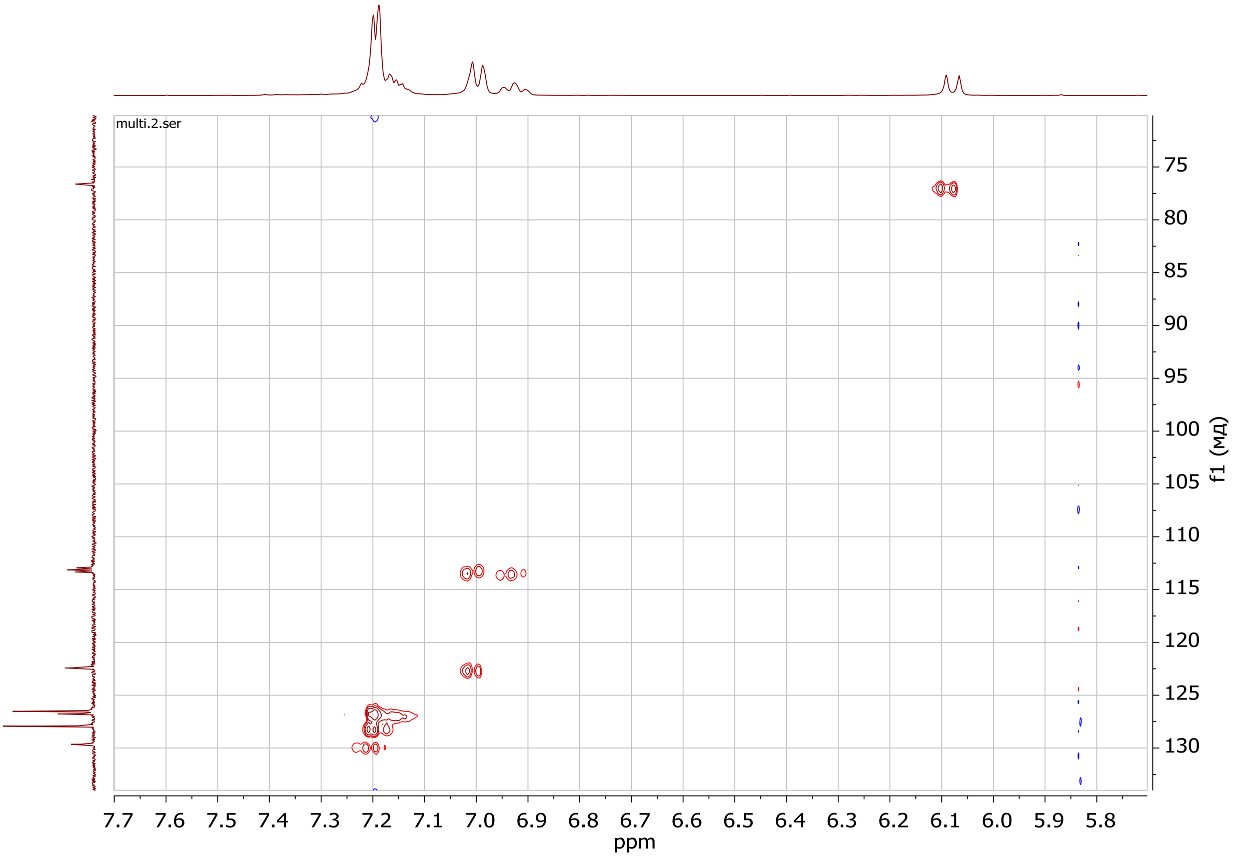

2D HSQC ^{13}C-^{1}H spectrum of **2k** (DMSO_d$_6$).

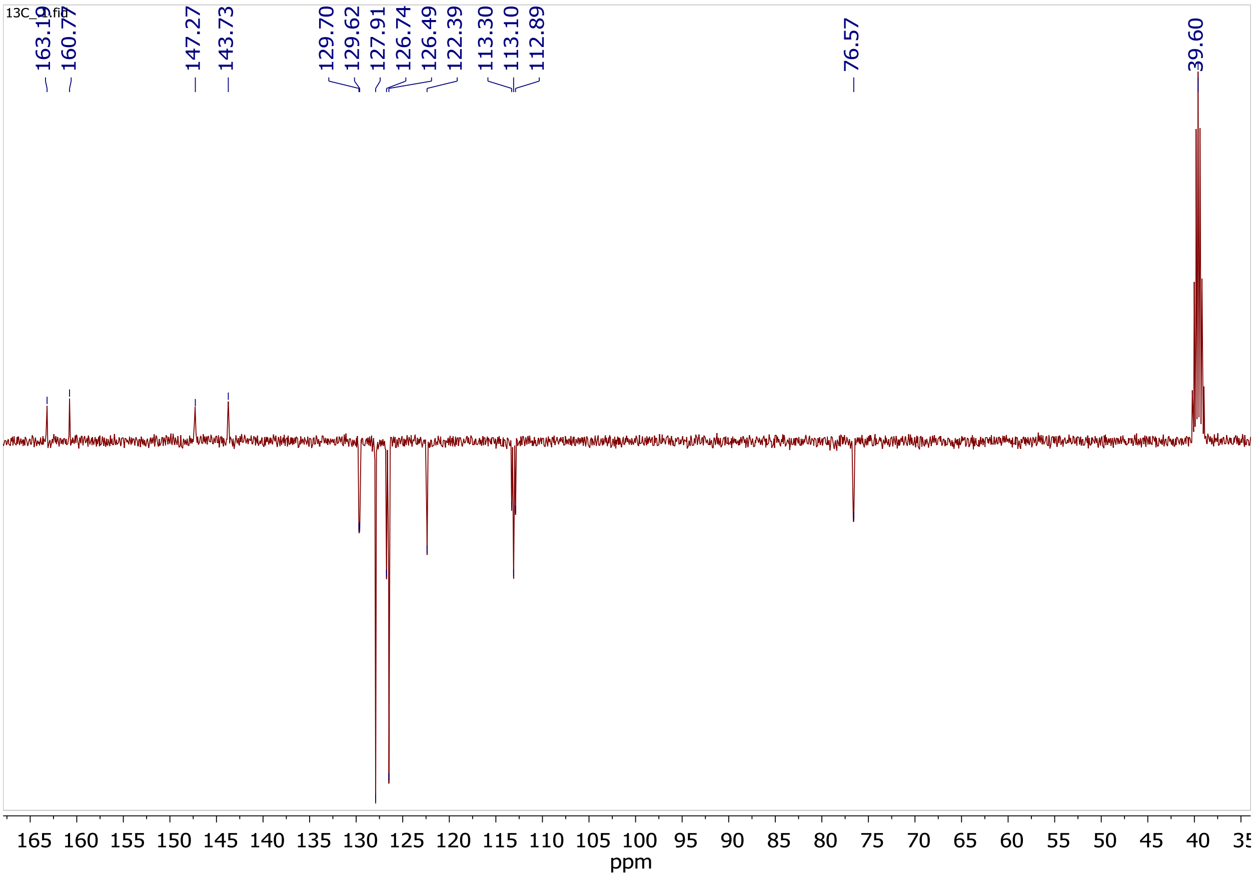

^{13}Cjm spectrum of **2k** (DMSO_d$_6$)

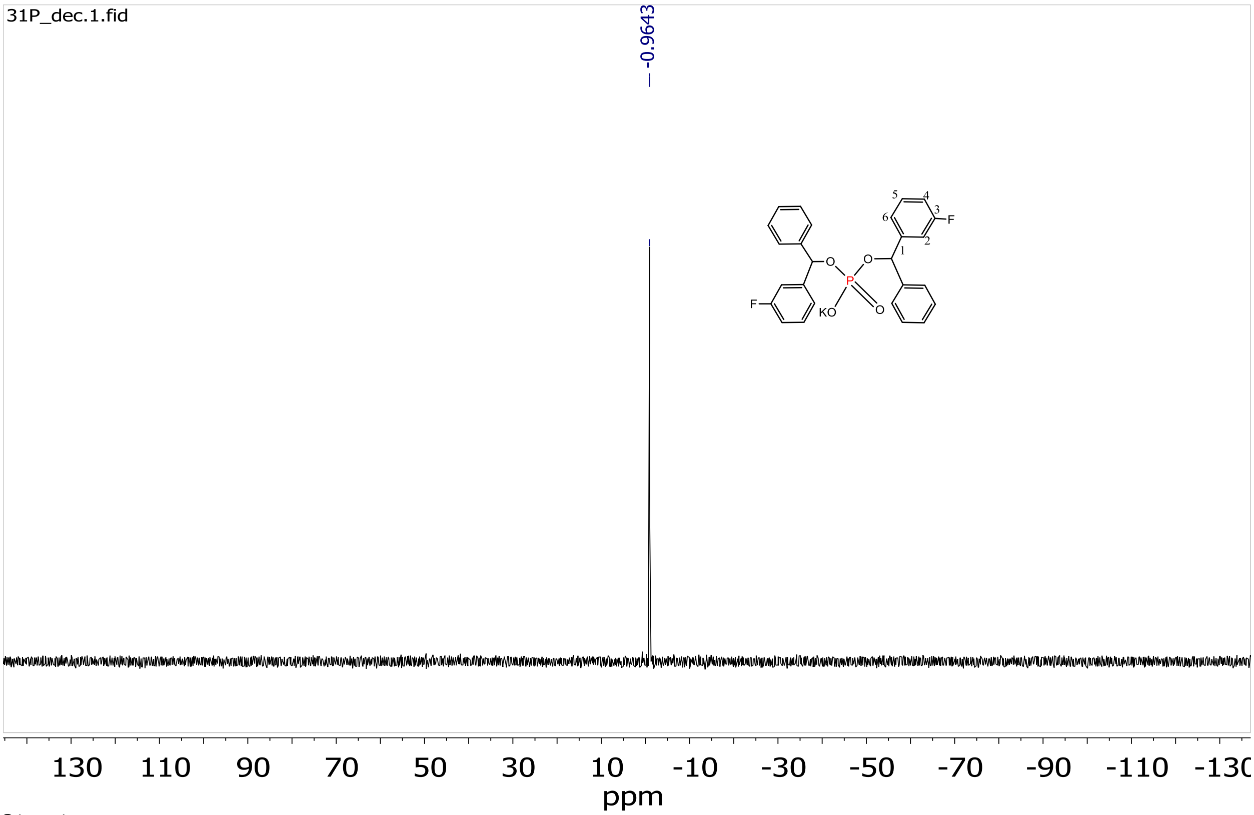

^{31}P{^{1}H} NMR spectrum of **2k** (DMSO_d$_6$).

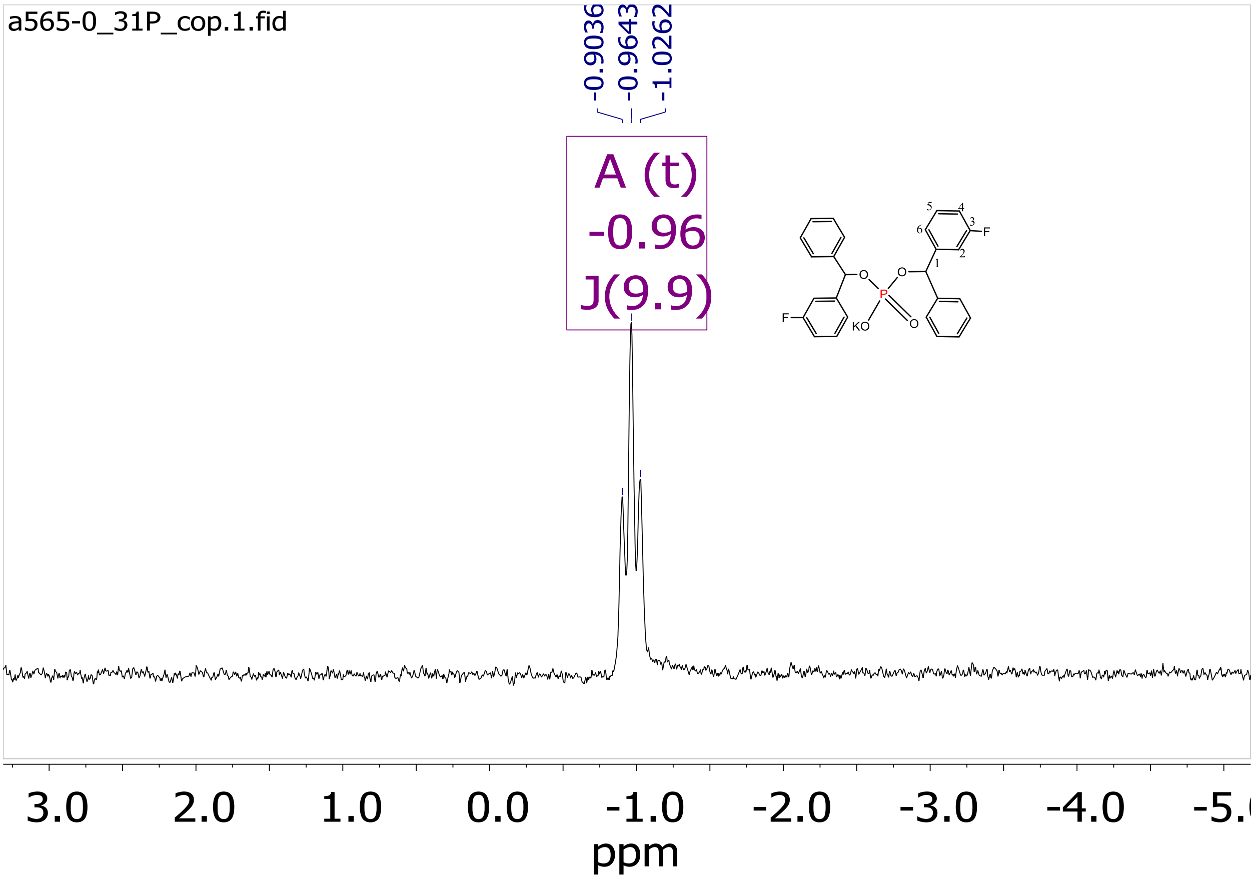

^{31}P NMR spectrum of **2k** (DMSO_d$_6$).

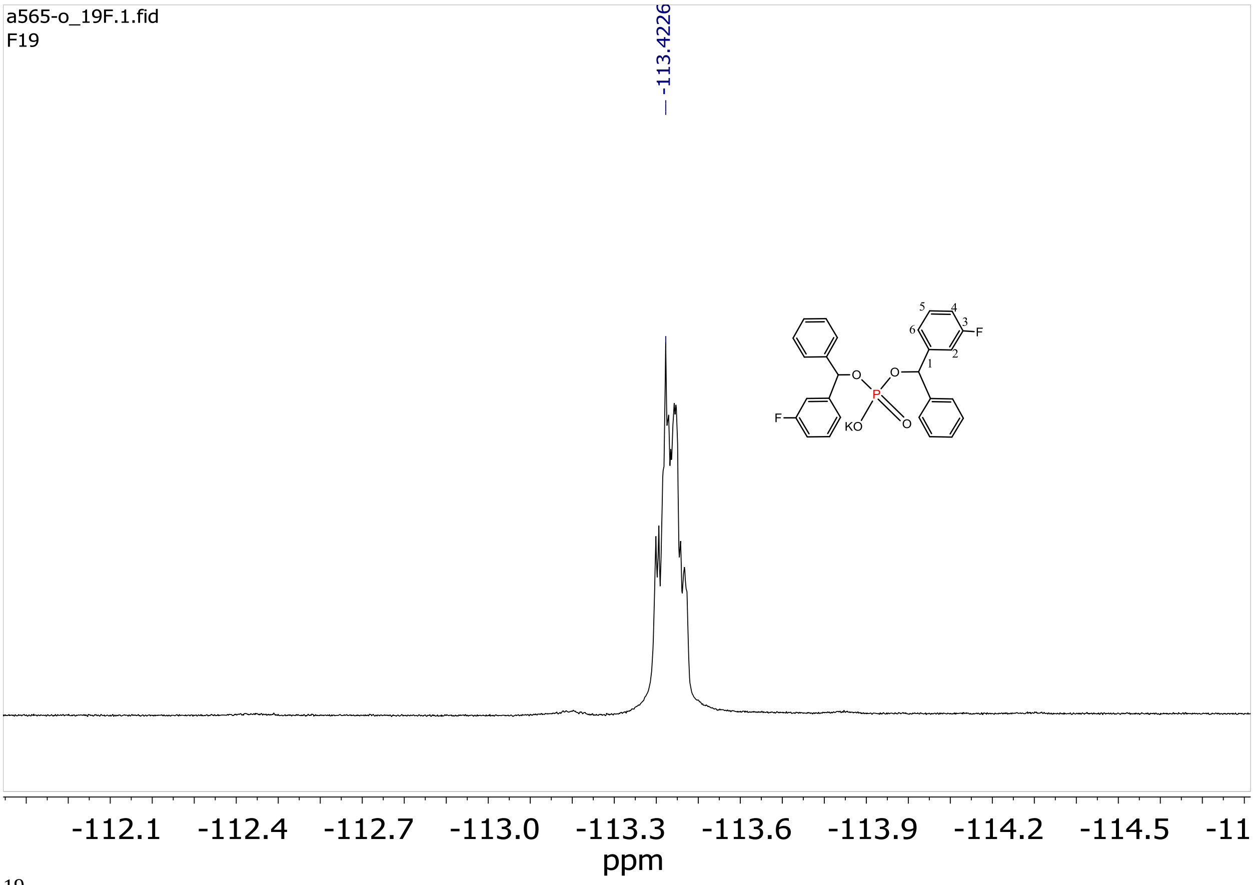

^{19}F NMR spectrum of **2k** (DMSO_d$_6$).

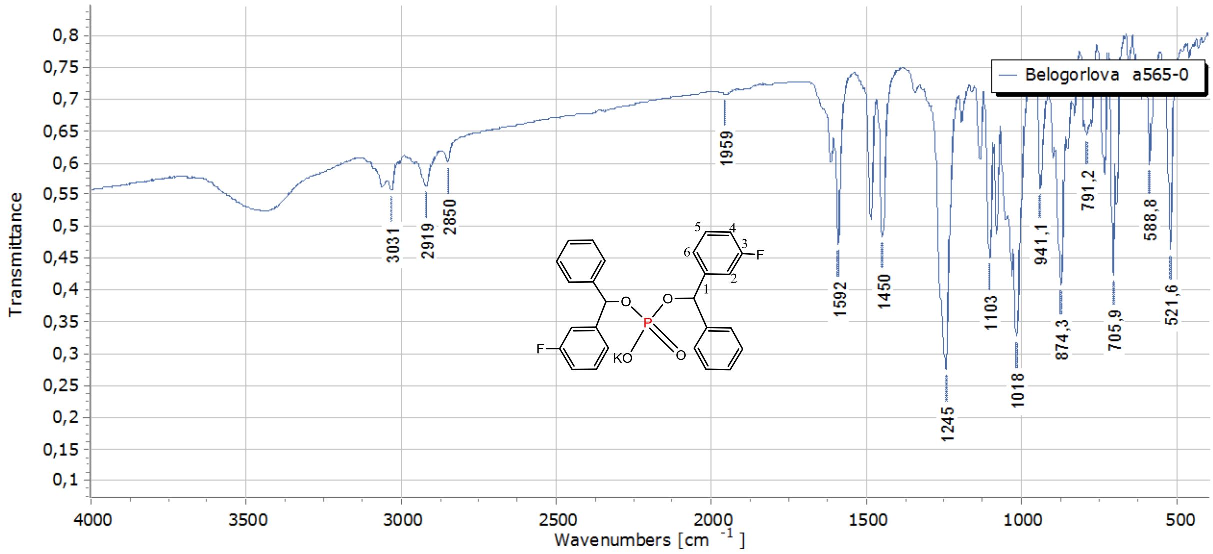

IR spectrum of **2k** (KBr, cm^{-1}).

Potassium bis(naphthalen-1-yl(phenyl)methyl)phosphate (2l)

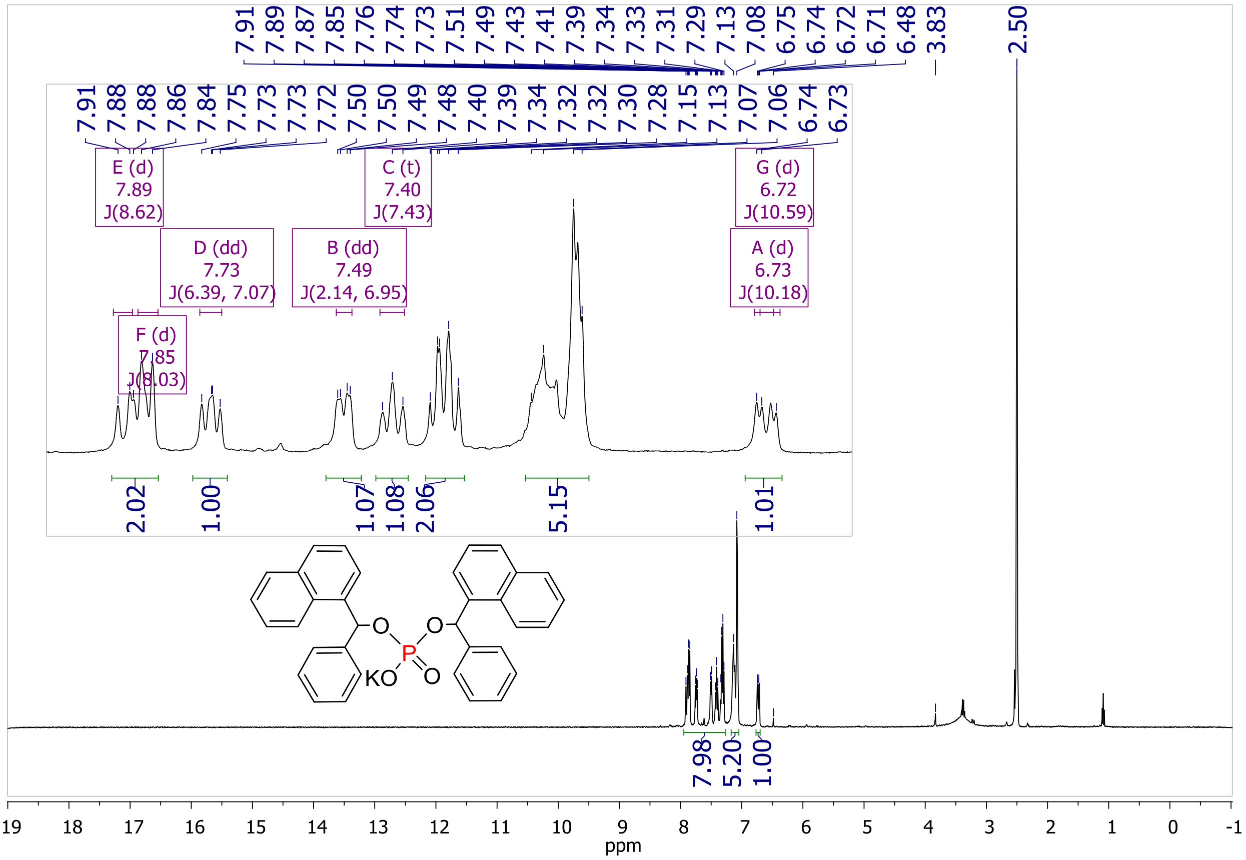

^{1}H NMR spectrum of **2l** (DMSO_d_6).

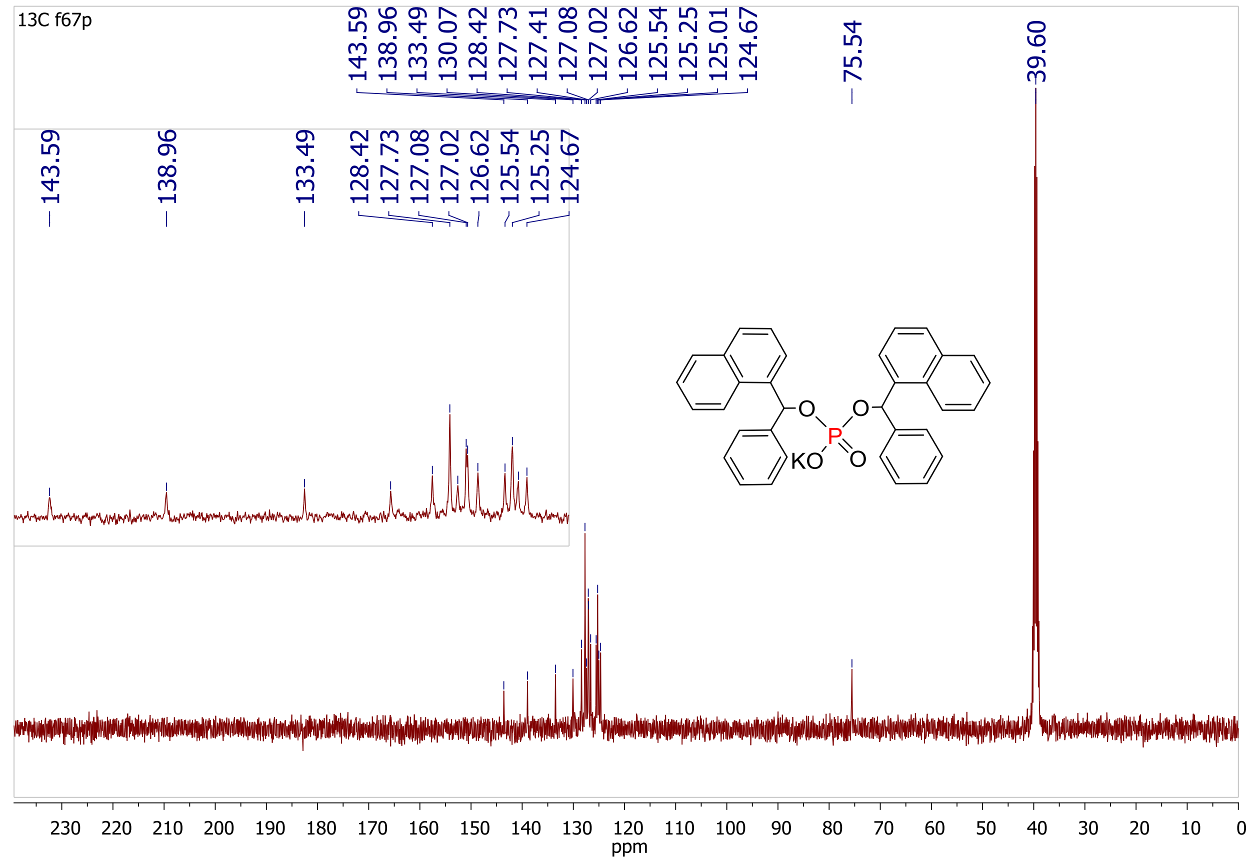

^{13}C NMR spectrum of **2l** (DMSO_d$_6$).

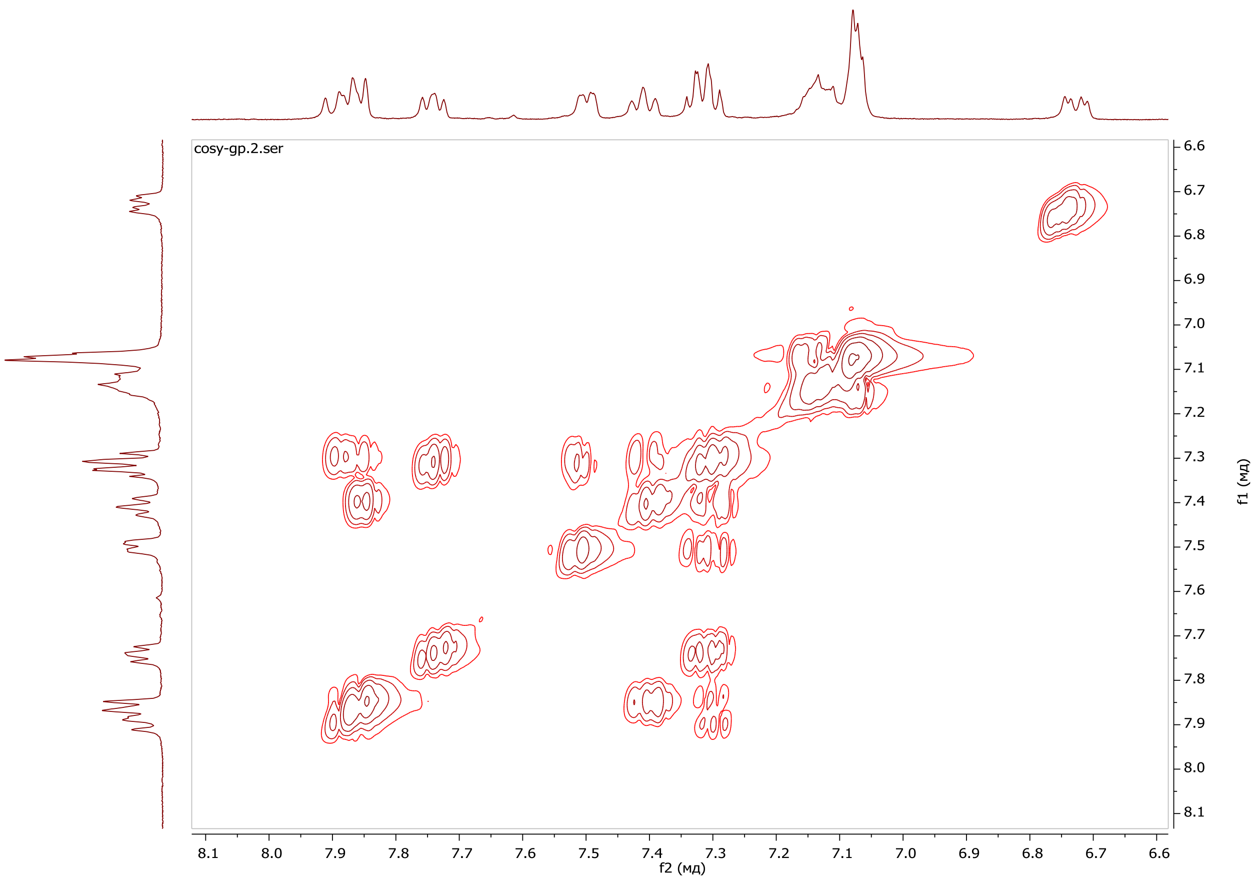

2D COSY ^{1}H-^{1}H spectrum of **2l** (DMSO_d$_6$).

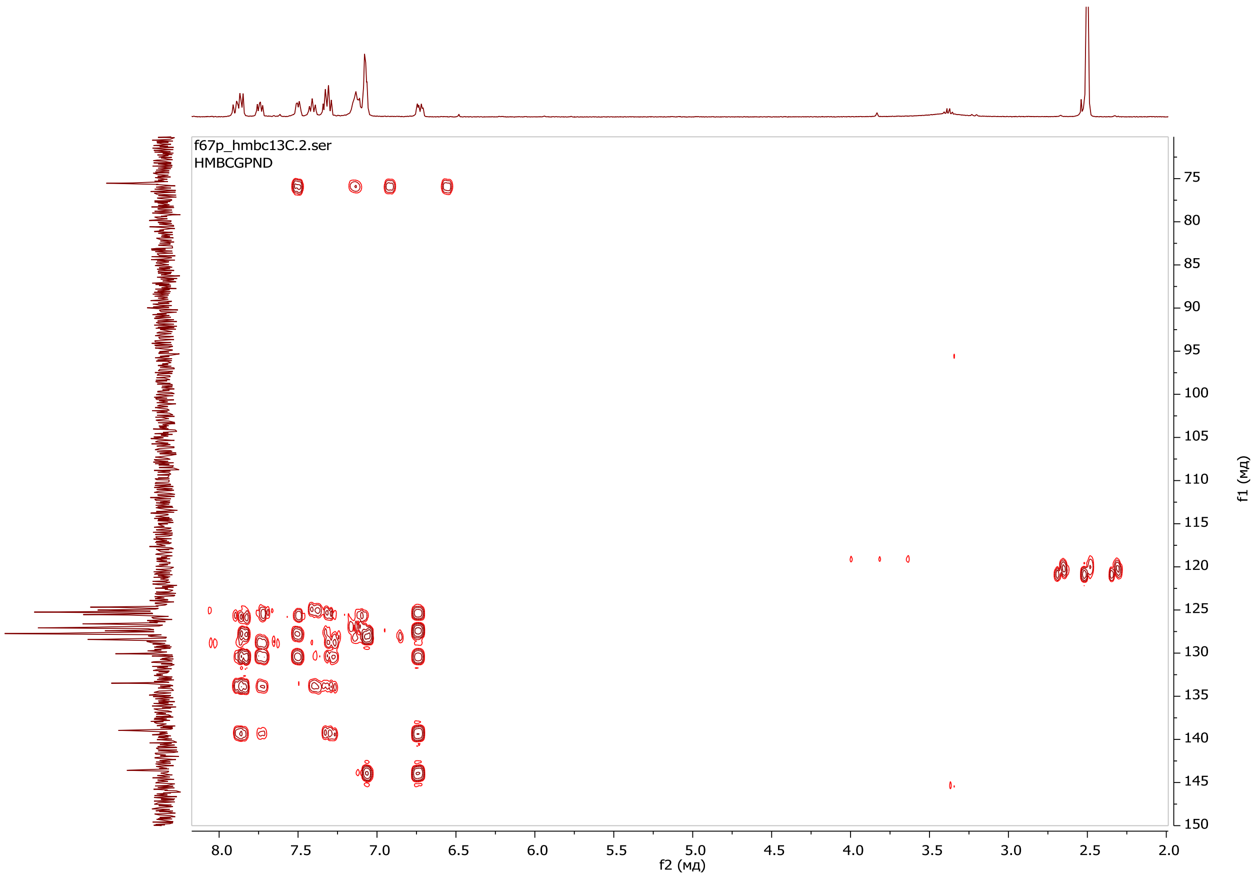

2D HMBC ^{13}C-^{1}H NMR spectrum of **2l** (DMSO_d_6).

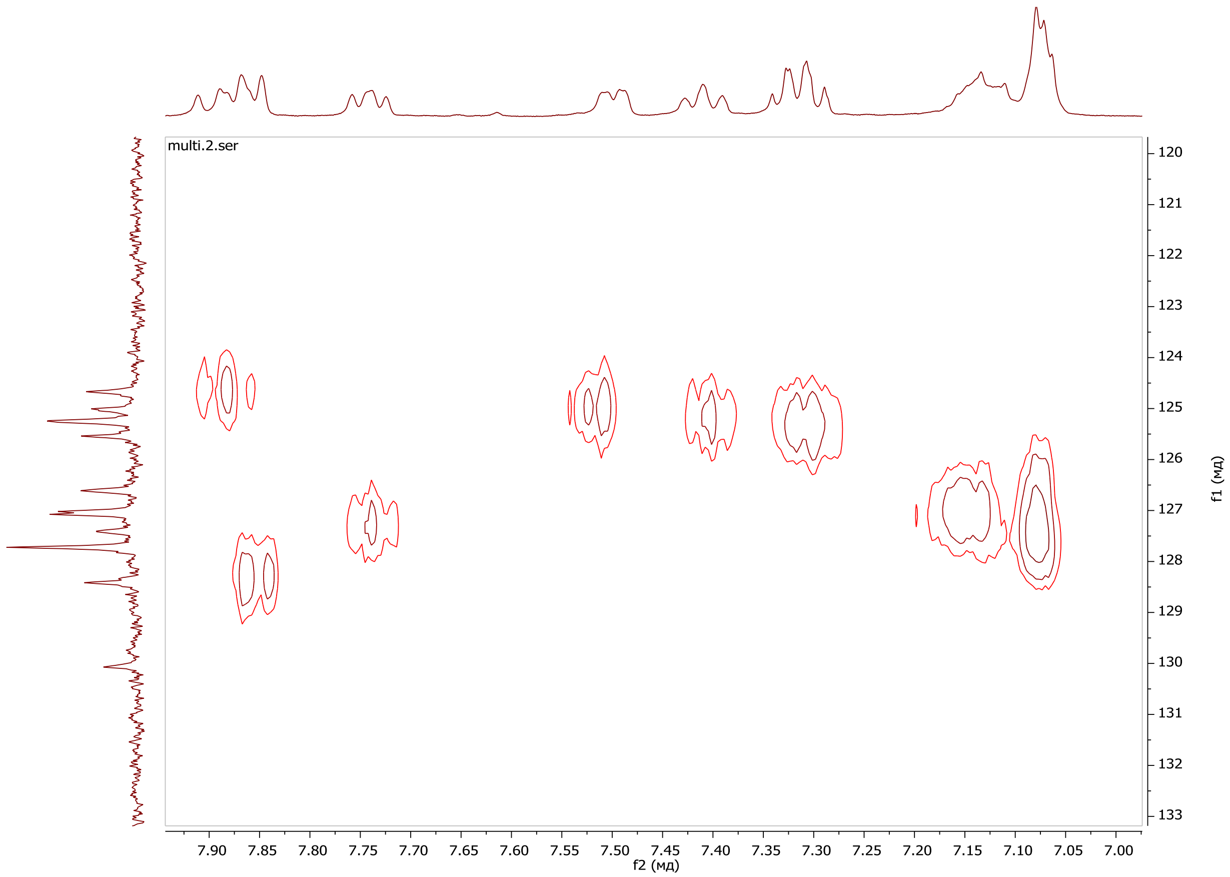

2D HSQC ^{13}C-^{1}H NMR spectrum of **2l** (DMSO_d_6).

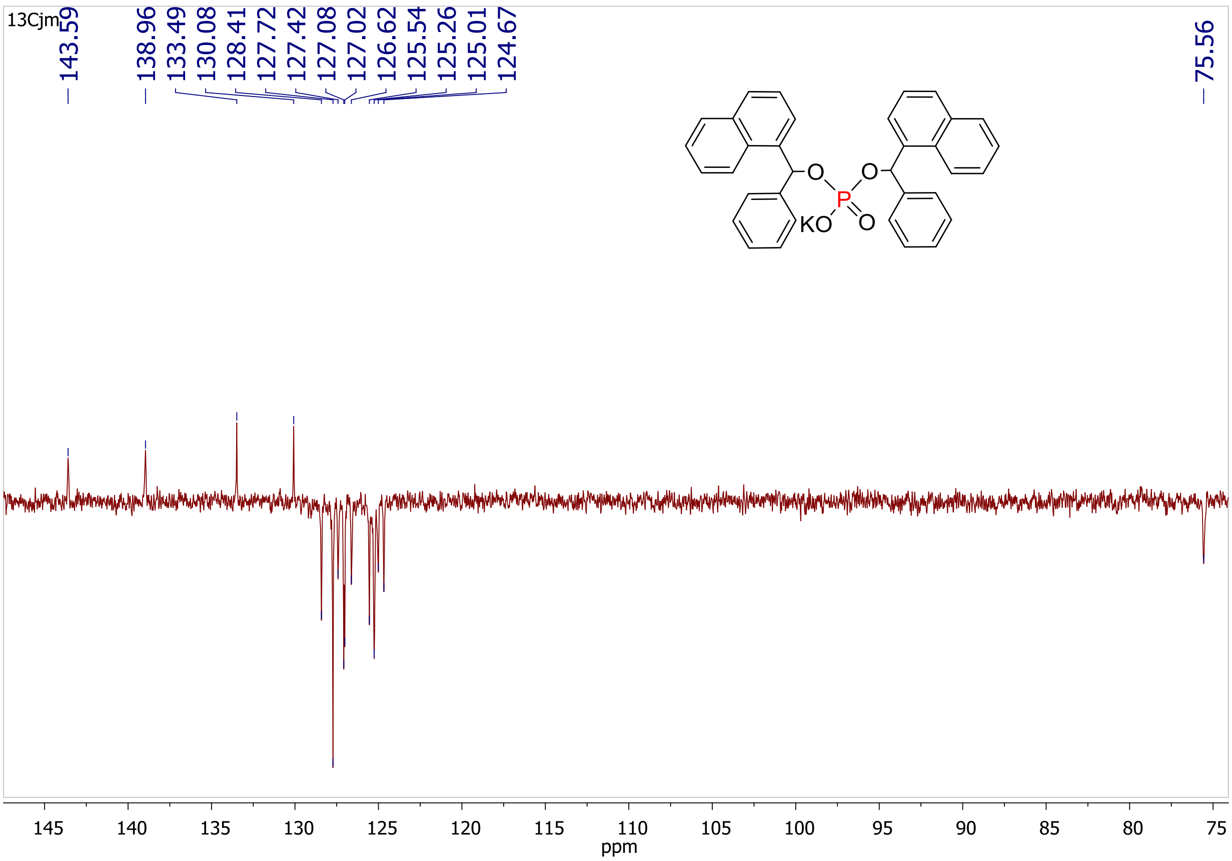

^{13}Cjm NMR spectrum of **2l** (DMSO_d$_6$).

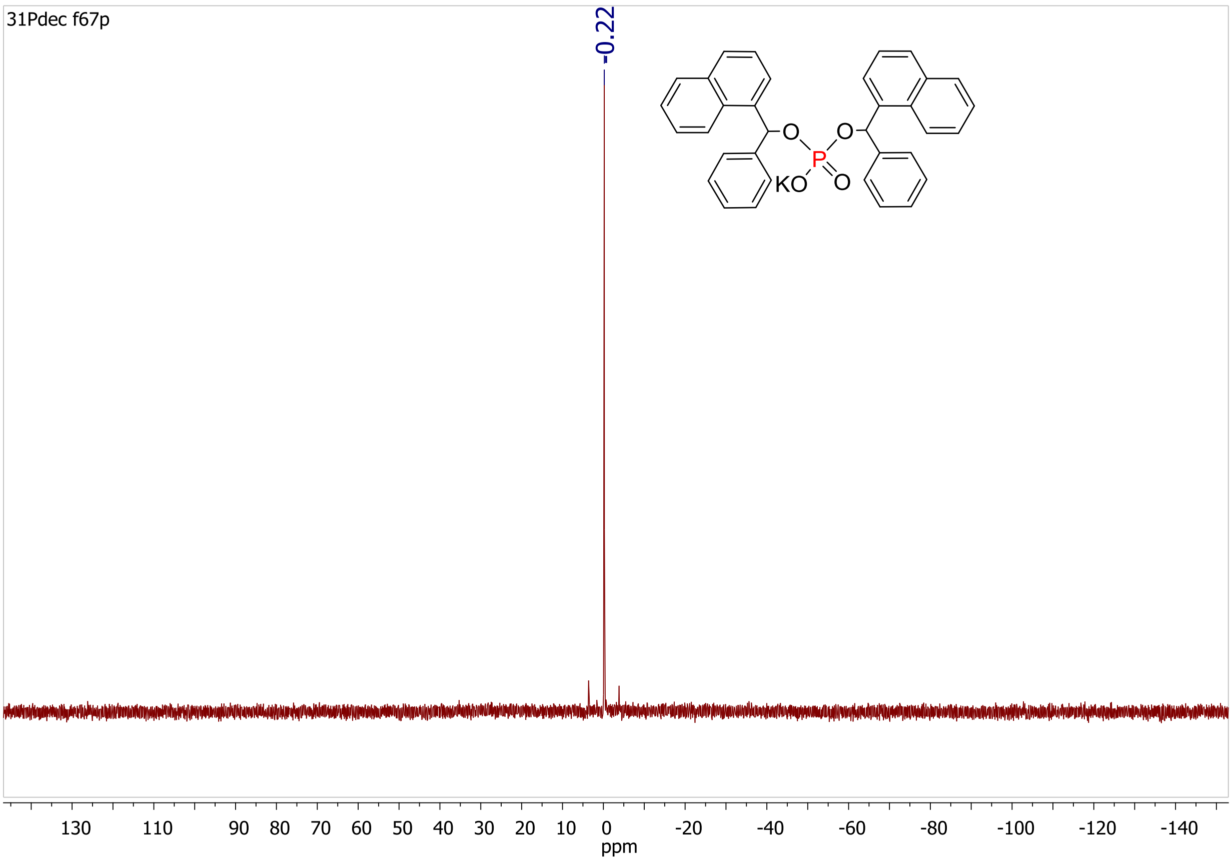

^{31}P{^{1}H} NMR spectrum of **2l** (DMSO_d$_6$).

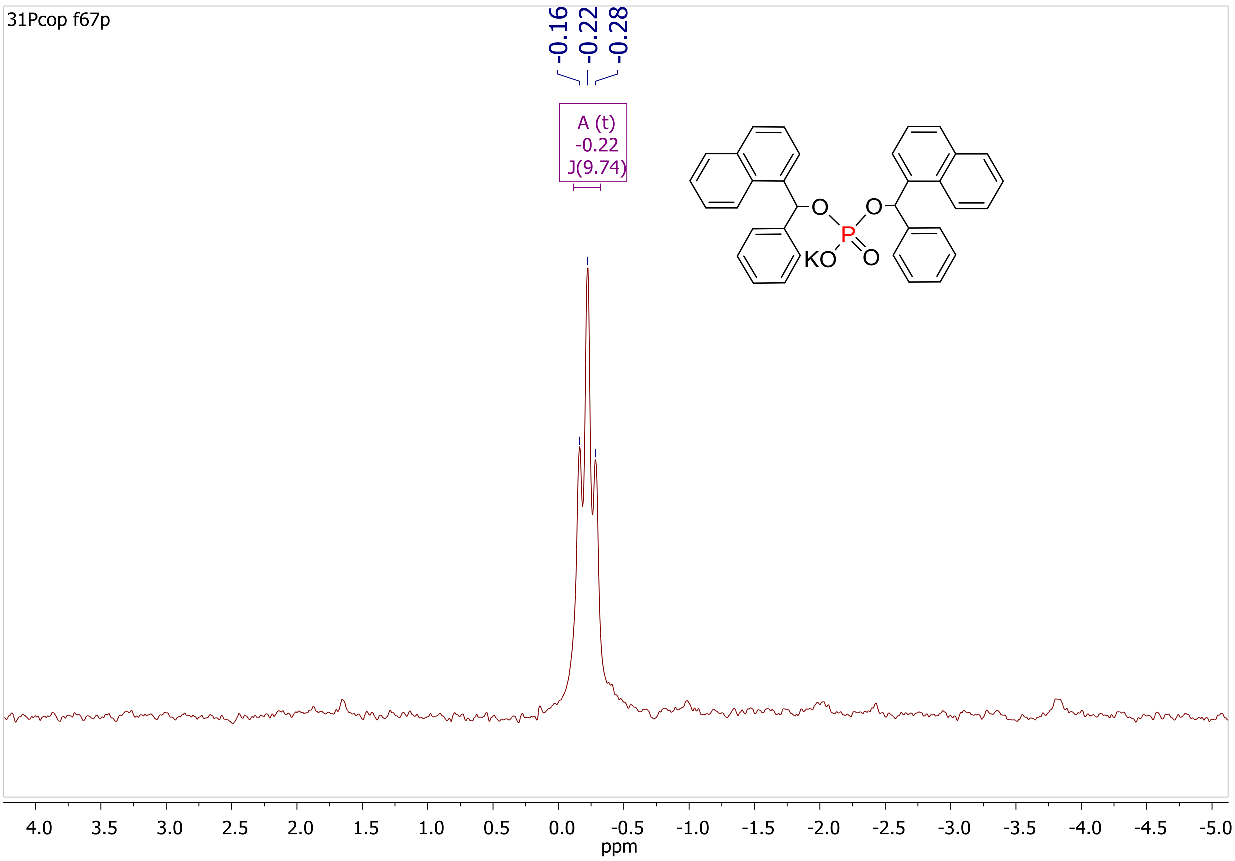

^{31}P NMR spectrum of **2l** (DMSO_d$_6$).

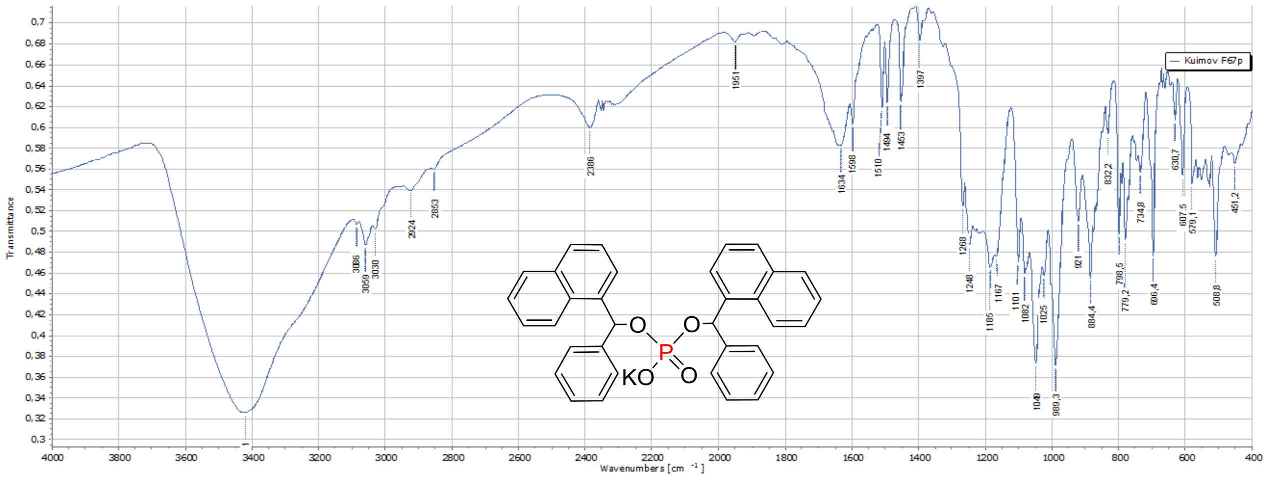

IR spectrum of **2l** (KBr, cm^{-1}).

Potassium bis[phenyl(pyridin-4-yl)methyl]phosphate (2m)

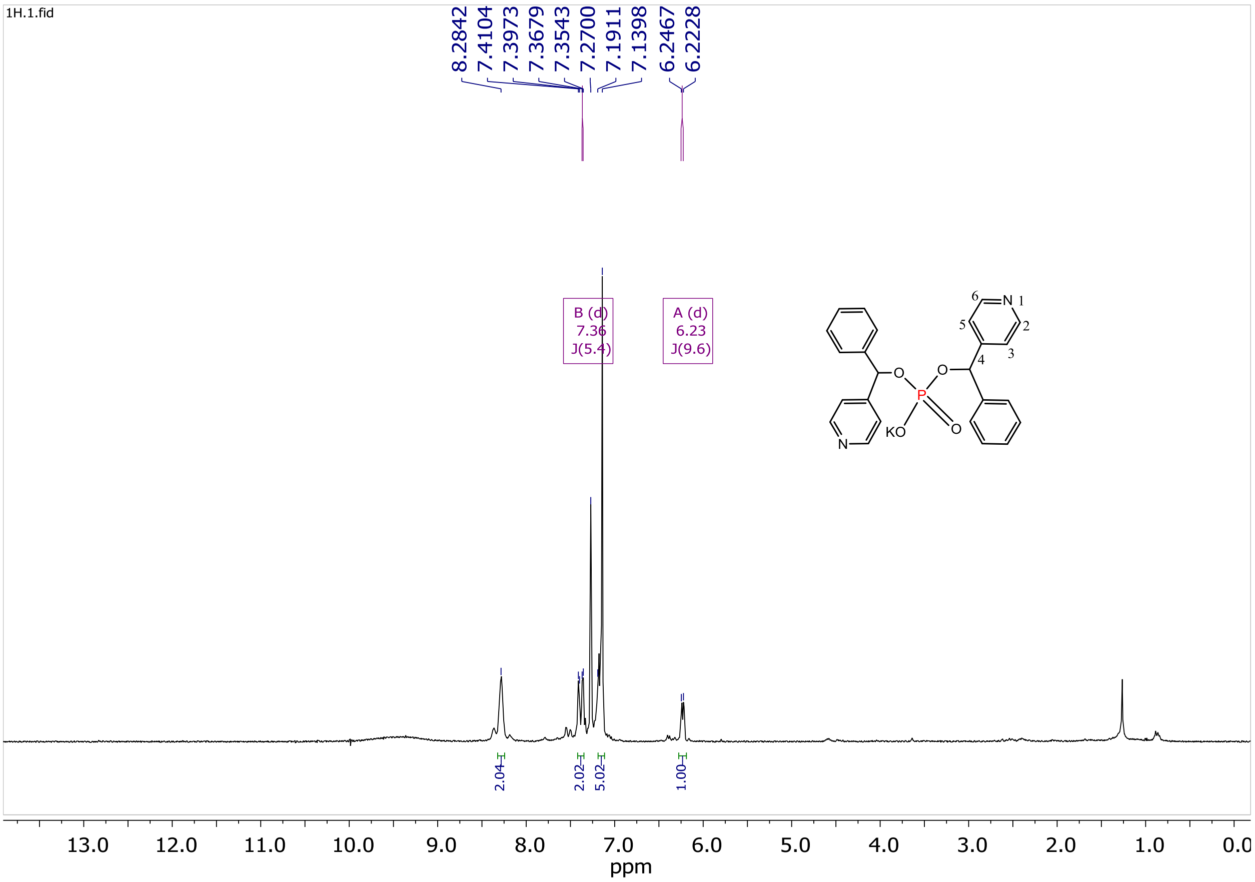

^{1}H NMR spectrum of **2m** ($CDCl_3$).

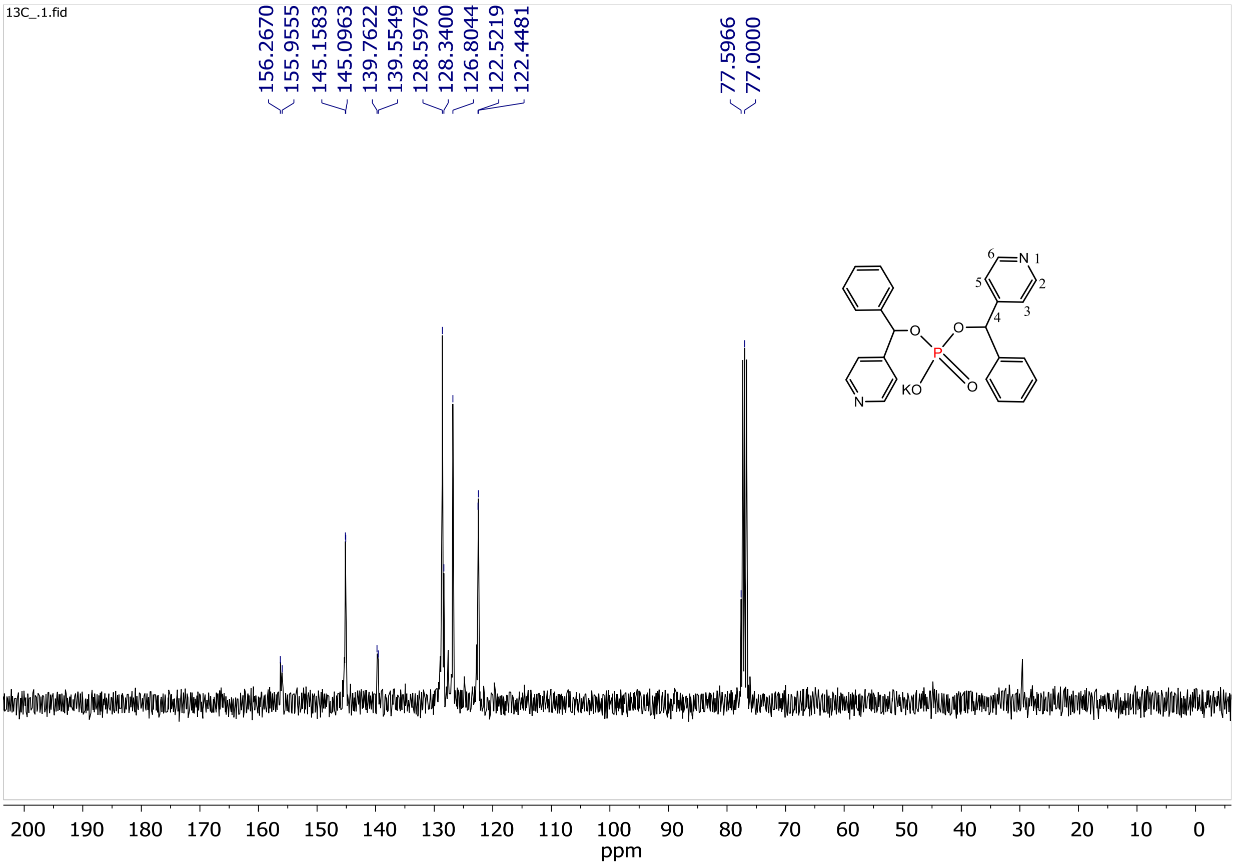

^{13}C NMR spectrum of **2m** ($CDCl_3$).

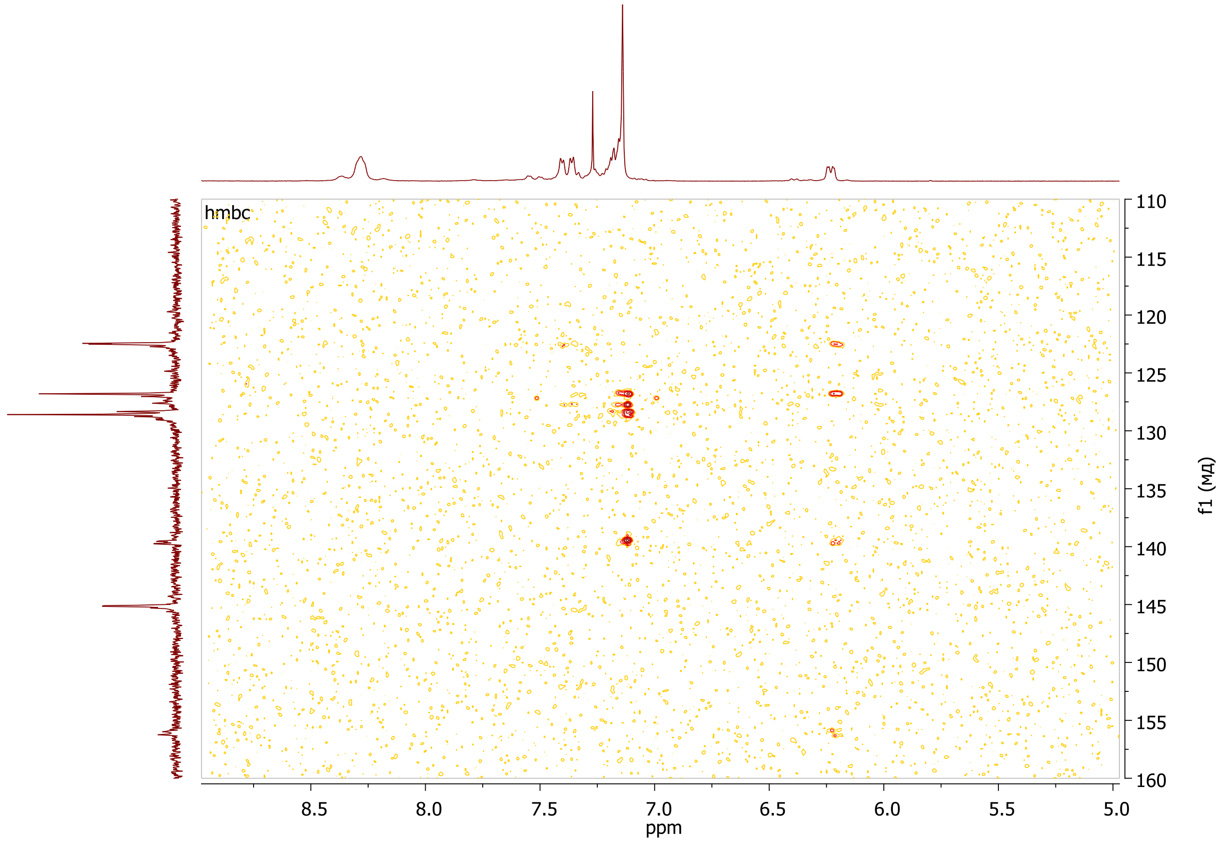

2D HMBC ^{13}C-^{1}H NMR spectrum of **2m** (DMSO_d$_6$).

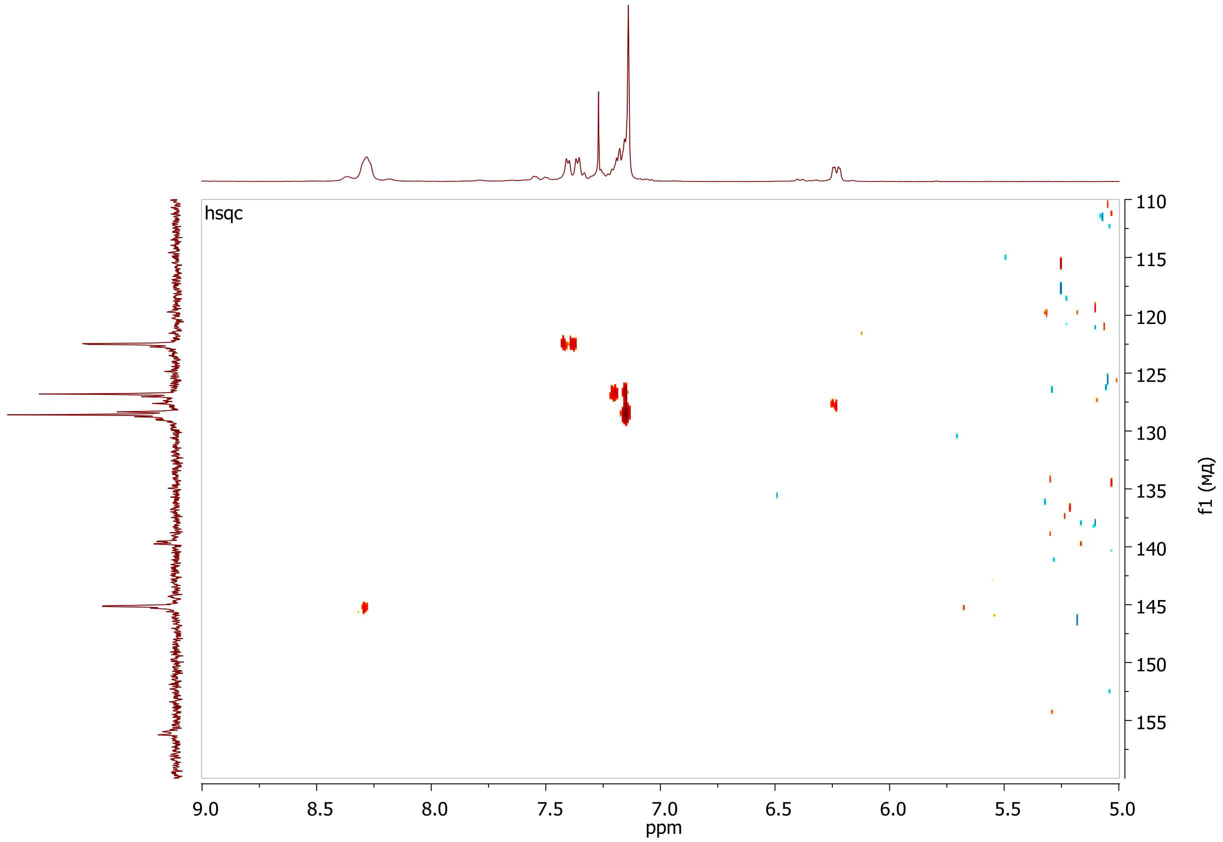

2D HSQC ^{13}C-^{1}H NMR spectrum of **2m** (DMSO_d$_6$).

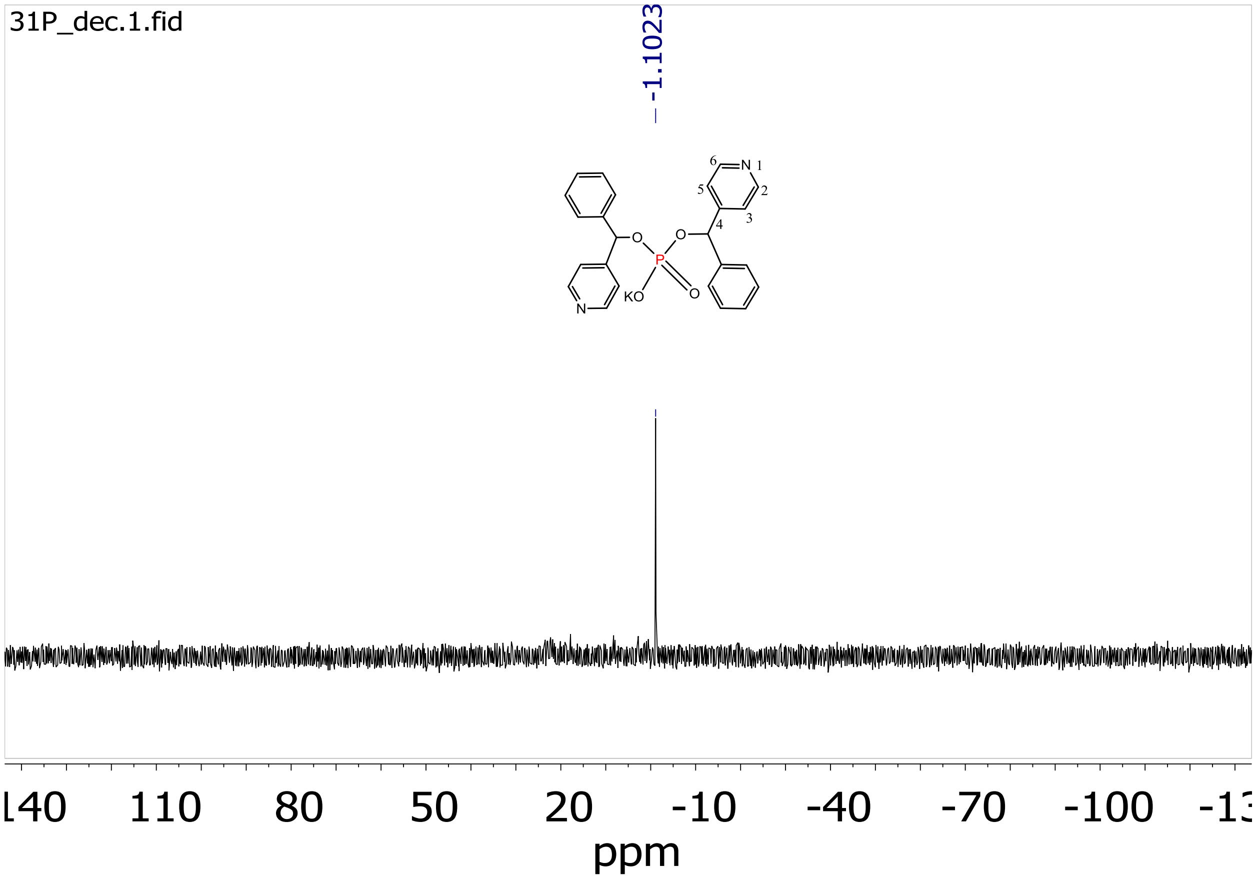

$^{31}P\{^{1}H\}$ NMR spectrum of **2m** ($CDCl_3$).

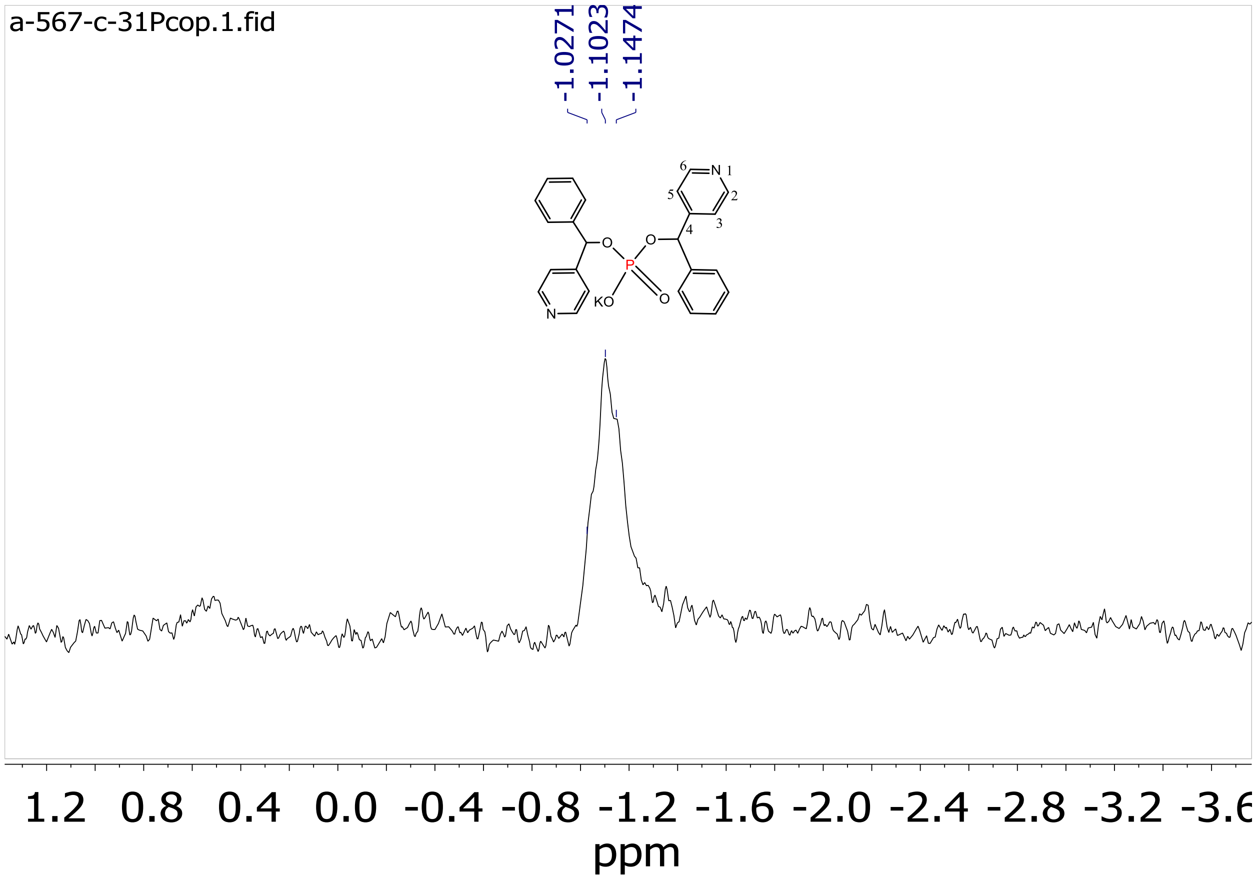

^{31}P NMR spectrum of **2m** ($CDCl_3$).

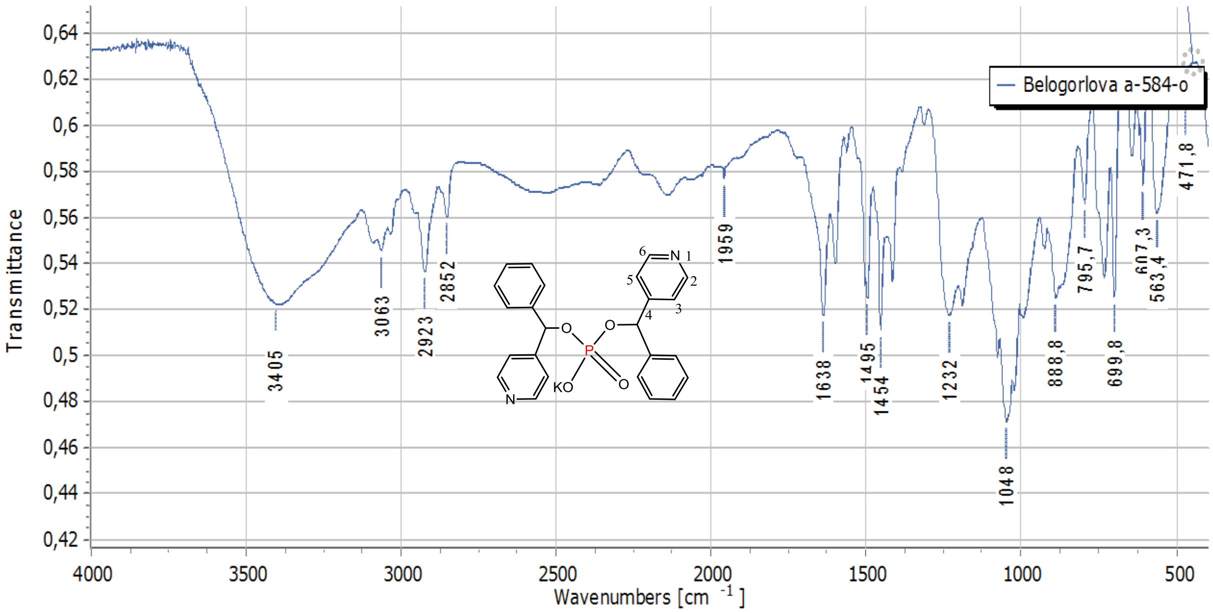

IR spectrum of **2m** (KBr, cm^{-1}).

Potassium bis[phenyl(pyridin-3-yl)methyl]phosphate (2n)

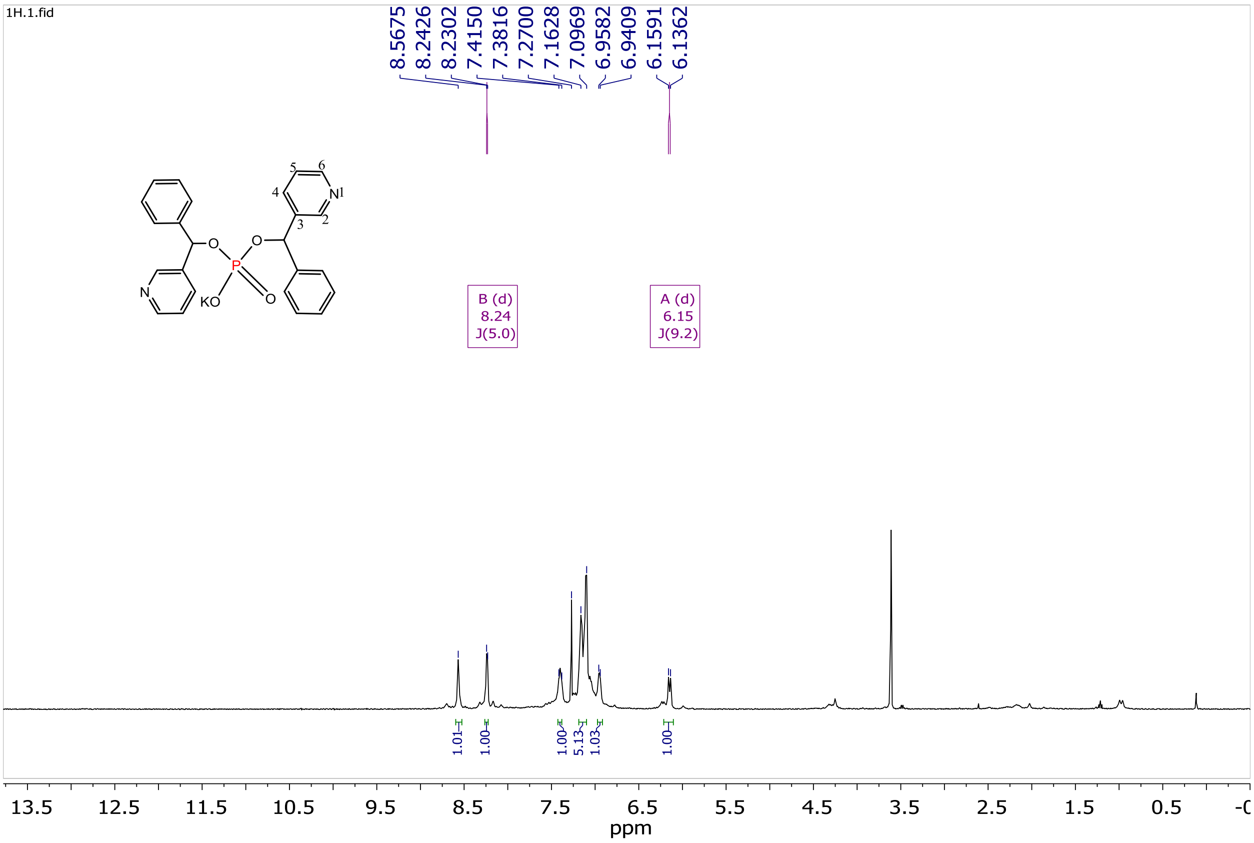

^{1}H NMR spectrum of **2n** ($CDCl_3$).

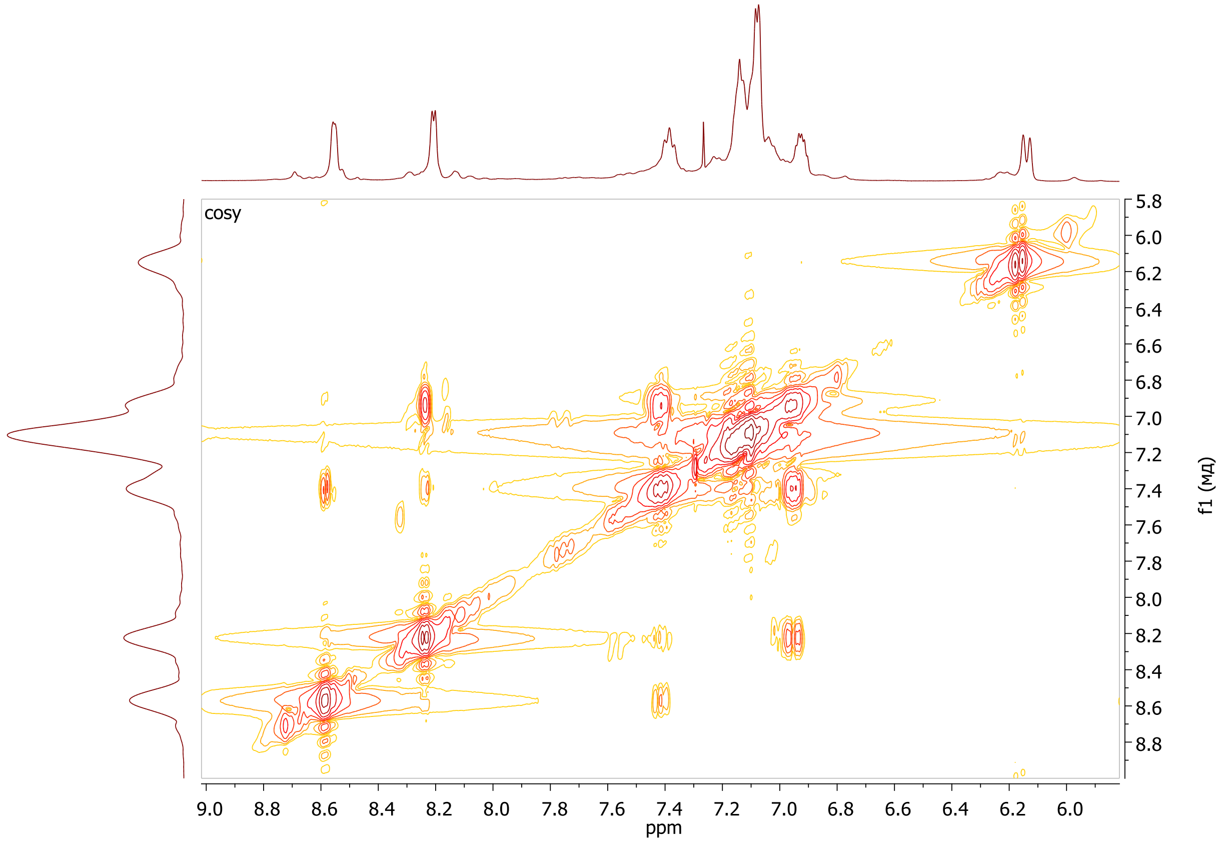

2D COSY ^1H-^1H spectrum of **2n** ($CDCl_3$).

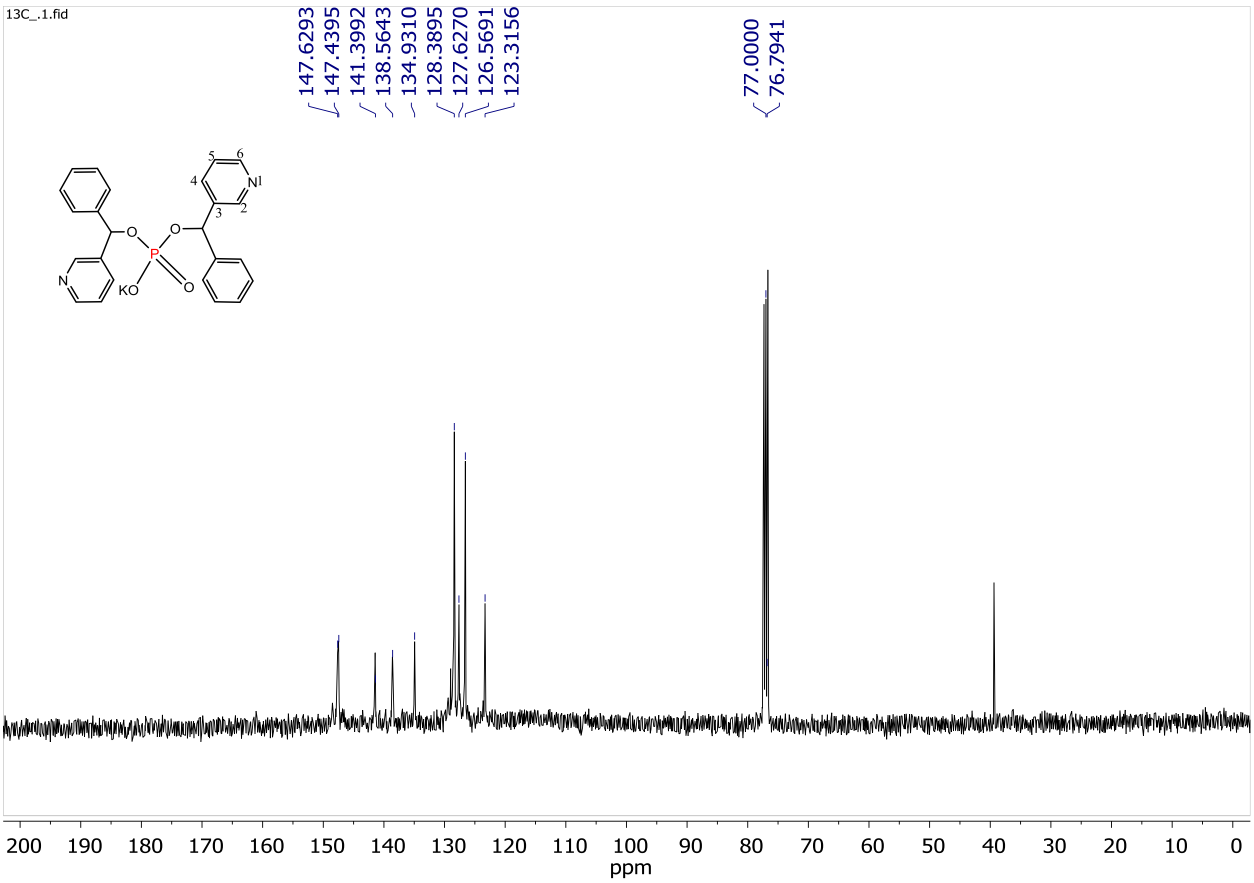

^{13}C NMR spectrum of **2n** ($CDCl_3$).

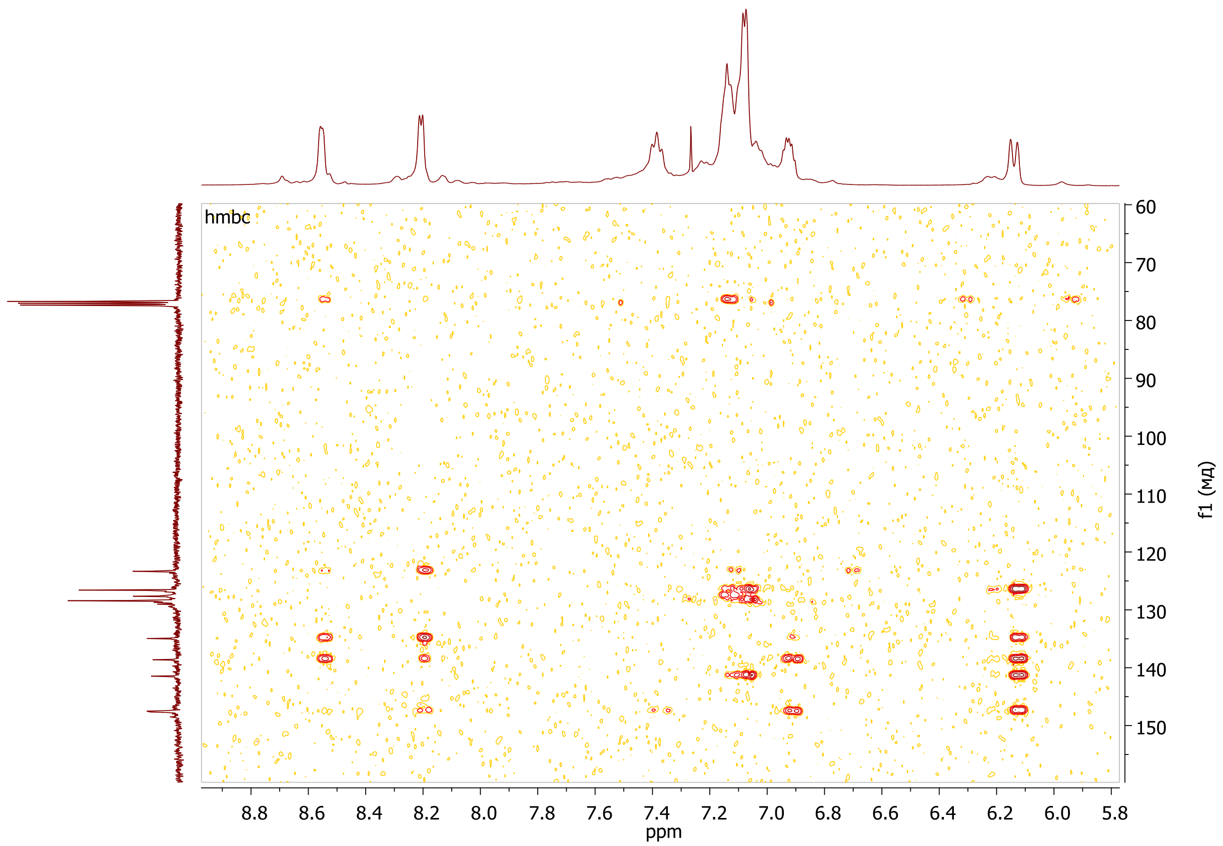

2D HMBC ^{13}C-^{1}H NMR spectrum of **2n** ($CDCl_3$).

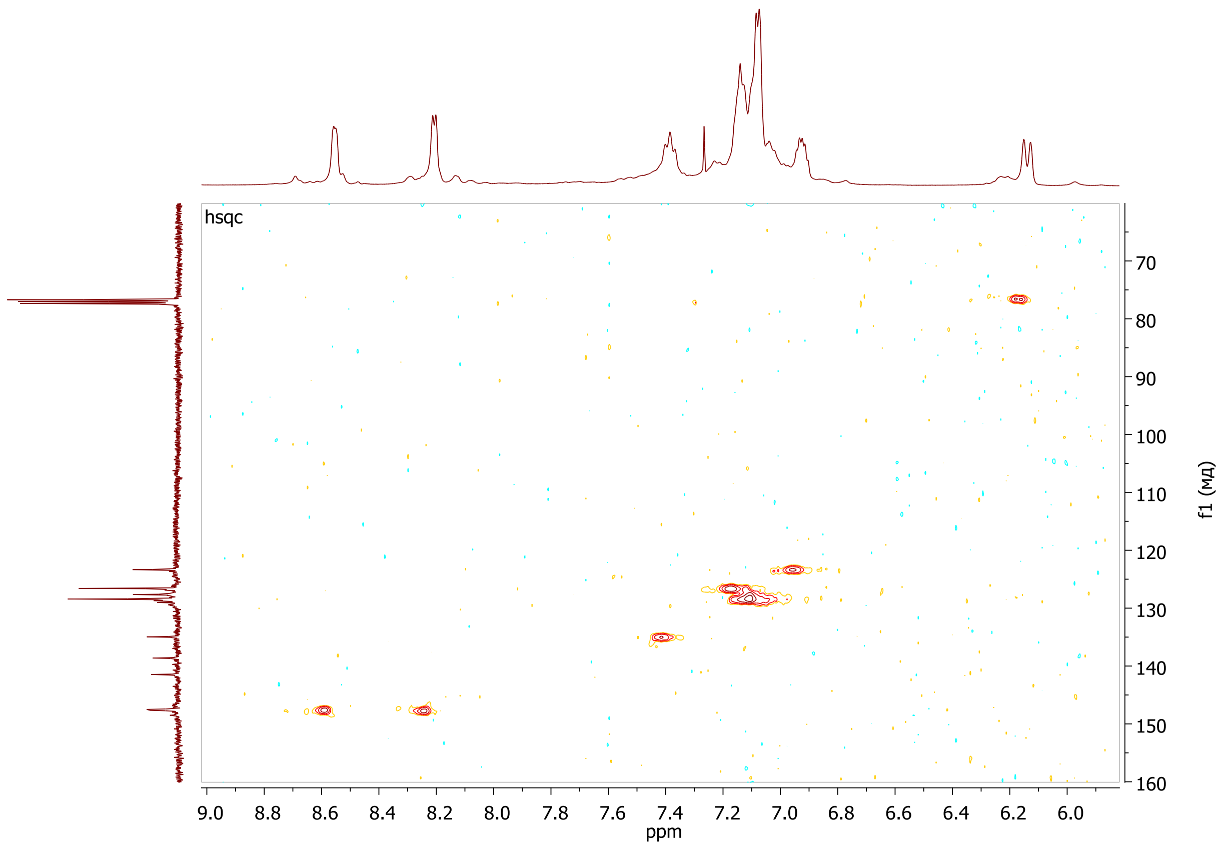

2D HSQC ^{13}C-^{1}H NMR spectrum of **2n** ($CDCl_3$).

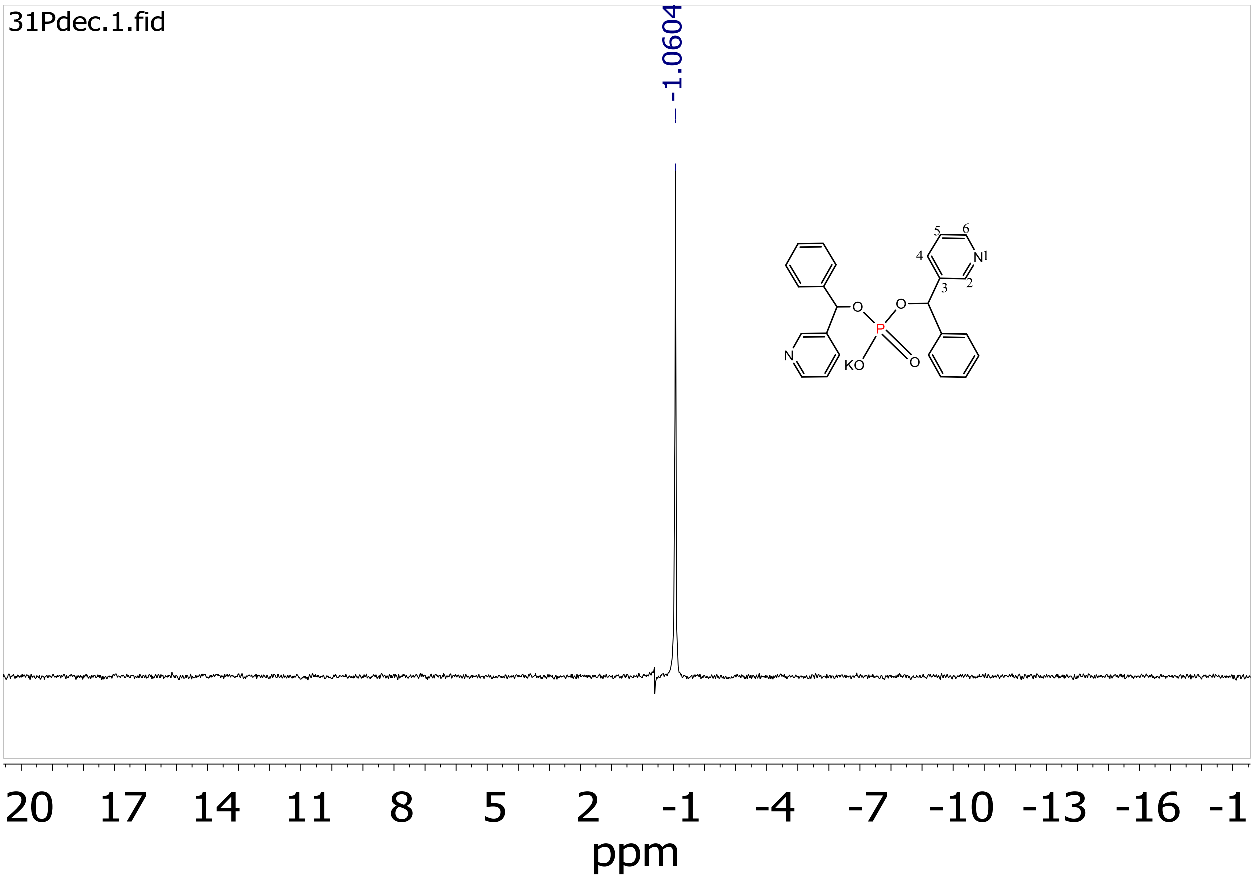

$^{31}P\{^{1}H\}$ NMR spectrum of **2n** ($CDCl_3$).

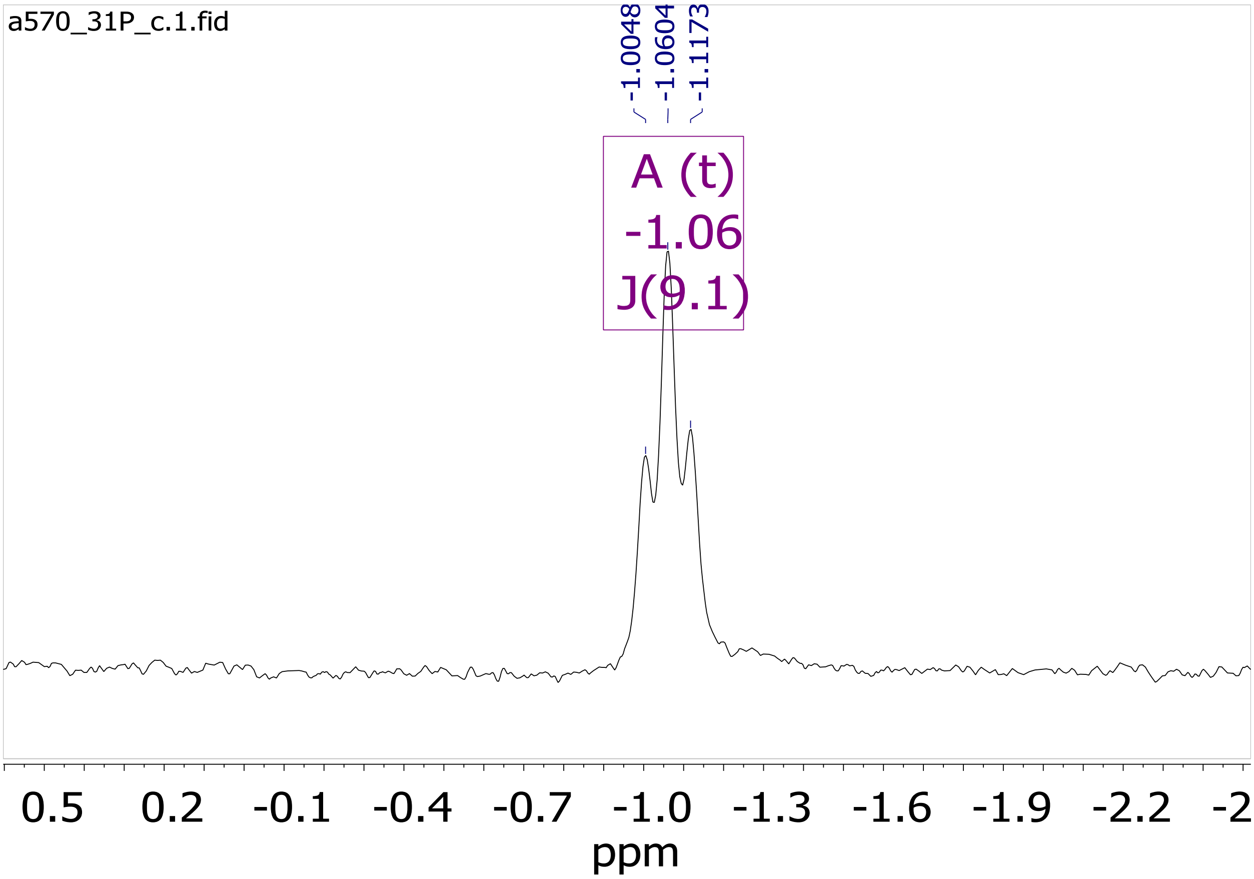

^{31}P NMR spectrum of **2n** ($CDCl_3$).

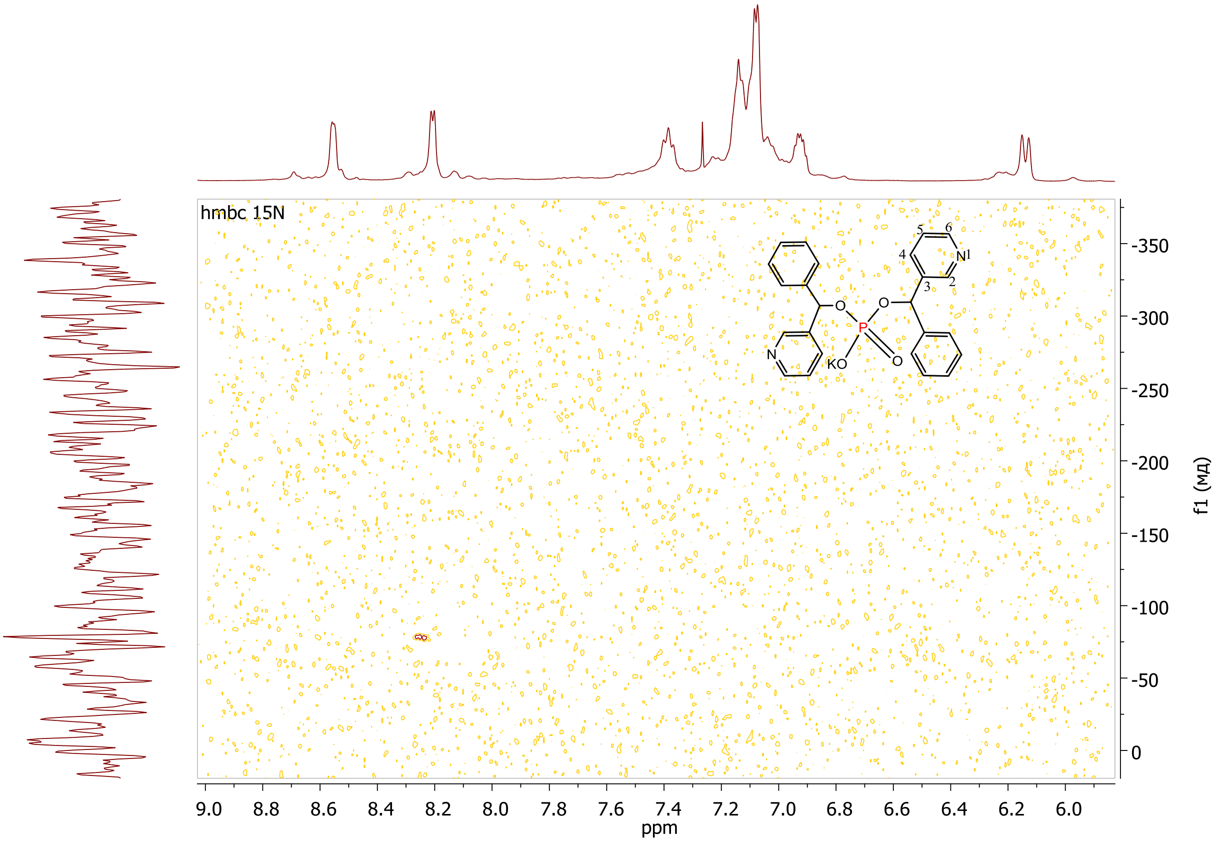

2D HMBC ^{15}N-^{1}H NMR spectrum of **2n** ($CDCl_3$).

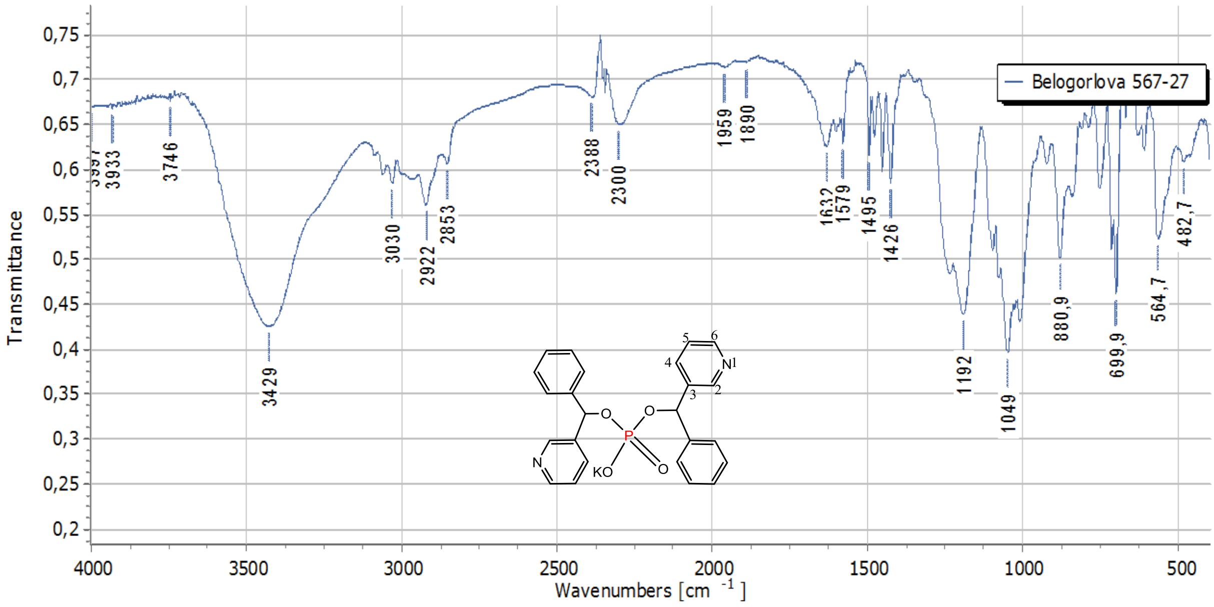

IR spectrum of **2n** (KBr, cm^{-1}).

Potassium bis[phenyl(2-thienyl)methyl]phosphate (2l).

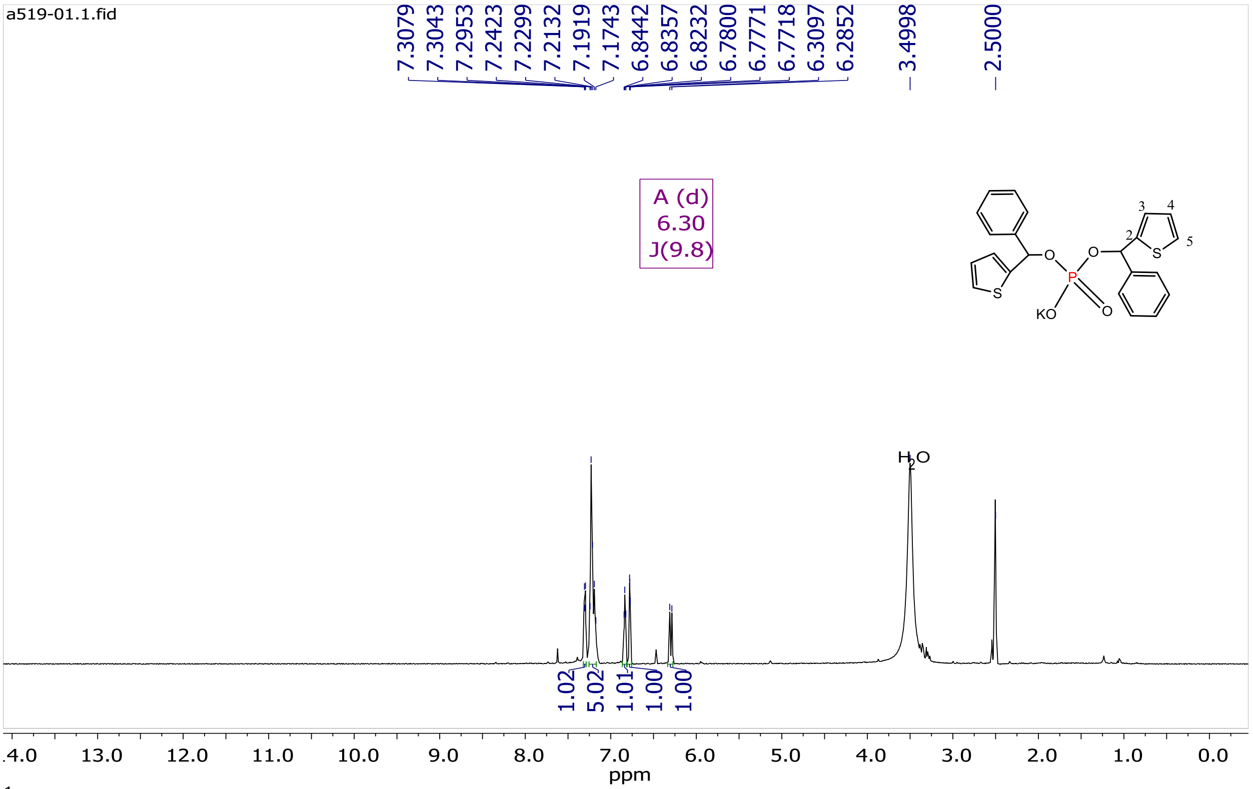

^{1}H NMR spectrum of **2o** (DMSO_d$_6$).

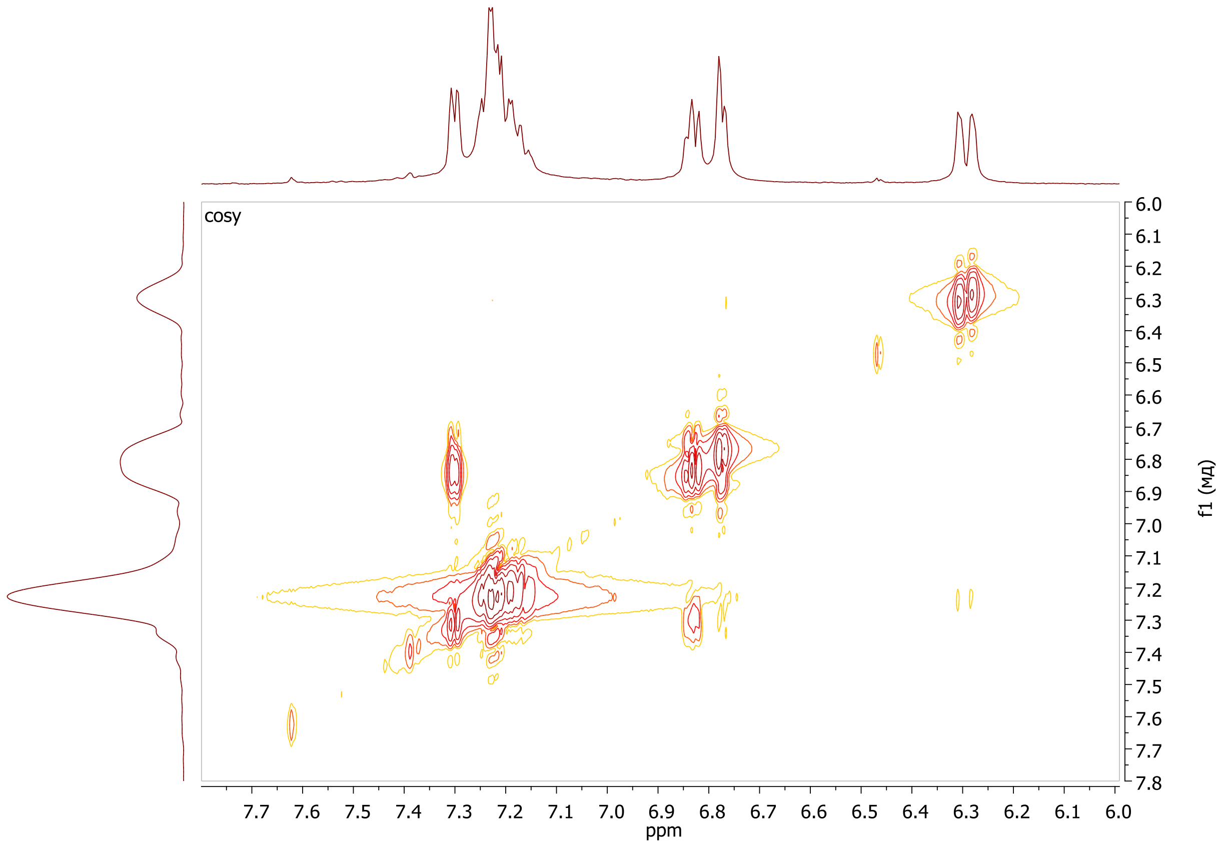

2D COSY spectrum of **2o** (DMSO_d$_6$).

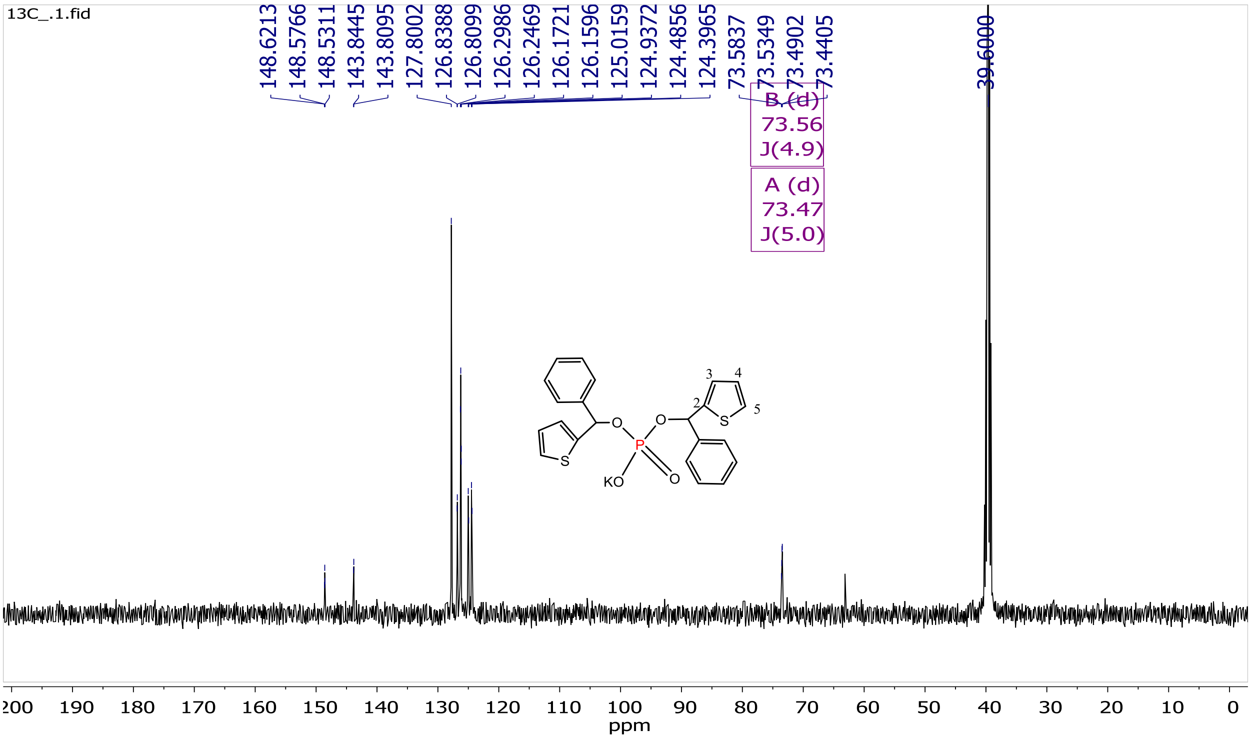

^{13}C NMR spectrum of **2o** (DMSO_d_6).

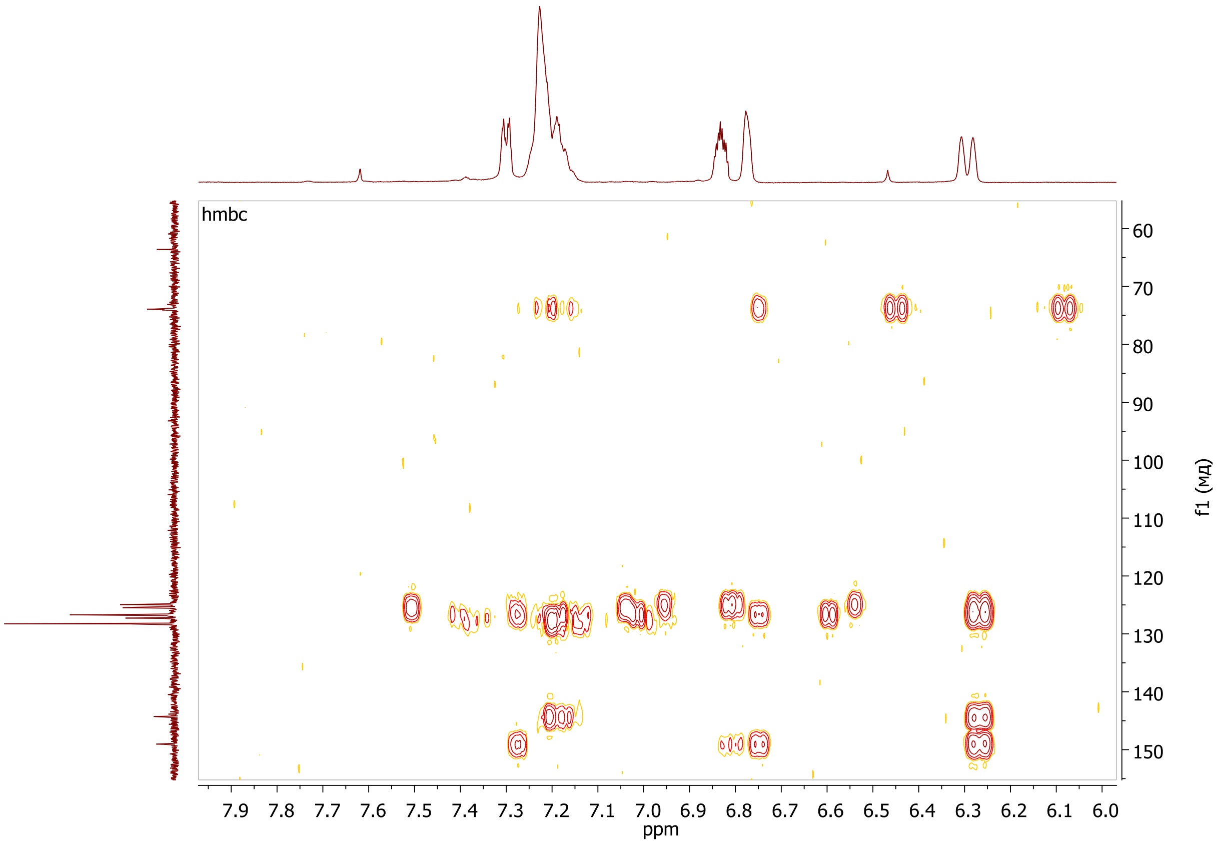

2D HMBC ^{13}C-^{1}H NMR spectrum of **2o** (DMSO_d_6).

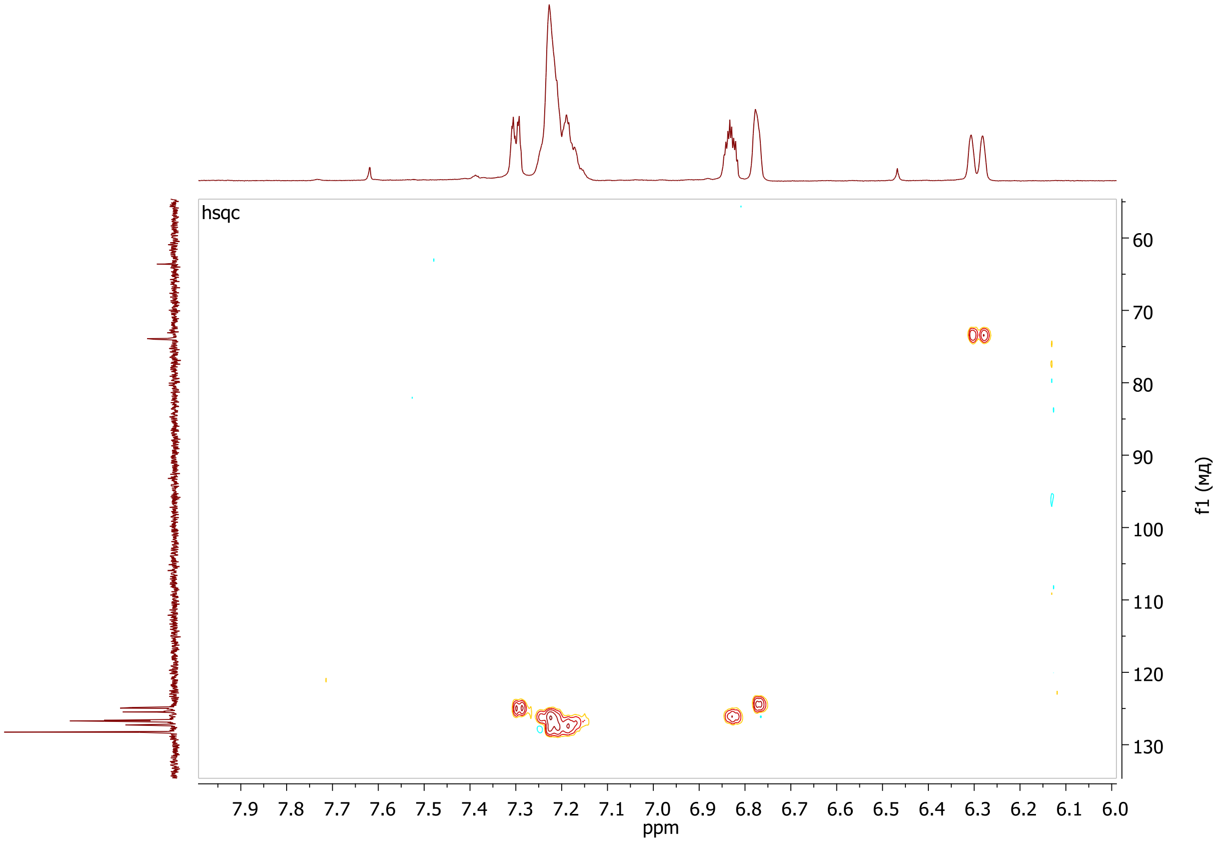

2D HSQC ^{13}C-^{1}H NMR spectrum of **2o** (DMSO_d$_6$).

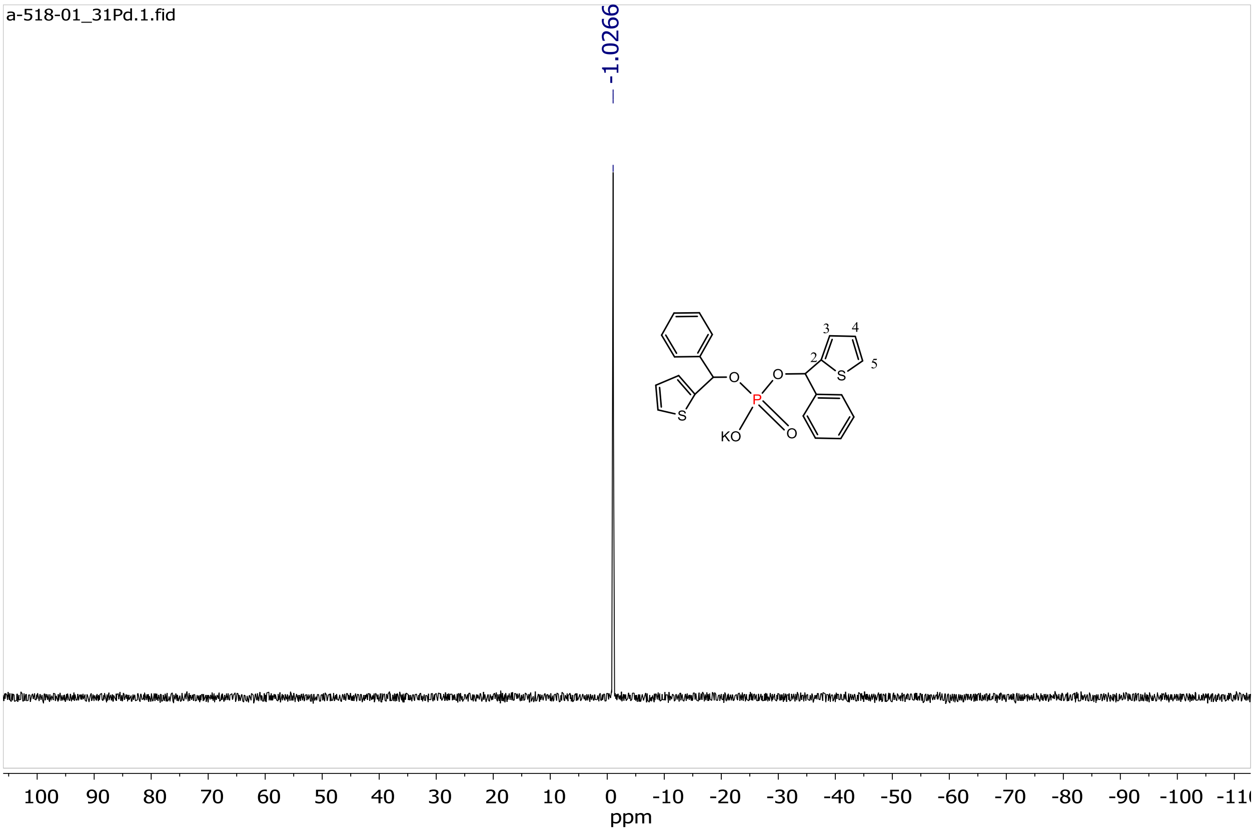

$^{31}P\{^{1}H\}$ NMR spectrum of **2o** (DMSO_d$_6$).

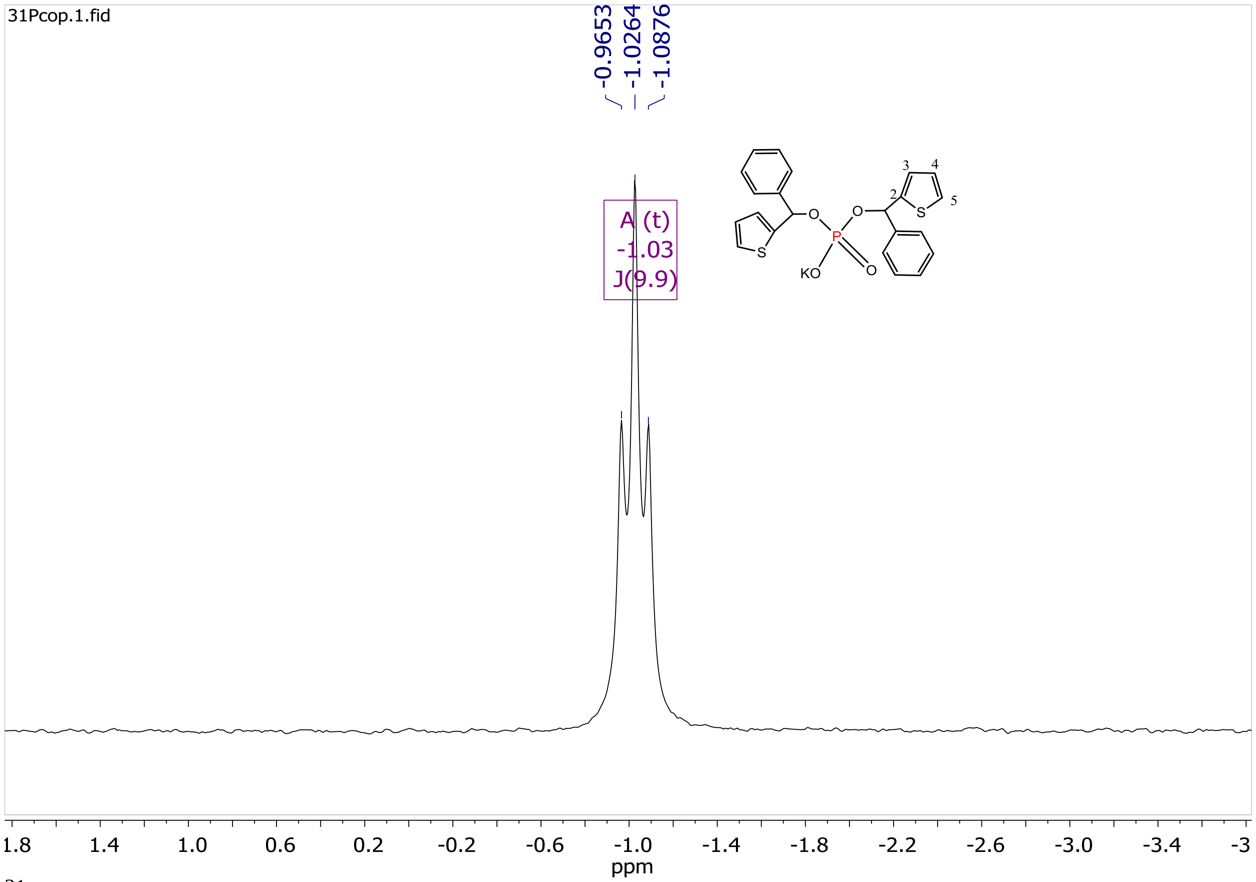

^{31}P NMR spectrum of **2o** (DMSO_d$_6$).

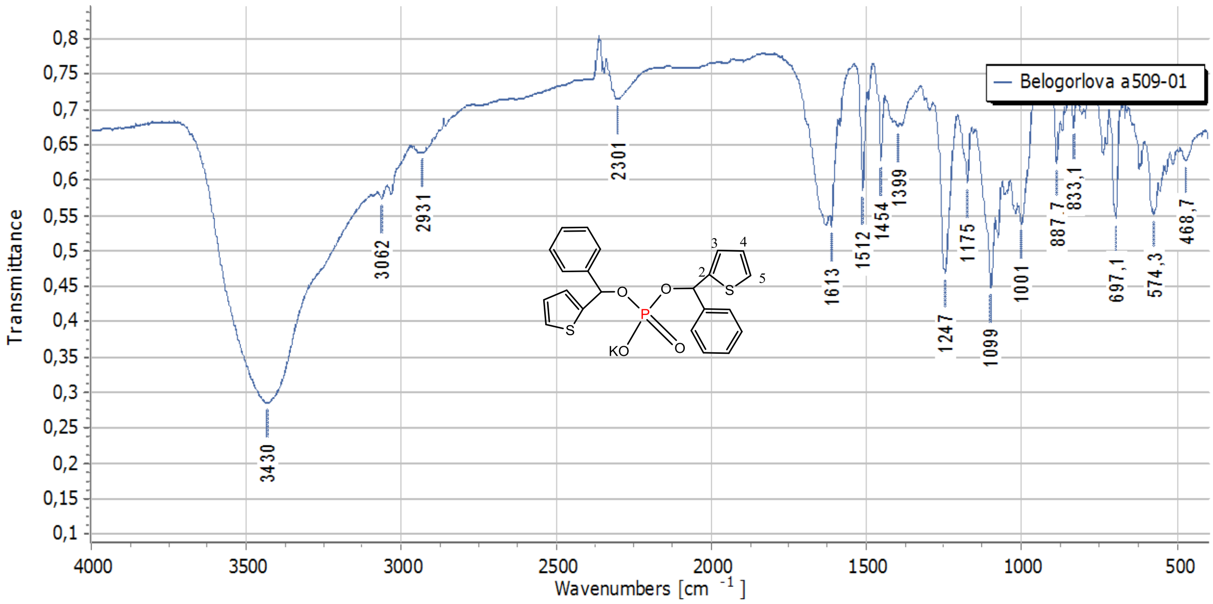

IR spectrum of **2o** (KBr, cm^{-1}).

Furan-2-yl(phenyl)methyl hypophosphite (2p)

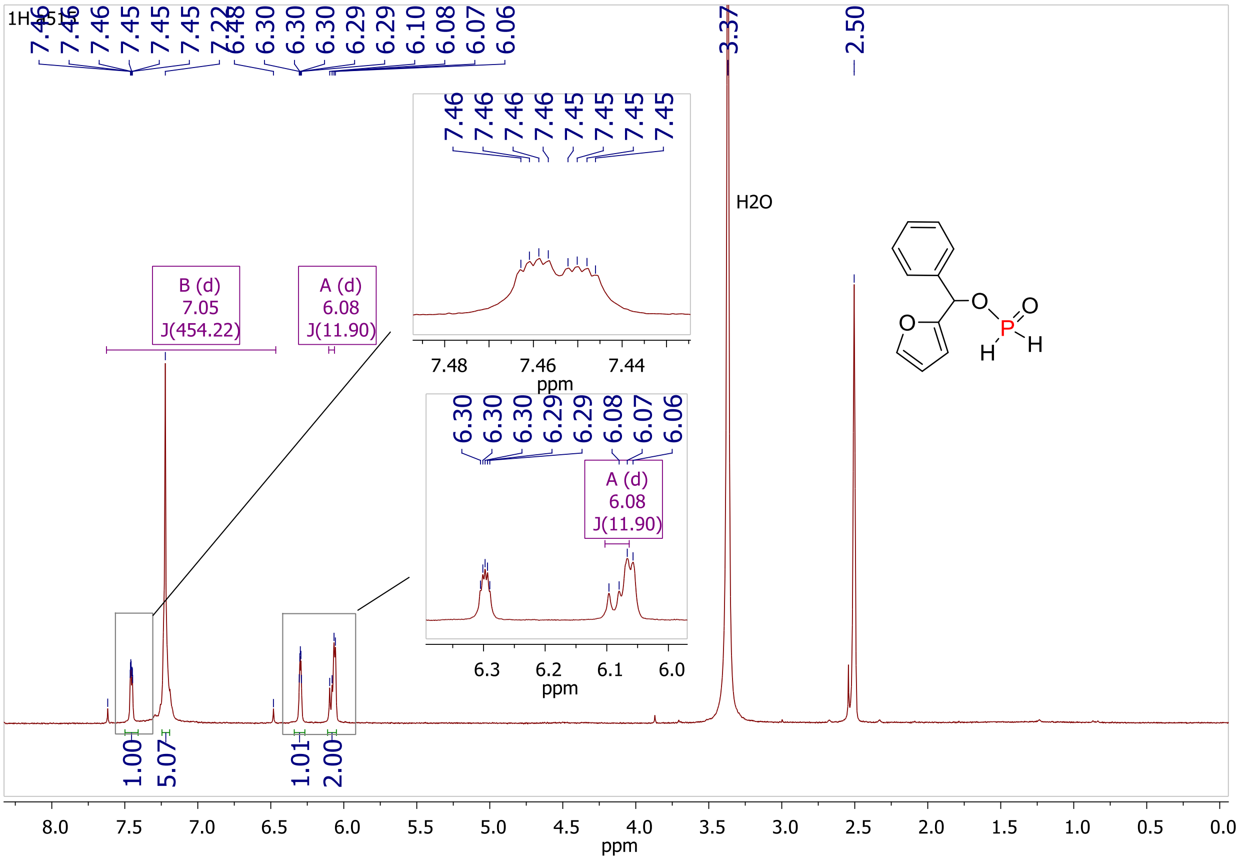

^{1}H NMR spectrum of **2p** (DMSO_d$_6$)

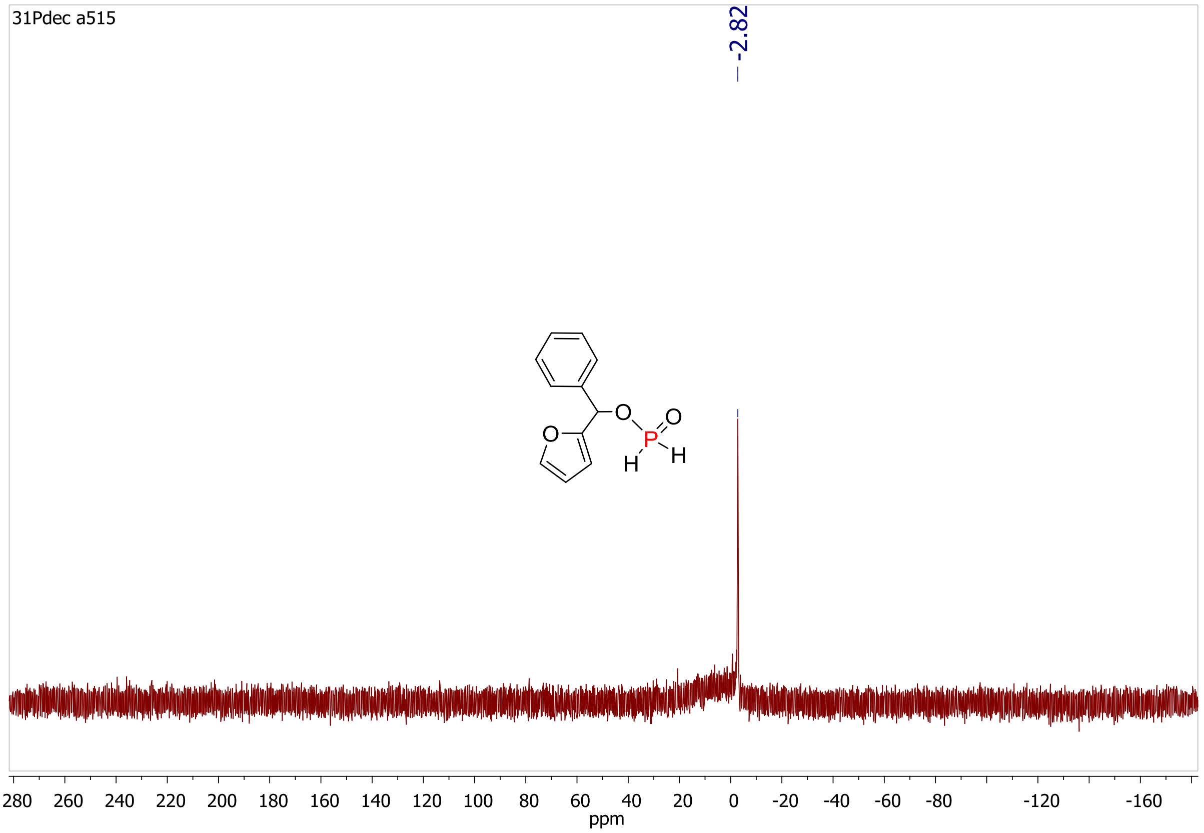

^{31}P{^{1}H} NMR spectrum of **2p** (DMSO_d$_6$).

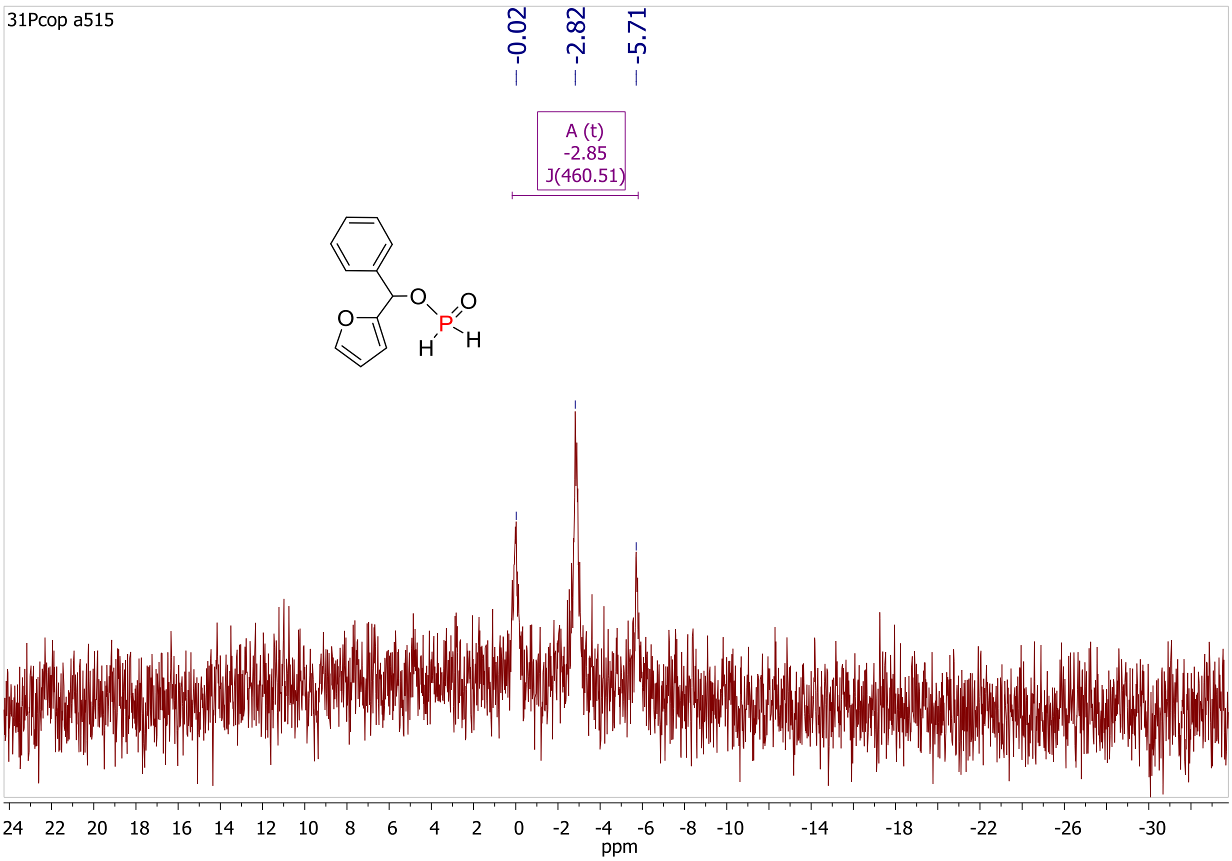

^{31}P NMR spectrum of **2p** (DMSO_d$_6$).

Diphenylcarbinol (3a)

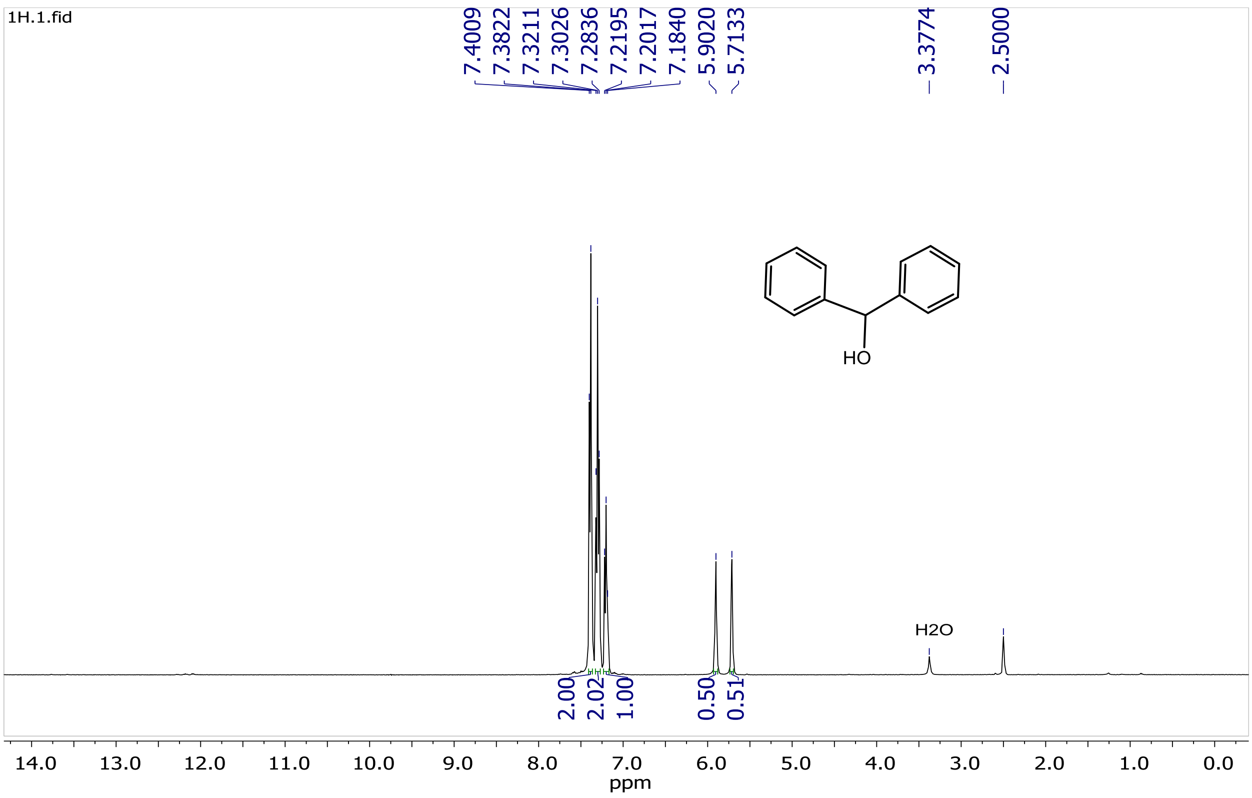

^{1}H NMR spectrum of **3a** (DMSO_d$_6$).

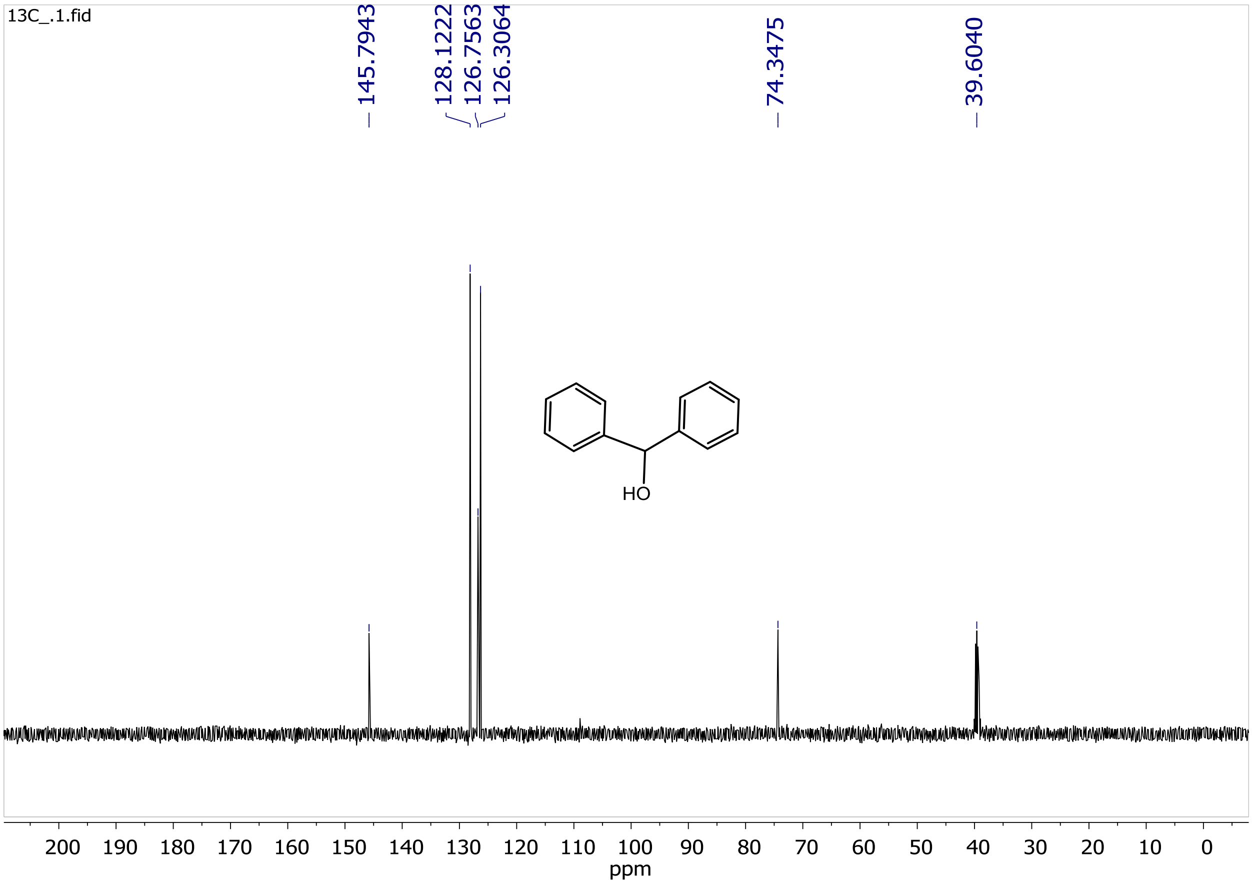

^{13}C NMR spectrum of **3a** (DMSO_d$_6$).

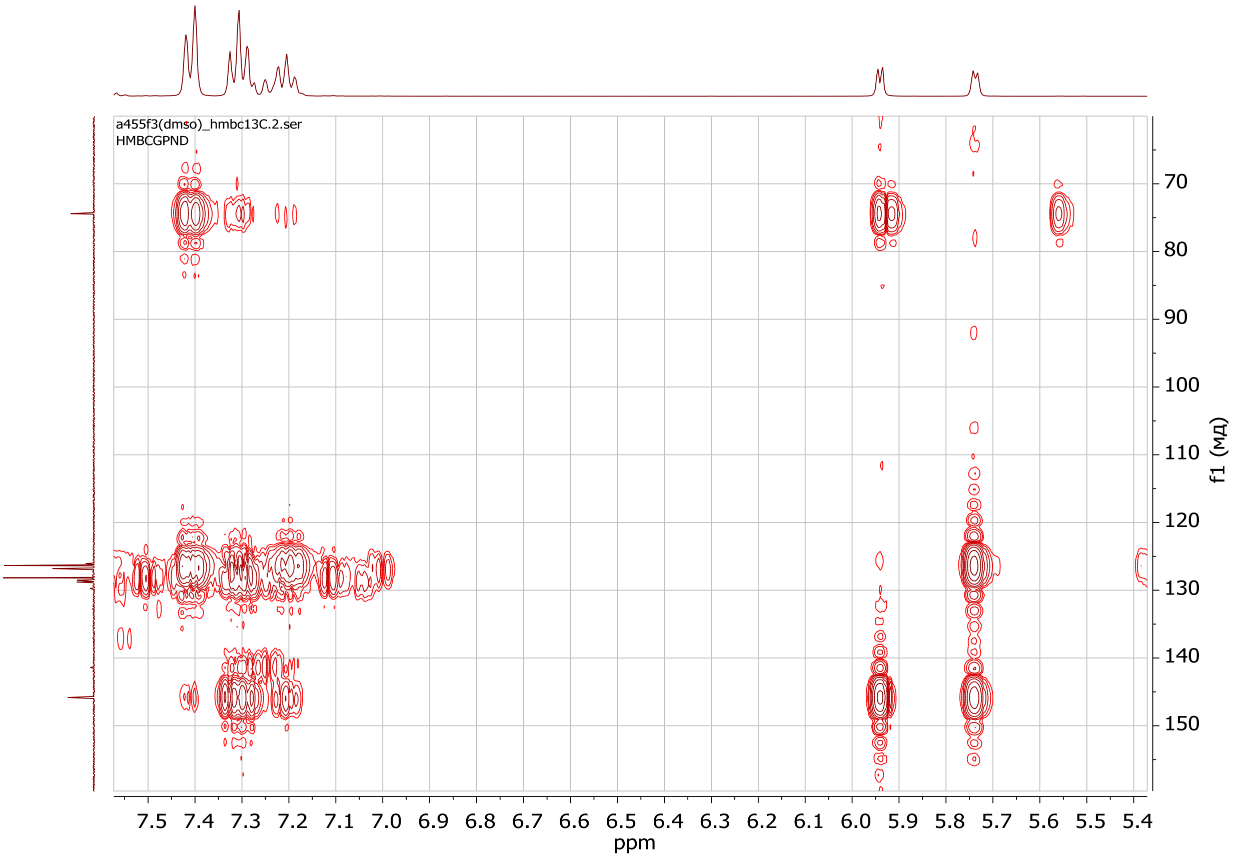

2D HMBC ^{13}C-^{1}H spectrum of **3a** (DMSO_d$_6$)

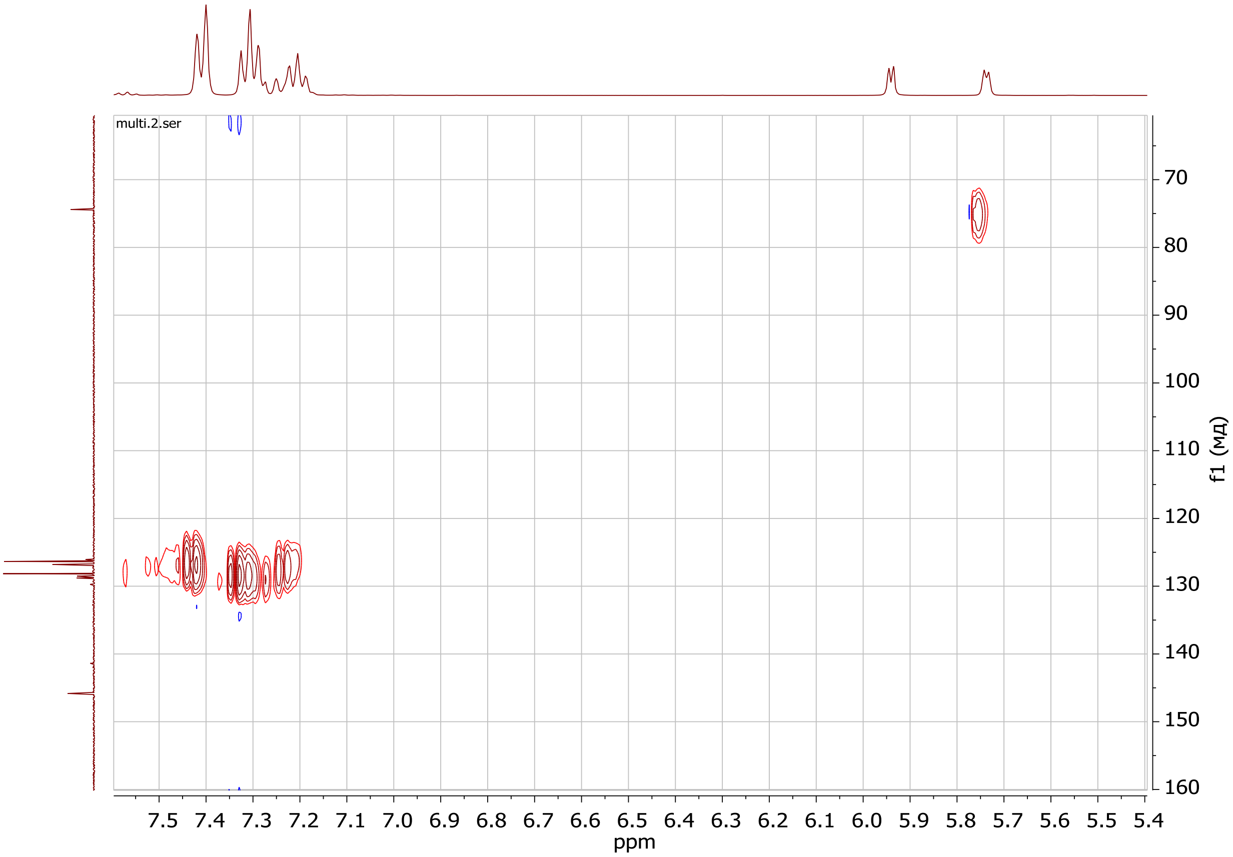

2D HSQC ^{13}C-^{1}H spectrum of **3a** (DMSO_d_6)

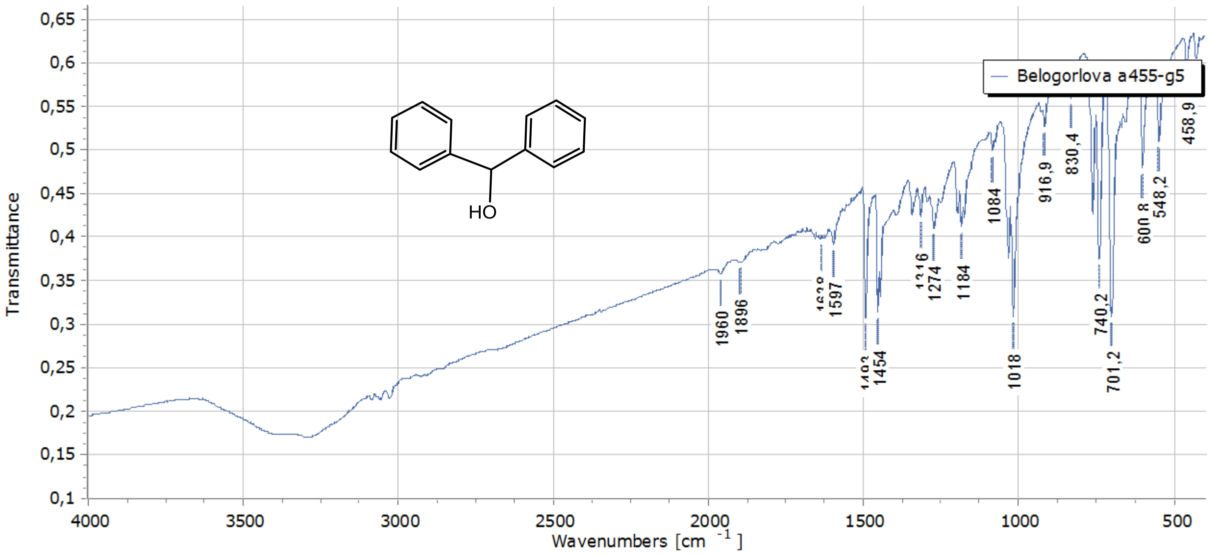

IR spectrum of **3a** (KBr, cm^{-1}).

Dibenzhydryl methyl phosphate (5a)

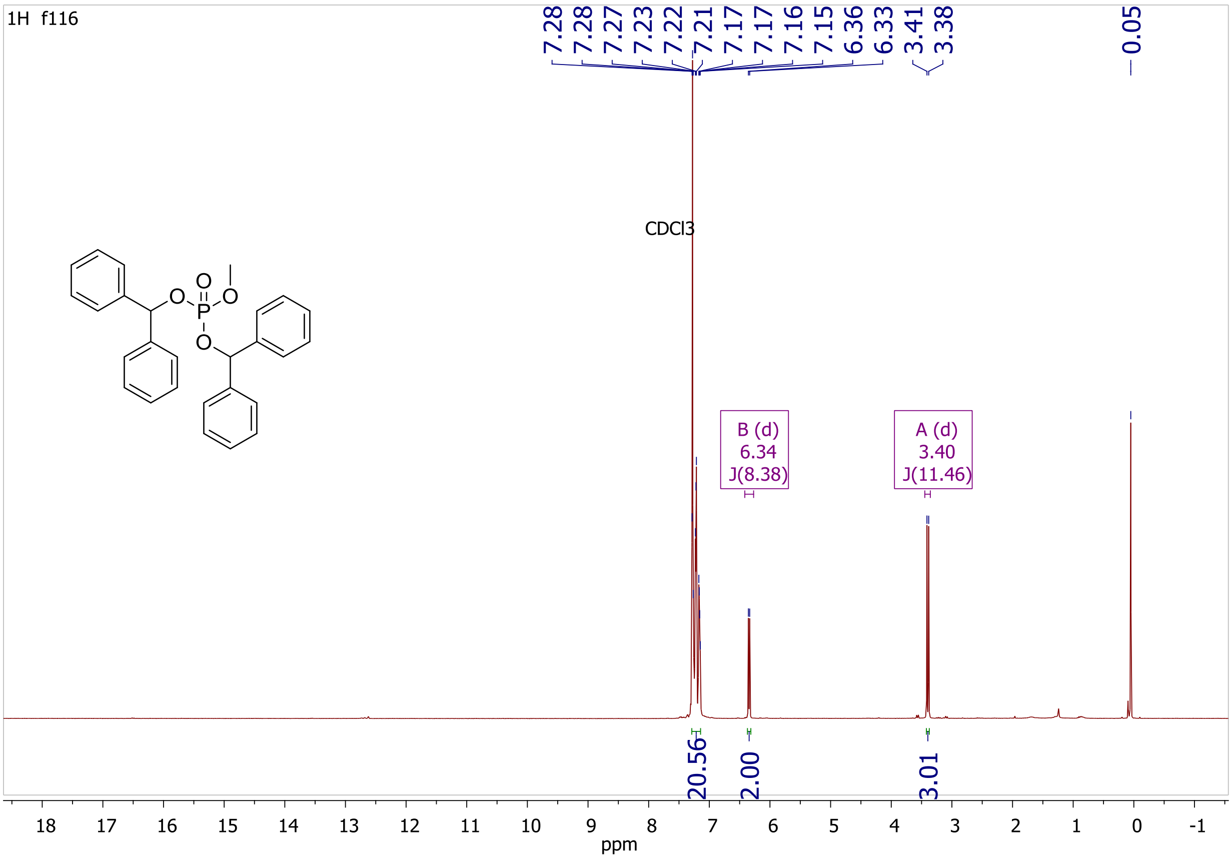

^{1}H NMR spectrum of **5a** ($CDCl_3$).

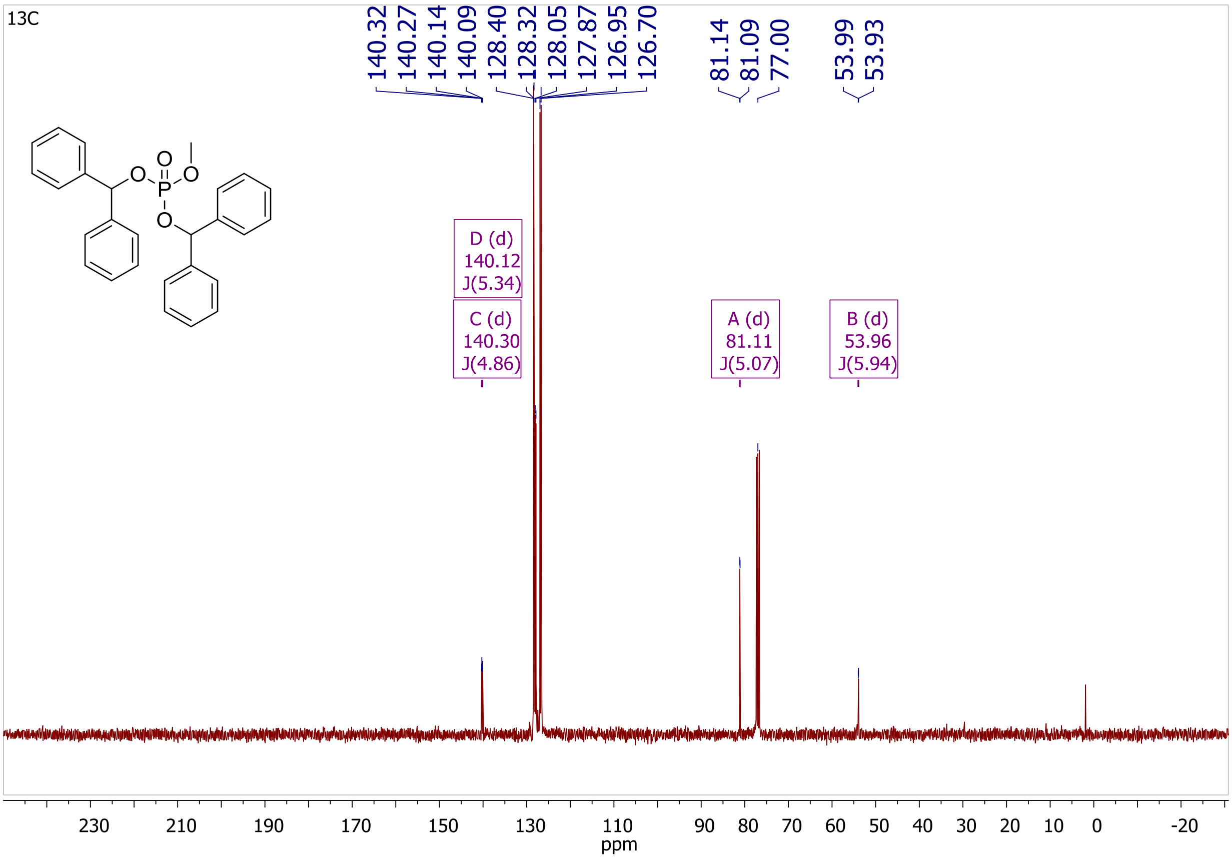

^{13}C NMR spectrum of **5a** ($CDCl_3$).

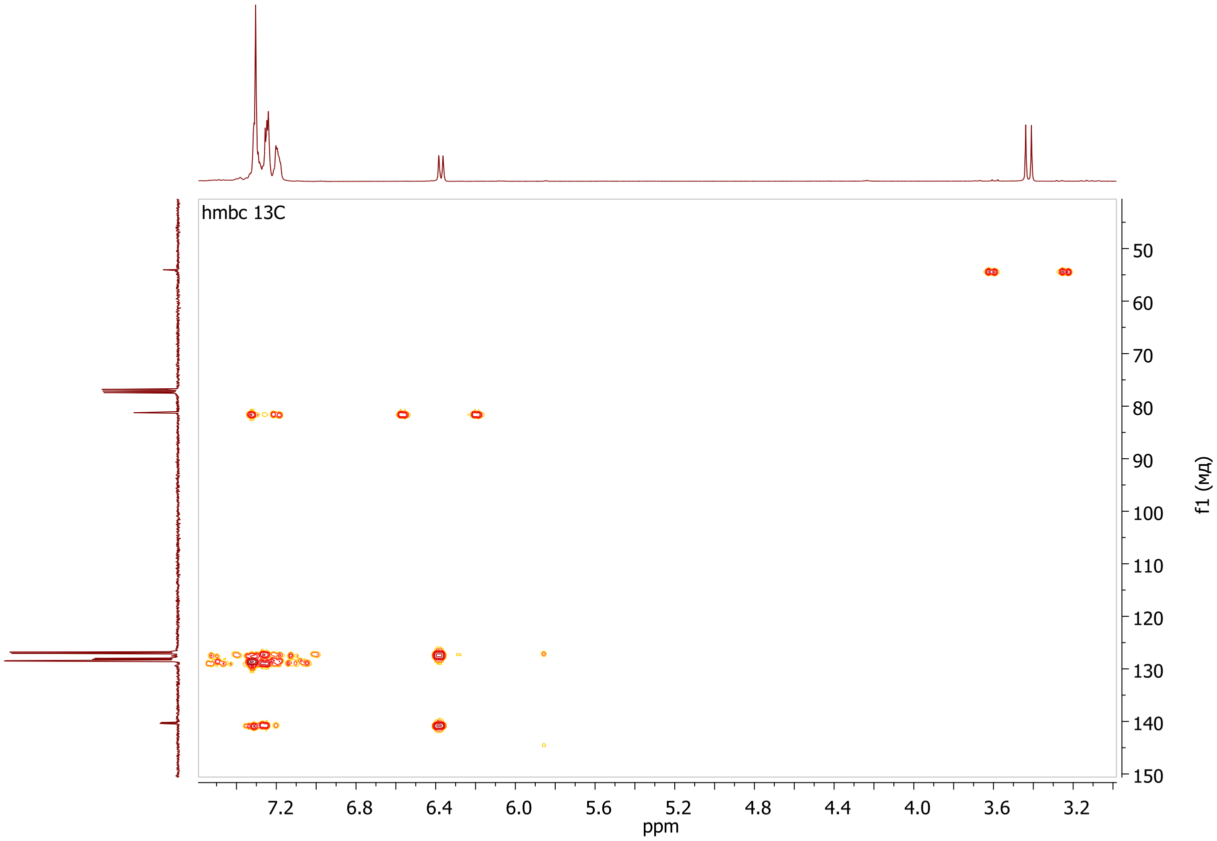

2D HMBC ^{13}C-^{1}H spectrum of **5a** ($CDCl_3$)

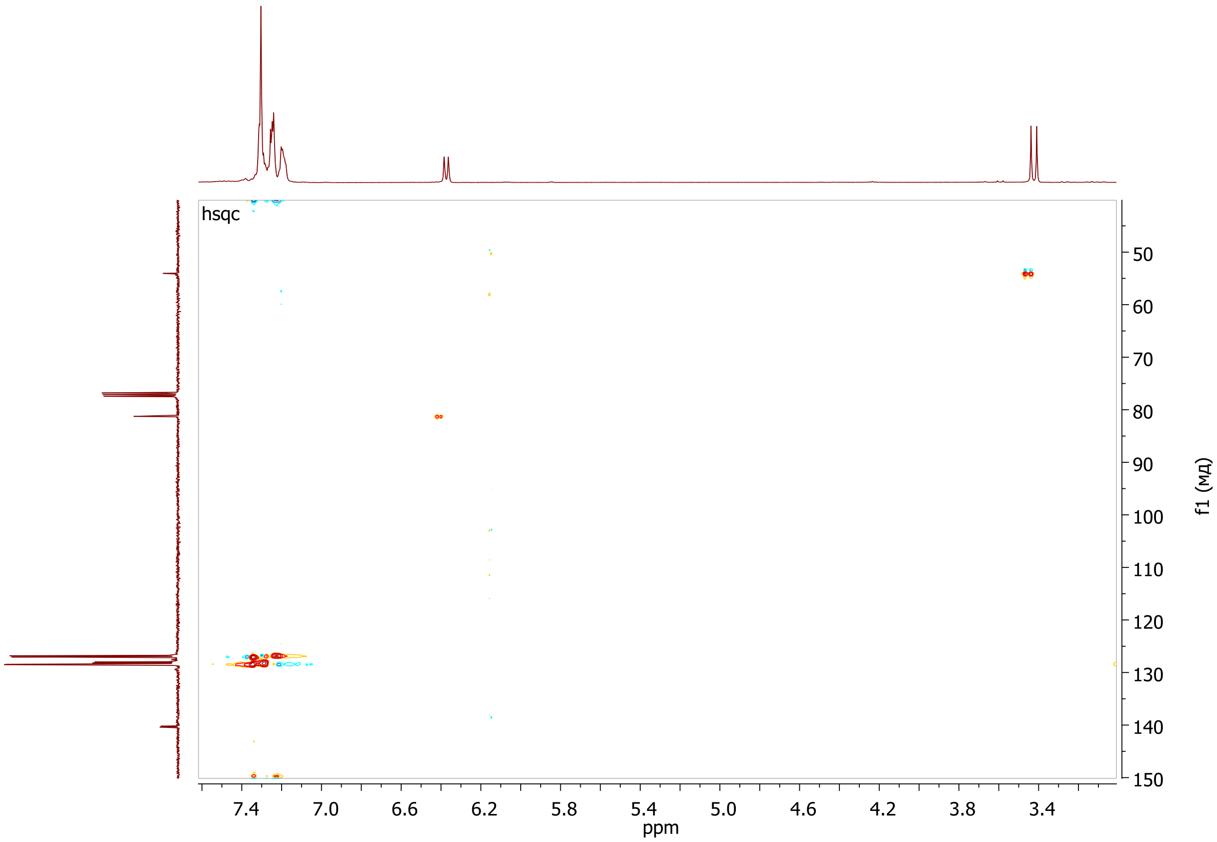

2D HSQC ^{13}C-^{1}H spectrum of **5a** ($CDCl_3$).

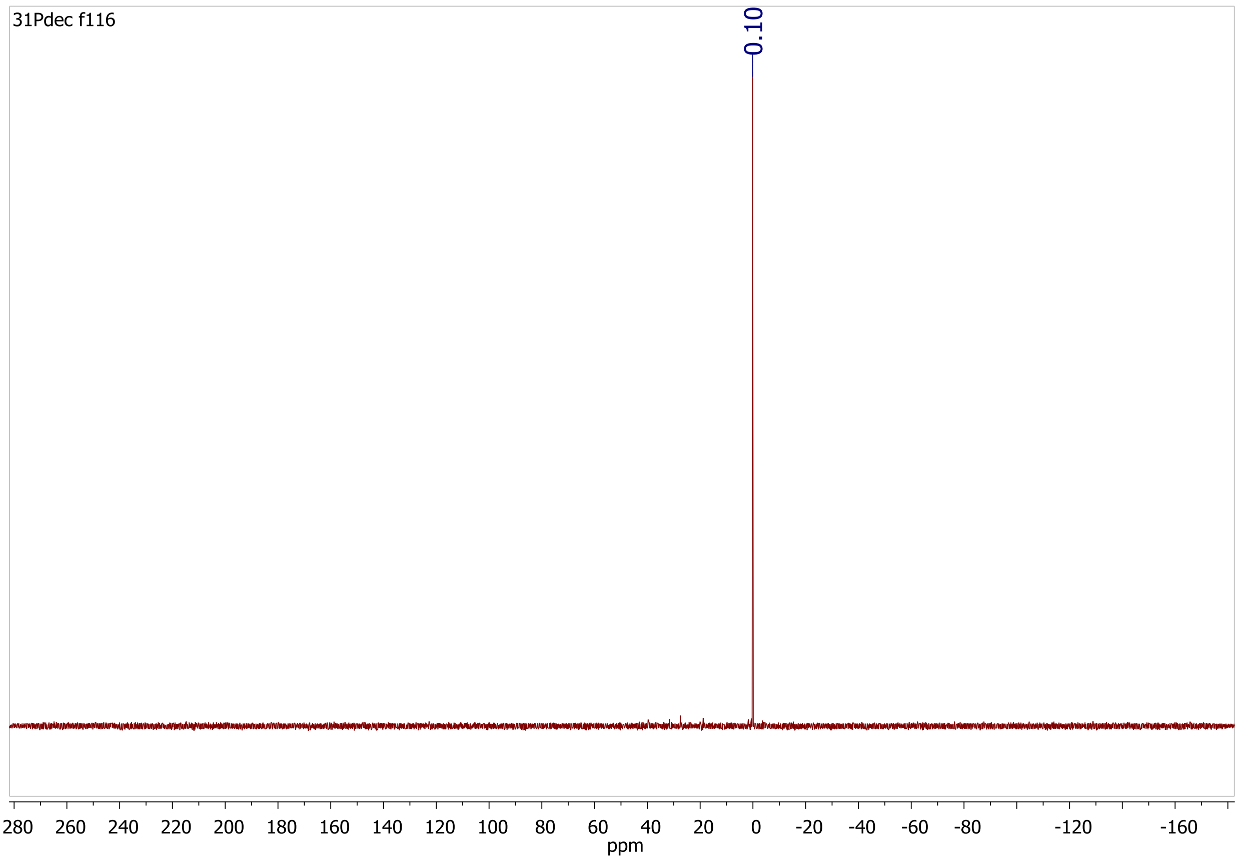

$^{31}P\{^1H\}$ NMR spectrum of **5a** ($CDCl_3$).

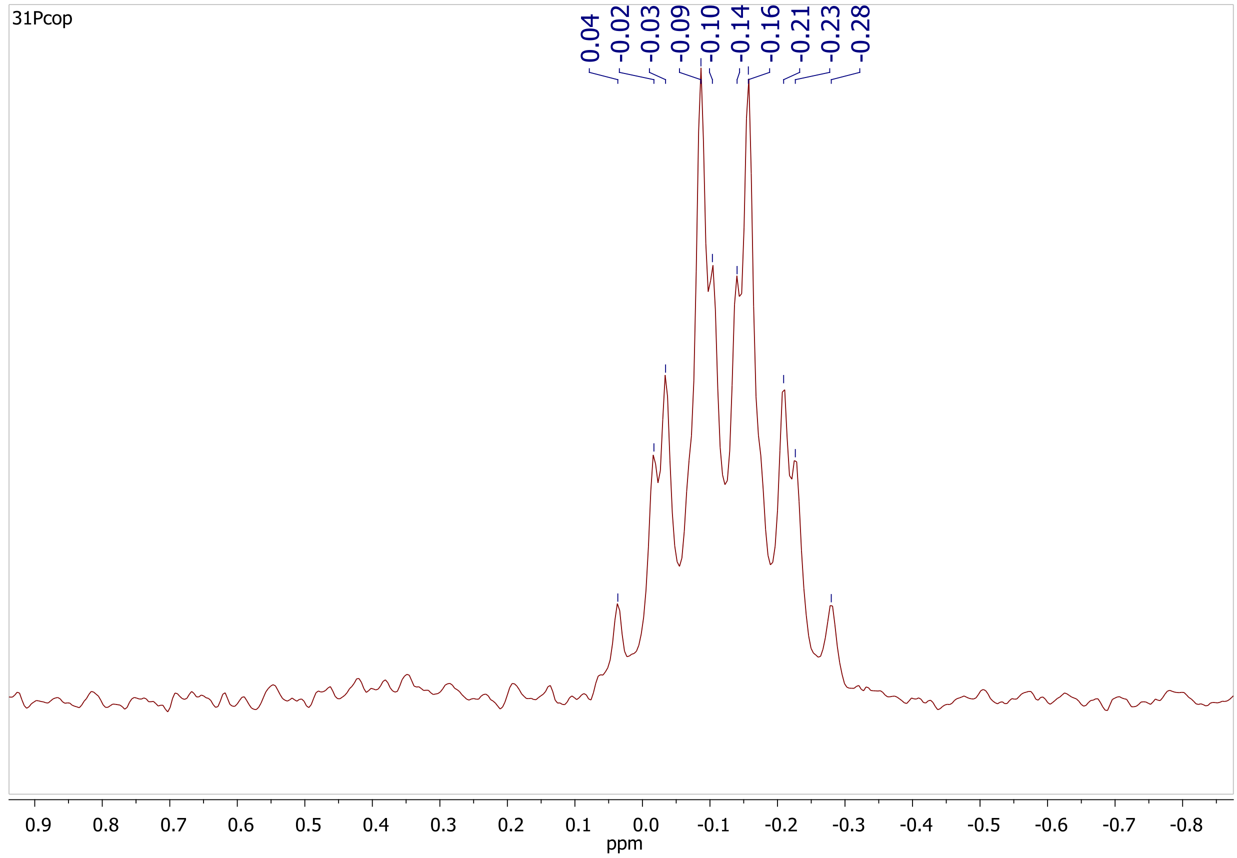

^{31}P NMR spectrum of **5a** ($CDCl_3$).

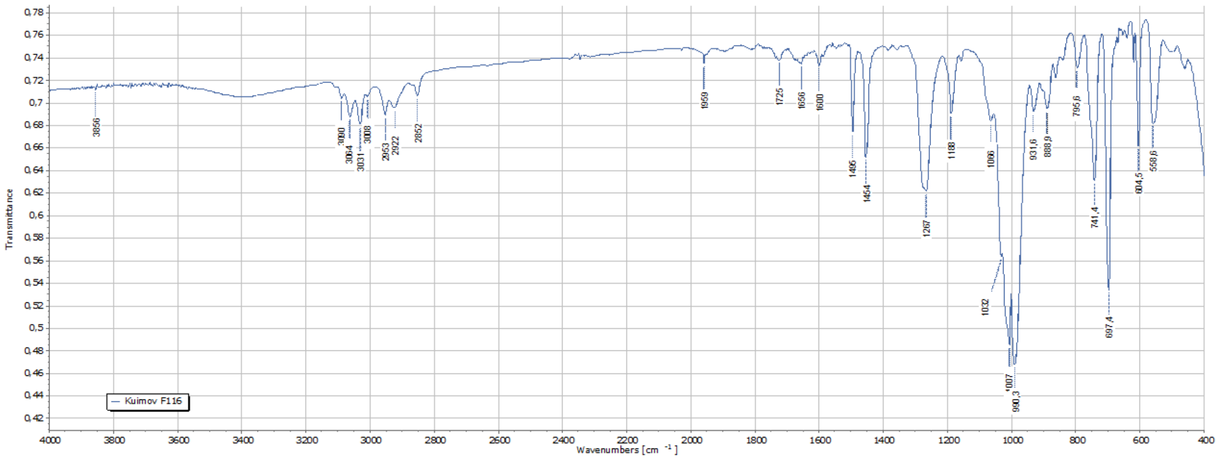

IR spectrum of **5a** (microlayer, cm^{-1}).

7. References

(1) Vogel, W. M.; Routsis, K. J.; Kehrer, V. J.; Landsman, D. A.; Tschinkel, J. G., Physicochemical properties of the potassium hydroxide-water system. Range: 55 to 85 weight % and 120° to 250°C. *Journal of Chemical & Engineering Data* **1967,** *12* (4), 465-472; (b) Abdel-Magid, A. F., Potassium Hydroxide. In *Encyclopedia of Reagents for Organic Synthesis (e-EROS)*, John Wiley & Sons, Ltd.: 2001.

(2) (a) Jeon, K. O.; Jun, J. H.; Yu, J. S.; Lee, C. K., Infrared and nuclear magnetic resonance properties of benzoyl derivatives of five-membered monoheterocycles and determination of aromaticity indices. *J. Heterocycl. Chem.* **2009,** *40*, 763-771; (b) Müller, C.; Gleixner, J.; Tahk, M.-J.; Kopanchuk, S.; Laasfeld, T.; Weinhart, M.; Schollmeyer, D.; Betschart, M. U.; Lüdeke, S.; Koch, P.; Rinken, A.; Keller, M., Structure-Based Design of High-Affinity Fluorescent Probes for the Neuropeptide Y Y1 Receptor. *J. Med. Chem.* **2022,** *65*, 4832-4853.

(3) Asachenko, A. F.; Valaeva, V. N.; Kudakina, V. A.; Uborsky, D. V.; Izmer, V. V.; Kononovich, D. S.; Voskoboynikov, A. Z., Coupling of aromatic aldehydes with aryl halides in the presence of nickel catalysts with diazabutadiene ligands. *Russ. Chem. Bull.* **2016,** *65*, 456-463.

(4) Li, J.; Zhao, J.; Ma, C.; Yu, Z.; Zhu, H.; Yun, L.; Meng, Q., Visible-Light-Driven Oxidative Cleavage of Alkenes Using Water-Soluble CdSe Quantum Dots. *Chemsuschem* **2021,** *14*, 4985-4992.

(5) Meng, M.; Yang, L.; Cheng, K.; Qi, C., Pd(II)-Catalyzed Denitrogenative and Desulfinative Addition of Arylsulfonyl Hydrazides with Nitriles. *J. Org. Chem.* **2018,** *83*, 3275-3284.

(6) Vandavasi, J. K.; Hua, X.; Halima, H. B.; Newman, S. G., A Nickel-Catalyzed Carbonyl-Heck Reaction. *Angew. Chem. Int. Ed.* **2017,** *56*, 15441-15445.

(7) Chodroff, S.; Klein, H. C., Acylation of Benzene Compounds with Iodine as a Catalyst. *J. Am. Chem. Soc.* **1948,** *70*, 1647-1648.

(8) (a) Ghosh, P.; Ganguly, B.; Das, S., Pd-NHC catalysed Carbonylative Suzuki coupling reaction and its application towards the synthesis of biologically active 3-aroylquinolin-4 (1H)-one and acridone scaffolds. *Applied Organometallic Chemistry* **2017,** *32*; (b) Muniyappan, N.; Sabiah, S., Synthesis, structure, and characterization of picolyl- and benzyl-linked biphenyl palladium N-heterocyclic carbene complexes and their catalytic activity in acylative cross-coupling reactions. *Applied Organometallic Chemistry* **2020,** *34*.

(9) Villani, F. J.; King, M. S. 3-Benzoylpyridine *Organic Syntheses* [Online], 1957, p. 6.

(10) Ye, R.; Cao, Y.; Xi, X.; Liu, L.; Chen, T., Metal- and radical-free aerobic oxidation of heteroaromatic methanes: an efficient synthesis of heteroaromatic aldehydes. *Org. Biomol. Chem.* **2019,** *17*, 4220-4224.

(11) Minnis, W., Phenyl Thienyl Ketone. *Organic Syntheses* **1932,** *12*, 62.

(12) D'Vries, R. F.; Grande, C. D.; Chaur, M. N.; Ellena, J. A.; Advincula, R. C., [4-(Allyloxy)phenyl](phenyl)methanone. *Acta Crystallogr., Sect. E: Struct. Rep. Online* **2014,** *70*, o814-o815.

(13) (a) Atherton, F. R.; Howard, H. T.; Todd, A. R., 220. Studies on phosphorylation. Part IV. Further studies on the use of dibenzyl chlorophosphonate and the examination of certain alternative phosphorylation methods. *J. Chem. Soc.* **1948**, 1106; (b) Lowe, G.; Sproat, B. S., A synthesis of adenosine 5′-[β-$^{18}O_2$]triphosphate. *J. Chem. Soc., Perkin Trans. 1* **1981**, 1874-1878; (c) Froussios, C.; Kolovos, M., Preparation of Diphenylmethyl Esters and Ethers of Unprotected Amino Acids and β-Hydroxy-α-amino Acids. *Synthesis* **1987**, *1987*, 1106-1108; (d) Gerrard, W.; Shepherd, B. D., 422. Formation of sulphites, phosphites, and phosphates of alcohols containing an aryl group. *J. Chem. Soc.* **1953**, 2069-2074; (e) Lehmann, H. A.; Grossmann, G., Zum Reaktionsverhalten des P_4-Molekuls und Vorzugsweise seiner in "Langsamer" Reaktion primar gebildeten Reaktionsproudukte mit Oxydationsstufen Zwischen null und drei. *Pure Appl. Chem.* **1980,** *52* (4), 905-915.

(14) (a) Kazakova, V. M.; Lipkind, G. M.; Makarov, I. G.; Shapiro, B. I.; Sirkin, Y. K., EPR study of some derivatives of aromatic ion-radicals. III. Various derivatives of benzophenone. *Radio spectroscopic and quantum chemical methods in structural studies* **1967**, 99-105; (b) Kazakova, V. M.; Sirkin, Y. K., Hyperfine structure of EPR spectra of benzophenone-k-ketyl. *Dokl. Chem.* **1960,** *131*, 346-347; (c) Rieger, P. H.; Fraenkel, G. K., Electron Spin Resonance Spectra of Carbonyl Anion Radicals. *J. Chem. Phys.* **1962,** *37*, 2811–2831; (d) Ayscough, P. B.; Wilson, R., 1033. Electron spin resonance studies of radical anions. Part I. Aromatic ketyls. *J. Chem. Soc.* **1963**, 5412-5417.

(15) (a) Salikhov, K. M., *Electron paramagnetic resonance: From fundamental research to pioneering applications*. AXAS Publishing Ltd.: Wellington, 2009; p 209; (b) Shukla, A., *EMR / ESR / EPR Spectroscopy for characterization of nanomaterials*. Springer India: New Delhi, 2017; p 183.

(16) (a) Milyukov, V. A.; Kataev, A. V.; Sinyashin, O. G.; Hey-Hawkins, E., A new method for the preparation of solution of sodium pentaphosphacyclopentadienide. *Russ. Chem. Bull.* **2006,** *55*, 1297–1299; (b) Jo, M.; Dragulescu-Andrasi, A.; Miller, L. Z.; Pak, C.; Shatruk, M., Nucleophilic Activation of Red Phosphorus for Controlled Synthesis of Polyphosphides. *Inorg. Chem.* **2020,** *59*, 5483-5489; (c) Du, S.; Hu, J.; Chai, Z.; Zhang, W. X.; Xi, Z., Isolation and Characterization of Four Phosphorus Cluster Anions P_7^{3-}, P_{14}^{4-}, P_{16}^{2-} and P_{26}^{4-} from the Nucleophilic Functionalization of White Phosphorus with 1,4-Dilithio-1,3-butadienes. *Chin. J. Chem.* **2018,** *37*, 71-75.

(17) Sheldrick, G. M., Crystal structure refinement with SHELXL. *Acta Crystallogr., Sect. C: Structural Chemistry* **2015,** *71*, 3-8.

(18) *SADABS*, 2008-1; Bruker AXS: Madison, WI, USA, 2008.

(19) (a) Spek, A. L. *PLATON, A Multipurpose Crystallographic Tool*, 10M; Utrecht University, Utrecht, The Netherlands, 2003; (b) Spek, A. L., Single-crystal structure validation with the program PLATON. *J. Appl. Crystallogr.* **2003,** *36*, 7-13.

(20) Macrae, C. F.; Edgington, P. R.; McCabe, P.; Pidcock, E.; Shields, G. P. T., R.; Towler, M.; van de Stree, J., Mercury: Visualization and Analysis of Crystal Structures. *J. Appl. Crystallogr.* **2006,** *39*, 453–457.

(21) Allen, F. H.; Kenard, O.; Watson, D. G.; Bramer, L.; Orpen, A. G.; Taylor, R., Tables of bond lengths determined by X-ray and neutron diffraction. Part 1. Bond lengths in organic compounds. *J. Chem. Soc., Perkin Trans. II.* **1987**, S1-S19.

(22) Deprèle, S.; Montchamp, J.-L., A novel and convenient preparation of hypophosphite esters. *J. Organomet. Chem.* **2002,** *643-644*, 154-163.
